# Molecular Characterization of Influenza C Viruses from Outbreaks in Hong Kong SAR, China

**DOI:** 10.1128/JVI.01051-20

**Published:** 2020-10-14

**Authors:** Rodney S. Daniels, Herman Tse, Burcu Ermetal, Zheng Xiang, Deborah J. Jackson, Jeremy Guntoro, Jérôme Nicod, Aengus Stewart, Karen J. Cross, Saira Hussain, John W. McCauley, Janice Lo

**Affiliations:** a Worldwide Influenza Centre (a WHO Collaborating Centre for Reference and Research on Influenza), The Francis Crick Institute, London, United Kingdom; b Centre for Health Protection, Department of Health, Hong Kong SAR, China; c Advanced Sequencing Facility, The Francis Crick Institute, London, United Kingdom; d Bioinformatics & Biostatistics, The Francis Crick Institute, London, United Kingdom; St. Jude Children's Research Hospital

**Keywords:** influenza C virus, outbreak surveillance, genome sequencing, virus evolution

## Abstract

In 2014, the Centre for Health Protection in Hong Kong introduced screening for influenza C virus (ICV) as part of its routine surveillance for infectious agents in specimens collected from patients presenting with symptoms of respiratory viral infection, including influenza-like illness (ILI). A retrospective analysis of ICV detections up to week 26 of 2019 revealed persistent low-level circulation, with two outbreaks having occurred in the winters of 2015 to 2016 and 2017 to 2018. These outbreaks occurred at the same time as, and were dwarfed by, seasonal epidemics of influenza types A and B. Gene sequencing studies on stored ICV-positive clinical specimens from the two outbreaks have shown that the hemagglutinin-esterase (HE) genes of the viruses fall into two of the six recognized genetic lineages (represented by C/Kanagawa/1/76 and C/São Paulo/378/82), with there being significant genetic drift compared to earlier circulating viruses within both lineages. The location of a number of encoded amino acid substitutions in hemagglutinin-esterase fusion (HEF) glycoproteins suggests that antigenic drift may also have occurred. Observations of ICV outbreaks in other countries, with some of the infections being associated with severe disease, indicates that ICV infection has the potential to have significant clinical and health care impacts in humans.

**IMPORTANCE** Influenza C virus infection of humans is common, and reinfection can occur throughout life. While symptoms are generally mild, severe disease cases have been reported, but knowledge of the virus is limited, as little systematic surveillance for influenza C virus is conducted and the virus cannot be studied by classical virologic methods because it cannot be readily isolated in laboratories. A combination of systematic surveillance in Hong Kong SAR, China, and new gene sequencing methods has been used in this study to assess influenza C virus evolution and provides evidence for a 2-year cycle of disease outbreaks. The results of studies like that reported here are key to developing an understanding of the impact of influenza C virus infection in humans and how virus evolution might be associated with epidemics.

## Abstract (precis)

Influenza C virus infection of humans is common, and reinfection can occur throughout life. While symptoms are generally mild, severe disease cases have been reported, but knowledge of the virus is limited, as little systematic surveillance for influenza C virus is conducted and the virus cannot be studied by classical virologic methods because it cannot be readily isolated in laboratories. A combination of systematic surveillance in Hong Kong SAR, China, and new gene sequencing methods has been used in this study to assess influenza C virus evolution and provides evidence for a 2-year cycle of disease outbreaks. The results of studies like that reported here are key to developing an understanding of the impact of influenza C virus infection in humans and how virus evolution might be associated with epidemics.

## INTRODUCTION

Influenza C virus (ICV) was first isolated in 1947 in the United State from four individuals aged 17 to 21 years (1). Seroprevalence studies for ICV have shown high rates, with the virus being distributed worldwide in the human population and initial exposure (infection) occurring during childhood (2–8), while recurrent infection with this virus occurs in both children and adults (9). Overall, as summarized in Atkinson et al. (10), seropositivity has been reported to be between 57 and 100%, being lower in children and rising in adulthood. ICV has been considered a pathogen of low significance, as it generally causes mild disease or clinically inapparent infection (9, 11, 12), but in recent years it has become increasingly apparent that the virus can be associated with severe disease, notably in children with lower respiratory tract infections (12–18).

Despite the high seroprevalence rates in the human population, taken as an indicator of widespread infections, historical diagnosis and epidemiological studies that would have relied on ICV isolation were hampered by low isolation rates in hens’ eggs (19) and in mammalian cell culture, despite a study in pediatric patients that showed an isolation rate of 0.51% from 36,973 clinical specimens which was comparable to detections by real-time reverse transcription-PCR (rt RT-PCR) (7), and propagation to a high titer has proved difficult (15, 20–22). With the advent of molecular techniques, notably rt RT-PCR and gene sequencing, epidemiologic and virologic studies have become possible but are still limited, as no country apart from Japan (23) has conducted long-term monitoring/surveillance of ICV. The studies that have been performed largely relate to retrospective research-related projects focusing on ICV detections, mainly by rt RT-PCR, in specific countries or states within a country (9, 16, 17, 22, 24–30). Such studies have focused on genetic characterization of the hemagglutinin-esterase (HE) gene that encodes the hemagglutinin-esterase fusion (HEF) glycoprotein, which combines the functions of the hemagglutinin (HA) and neuraminidase (NA) of influenza type A and B viruses and is the major antigenic component of ICVs (31). Studies have shown that HE genes fall within six genetic lineages (32) that correspond to antigenic groups (7) with antigenic sites identified previously (33–35). Of the other six RNA segments of the ICV genome, four are monocistronic, encoding the proteins of the polymerase complex (three polymerases, PB2, PB1, P3, plus nucleoprotein [NP]), whereas the matrix (MP) and nonstructural (NS) genes encode two proteins each, namely CM1 and CM2 and NS1 and NS2, respectively; reviews of their genetics and functions are available (31, 36). While analyses of the other six gene segments of ICVs have been limited, evidence for genetic reassortment has been presented for viruses circulating from 1947 to 2014 (23, 37).

Here, we present an analysis of ICV surveillance in Hong Kong, which has been conducted since the beginning of 2014, backed up by gene sequencing directly from ICV-positive clinical specimens, thereby avoiding any potential adaptation during attempts at virus isolation in either hens’ eggs or mammalian cell culture.

## RESULTS

### Detection of ICV infections in Hong Kong.

**FIG 1:**
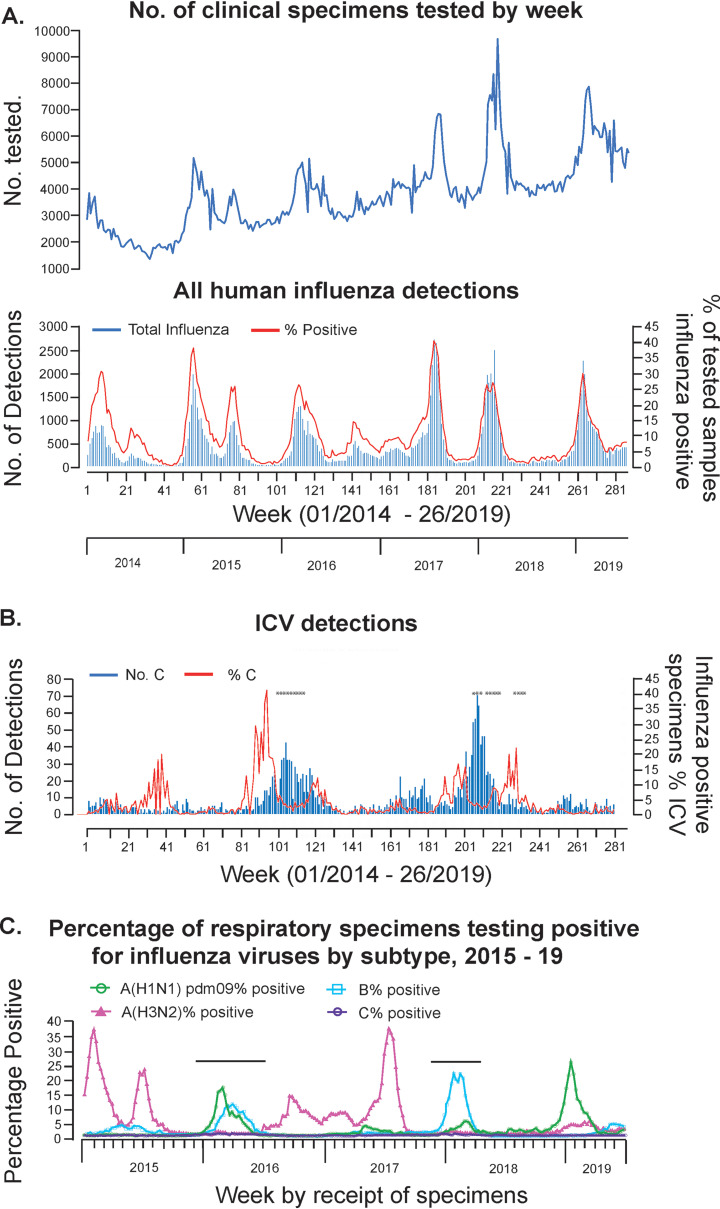
ICV surveillance in Hong Kong 2014 to 2019. Results of weekly influenza surveillance in Hong Kong covering the period from week 01 of 2014 to week 26 of 2019 are shown for (A) all human influenza virus detections in terms of numbers tested, numbers detected, and the percentage of tested specimens showing positivity and (B) ICV detections by number and percentage of the total number of influenza detections. Clinical specimens from the weeks indicated (*) were analyzed by gene sequencing. (C) For the period from week 01 of 2015 to week 26 of 2019 the proportions of influenza types/subtypes detected by week are shown with the ICV outbreak periods indicated above the virus detection profiles. All data were taken from *Flu Express* reports published by the Centre for Health Protection, Hong Kong, and panel C is reproduced from reference 68.

Since the start of 2014, the Centre for Health Protection in Hong Kong SAR, China (CHP-HK), had used rt RT-PCR to screen for ICV in specimens taken from patients presenting with influenza-like illness (ILI) symptoms, as well as those with clinical features of viral respiratory infections. Persistent low-level circulation of ICVs was observed with detections of 165, 231, 653, 479, 733, and 133 cases (total *n* = 2,394) on an annual basis from 2014 to 2019 (to week 26 for 2019), respectively (Fig. 1A and B). Over this time, a total of 1,088,090 specimens were tested, giving an ICV detection rate of 0.22%. A majority of the detections were made in the course of two outbreaks which took place in the winters of 2015 to 2016, when A(H1N1)pdm09 and type B viruses were dominating, and 2017 to 2018, when type B (Yamagata-lineage) viruses with lower levels of A(H1N1)pdm09 dominated (Fig. 1C).

Over the course of the two outbreaks, week 49 of 2015 to week 27 of 2016 and week 45 of 2017 to week 15 of 2018, there were 661 and 719 ICV detections (total *n* = 1,380/2,394, i.e., 56.7% of the total to week 26 of 2019), respectively, based on rt RT-PCR (Table 1). Over these time periods, totals of 117,132 and 126,095 specimens were tested, giving ICV detection rates of 0.54% and 0.56%, respectively. In no week did the ICV detection rate exceed 0.96% (week 5 of 2018). In both outbreaks, the number of male patients outnumbered that of female patients, and detections were spread across different age ranges but with great majorities, 72% and 68%, respectively, occurring in those aged up to 5 years. The apparent male preponderance among ICV-positive cases is of interest, given that in Hong Kong, there is a female predominance in the population structure (https://www.censtatd.gov.hk/hkstat/sub/so20.jsp). However, further studies on the epidemiologic aspects of ICV infection would be required to address the significance of the observed male/female case ratios.

**TABLE 1:** Characteristics of ICV outbreaks in Hong Kong

Outbreak yrs	Duration (wks)^*a*^	No. of detections	Gender (no. of patients)		No. of patients (% of total no. of detections) in age range (yrs) of:				
			Male	Female	≤5	6–15	16–45	46–65	>65
2015–2016	wk49/2015 to wk27/2016 (32)	661	364	297	474 (71.7)	43 (6.5)	41 (6.2)	40 (6.1)	63 (9.5)
2017–2018	wk45/2017 to wk15/2018 (23)	719	415	304	486 (67.6)	61 (8.5)	63 (8.8)	44 (6.1)	65 (9.0)

a Duration relates to the span of weeks when there were ≥10 detections/week; the number of weeks is shown in parentheses. wk49/2015, week 49 of 2015, etc.

### ICV-positive samples shared with WHO Collaborating Centre, London.

Four virus isolates, one each from 2008, 2009, and 2011 (Kanagawa lineage) and one from 2018 (São Paulo S1 sublineage), and 98 clinical specimens from the course of the two outbreaks were shared for analysis by newly developed Sanger sequencing (HE gene) and next-generation sequencing (NGS) (whole-genome) protocols. The clinical specimens were from 47 females and 51 males and fell across the following spectrum of age ranges (in years): up to 5 (*n* = 52), 6 to 15 (*n* = 12), 16 to 45 (*n* = 13), 46 to 65 (*n* = 8), and >65 (*n* = 13). For 88 of the patients, a clinical diagnosis at the time of specimen collection was given, with 20 patients being admitted to the hospital for various reasons (e.g., sepsis, cardiac conditions, gastric problems, leukemia, loss of consciousness, and/or drug overdose) with no indicated signs of respiratory conditions; these patients were in the age ranges (in years) of 0 to 5 (*n* = 5), 6 to 15 (*n* = 1), 16 to 45 (*n* = 6), 46 to 65 (*n* = 3), and >65 (*n* = 5). The other 68 patients all presented with respiratory conditions, although not all would have fulfilled ILI classification criteria; these patients were in the age ranges (in years) of 0 to 5 (*n* = 41), 6 to 15 (*n* = 11), 16 to 45 (*n* = 5), 46 to 65 (*n* = 4), and >65 (*n* = 7).

### ICV HE gene sequencing.

**FIG 2:**
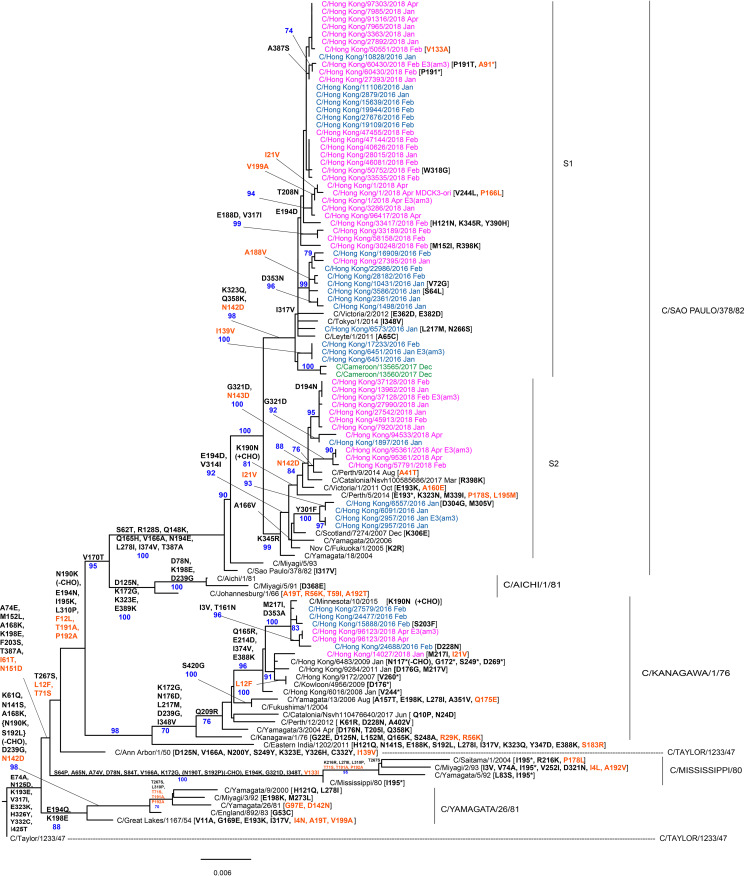
Phylogenetic analysis of ICV HE genes. The phylogeny was generated as described in Materials and Methods, and reference sequences from different clades were downloaded from the EpiFlu Database of GISAID. Viruses from the 2015 to 2016 and 2017 to 2018 outbreaks in Hong Kong are shown in blue and pink, respectively, and other viruses sequenced at WHO Collaborating Centre, London, are shown in green. Group-defining amino acid substitutions are shown on nodes, together with bootstrap values of 70 and above, and virus specific substitutions are shown after virus names, with HEF2 substitutions being in orange and those affecting N-linked glycosylation indicated (±CHO). Bar indicates the proportion of nucleotide changes between sequences. We gratefully acknowledge the authors and originating and submitting laboratories of the sequences from the EpiFlu Database of GISAID that were downloaded for use in the preparation of this study (all submitters of data may be contacted directly via the GISAID website [http://gisaid.org/] and the relevant sequence accession numbers are given in Table S5 in the supplemental material).

HE gene sequences encoding full-length HEF glycoproteins were obtained for the four viruses and 25/44 (55.6%) clinical specimens relating to the 2015 to 2016 outbreak and 35/54 (64.8%) relating to the 2017 to 2018 outbreak (see Table S1 in the supplemental material). Phylogenies including the Hong Kong ICV sequences and representative (Fig. 2) and complete (see Fig. S1D in the supplemental material) sets of HE sequences available in Global Initiative on Sharing All Influenza Data (GISAID) are shown. Both phylogenies support, with strong bootstrap values, the six lineages previously designated (23, 38, 39). The phylogenies show that viruses from both outbreaks were spread between the C/Kanagawa/1/76 and C/São Paulo/378/82 lineages, but with the majority (68%) falling into a sublineage (S1) of the latter represented by C/Tokyo/1/2014 and carrying amino acid substitutions of K323Q and Q358K in HEF1 and N142D in HEF2, with others (22%) falling into a sublineage (S2) defined by K345R in HEF1 and represented by C/Fukuoka/1/2005 (Fig. 2). Both sublineages are supported by strong bootstrap values, and sublineage S2 splits into two groups based on A166V substitution (represented by C/Scotland/6855/2007) and K190N substitution (represented by C/Catalonia/1318/2009) in HEF1 (Fig. S1D). Sequences of virus isolates from Hong Kong in 2007, 2008, 2009, and 2011 all fell within the C/Kanagawa/1/76 Lineage.

**FIG 3:**
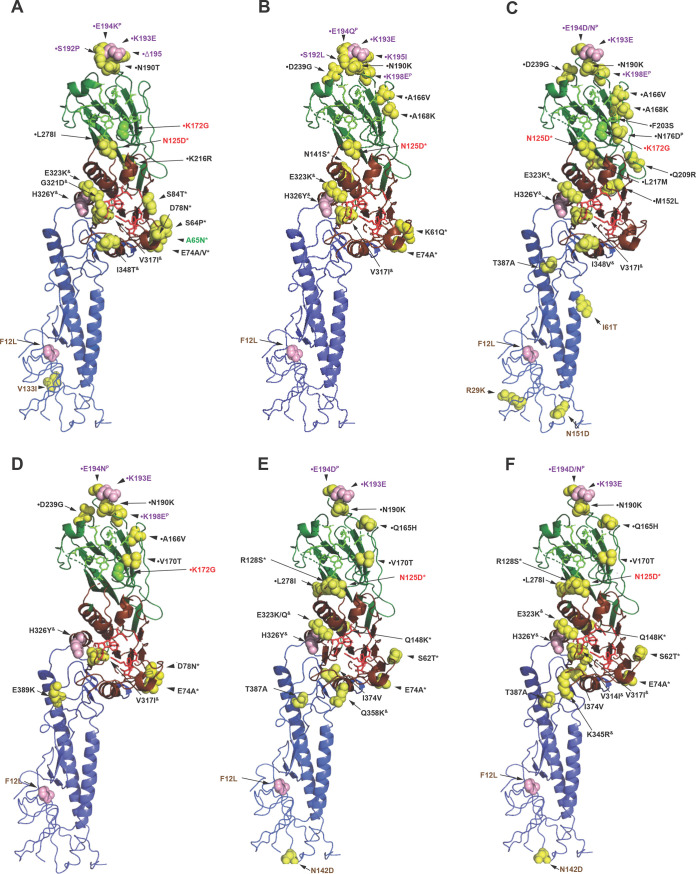
HEF structures indicating the amino acid substitutions that define the genetic/antigenic lineages. The structure of an HEF monomer derived from C/Johannesburg/1/66 (51) was downloaded from the Protein Data Bank (PDB identifier 1FLC) and annotated using PyMOL. Amino acid substitutions (compared to HEF of C/Taylor/1233/47) that define later genetic/antigenic lineages are shown as follows: (A) Mississippi, (B) Yamagata, (C) Kanagawa, (D) Aichi, (E) Sao Paulo (C/Tokyo/1/2014, S1 sublineage), and (F) Sao Paulo (C/Fukuoka/1/2005, S2 sublineage). Each cartoon shows the receptor-binding domain (HEF1) in green, the esterase domain (HEF1) in red and the fusion domain (HEF2) in blue. Residues involved directly in receptor binding are shown as bright green sticks, and those involved directly in esterase activity as bright red sticks. Amino acid substitutions are shown as spheres, color-coded as follows: pink, substitutions conserved across all six groups; yellow, lineage-defining substitutions; bright green, substitution (K172G) at a residue involved directly in receptor binding (which is found in Mississippi, Aichi, and Kanagawa lineages only). Amino acid substitutions are color-coded as follows: brown, HEF2; black, HEF1 with those in designated antigenic sites indicated in green (site A2), red (site A1), and purple (site Y1), with substitutions at HEF1 positions 193 and 198 forming parts of sites A1 and A3, respectively (40). Amino acid substitutions falling in esterase domain 1 (*), esterase domain 2 (^&^), and the receptor-binding domain (•) are indicated (51), as are those under positive selection (^P^) (39).

Mapping of residues associated with neutralization-sensitive epitopes on HEF, possibly associated with antigenic drift, has been performed with a small panel of murine monoclonal antibodies raised against either C/Ann Arbor/1/50 (Taylor lineage) or C/Yamagata/15/2004 (Yamagata lineage) (40). Figures 2 and 3 show lineage-defining amino acid substitutions in HEF1 and clearly identify residues identified by Matsuzaki et al. (40). Based on node-defining substitutions in Fig. 2, lineages after Taylor carry K193E; Mississippi lineage carries A65N, N125D, K172G, S192P, E194K and Δ195; Yamagata lineage, N125D, N190K, S192L, E194Q, K195I, and K198E; Kanagawa lineage, N125D, K172G, N190K, K190N, E194D, E194N, K198E, F203S, and D353A; Aichi lineage, K172G, N190K, E194N, and K198E; São Paulo sublineage S1, N125D, N190K, and E194D; and São Paulo sublineage S2, N125D, N190K, K190N, E194D, and D194N. A number of individual viruses carry HEF amino acid substitutions at these positions (see “tip” labels in Fig. 2), and there are numerous other substitutions that map in the vicinities of the residues that lie within neutralization-sensitive epitopes (see Fig. S2A in the supplemental material). Amino acid substitutions at these additional positions possibly reflects antigenic drift in humans, which may exert somewhat different antibody selection pressures on ICV HEF than that in mice.

Minority variants in the HE gene sequences generated for samples from Hong Kong, representing those found in less than 20% of sequence reads, are indicated in Table S2 in the supplemental material. Overall, just 490 nucleotide variants were identified, as follows: 158 synonymous, 293 nonsynonymous, and 39 indels (37 causing frame shifts and two that would result in 3-amino-acid insertions). The 90 variants in the HEF1 coding region were located at 68 nucleotide positions that involved 59 codons, with 69 (77%) of the variant calls being nonsynonymous. The 400 variants in the HEF2 coding region were located at 90 nucleotide positions involving 70 codons, with 263 (66%) being nonsynonymous. Broad ranges of variant frequencies were observed for synonymous and nonsynonymous variants in both HEF1 and HEF2 coding regions (overall range, 1.0 to 19.9%), with 77 of the variant calls having frequencies of ≥10%. Of these 77, 38 clustered at codon positions 449 (*n* = 18 [27%] of 67, V → A), 452 (*n* = 15 [19%] of 78, G → E/K/R), and 453 (*n* = 5 [83%] of 6, I → V) in HEF2 and encoded the amino acid substitutions indicated, with there being a total of 151 variant calls across these three codons.

### ICV non-glycoprotein-encoding gene sequencing.

Recovery of nucleotide sequences encoding full-length open reading frame protein products for the other six genes from Hong Kong samples was generally good for virus isolates (*n* = 11) but variable for clinical specimens (*n* = 98) (Table 2). Recoveries of the shortest genes (MP and NS) were comparable to that for HE, while recoveries of genes encoding components of the polymerase complex (PB2, PB1, P3, and NP) averaged around 30%, but with PB1 recovery of only 19%.

**TABLE 2:** ICV “internal” gene recovery from Hong Kong samples^*a*^

Gene segment	No. of full-length genes recovered		
	Total	Clinical specimen^*b*^	Virus
PB2	42	32 (33)	10
PB1	29	19 (19)	10
P3	43	32 (33)	11
NP	47	36 (37)	11
MP	63	52 (53)	11
NS	72	61 (62)	11

a Gene rescue was attempted for 98 clinical specimens and 11 virus isolates.

b Percent recoveries relative to the 98 clinical specimens analyzed are shown in parentheses.

Phylogenetic analyses of the eight open reading frames (PB2, PB1, P3, NP, CM1, CM2, NS1, and NS2) within the six genes, relative to the HE lineage where known, are shown in Fig. S1. All proteins show numbers of amino acid substitutions, with CM1 showing the fewest, along the trunks of the phylogenies associated with evolution from the Taylor lineage to the currently circulating Kanagawa (K) and São Paulo (S1 and S2) lineages, as follows: PB2—L657F, S387N, and S436T (K, S1, S2) and V289I (S1, S2); PB1— I181V, D76N, S88G, I202V, V518I, and K59E (K, S1, S2) or K119R and N380S (S1 subgroup); P3—T55A, S58N, I708T, E193K, V359I, N178D, and V330I (K, S1, S2); NP—T555A and E74D (K, S1, S2) or A298T (S1 subgroup); CM1—T189A and K96R (S1 subgroup); CM2—K32N (K, S1, S2) with Y14C, T18I, and I13M (S1) or either A39E or I63V (S2); NS1—F91L, and Q212R with L91P, N226D (S1), or G88E (S2) or L241I (K, S1); NS2—K108E, L142H, K76R, Y162H, and T170I (K, S1, S2) with R76K, H162N, and T128A or S116L or D70G (S1), or with T39A (S2, K) or V34I (S2) or R76K (S2) or N86S (K). The functional significance of any of these substitutions, together with those in the proteins of individual viruses, is unknown.

Bayesian methods for phylogenetic tree building and substitution rate estimation have been used to study the molecular evolution of ICV and have shown that gene reassortment plays a significant role in the generation of viruses with specific gene constellations, thereby increasing genetic diversity; these viruses may have relatively short lifespans due to further reassortment (23, 37). The virus names in the maximum likelihood phylogenies shown in Fig. S1 are annotated with the HE lineage, where known, using orange symbols, with symbols for viruses that fall outside lineage groupings being colored green. Viruses with genes with green symbols against their names are considered to be reassortants, and the inferences contained in Fig. S1 are summarized in Table 3. Fifteen of the 58 viruses represented in Table 3, including the lineage prototype A/Kanagawa/1/76, appear to be triple reassortants; 33 viruses were reassortants based on polymerase complex gene reassortment involving one to four of the genes (PB1, PB2, P3, and NP); 21 were reassortants involving the MP gene and 24 involved the NS gene, with 11 involving both MP and NS; 11 of the reassortants involving the MP gene and nine of those involving the NS gene appeared to show within-segment recombination, possibly through their internal splice sites, with one of the viruses (C/Eastern India/1202/2011) showing evidence of recombination in both segments. Two viruses sequenced in the course of this study, C/Hong Kong/14027/2018 (K) and C/Hong Kong/33189/2018 (S1), appear to be reassortant in their MP genes, with C/Hong Kong/14027/2018 also being reassortant in its NS gene. This level of reassortment is considerably lower than that found in a study from Japan covering the period from 1990 to 1999, when viruses of the Aichi/1/81, Yamagata/26/81, and Mississippi/80 lineages were the dominant ICVs in circulation (41).

**TABLE 3:**
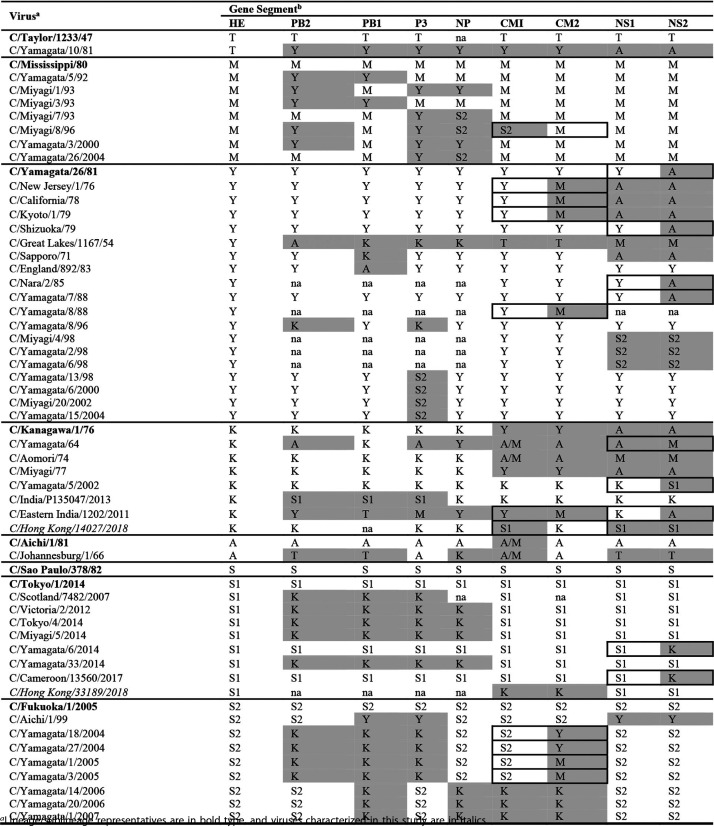
ICV reassortants based on HE lineage

a Lineage/sublineage representatives are in bold type, and viruses characterized in this study are in italics.

b HE lineage/sublineage indicated as follows: T, Taylor; M, Mississippi; Y, Yamagata; K, Kanagawa; A, Aichi; S, Sao Paulo (S1, S2); mismatches compared to the HE lineage/sublineage are highlighted. For MP (CM1 and CM2) and NS (NS1 and NS2), within gene fragment recombination events are boxed. na, full-length open reading frame sequence not available. For each virus, we elected to relate everything back to the HE lineage, as assigned in Fig. S2D in the supplemental material, when looking for reassortants and the way in which the other six genes grouped according to the HE lineage (Fig. S2A to C and E to I). Using this method, a number of the HE lineage representative viruses, notably C/Kanagawa/1/76, are clearly reassortants themselves. Only two viruses from the recent outbreaks in Hong Kong, both from the 2018 outbreak, appear to be reassortant.

### Virus isolation.

Seven of eight clinical samples passaged in the amniotic cavity of chicken eggs yielded low hemagglutination titers (4 to 8 hemagglutination units). The seven isolates all yielded good gene sequences with no significant amino acid substitutions in HEF compared with the respective clinical specimens (Fig. 2). Attempts to increase virus yields to allow antigenic analysis by passaging virus from third amniotic passages in both the allantoic cavity of 10-day-old embryonated eggs and in Madin-Darby canine kidney (MDCK) cell culture were unsuccessful.

## DISCUSSION

Seropositivity for ICV is as high as 90% in children by 7 to 10 years of age, indicating that it is a major cause of acute respiratory illness that goes undiagnosed due to presentation as mild “cold-like” symptoms in the vast majority of cases (42, 43), with only more severe cases seeking medical assistance and even fewer being tested for the virus (43). This, linked with low isolation rates for ICV, has meant there have been relatively few viruses available for study, and consequently, while numbers of HE gene sequences have been adequate, small numbers of full-genome sequences have been available for analysis (38).

Outside of Japan, little ICV surveillance has been conducted in an ongoing systematic way, but in 2014 the Centre for Health Protection in Hong Kong introduced ICV testing as part of routine surveillance for viral respiratory infectious agents. Over the period of January 2014 to June 2019, in excess of 1.08 million human respiratory specimens were tested for ICV, resulting in detection of 2,394 cases, a detection rate of 0.22%, with 1,380 (57.6%) of these being associated with two outbreaks in the winters of 2015 to 2016 and 2017 to 2018. The age distribution of cases in both outbreaks was similar, with 78% and 76%, respectively, being in children aged up to 15 years of age and with the remainder spread fairly evenly across the other three age groups, but with ∼9% being in adults aged 65 years or older in both outbreaks (Table 1). These observations in the adult population are likely to represent reinfections.

Here, we report a retrospective next-generation sequencing study for archived clinical specimens from these two outbreaks which attempted to recover full-genome (full-length open reading frame) sequences. At least one full-length open reading frame gene sequence was recovered from 63/98 (64%) clinical specimens, and analyses conducted in this study focus on these sequences and on similar full-length sequences downloaded from the EpiFlu Database of GISAID.

The great majority of ICV studies have focused on the HE gene and its product, HEF glycoprotein, which is the major virus component targeted by an antibody response. We confirm that ICV can be classified into six lineages based on the HE gene (Fig. 2), consistent with a previous report (32), although it is apparent that four lineages (C/Taylor, C/Mississippi, C/Yamagata, and C/Aichi) have died out and the C/São Paulo lineage has split into two sublineages. Others have reported the splitting of the São Paulo HE lineage into two, described here as S1 (representative C/Tokyo/1/2014) and S2 (representative C/Fukuoka/1/2005), which are not distinguishable with polyclonal immune sera but are distinguishable with anti-HEF monoclonal antibodies in hemagglutination inhibition assays (44). All HE gene sequences from the two outbreaks in Hong Kong cluster within the C/Kanagawa and C/São Paulo lineages, with the majority (68%) falling in the S1 (C/Tokyo/1/2014) sublineage.

Figure 3 indicates HEF amino acid substitutions that have occurred during the evolution of the five lineages after C/Taylor. These substitutions largely map in and around the receptor-binding site and at locations shown to be in neutralization-sensitive epitopes; thus, it is likely that positive selection for evasion of the host immune system operates in ICVs (37, 40). However, the designation of neutralization-sensitive epitopes (A-1 to A-4 and Y-1, with there being overlap between A-1 and Y-1) is largely based on selection of virus variants that escape neutralization by murine monoclonal antibodies raised against viruses from the C/Taylor and C/Yamagata lineages. Of the amino acid substitutions indicated in Fig. 3, those at residues 125, 172, 193 (A-1 site), 65 (A-2 site), 198 (A-3 site), 192, 193, 194, 195, and 198 (Y-1 site) have been identified in neutralization escape variants (40). Other substitutions associated with lineage emergence, which may have resulted from human immune pressure, map close to these residues. In a study of HE codon usage, it was observed that HEF1 residues 176, 194, and 198 were under positive selection, possibly in response to antibody pressures (39).

It was shown in 1984 that N-linked glycosylation of hemagglutinin (HA) on influenza A(H3N2) viruses could prevent binding of neutralizing antibody (45). Since the pandemic of 1968, the number of utilized N-linked glycosylation sequons in the globular head of HA of A(H3N2) viruses has steadily increased, with variation in the location of the sequons, such that there are at least 33 sequons in a functional HA trimer (46). While addition of these sequons has been associated with alterations in receptor binding, they have not interfered with overall virus fitness but have been associated with escape from human antibody responses (47). The sequons (NXS/T, where X can be any amino acid but P [48]) in HEF of human ICV isolates show remarkable conservation (Fig. S2A). Compared with C/Taylor/1233/47 all other virus lineages showed early loss of the proline-containing sequon (NPS) at HEF1 positions 190 to 192, four due to N190K amino acid substitution, while the Mississippi lineage did so due to N190T and S192P substitution (Fig. 2). However, the sequon has been regenerated by K190N substitution in an individual virus (C/Great Lakes/1167/54, Yamagata lineage) and relatively recently in C/Minnesota viruses of the Kanagawa lineage and viruses from a number of countries that fall into the São Paulo C/Catalonia/1318/2009 group. The substitutions at positions 190 to 192 fall within a neutralization-sensitive epitope (A-1/Y-1) identified by selection of virus variants that escape neutralization by murine monoclonal antibodies (40). Three viruses showed loss of glycosylation sequons; two in the Kanagawa lineage at positions 117 to 119 due to HEF1 N117Y substitution (C/Singapore/Dso-070193/2006) and N117N/S polymorphism (C/Hong Kong/6483/2009) and one in the C/Catalonia/1318/2009 group due to HEF2 N106N/S polymorphism (C/Bretagne/2503/2013).

Overall, the substitutions shown in Fig. 3 can be considered to map to five locations on the HEF1 head around positions 172/176, 193, 74, 278, and 323, which equate to antigenic sites A to E in influenza A H3 hemagglutinin (HA), with site B, which is equivalent to sites A-1/Y-1 in ICV HEF, located at the membrane-distal end in both H3-HA and HEF being immunodominant (40, 49, 50). From a structure/function point of view, the ICV esterase domain has been shown to involve residues 41 to 150 and 311 to 366 of HEF, while the receptor-binding domain sits above the esterase domain and involves residues 151 to 310 (51). Fig. 3 shows that during evolution of the ICV lineages there have been 11 (positions 62 to 148), eight (positions 314 to 358) and 20 (positions 165 to 278) amino acid substitutions, respectively, in these three domains. That substitutions occur at these sites in HEF indicates that there are probably subtle antigenic differences between the six ICV lineages, with evolution likely being driven by human antibody pressure targeting the esterase and receptor-binding sites. In this context, in a recent study based on 148 young to elderly adults (up to 90 years of age) in 2014 and 2015, while there was a 77% (99 of 129 blood samples) seropositivity rate, only 1.4% (2 of 148 upper respiratory tract samples, from asymptomatic individuals aged 51 and 70) were rt RT-PCR positive for ICV (10). Host immune pressure directed at the HEF glycoprotein is likely to be a major driver of HE gene evolution, and identification of minor variants may give some idea of the direction of evolution. Table S2 in the supplemental material shows minor variants from consensus sequences supported by at least 10 reads, which yielded frequencies of between 1.0 and 19.9%, for HE genes of Hong Kong viruses sequenced at the Worldwide Influenza Centre (WIC). Of 47 viable nonsynonymous variants (i.e., those that would introduce neither out-of-reading-frame shifts nor stop codons) in the HEF1 coding domain, 29 encode amino acid substitutions at positions either directly implicated as or close to those identified as forming parts of antigenic sites A1, A2, and A3 (40). In addition, minor variants encoding substitutions at one or more HEF1 glycoprotein positions, 165 (H165L), 167 (T167S), and 169 (G169E/D), were observed only in egg isolates of C/Hong Kong/1/2018 (S1), C/Hong Kong/60430/2018 (S1), C/Hong Kong/37128/2018 (S2), and C/Hong Kong/95361/2018 (S2) within the receptor-binding domain (51). These may represent egg adaptation substitutions like those that have been well documented for influenza A and B viruses (52). Furthermore, the clustering of minor variants at HEF2 glycoprotein positions 20 to 24 (VEAGI), V20A, G23E/K/R, and I24V is notable, as the introduction of charged amino acids in the proximity of the fusion peptide has been shown to destabilize influenza A hemagglutinin (53).

It has been shown that there is less than a 2-fold difference in mean nucleotide substitution rate across the different genes of ICV, and these rates are of a similar magnitude to those for influenza B but an order of magnitude lower than for influenza A, which may be related to humans being the natural host for type B and C viruses (37).

During a 9-year survey period (1990 to 1999) in Sendai City, Japan, involving children of less than 15 years of age, ICVs were detected as outbreaks over a 4-month winter/spring period in almost every year, and evidence was provided that supports replacement of dominant HEF antigenic groups, at least in restricted geographic areas (41). The antigenic group replacement corresponded with switching between viruses that fell within three virus lineages that appear to have now died out (Fig. 2), A/Aichi/1/81, C/Yamagata/26/81, and C/Mississippi/80. Antigenic differences between the recently circulating viruses within a lineage were minimal compared to viruses of the same lineage that had circulated previously. This same study reported a very high level of gene reassortment among 44/45 (97.8%) ICV isolates based on nearly full-length gene sequencing for HE, MP, and NS and fragments of the four genes (PB2, PB1, P3, and NP) encoding the polymerase complex, suggestive of cocirculation of the three lineages with frequent coinfection. Consequently, the authors suggested that ICV evolved a reassortment pathway to allow continued spread in humans because functional constraints on HEF glycoprotein, the major antigenic component of the virus, restrict its ability to escape immune pressure exerted by humans, which may be of relatively short duration against viruses of a particular lineage (41).

Our study of Hong Kong SAR, China, at a time when viruses of the C/Kanagawa/1/76 and C/São Paulo/3781/82 lineages were circulating, focusing on full-genome sequencing with complete open reading frames for all gene segments, was successful for 9/11 virus isolates (one lacked PB2 and one PB1) but only for 18/63 clinical specimens (see Table S1 in the supplemental material). Two partial genomes from clinical specimens, C/Hong Kong/14027/2018 (K) and C/Hong Kong/33189/2018 (S1), showed reassortment involving MP and NS genes from K and S1 lineage viruses with the MP of C/Hong Kong/14027/2018 (K), showing evidence for recombination, possibly through the MP gene splice site, resulting in CM1 (S1) and CM2 (K) products (Table 3). In the case of influenza A viruses, the host splicing machinery is hijacked for processing of MP and NS segments, with the segments displaying high levels of similarity and secondary structure to human consensus splice sites (54). Similar potential reassortment and recombination events involving ICV lineages that appear to have disappeared were observed in viruses from earlier years based on full-length gene open reading frame sequences downloaded from GISAID. Such reassortant viruses may have an increased ability to spread between humans compared to their parental viruses. In a more recent study from the same Japanese group, spanning the years 1947 to 2014, it was proposed that some genome constellations might be advantageous and result in ICV epidemics (23), as was observed in our analysis of data collected in Hong Kong. In this context, in Japan at least, reemergence of the C/Kanagawa lineage was detected in 1996 and it continued to circulate together with C/São Paulo lineage viruses, which emerged in 2004 and spread widely, throughout the study period and beyond. Our observation of ICV outbreaks occurring 2 years apart in Hong Kong is notable given the evidence of biennial ICV epidemics in Japan (7), with similar trends having been indicated in studies from other countries, as reviewed in Sederdahl and Williams (43). The occurrence of biennial epidemics is suggestive of herd immunity temporarily curtailing ICV circulation after each peak of transmission and an inability of the HEF glycoprotein to accommodate frequent antigenic drift substitutions in light of functional constraints. In support of this, it has been suggested that replacement of a dominant antigenic group may be caused by immune selection within older children and/or adults in the community, with replacement often involving a switch in ICV lineage and the lineages being antigenically distinguishable (7). We do not know which amino acid substitutions in the HEF glycoprotein are potential markers for causing an epidemic but presume that this will depend on the levels of population immunity. However, by drawing analogies with influenza A and influenza B viruses we can postulate that epitopes in the vicinity of the receptor-binding site are likely to be good targets for neutralizing antibody while retaining sufficient flexibility to allow retention of fitness by antibody-resistant mutant viruses.

While ICV infection is generally viewed as a mild disease of children, there are numerous reports showing that reinfection can occur, even in later life, and an increasing number that describe severe disease in children with associated economic burden (reviewed in references 10 and 43). This evolving understanding of ICV epidemiology related to the prevalence and recurrence of multiple lineages, together with potential cross-species transmission, raises public health concern. Pigs have been considered as an animal reservoir for ICV zoonotic transmission. Genomes of human and pig ICVs were compared by two-dimensional (2D) oligonucleotide mapping and found to be similar but not identical, and proteins of human ICVs isolated over a 35-year period (1947 to 1981) and those of pig ICVs isolated in 1981 and 1982 were highly conserved (55, 56). The question of whether or not pigs are a natural reservoir for ICVs and a source of zoonotic transmissions could not be answered, but later analyses led to the suggestion that interspecies transmission of ICV between humans and pigs is occurring, possibly in both directions (57). Exploring codon usage of human and pig ICV isolates showed that pigs exerted a stronger evolutionary pressure on ICVs than did human hosts and led the authors to suggest that humans are the primary reservoir (39). Due to the paucity of sequence information available for nonhuman ICVs, hard conclusions cannot be drawn, but based on the HE gene phylogenies incorporating sequences from nonhuman ICVs, it would appear that C/bovine/Montana/12/2016 represents a reverse zoonotic event followed by diversification in cattle (see Fig. S1B and C in the supplemental material). Sequences available for influenza D viruses are all from swine/bovine sources, indicative of no human cases having been detected and recorded (see Fig. S2C in the supplemental material).

Given the potential risk of more virulent strains emerging, there is need for continued, and possibly increased/improved, ICV surveillance along the lines of that employed in Hong Kong, where the great majority of specimens come from hospitalized patients. Given the latter caveat and the high ICV seroprevalence, it is likely that the great majority of ICV infections diagnosed will be from severe cases which can be considered the “tip of the iceberg” in relation to infection in the general population, although an increase in severe case numbers would provide an early warning of potential epidemics/pandemics. To be prepared for such an event, it would be beneficial to develop antivirals and vaccines targeting ICVs. Vaccination against ICV is a possible public health option, but burden of disease estimates in all age groups is required before development of such a policy could be recommended.

Studies like those described here that provide a detailed molecular analysis of ICVs in circulation that cause outbreaks or epidemics are key to understanding the impact of ICVs and how their evolution might be associated with epidemics, information critical to the development of health policies. Furthermore, increased surveillance in nonhuman species (particularly those which are farmed extensively and intensely) for ICV and other potential zoonotic agents would be beneficial, as indicated by the last influenza A pandemic caused by A(H1N1)pdm09 following a zoonotic event involving pigs with the first cases being identified in Mexico (58) and by the recently declared COVID-19 pandemic (59).

## MATERIALS AND METHODS

### Respiratory sample collection and screening in Hong Kong SAR, China.

The Centre for Health Protection in Hong Kong SAR, China (CHP-HK), conducts daily testing (by nucleic acid detection) and storage of respiratory specimens in relation to influenza surveillance, as indicated on page 8 of their specimen handbook (https://www.chp.gov.hk/files/pdf/grp-specimenhandbook-en-2004122802.pdf). Clinical specimens from patients with symptoms of respiratory viral infections, including influenza-like illness (ILI), received from various sources, public and private hospitals, and clinics, with the great majority coming from hospitalized patients (https://www.itc.gov.hk/en/quality/hkas/doc/scopes/801P.pdf), are tested for infectious agent detection/diagnosis and characterization (https://www.chp.gov.hk/en/statistics/data/10/641/642/2274.html). The influenza-like illness classification used is that patients should have fever (>38°C) with cough and/or sore throat, as indicated on page 4 of the sentinel partner recruitment document (https://www.chp.gov.hk/files/pdf/letter_to_private_doctors_recruitment_of_new_sentinel_partners.pdf). All specimens are screened for a variety of respiratory infectious agents, namely influenza types A, B, and C viruses; parainfluenza viruses 1 to 4, respiratory syncytial virus (RSV), adenovirus, human metapneumovirus, and picornavirus (enterovirus/rhinovirus) (https://www.chp.gov.hk/en/statistics/data/10/641/642/2274.html). ICV is tested for using rt RT-PCR targeting the NP gene of the virus. The assay was developed by the Microbiology Division, CHP-HK (a WHO-recognized National Influenza Centre and H5 Reference Laboratory), and details can be found on page 23 of the “WHO information for the molecular detection of influenza viruses” protocols (https://www.who.int/influenza/gisrs_laboratory/Protocols_influenza_virus_detection_Jan_2020.pdf). The sensitivity of the assay was optimized by adopting standard PCR conditions in accordance with reagent suppliers’ instructions. Threshold cycle (*C_T_*) values of 35 or below were considered positive, and specimens yielding higher *C_T_* values were retested to confirm results.

### Samples shared with WHO Collaborating Centre, London, for ICV characterization.

Of the archived ICV-positive clinical specimens still available, those with low *C_T_* values (in the range of 11.9 to 33.2; *n* = 98), together with four virus isolates, were shared as part of ongoing influenza surveillance conducted under the WHO Global Influenza Surveillance and Response System (GISRS). Three viruses (C/Hong Kong/6016/2008, C/Hong Kong/6483/2009, and C/Hong Kong/1/2018) were isolated in Madin-Darby canine kidney (MDCK) cells and one (C/Hong Kong/9284/2011) in LLC-MK2 rhesus monkey kidney cells. Clinical specimens and viruses that yielded a full open reading frame sequence for at least one ICV gene are listed in Table S1 in the supplemental material. The samples shared to enable this study were clinical specimens that had completed their diagnostic purpose, rendering them surplus materials. Patient confidentiality was maintained throughout to ensure no infringement on patient privacy and protection of personal data.

### ICV gene sequencing.

Nucleic acid (RNA) was extracted from all ICV-positive samples on a QIAcube HT platform (catalog no. 9001793) using a QIAamp 96 Virus QIAcube HT kit (catalog no. 57731) following the manufacturer’s instructions. NGS was performed on all RNA extracts, and for selected samples sequences of HE genes were confirmed by Sanger sequencing. RT-PCRs were performed using OneStep *ahead* RT-PCR kits (catalog no. 220213; Qiagen) with primers designed based on alignments of all ICV gene sequences available in the EpiFlu Database of the Global Initiative on Sharing All Influenza Data (GISAID) as of 18 April 2018 (see Table S3 in the supplemental material). RT-PCRs were set up and run using the protocols indicated in Table S4. RT-PCR products were purified using GE Healthcare GF 96 PCR purification kits (catalog no. 28903445) following the manufacturer’s instructions. Sanger sequencing of RT-PCR products was performed using ABI prism BigDye Terminator cycle sequencing kits (catalog no. 4336911) and an ABI 3730XL DNA Analyzer. NGS was performed, following library preparation with QIAseq FX DNA library kits (catalog no. 180475; Qiagen), on the Illumina MiSeq platform.

### Sequence assembly and curation.

Sequences emerging from Sanger sequencing were assembled using the Staden package (http://staden.sourceforge.net/), and those for HE genes from NGS were assembled using DNAStar’s Lasergene genomic suite of programs, particularly SeqMan NGen for assembly, with the HE sequence of C/Cameroon/13560/2017 (São Paulo S1 lineage; sequenced at the Worldwide Influenza Centre [WIC] just prior to receipt of the samples from Hong Kong) used as a reference, and SeqMan Pro for analysis and generation of consensus sequences. Over the course of this study, an assembly pipeline was developed for NGS output and used for whole-genome analyses, using the following software packages. Sequencing adapters and low-quality bases at the ends of the sequenced paired-end reads were first trimmed using Trim Galore (https://github.com/FelixKrueger/TrimGalore/blob/master/Docs/Trim_Galore_User_Guide.md). The reads were then aligned against a database of reference sequences corresponding to the different types and subtypes/lineages of influenza using the Burrow-Wheeler Aligner (BWA), resulting in a BAM format alignment (60). PCR duplicates were removed from the alignments using Picard (https://broadinstitute.github.io/picard/). Tools in the GATK suite were used to perform indel realignment and base quality score recalibration (61, 62) to generate a finalized BAM alignment. The base depths at each position of every alignment were extracted using the bedtools *genome coverage* tool (63), and Perl scripts were subsequently used to identify the optimal alignment. Alignments with the minimal number of zero-coverage positions were deemed optimal, with ambiguities being resolved through the consideration of reference coverage at higher depths (10×, 20×, 50×, 100×, and 1,000×). The consensus sequences were then generated using the SAMtools *mpileup* tool (64) and Perl scripts. The consensus only reports bases at reference positions with 10 or more reads. A single base was reported at a given position if the reported base was detected at a frequency of 80% or more, and ambiguity codes were reported at reference positions where multiple bases were detected at frequencies between 20% and 80%. Variants were identified by using a combination of the tools VarScan (65) and BCFtools (66). Finally, aligned reads from the chosen GATK-recalibrated BAM alignment file were extracted and stored in a FASTQ file with the help of the samtools *fastq* command (64). The generated FASTQ files contain only sequences from the identified influenza subtype/lineage, as contaminants from humans or other organisms would have been removed through the alignment process. Integrative Genomics Viewer (IGV) was used to visualize BAM aligned reads and variants (67). Nucleotide and deduced amino acid sequences were aligned and analyzed further using BioEdit (http://www.mbio.ncsu.edu/BioEdit/bioedit.html) before submission of sequences to GISAID.

Using consensus sequences for each set of HE gene sequences as references, identification of minority variants in sequences of HE genes followed a similar process to that used for consensus generation. Variants were called from GATK-recalibrated BAM alignments using VarScan (65) and BCFtools (66). VarScan parameters were set to discard bases with a Phred quality score of less than 30 and accept variants supported by at least 1% of reads. A high Phred quality threshold was used to reduce the risk of false positives. R scripts were used to scan the resultant variant call format (VCF) files for variants supported by 20% or fewer reads. To further reduce the risk of false positives, all variants with fewer than 10 supporting reads were removed.

### Phylogenetic analyses.

Maximum likelihood phylogenetic trees were estimated using RaxML v8.2X (https://cme.h-its.org/exelixis/software.html), followed by annotation with amino acid substitutions defining nodes and individual virus gene products using treesub (https://github.com/tamuri/treesub/blob/master/README.md). One hundred replicates were run to produce the bootstrap values indicated on the trees, which were visualized using FigTree (http://tree.bio.ed.ac.uk/software/figtree/) and highlighted using Adobe Illustrator CC 2015.3. Phylogenies relate to coding sequences of all gene products except for the HE gene, where the signal peptide coding sequences were removed to give mature HEF amino acid numbering. All ICV gene sequences not generated in the course of this study were recovered from the EpiFlu database of GISAID on 30 June 2019.

### Virus isolation.

Based on the completeness of whole-genome sequencing, the location of HE gene sequences in phylogenetic analyses, and the volume of each specimen, a selection (*n* = 8) were subjected to virus isolation in eggs. Clinical specimen aliquots (0.1 ml), in triplicate, were inoculated into the amniotic cavity of 14-day-old embryonated brown hens’ eggs (Hy-Line), which were incubated for 72 h at 34°C. Using pools of amniotic fluid from three eggs, two further blind passages were performed. Virus isolation was assessed by hemagglutination assay, which was performed at 4°C using 0.75% turkey red blood cells.

### Data availability.

Accession numbers generated by the EpiFlu database of GISAID for all sequence data generated in the course of this study (Table S1) and downloaded from GISAID for the generation of phylogenies (Table S5) are given in the supplemental material.

## Supplementary Material

Supplemental file 1

Supplemental file 2
