# Supplementary material for: Molecular Characterization of Influenza C Viruses from Outbreaks in Hong Kong SAR, China
Source: J Virol. 2020 Oct 14;94(21):e01051-20. doi: 10.1128/JVI.01051-20 (PMC7565627; doi:10.1128/JVI.01051-20)
Supplement: Supplemental file 1 [file JVI.01051-20-s0001.pdf]

Figure S1. Phylogenetic analysis of the coding regions of all seven ICV gene fragments.

The phylogenies were generated as described in Materials and Methods and are zoomable. Viruses from the 2015-16 and 2017-18 outbreaks in Hong Kong are shown in blue and pink respectively and other viruses sequenced at WHO CC, London are shown in green. Group defining amino acid substitutions are shown on nodes and virus specific substitutions are shown after virus names. For all gene products alignments of full-length coding sequences, based on the following numbers of nucleotides, were used: PB2 2322, PB1 2262, P3 2127, HE 1923, NP 1695, CM1 726, CM2 417, NS1 738 and NS2 546. Where known, the HE gene lineage is indicated after the virus names in panels A-C and E-I, based on the lineage assignments in panel D: ▽ C/Taylor/1233/47, ○ C/Mississippi/80, □ C/Yamagata/26/81, △ C/Aichi/1/81, ● C/Kanagawa/1/76 and ▲▼ C/Sao Paulo/378/82 (▼ C/Sao Paulo/378/82-S1, ▲ C/Sao Paulo/378/82-S2); symbols in green (▽, ○, □, △, ●, ▼, ▲) indicate gene products that fall outside of the main groupings of viruses with HE genes falling in the respective HE lineages. The bar indicates the proportion of nucleotide changes between sequences. Bootstrap values derived by running 100 replicates are shown in dark blue; only those of 70 and above are shown as indicators of branch robustness. We gratefully acknowledge the authors, originating and submitting laboratories of the sequences from the EpiFlu™ database of GISAID which were downloaded for use in the preparation of this manuscript (all submitters of data may be contacted directly via the GISAID website and the relevant sequence accession numbers are given in Table S5).

Figure S2. HEF glycoprotein alignment with HE gene and HEF phylogenies.

A: Human ICV HEF proteins from viruses representing the phylogenetic groups in Figure 2 are aligned together with those from pigs in China. Potential N-linked glycosylation sequons are

highlighted in yellow and the HEF1/HEF2 processing site is indicated in cyan. All sequences were downloaded from GISAID. The names of lineage and sublineage representatives are underlined. Residues associated with neutralization sensitive epitopes are indicated at the bottom of each alignment row: A-1, A-2, A-3, A-4, Y-1 with the C/Taylor/1233/47 row repeated for amino acids 101-200 to show positions associated with overlapping epitopes (1). **B:** HE gene phylogeny for sequences of representative human ICVs and non-human ICVs available in the GenBank database. The phylogeny was generated and annotated as described in Materials and Methods with non-human viruses indicated in red. Non-human ICVs are shown in red: the three pig viruses map with those of the C/Yamagata/26/81 lineage while the bovine virus maps with those of the C/Mississippi/80 lineage. **C:** HEF glycoprotein phylogeny for the same ICVs in panel B, with sequences from influenza D viruses available in the GenBank database. A protein alignment was used to generate the Maximum Likelihood tree due to alignment issues at the gene level (introduction of non-triplet gaps that led to generation of spurious stop codons within reading frames). Non-human ICVs are shown in red and influenza D viruses that had formerly been designated as ICVs are shown in blue. Bootstrap values derived by running 100 replicates are shown in dark blue; those of 70 and above are considered as indicators of branch robustness.

1. Matsuzaki Y, Sugawara K, Furuse Y, Shimotai Y, Hongo S, Mizuta K, Nishimura H. 2018. Neutralizing Epitopes and Residues Mediating the Potential Antigenic Drift of the Hemagglutinin-Esterase Protein of Influenza C Virus. Viruses 10:e417-e434.

Figure S1. Phylogenetic analysis of the coding regions of all seven ICV gene fragments.

A: PB2

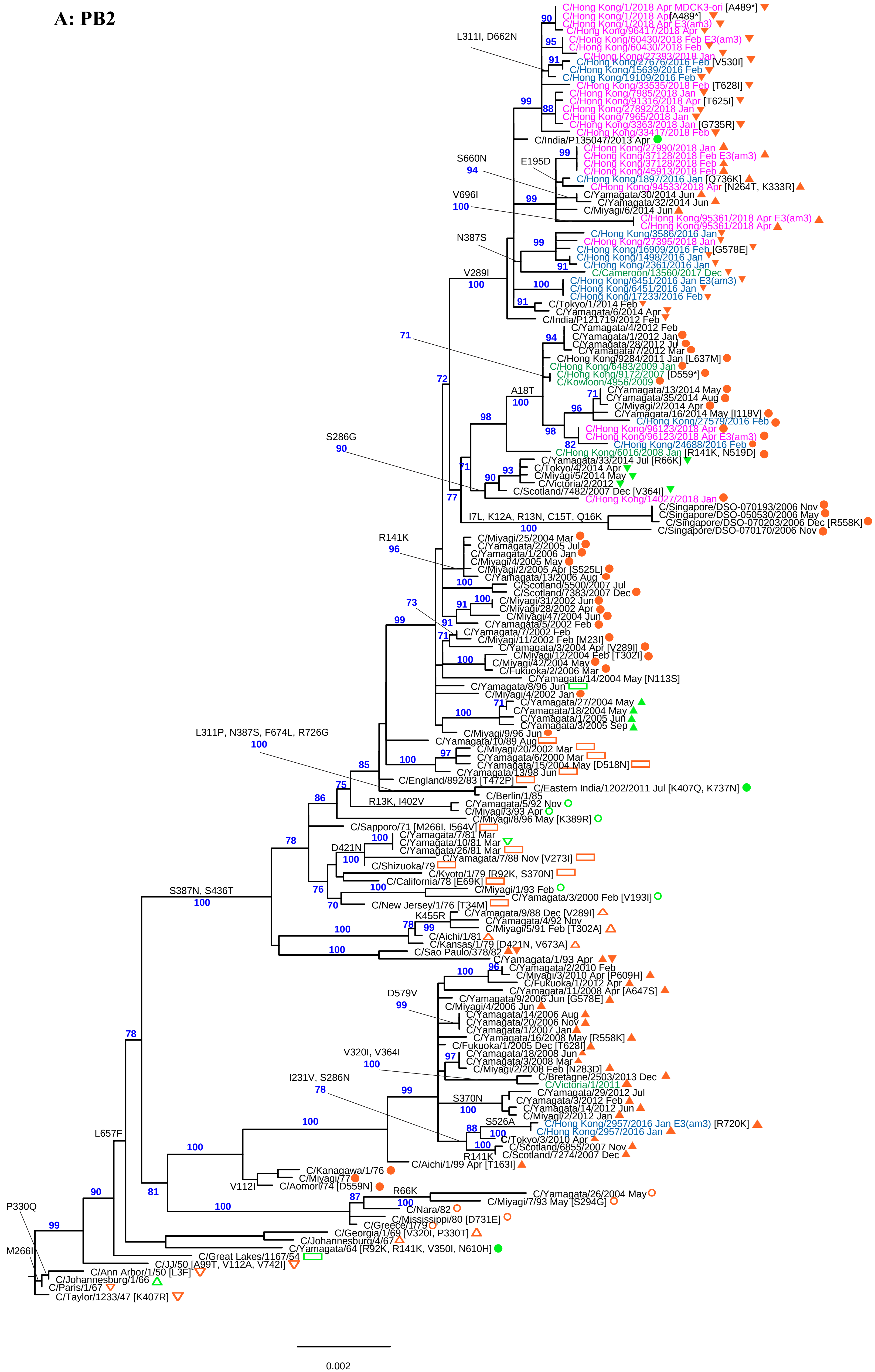

B: PB1

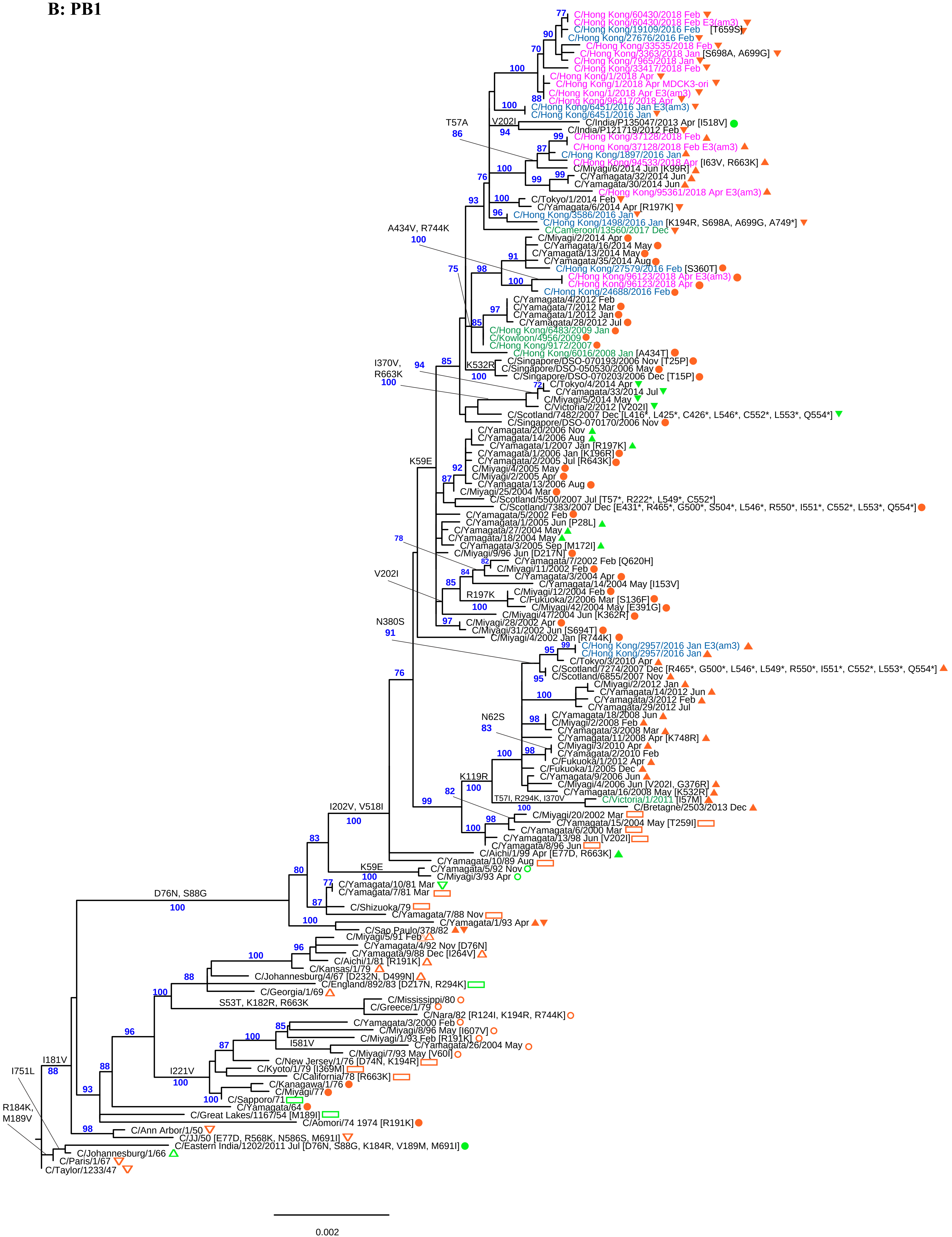

77
C/Hong Kong/60430/2018 Feb
C/Hong Kong/60430/2018 Feb E3(am3)
90
C/Hong Kong/19109/2016 Feb [T659S]
C/Hong Kong/27676/2016 Feb
70
C/Hong Kong/33535/2018 Feb
C/Hong Kong/3363/2018 Jan [S698A, A699G]
C/Hong Kong/7965/2018 Jan
100
C/Hong Kong/33417/2018 Feb
C/Hong Kong/1/2018 Apr
C/Hong Kong/1/2018 Apr MDCK3-ori
C/Hong Kong/1/2018 Apr E3(am3)
88
C/Hong Kong/96417/2018 Apr
100
C/Hong Kong/6451/2016 Jan E3(am3)
C/Hong Kong/6451/2016 Jan
T57A
86
V202I
94
C/India/P135047/2013 Apr [I518V]
C/India/P121719/2012 Feb
99
C/Hong Kong/37128/2018 Feb
87
C/Hong Kong/37128/2018 Feb E3(am3)
C/Hong Kong/1897/2016 Jan
C/Hong Kong/94533/2018 Apr [I63V, R663K]
100
C/Miyagi/6/2014 Jun [K99R]
99
C/Yamagata/32/2014 Jun
99
C/Yamagata/30/2014 Jun
76
C/Hong Kong/95361/2018 Apr E3(am3)
100
C/Tokyo/1/2014 Feb
93
C/Yamagata/6/2014 Apr [R197K]
96
C/Hong Kong/3586/2016 Jan
C/Hong Kong/1498/2016 Jan [K194R, S698A, A699G, A749*]
A434V, R744K
100
C/Cameroon/13560/2017 Dec
C/Miyagi/2/2014 Apr
C/Yamagata/16/2014 May
C/Yamagata/13/2014 May
91
C/Yamagata/35/2014 Aug
75
98
C/Hong Kong/27579/2016 Feb [S360T]
100
C/Hong Kong/96123/2018 Apr E3(am3)
C/Hong Kong/96123/2018 Apr
C/Hong Kong/24688/2016 Feb
C/Yamagata/4/2012 Feb
97
C/Yamagata/7/2012 Mar
C/Yamagata/1/2012 Jan
85
C/Yamagata/28/2012 Jul
C/Hong Kong/6483/2009 Jan
C/Kowloon/4956/2009
C/Hong Kong/9172/2007
C/Hong Kong/6016/2008 Jan [A434T]
I370V,
R663K
100
94
85
K532R
C/Singapore/DSO-070193/2006 Nov [T25P]
C/Singapore/DSO-050530/2006 May
100
C/Singapore/DSO-070203/2006 Dec [T15P]
72
C/Tokyo/4/2014 Apr
C/Yamagata/33/2014 Jul
C/Miyagi/5/2014 May
C/Victoria/2/2012 [V202I]
C/Scotland/7482/2007 Dec [L416*, L425*, C426*, L546*, C552*, L553*, Q554*]
C/Singapore/DSO-070170/2006 Nov
C/Yamagata/20/2006 Nov
C/Yamagata/14/2006 Aug
C/Yamagata/1/2007 Jan [R197K]
C/Yamagata/1/2006 Jan [K196R]
K59E
C/Yamagata/2/2005 Jul [R643K]
92
C/Miyagi/4/2005 May
87
C/Miyagi/2/2005 Apr
C/Yamagata/13/2006 Aug
C/Miyagi/25/2004 Mar
C/Scotland/5500/2007 Jul [T57*, R222*, L549*, C552*]
C/Scotland/7383/2007 Dec [E431*, R465*, G500*, S504*, L546*, R550*, I551*, C552*, L553*, Q554*]
C/Yamagata/5/2002 Feb
C/Yamagata/1/2005 Jun [P28L]
C/Yamagata/27/2004 May
C/Yamagata/18/2004 May
78
C/Yamagata/3/2005 Sep [M172I]
C/Miyagi/9/96 Jun [D217N]
82
C/Yamagata/7/2002 Feb [Q620H]
C/Miyagi/11/2002 Feb
84
C/Yamagata/3/2004 Apr
V202I
85
C/Yamagata/14/2004 May [I153V]
R197K
C/Miyagi/12/2004 Feb
C/Fukuoka/2/2006 Mar [S136F]
100
C/Miyagi/42/2004 May [E391G]
C/Miyagi/47/2004 Jun [K362R]
N380S
97
C/Miyagi/28/2002 Apr
91
C/Miyagi/31/2002 Jun [S694T]
C/Miyagi/4/2002 Jan [R744K]
99
C/Hong Kong/2957/2016 Jan E3(am3)
95
C/Hong Kong/2957/2016 Jan
C/Tokyo/3/2010 Apr
C/Scotland/7274/2007 Dec [R465*, G500*, L546*, L549*, R550*, I551*, C552*, L553*, Q554*]
76
95
C/Scotland/6855/2007 Nov
C/Miyagi/2/2012 Jan
100
C/Yamagata/14/2012 Jun
C/Yamagata/3/2012 Feb
C/Yamagata/29/2012 Jul
N62S
C/Yamagata/18/2008 Jun
98
C/Miyagi/2/2008 Feb
83
C/Yamagata/3/2008 Mar
C/Yamagata/11/2008 Apr [K748R]
98
C/Miyagi/3/2010 Apr
100
C/Yamagata/2/2010 Feb
C/Fukuoka/1/2012 Apr
C/Fukuoka/1/2005 Dec
K119R
C/Yamagata/9/2006 Jun
100
C/Miyagi/4/2006 Jun [V202I, G376R]
82
C/Yamagata/16/2008 May [K532R]
T57I, R294K, I370V
C/Victoria/1/2011 [I57M]
99
C/Bretagne/2503/2013 Dec
I202V, V518I
100
C/Miyagi/20/2002 Mar
98
C/Yamagata/15/2004 May [T259I]
100
C/Yamagata/6/2000 Mar
100
C/Yamagata/13/98 Jun [V202I]
83
C/Yamagata/8/96 Jun
C/Aichi/1/99 Apr [E77D, R663K]
C/Yamagata/10/89 Aug
80
K59E
C/Yamagata/5/92 Nov
100
C/Miyagi/3/93 Apr
77
C/Yamagata/10/81 Mar
D76N, S88G
C/Yamagata/7/81 Mar
87
100
C/Shizuoka/79
C/Yamagata/7/88 Nov
100
C/Yamagata/1/93 Apr
C/Sao Paulo/378/82
C/Miyagi/5/91 Feb
96
C/Yamagata/4/92 Nov [D76N]
100
C/Yamagata/9/88 Dec [I264V]
C/Aichi/1/81 [R191K]
C/Kansas/1/79
88
C/Johannesburg/4/67 [D232N, D499N]
C/England/892/83 [D217N, R294K]
100
C/Georgia/1/69
C/Mississippi/80
S53T, K182R, R663K
C/Greece/1/79
C/Nara/82 [R124I, K194R, R744K]
85
C/Yamagata/3/2000 Feb
96
100
C/Miyagi/8/96 May [I607V]
C/Miyagi/1/93 Feb [R191K]
87
I581V
C/Yamagata/26/2004 May
C/Miyagi/7/93 May [V60I]
C/New Jersey/1/76 [D74N, K194R]
I181V
88
C/Kyoto/1/79 [I369M]
I751L
88
I221V
C/California/78 [R663K]
100
C/Kanagawa/1/76
93
C/Miyagi/77
100
C/Sapporo/71
R184K,
M189V
C/Yamagata/64
C/Great Lakes/1167/54 [M189I]
C/Aomori/74 1974 [R191K]
98
C/Ann Arbor/1/50
C/JJ/50 [E77D, R568K, N586S, M691I]
C/Eastern India/1202/2011 Jul [D76N, S88G, K184R, V189M, M691I]
C/Johannesburg/1/66
C/Paris/1/67
C/Taylor/1233/47
0.002

C: P3

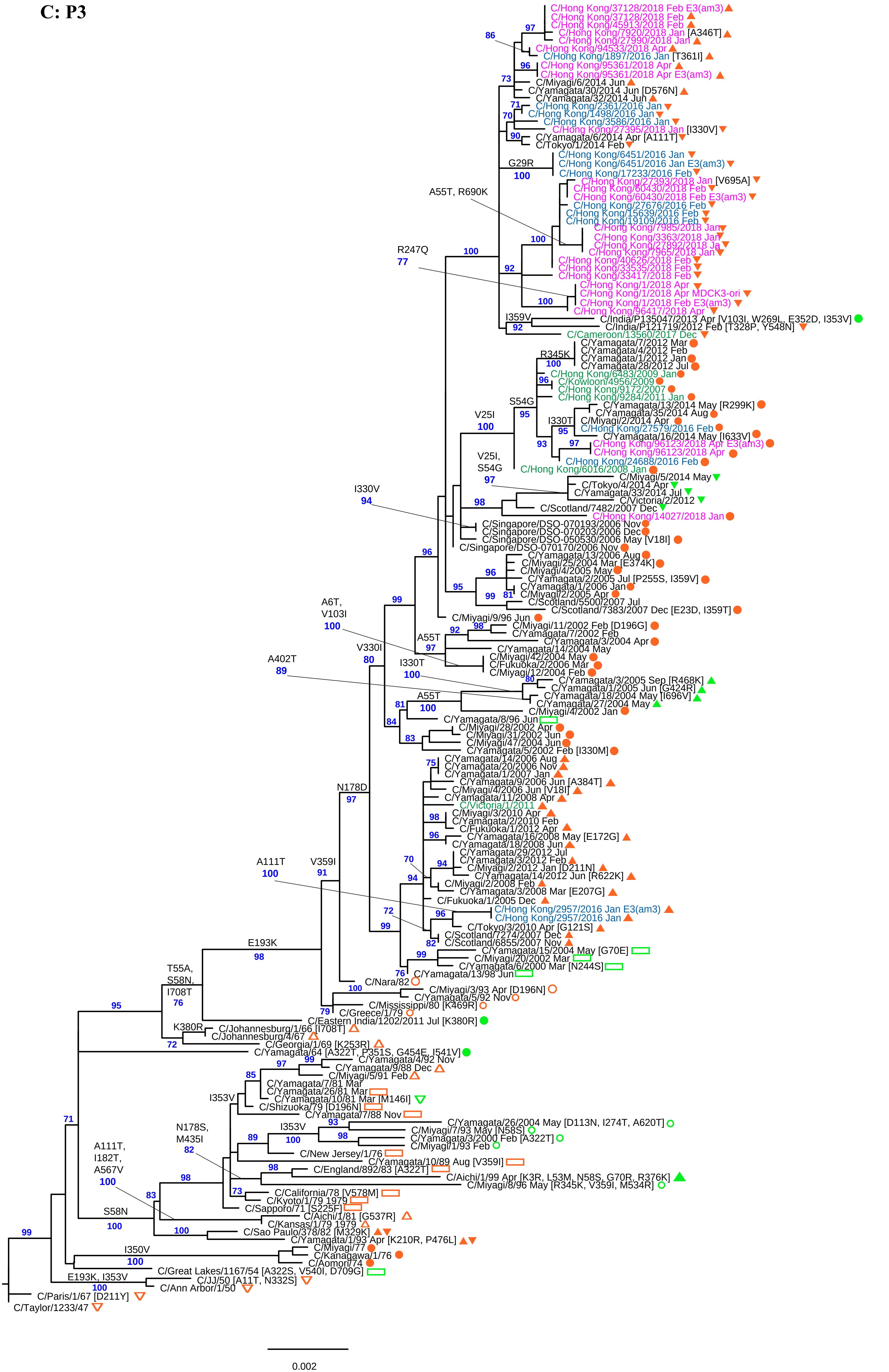
C/Hong Kong/37128/2018 Feb E3(am3)
C/Hong Kong/37128/2018 Feb
C/Hong Kong/45913/2018 Feb
C/Hong Kong/7920/2018 Jan [A346T]
C/Hong Kong/27990/2018 Jan
C/Hong Kong/94533/2018 Apr
C/Hong Kong/1897/2016 Jan [T361I]
C/Hong Kong/95361/2018 Apr
C/Hong Kong/95361/2018 Apr E3(am3)
C/Miyagi/6/2014 Jun
C/Yamagata/30/2014 Jun [D576N]
C/Yamagata/32/2014 Jun
C/Hong Kong/2361/2016 Jan
C/Hong Kong/1498/2016 Jan
C/Hong Kong/3586/2016 Jan
C/Hong Kong/27395/2018 Jan [I330V]
C/Yamagata/6/2014 Apr [A111T]
C/Tokyo/1/2014 Feb
C/Hong Kong/6451/2016 Jan
C/Hong Kong/6451/2016 Jan E3(am3)
C/Hong Kong/17233/2016 Feb
C/Hong Kong/27393/2018 Jan [V695A]
C/Hong Kong/60430/2018 Feb
C/Hong Kong/60430/2018 Feb E3(am3)
C/Hong Kong/27676/2016 Feb
C/Hong Kong/15639/2016 Feb
C/Hong Kong/19109/2016 Feb
C/Hong Kong/7985/2018 Jan
C/Hong Kong/3363/2018 Jan
C/Hong Kong/27892/2018 Ja
C/Hong Kong/7965/2018 Jan
C/Hong Kong/40626/2018 Feb
C/Hong Kong/33535/2018 Feb
C/Hong Kong/33417/2018 Feb
C/Hong Kong/1/2018 Apr
C/Hong Kong/1/2018 Apr MDCK3-ori
C/Hong Kong/1/2018 Feb E3(am3)
C/Hong Kong/96417/2018 Apr
C/India/P135047/2013 Apr [V103I, W269L, E352D, I353V]
C/India/P121719/2012 Feb [T328P, Y548N]
C/Cameroon/13560/2017 Dec
C/Yamagata/7/2012 Mar
C/Yamagata/4/2012 Feb
C/Yamagata/1/2012 Jan
C/Yamagata/28/2012 Jul
C/Hong Kong/6483/2009 Jan
C/Kowloon/4956/2009
C/Hong Kong/9172/2007
C/Hong Kong/9284/2011 Jan
C/Yamagata/13/2014 May [R299K]
C/Yamagata/35/2014 Aug
C/Miyagi/2/2014 Apr
C/Hong Kong/27579/2016 Feb
C/Yamagata/16/2014 May [I633V]
C/Hong Kong/96123/2018 Apr E3(am3)
C/Hong Kong/96123/2018 Apr
C/Hong Kong/24688/2016 Feb
C/Hong Kong/6016/2008 Jan
C/Miyagi/5/2014 May
C/Tokyo/4/2014 Apr
C/Yamagata/33/2014 Jul
C/Victoria/2/2012
C/Scotland/7482/2007 Dec
C/Hong Kong/14027/2018 Jan
C/Singapore/DSO-070193/2006 Nov
C/Singapore/DSO-070203/2006 Dec
C/Singapore/DSO-050530/2006 May [V18I]
C/Singapore/DSO-070170/2006 Nov
C/Yamagata/13/2006 Aug
C/Miyagi/25/2004 Mar [E374K]
C/Miyagi/4/2005 May
C/Yamagata/2/2005 Jul [P255S, I359V]
C/Yamagata/1/2006 Jan
C/Miyagi/2/2005 Apr
C/Scotland/5500/2007 Jul
C/Scotland/7383/2007 Dec [E23D, I359T]
C/Miyagi/9/96 Jun
C/Miyagi/11/2002 Feb [D196G]
C/Yamagata/7/2002 Feb
C/Yamagata/3/2004 Apr
C/Yamagata/14/2004 May
C/Miyagi/42/2004 May
C/Fukuoka/2/2006 Mar
C/Miyagi/12/2004 Feb
C/Yamagata/3/2005 Sep [R468K]
C/Yamagata/1/2005 Jun [G424R]
C/Yamagata/18/2004 May [I696V]
C/Yamagata/27/2004 May
C/Miyagi/4/2002 Jan
C/Yamagata/8/96 Jun
C/Miyagi/28/2002 Apr
C/Miyagi/31/2002 Jun
C/Miyagi/47/2004 Jun
C/Yamagata/5/2002 Feb [I330M]
C/Yamagata/14/2006 Aug
C/Yamagata/20/2006 Nov
C/Yamagata/1/2007 Jan
C/Yamagata/9/2006 Jun [A384T]
C/Miyagi/4/2006 Jun [V18I]
C/Yamagata/11/2008 Apr
C/Victoria/1/2011
C/Miyagi/3/2010 Apr
C/Yamagata/2/2010 Feb
C/Fukuoka/1/2012 Apr
C/Yamagata/16/2008 May [E172G]
C/Yamagata/18/2008 Jun
C/Yamagata/29/2012 Jul
C/Yamagata/3/2012 Feb
C/Miyagi/2/2012 Jan [D211N]
C/Yamagata/14/2012 Jun [R622K]
C/Miyagi/2/2008 Feb
C/Yamagata/3/2008 Mar [E207G]
C/Fukuoka/1/2005 Dec
C/Hong Kong/2957/2016 Jan E3(am3)
C/Hong Kong/2957/2016 Jan
C/Tokyo/3/2010 Apr [G121S]
C/Scotland/7274/2007 Dec
C/Scotland/6855/2007 Nov
C/Yamagata/15/2004 May [G70E]
C/Miyagi/20/2002 Mar
C/Yamagata/6/2000 Mar [N244S]
C/Yamagata/13/98 Jun
C/Nara/82
C/Miyagi/3/93 Apr [D196N]
C/Yamagata/5/92 Nov
C/Mississippi/80 [K469R]
C/Greece/1/79
C/Eastern India/1202/2011 Jul [K380R]
C/Johannesburg/1/66 [I708T]
C/Johannesburg/4/67
C/Georgia/1/69 [K253R]
C/Yamagata/64 [A322T, P351S, G454E, I541V]
C/Yamagata/4/92 Nov
C/Yamagata/9/88 Dec
C/Miyagi/5/91 Feb
C/Yamagata/7/81 Mar
C/Yamagata/26/81 Mar
C/Yamagata/10/81 Mar [M146I]
C/Shizuoka/79 [D196N]
C/Yamagata/7/88 Nov
C/Yamagata/26/2004 May [D113N, I274T, A620T]
C/Miyagi/7/93 May [N58S]
C/Yamagata/3/2000 Feb [A322T]
C/Miyagi/1/93 Feb
C/New Jersey/1/76
C/Yamagata/10/89 Aug [V359I]
C/England/892/83 [A322T]
C/Aichi/1/99 Apr [K3R, L53M, N58S, G70R, R376K]
C/Miyagi/8/96 May [R345K, V359I, M534R]
C/California/78 [V578M]
C/Kyoto/1/79 1979
C/Sapporo/71 [S225F]
C/Aichi/1/81 [G537R]
C/Kansas/1/79 1979
C/Sao Paulo/378/82 [M329K]
C/Yamagata/1/93 Apr [K210R, P476L]
C/Miyagi/77
C/Kanagawa/1/76
C/Aomori/74
C/Great Lakes/1167/54 [A322S, V540I, D709G]
C/JJ/50 [A11T, N332S]
C/Ann Arbor/1/50
C/Paris/1/67 [D211Y]
C/Taylor/1233/47
G29R
A55T, R690K
R247Q
I359V
R345K
S54G
V25I
I330T
V25I,
S54G
I330V
A6T,
V103I
A55T
I330T
A402T
V330I
A55T
N178D
A111T
V359I
E193K
T55A,
S58N,
I708T
K380R
I353V
I353V
N178S,
M435I
A111T,
I182T,
A567V
S58N
I350V
E193K, I353V
0.002

D: HE

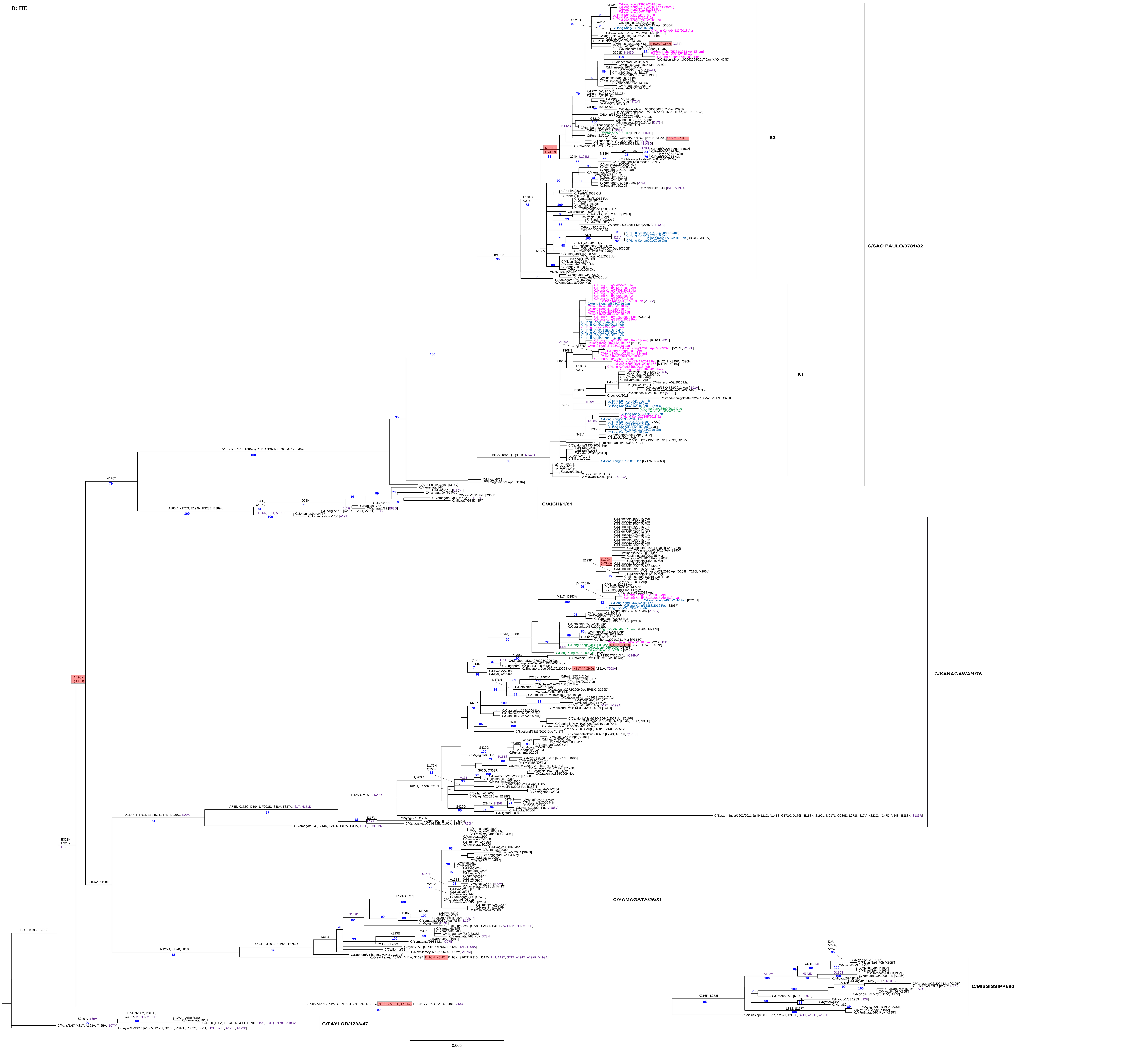
S2
S1
C/SAO PAULO/3781/82
C/AICHI/1/81
C/KANAGAWA/1/76
C/YAMAGATA/26/81
C/MISSISSIPPI/80
C/TAYLOR/1233/47
0.005

E: NP

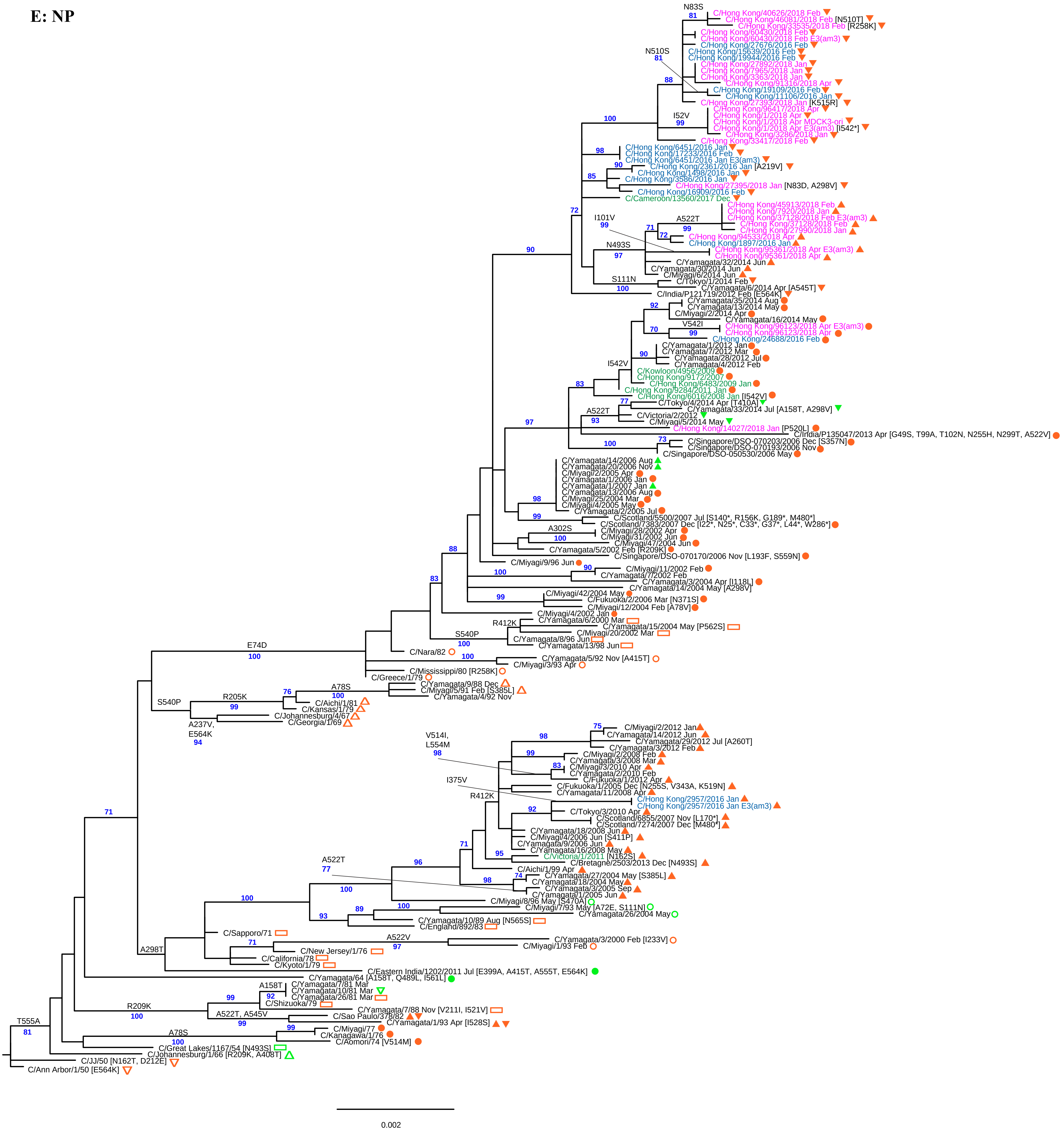
N83S
81
C/Hong Kong/40626/2018 Feb
C/Hong Kong/46081/2018 Feb [N510T]
C/Hong Kong/33535/2018 Feb [R258K]
C/Hong Kong/60430/2018 Feb
C/Hong Kong/60430/2018 Feb E3(am3)
C/Hong Kong/27676/2016 Feb
N510S
81
C/Hong Kong/15639/2016 Feb
C/Hong Kong/19944/2016 Feb
C/Hong Kong/27892/2018 Jan
C/Hong Kong/7965/2018 Jan
C/Hong Kong/3363/2018 Jan
88
C/Hong Kong/91316/2018 Apr
C/Hong Kong/19109/2016 Feb
C/Hong Kong/11106/2016 Jan
C/Hong Kong/27393/2018 Jan [K515R]
I52V
99
100
C/Hong Kong/96417/2018 Apr
C/Hong Kong/1/2018 Apr
C/Hong Kong/1/2018 Apr MDCK3-ori
C/Hong Kong/1/2018 Apr E3(am3) [I542*]
C/Hong Kong/3286/2018 Jan
C/Hong Kong/33417/2018 Feb
98
C/Hong Kong/6451/2016 Jan
C/Hong Kong/17233/2016 Feb
C/Hong Kong/6451/2016 Jan E3(am3)
90
C/Hong Kong/2361/2016 Jan [A219V]
85
C/Hong Kong/1498/2016 Jan
C/Hong Kong/3586/2016 Jan
C/Hong Kong/27395/2018 Jan [N83D, A298V]
C/Hong Kong/16909/2016 Feb
C/Cameroon/13560/2017 Dec
72
C/Hong Kong/45913/2018 Feb
C/Hong Kong/7920/2018 Jan
C/Hong Kong/37128/2018 Feb E3(am3)
I101V
99
A522T
99
71
C/Hong Kong/37128/2018 Feb
C/Hong Kong/27990/2018 Jan
72
C/Hong Kong/94533/2018 Apr
C/Hong Kong/1897/2016 Jan
N493S
97
C/Hong Kong/95361/2018 Apr E3(am3)
C/Hong Kong/95361/2018 Apr
90
C/Yamagata/32/2014 Jun
C/Yamagata/30/2014 Jun
C/Miyagi/6/2014 Jun
S111N
100
C/Tokyo/1/2014 Feb
C/Yamagata/6/2014 Apr [A545T]
C/India/P121719/2012 Feb [E564K]
92
C/Yamagata/35/2014 Aug
C/Yamagata/13/2014 May
C/Miyagi/2/2014 Apr
C/Yamagata/16/2014 May
V542I
70
99
C/Hong Kong/96123/2018 Apr E3(am3)
C/Hong Kong/96123/2018 Apr
C/Hong Kong/24688/2016 Feb
90
C/Yamagata/1/2012 Jan
C/Yamagata/7/2012 Mar
C/Yamagata/28/2012 Jul
I542V
C/Yamagata/4/2012 Feb
C/Kowloon/4956/2009
C/Hong Kong/9172/2007
C/Hong Kong/6483/2009 Jan
83
C/Hong Kong/9284/2011 Jan
C/Hong Kong/6016/2008 Jan [I542V]
77
C/Tokyo/4/2014 Apr [T410A]
A522T
C/Yamagata/33/2014 Jul [A158T, A298V]
C/Victoria/2/2012
93
C/Miyagi/5/2014 May
97
C/Hong Kong/14027/2018 Jan [P520L]
C/India/P135047/2013 Apr [G49S, T99A, T102N, N255H, N299T, A522V]
100
73
C/Singapore/DSO-070203/2006 Dec [S357N]
C/Singapore/DSO-070193/2006 Nov
C/Singapore/DSO-050530/2006 May
C/Yamagata/14/2006 Aug
C/Yamagata/20/2006 Nov
C/Miyagi/2/2005 Apr
C/Yamagata/1/2006 Jan
C/Yamagata/1/2007 Jan
C/Yamagata/13/2006 Aug
98
C/Miyagi/25/2004 Mar
C/Miyagi/4/2005 May
99
C/Yamagata/2/2005 Jul
C/Scotland/5500/2007 Jul [S140*, R156K, G189*, M480*]
C/Scotland/7383/2007 Dec [I22*, N25*, C33*, G37*, L44*, W286*]
A302S
C/Miyagi/28/2002 Apr
100
C/Miyagi/31/2002 Jun
C/Miyagi/47/2004 Jun
C/Yamagata/5/2002 Feb [R209K]
C/Singapore/DSO-070170/2006 Nov [L193F, S559N]
88
C/Miyagi/9/96 Jun
100
90
C/Miyagi/11/2002 Feb
C/Yamagata/7/2002 Feb
C/Yamagata/3/2004 Apr [I118L]
83
C/Yamagata/14/2004 May [A298V]
99
C/Miyagi/42/2004 May
C/Fukuoka/2/2006 Mar [N371S]
C/Miyagi/12/2004 Feb [A78V]
C/Miyagi/4/2002 Jan
R412K
C/Yamagata/6/2000 Mar
C/Yamagata/15/2004 May [P562S]
S540P
C/Miyagi/20/2002 Mar
100
C/Yamagata/8/96 Jun
E74D
C/Yamagata/13/98 Jun
100
C/Nara/82
100
C/Yamagata/5/92 Nov [A415T]
C/Miyagi/3/93 Apr
C/Mississippi/80 [R258K]
C/Greece/1/79
A78S
C/Yamagata/9/88 Dec
76
100
C/Miyagi/5/91 Feb [S385L]
S540P
R205K
C/Yamagata/4/92 Nov
99
C/Aichi/1/81
C/Kansas/1/79
C/Johannesburg/4/67
A237V,
C/Georgia/1/69
E564K
94
75
C/Miyagi/2/2012 Jan
V514I,
98
C/Yamagata/14/2012 Jun
L554M
C/Yamagata/29/2012 Jul [A260T]
98
C/Yamagata/3/2012 Feb
99
C/Miyagi/2/2008 Feb
C/Yamagata/3/2008 Mar
83
C/Miyagi/3/2010 Apr
C/Yamagata/2/2010 Feb
I375V
C/Fukuoka/1/2012 Apr
C/Fukuoka/1/2005 Dec [N255S, V343A, K519N]
R412K
C/Yamagata/11/2008 Apr
C/Hong Kong/2957/2016 Jan
C/Hong Kong/2957/2016 Jan E3(am3)
92
C/Tokyo/3/2010 Apr
C/Scotland/6855/2007 Nov [L170*]
C/Scotland/7274/2007 Dec [M480*]
C/Yamagata/18/2008 Jun
C/Miyagi/4/2006 Jun [S411P]
71
C/Yamagata/9/2006 Jun
C/Yamagata/16/2008 May
95
C/Victoria/1/2011 [N162S]
A522T
96
C/Bretagne/2503/2013 Dec [N493S]
77
C/Aichi/1/99 Apr
74
C/Yamagata/27/2004 May [S385L]
98
C/Yamagata/18/2004 May
C/Yamagata/3/2005 Sep
100
C/Yamagata/1/2005 Jun
100
C/Miyagi/8/96 May [S470A]
100
C/Miyagi/7/93 May [A72E, S111N]
89
93
C/Yamagata/26/2004 May
C/Yamagata/10/89 Aug [N565S]
C/England/892/83
71
C/Sapporo/71
A522V
C/Yamagata/3/2000 Feb [I233V]
97
C/Miyagi/1/93 Feb
C/New Jersey/1/76
C/California/78
A298T
C/Kyoto/1/79
C/Eastern India/1202/2011 Jul [E399A, A415T, A555T, E564K]
C/Yamagata/64 [A158T, Q489L, I561L]
A158T
C/Yamagata/7/81 Mar
99
92
C/Yamagata/10/81 Mar
C/Yamagata/26/81 Mar
C/Shizuoka/79
R209K
C/Yamagata/7/88 Nov [V211I, I521V]
100
A522T, A545V
C/Sao Paulo/378/82
99
C/Yamagata/1/93 Apr [I528S]
99
C/Miyagi/77
A78S
C/Kanagawa/1/76
100
C/Aomori/74 [V514M]
T555A
C/Great Lakes/1167/54 [N493S]
81
C/Johannesburg/1/66 [R209K, A408T]
C/JJ/50 [N162T, D212E]
C/Ann Arbor/1/50 [E564K]
0.002

F: CM1

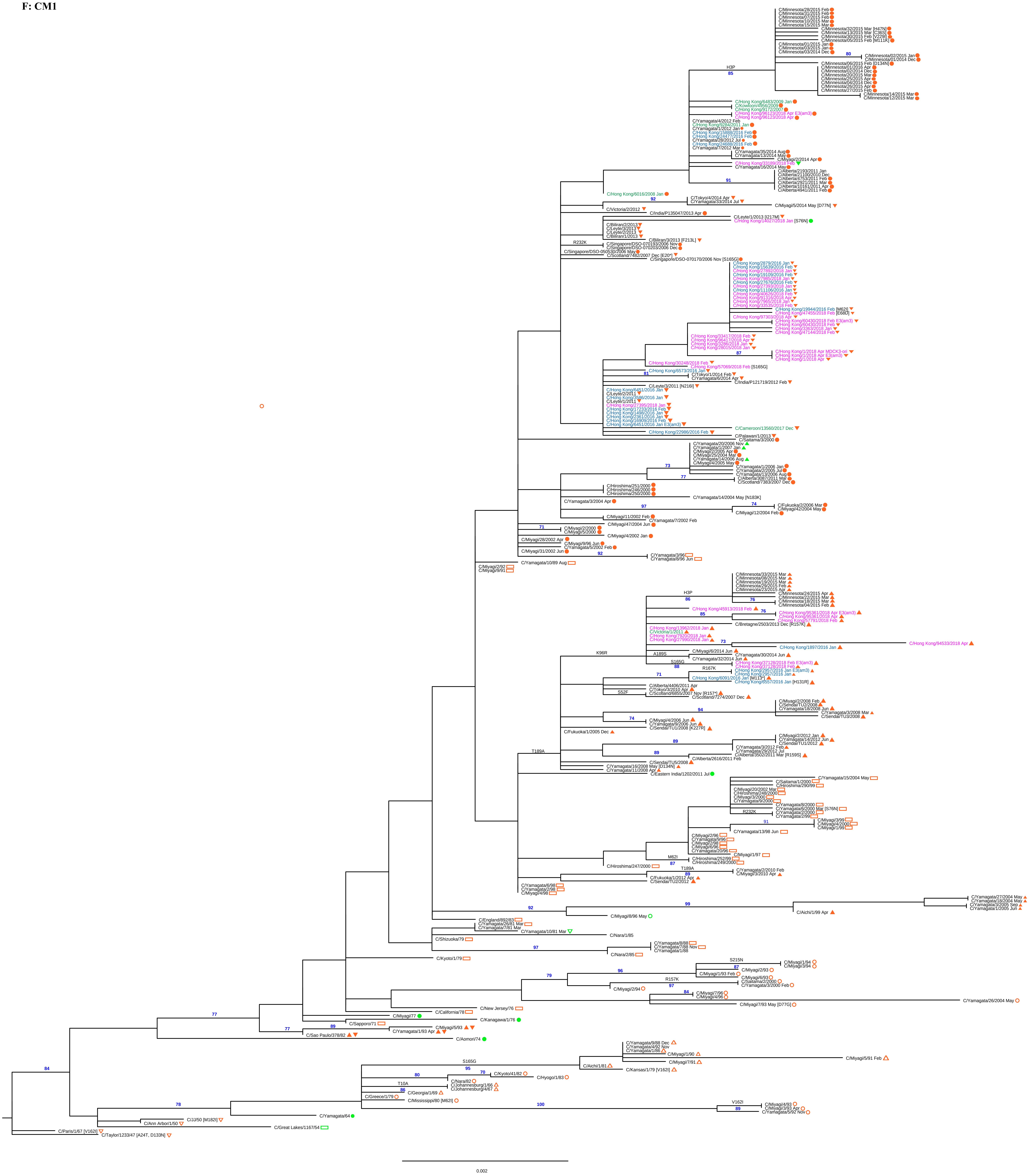

C/Minnesota/28/2015 Feb
C/Minnesota/31/2015 Feb
C/Minnesota/07/2015 Feb
C/Minnesota/10/2015 Mar
C/Minnesota/15/2015 Mar
C/Minnesota/32/2015 Mar [H47N]
C/Minnesota/13/2015 Mar [C36S]
C/Minnesota/30/2015 Feb [V229I]
C/Minnesota/05/2015 Feb [M111K]
C/Minnesota/01/2015 Jan
C/Minnesota/03/2015 Jan
C/Minnesota/03/2014 Dec
80
C/Minnesota/02/2015 Jan
C/Minnesota/01/2014 Dec
C/Minnesota/06/2015 Feb [D134N]
C/Minnesota/01/2016 Apr
C/Minnesota/02/2014 Dec
C/Minnesota/20/2015 Mar
C/Minnesota/04/2016 Apr
C/Minnesota/25/2015 Apr
C/Minnesota/26/2015 Apr
C/Minnesota/27/2015 Feb
C/Minnesota/14/2015 Mar
C/Minnesota/12/2015 Mar
H3P
85
C/Hong Kong/6483/2009 Jan
C/Kowloon/4956/2009
C/Hong Kong/9172/2007
C/Hong Kong/96123/2018 Apr E3(am3)
C/Hong Kong/96123/2018 Apr
C/Yamagata/4/2012 Feb
C/Hong Kong/9284/2011 Jan
C/Yamagata/1/2012 Jan
C/Hong Kong/15888/2016 Feb
C/Hong Kong/24477/2016 Feb
C/Yamagata/28/2012 Jul
C/Hong Kong/24688/2016 Feb
C/Yamagata/7/2012 Mar
C/Yamagata/35/2014 Aug
C/Yamagata/13/2014 May
C/Miyagi/2/2014 Apr
C/Hong Kong/33189/2018 Feb
C/Yamagata/16/2014 May
C/Alberta/2193/2011 Jan
C/Alberta/21100/2010 Dec
C/Alberta/4753/2011 Feb
C/Alberta/2921/2011 Mar
C/Alberta/10161/2011 Apr
C/Alberta/4941/2011 Feb
91
C/Hong Kong/6016/2008 Jan
92
C/Tokyo/4/2014 Apr
C/Yamagata/33/2014 Jul
C/Miyagi/5/2014 May [D77N]
C/Victoria/2/2012
C/India/P135047/2013 Apr
C/Leyte/1/2013 [I217M]
C/Hong Kong/14027/2018 Jan [S76N]
C/Biliran/2/2013
C/Leyte/3/2013
C/Leyte/2/2013
C/Biliran/1/2013
C/Biliran/3/2013 [F213L]
R232K
C/Singapore/DSO-070193/2006 Nov
C/Singapore/DSO-070203/2006 Dec
C/Singapore/DSO-050530/2006 May
C/Scotland/7482/2007 Dec [E20*]
C/Singapore/DSO-070170/2006 Nov [S165G]
C/Hong Kong/2879/2016 Jan
C/Hong Kong/15639/2016 Feb
C/Hong Kong/27892/2018 Jan
C/Hong Kong/19109/2016 Feb
C/Hong Kong/7985/2018 Jan
C/Hong Kong/27676/2016 Feb
C/Hong Kong/27393/2018 Jan
C/Hong Kong/11106/2016 Jan
C/Hong Kong/40676/2018 Feb
C/Hong Kong/91316/2018 Apr
C/Hong Kong/7965/2018 Jan
C/Hong Kong/33535/2018 Feb
C/Hong Kong/19944/2016 Feb [M62I]
C/Hong Kong/47455/2018 Feb [E68D]
C/Hong Kong/97303/2018 Apr
C/Hong Kong/60430/2018 Feb E3(am3)
C/Hong Kong/60430/2018 Feb
C/Hong Kong/3363/2018 Jan
C/Hong Kong/47144/2018 Feb
C/Hong Kong/33417/2018 Feb
C/Hong Kong/96417/2018 Apr
C/Hong Kong/3286/2018 Jan
C/Hong Kong/28015/2018 Jan
87
C/Hong Kong/1/2018 Apr MDCK3-ori
C/Hong Kong/1/2018 Apr E3(am3)
C/Hong Kong/1/2018 Apr
C/Hong Kong/30248/2018 Feb
C/Hong Kong/57069/2018 Feb [S165G]
81
C/Hong Kong/6573/2016 Jan
C/Tokyo/1/2014 Feb
C/Yamagata/6/2014 Apr
C/India/P121719/2012 Feb
C/Leyte/3/2011 [N216I]
C/Hong Kong/6451/2016 Jan
C/Leyte/2/2011
C/Hong Kong/3586/2016 Jan
C/Leyte/1/2011
C/Hong Kong/27295/2018 Jan
C/Hong Kong/17233/2016 Feb
C/Hong Kong/1498/2016 Jan
C/Hong Kong/2361/2016 Jan
C/Hong Kong/16909/2016 Feb
C/Hong Kong/6451/2016 Jan E3(am3)
C/Cameroon/13560/2017 Dec
C/Hong Kong/22986/2016 Feb
C/Palawan/1/2013
C/Saitama/3/2000
C/Yamagata/20/2006 Nov
C/Yamagata/1/2007 Jan
C/Miyagi/2/2006 Apr
C/Miyagi/25/2004 Mar
C/Yamagata/14/2006 Aug
C/Miyagi/4/2005 May
73
C/Yamagata/1/2006 Jan
C/Yamagata/2/2005 Jul
C/Yamagata/13/2006 Aug
77
C/Alberta/3087/2011 Mar
C/Scotland/7383/2007 Dec
C/Hiroshima/251/2000
C/Hiroshima/246/2000
C/Hiroshima/250/2000
C/Yamagata/14/2004 May [N183K]
C/Yamagata/3/2004 Apr
97
74
C/Fukuoka/2/2006 Mar
C/Miyagi/42/2004 May
C/Miyagi/12/2004 Feb
C/Miyagi/11/2002 Feb
C/Yamagata/7/2002 Feb
C/Miyagi/47/2004 Jun
71
C/Miyagi/2/2000
C/Miyagi/5/2000
C/Miyagi/4/2002 Jan
C/Miyagi/28/2002 Apr
C/Miyagi/9/96 Jun
C/Yamagata/5/2002 Feb
C/Miyagi/31/2002 Jun
92
C/Yamagata/3/96
C/Yamagata/8/96 Jun
C/Miyagi/2/92
C/Miyagi/9/91
C/Yamagata/10/89 Aug
C/Minnesota/33/2015 Mar
C/Minnesota/08/2015 Mar
C/Minnesota/19/2015 Mar
C/Minnesota/29/2015 Feb
C/Minnesota/23/2015 Apr
H3P
86
76
C/Minnesota/24/2015 Apr
C/Minnesota/22/2015 Mar
C/Minnesota/18/2015 Mar
C/Minnesota/04/2015 Feb
C/Hong Kong/45913/2018 Feb
85
76
C/Hong Kong/95361/2018 Apr E3(am3)
C/Hong Kong/95361/2018 Apr
C/Hong Kong/57791/2018 Feb
C/Bretagne/2503/2013 Dec [R157K]
C/Hong Kong/13962/2018 Jan
C/Victoria/1/2011
C/Hong Kong/7920/2018 Jan
C/Hong Kong/27990/2018 Jan
73
C/Hong Kong/94533/2018 Apr
C/Hong Kong/1897/2016 Jan
K96R
A189S
C/Miyagi/6/2014 Jun
C/Yamagata/30/2014 Jun
C/Yamagata/32/2014 Jun
S165G
88
R167K
C/Hong Kong/37128/2018 Feb E3(am3)
C/Hong Kong/37128/2018 Feb
C/Hong Kong/2957/2016 Jan E3(am3)
C/Hong Kong/2957/2016 Jan
71
C/Hong Kong/6091/2016 Jan [M113*]
C/Hong Kong/6557/2016 Jan [H131R]
C/Alberta/4406/2011 Apr
C/Tokyo/3/2010 Apr
S52F
C/Scotland/6855/2007 Nov [R157*]
C/Scotland/7274/2007 Dec
C/Miyagi/2/2008 Feb
C/Sendai/TU2/2008
C/Yamagata/18/2008 Jun
94
C/Yamagata/3/2008 Mar
C/Sendai/TU3/2008
74
C/Miyagi/4/2006 Jun
C/Yamagata/9/2006 Jun
C/Sendai/TU1/2008 [K227R]
C/Fukuoka/1/2005 Dec
C/Miyagi/2/2012 Jan
C/Yamagata/14/2012 Jun
89
C/Sendai/TU1/2012
C/Yamagata/3/2012 Feb
T189A
89
C/Yamagata/29/2012 Jul
C/Alberta/3502/2011 Mar [R159S]
C/Alberta/2616/2011 Feb
C/Sendai/TU5/2008
C/Yamagata/16/2008 May [D134N]
C/Yamagata/11/2008 Apr
C/Eastern India/1202/2011 Jul
C/Yamagata/15/2004 May
C/Saitama/1/2000
C/Hiroshima/290/99
C/Miyagi/20/2002 Mar
C/Hiroshima/248/2000
C/Miyagi/3/2000
C/Yamagata/9/2000
C/Yamagata/8/2000
C/Yamagata/6/2000 Mar [S76N]
R232K
C/Yamagata/2/2000
C/Yamagata/2/99
91
C/Miyagi/3/99
C/Miyagi/4/2000
C/Miyagi/1/99
C/Yamagata/13/98 Jun
C/Miyagi/2/96
C/Yamagata/9/96
C/Miyagi/2/98
C/Miyagi/6/96
C/Yamagata/20/96
C/Miyagi/1/97
M62I
87
C/Hiroshima/252/99
C/Hiroshima/249/2000
C/Hiroshima/247/2000
T189A
89
C/Yamagata/2/2010 Feb
C/Miyagi/3/2010 Apr
C/Fukuoka/1/2012 Apr
C/Sendai/TU2/2012
C/Yamagata/6/98
C/Yamagata/2/98
C/Miyagi/4/98
99
C/Yamagata/27/2004 May
C/Yamagata/18/2004 May
C/Yamagata/3/2005 Sep
C/Yamagata/1/2005 Jun
92
C/Miyagi/8/96 May
C/Aichi/1/99 Apr
C/England/892/83
C/Yamagata/26/81 Mar
C/Yamagata/7/81 Mar
C/Yamagata/10/81 Mar
C/Shizuoka/79
C/Nara/1/85
97
C/Yamagata/8/88
C/Yamagata/7/88 Nov
C/Yamagata/1/88
C/Nara/2/85
C/Kyoto/1/79
S215N
87
C/Miyagi/1/94
C/Miyagi/3/94
96
C/Miyagi/1/93 Feb
C/Miyagi/2/93
79
C/Miyagi/6/93
R157K
C/Saitama/2/2000
97
C/Yamagata/3/2000 Feb
C/Miyagi/2/94
84
C/Miyagi/7/96
C/Miyagi/4/96
C/Yamagata/26/2004 May
C/Miyagi/7/93 May [D77G]
C/New Jersey/76
C/California/78
77
C/Miyagi/77
C/Kanagawa/1/76
C/Sapporo/71
89
C/Miyagi/5/93
77
C/Yamagata/1/93 Apr
C/Sao Paulo/378/82
C/Aomori/74
C/Yamagata/9/88 Dec
C/Yamagata/4/92 Nov
C/Yamagata/1/86
C/Miyagi/1/90
C/Miyagi/5/91 Feb
C/Miyagi/7/91
S165G
C/Aichi/1/81
95
C/Kansas/1/79 [V162I]
70
C/Kyoto/41/82
C/Hyogo/1/83
80
C/Nara/82
T10A
C/Johannesburg/1/66
C/Johannesburg/4/67
86
C/Georgia/1/69
C/Greece/1/79
C/Mississippi/80 [M62I]
78
100
V162I
C/Miyagi/4/93
C/Miyagi/3/93 Apr
89
C/Yamagata/5/92 Nov
C/Yamagata/64
C/JJ/50 [M182I]
C/Ann Arbor/1/50
C/Great Lakes/1167/54
C/Paris/1/67 [V162I]
C/Taylor/1233/47 [A24T, D133N]
0.002

G: CM2

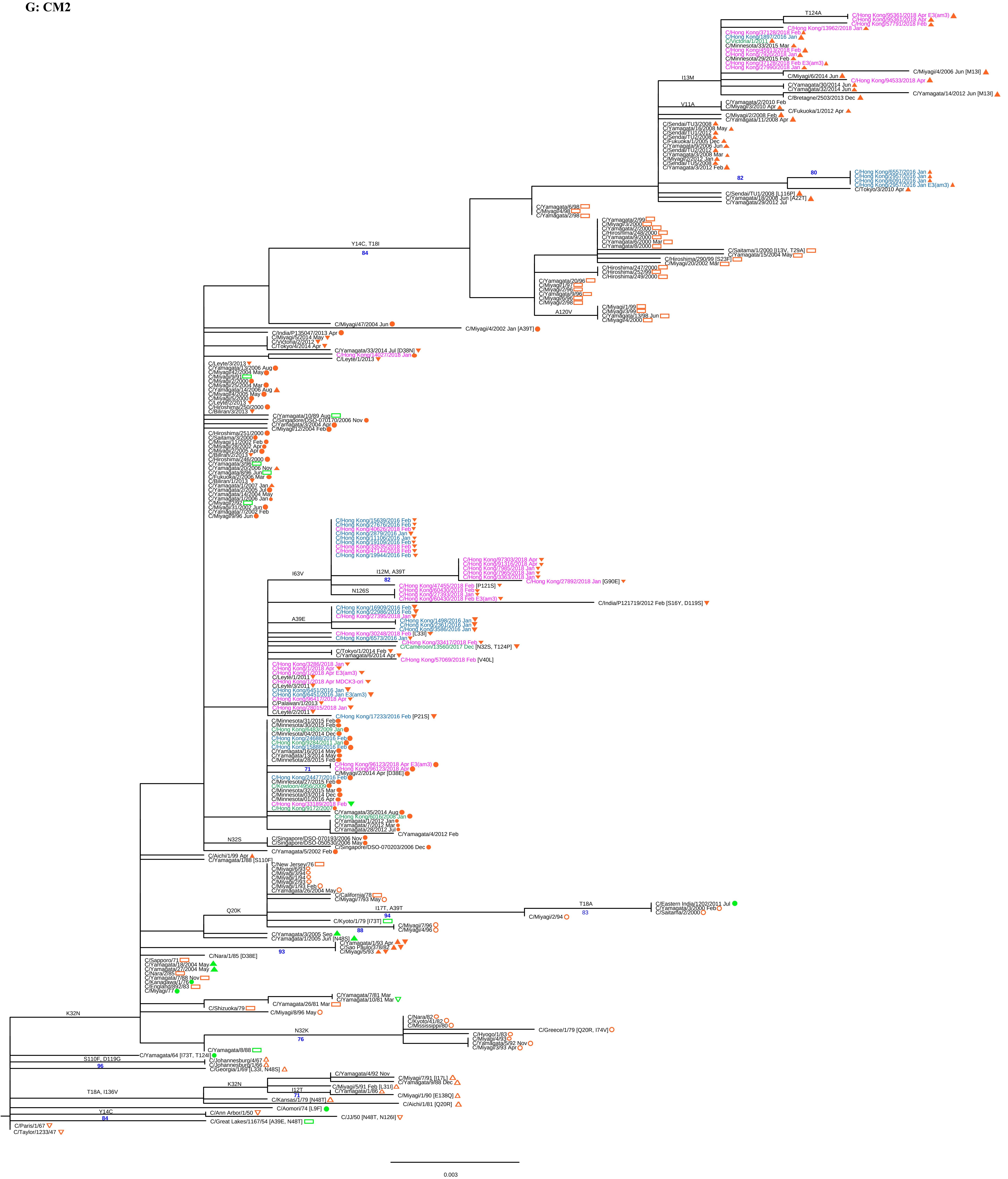

T124A
C/Hong Kong/95361/2018 Apr E3(am3)
C/Hong Kong/95361/2018 Apr
C/Hong Kong/57791/2018 Feb
C/Hong Kong/13962/2018 Jan
C/Hong Kong/37128/2018 Feb
C/Hong Kong/1897/2016 Jan
C/Victoria/1/2011
C/Minnesota/33/2015 Mar
C/Hong Kong/45913/2018 Feb
C/Hong Kong/7920/2018 Jan
C/Minnesota/29/2015 Feb
C/Hong Kong/37128/2018 Feb E3(am3)
C/Hong Kong/27990/2018 Jan
I13M
C/Miyagi/4/2006 Jun [M13I]
C/Miyagi/6/2014 Jun
C/Hong Kong/94533/2018 Apr
C/Yamagata/30/2014 Jun
C/Yamagata/32/2014 Jun
C/Yamagata/14/2012 Jun [M13I]
C/Bretagne/2503/2013 Dec
V11A
C/Yamagata/2/2010 Feb
C/Miyagi/3/2010 Apr
C/Fukuoka/1/2012 Apr
C/Miyagi/2/2008 Feb
C/Yamagata/11/2008 Apr
C/Sendai/TU3/2008
C/Yamagata/16/2008 May
C/Sendai/TU1/2012
C/Sendai/TU2/2008
C/Fukuoka/1/2005 Dec
C/Yamagata/9/2006 Jun
C/Sendai/TU2/2012
C/Yamagata/3/2008 Mar
C/Miyagi/2/2012 Jan
C/Sendai/TU5/2008
C/Yamagata/3/2012 Feb
80
C/Hong Kong/6557/2016 Jan
C/Hong Kong/2957/2016 Jan
C/Hong Kong/6091/2016 Jan
C/Hong Kong/2957/2016 Jan E3(am3)
82
C/Tokyo/3/2010 Apr
C/Sendai/TU1/2008 [L116P]
C/Yamagata/18/2008 Jun [A22T]
C/Yamagata/29/2012 Jul
C/Yamagata/6/98
C/Miyagi/4/98
C/Yamagata/2/98
C/Yamagata/2/99
C/Miyagi/3/2000
C/Yamagata/2/2000
C/Hiroshima/248/2000
C/Yamagata/9/2000
C/Yamagata/6/2000 Mar
C/Yamagata/8/2000
C/Saitama/1/2000 [I13V, T29A]
C/Yamagata/15/2004 May
C/Hiroshima/290/99 [S23F]
C/Miyagi/20/2002 Mar
C/Hiroshima/247/2000
C/Hiroshima/252/99
C/Hiroshima/249/2000
Y14C, T18I
84
C/Yamagata/20/96
C/Miyagi/1/97
C/Miyagi/2/96
C/Yamagata/9/96
C/Miyagi/6/96
C/Miyagi/2/98
A120V
C/Miyagi/1/99
C/Miyagi/3/99
C/Yamagata/13/98 Jun
C/Miyagi/4/2000
C/Miyagi/47/2004 Jun
C/Miyagi/4/2002 Jan [A39T]
C/India/P135047/2013 Apr
C/Miyagi/5/2014 May
C/Victoria/2/2012
C/Tokyo/4/2014 Apr
C/Yamagata/33/2014 Jul [D38N]
C/Hong Kong/14027/2018 Jan
C/Leyte/1/2013
C/Leyte/3/2013
C/Yamagata/13/2006 Aug
C/Miyagi/42/2004 May
C/Miyagi/9/91
C/Miyagi/2/2000
C/Miyagi/25/2004 Mar
C/Yamagata/14/2006 Aug
C/Miyagi/4/2005 May
C/Miyagi/5/2000
C/Leyte/2/2013
C/Hiroshima/250/2000
C/Biliran/3/2013
C/Yamagata/10/89 Aug
C/Singapore/DSO-070170/2006 Nov
C/Yamagata/3/2004 Apr
C/Miyagi/12/2004 Feb
C/Hiroshima/251/2000
C/Saitama/3/2000
C/Miyagi/11/2002 Feb
C/Miyagi/28/2002 Apr
C/Miyagi/2/2005 Apr
C/Biliran/2/2013
C/Hiroshima/246/2000
C/Yamagata/3/96
C/Yamagata/20/2006 Nov
C/Yamagata/8/96 Jun
C/Fukuoka/2/2006 Mar
C/Biliran/1/2013
C/Yamagata/1/2007 Jan
C/Yamagata/2/2005 Jul
C/Yamagata/14/2004 May
C/Yamagata/1/2006 Jan
C/Miyagi/2/92
C/Miyagi/31/2002 Jun
C/Yamagata/7/2002 Feb
C/Miyagi/9/96 Jun
C/Hong Kong/15639/2016 Feb
C/Hong Kong/27676/2016 Feb
C/Hong Kong/40626/2018 Feb
C/Hong Kong/2879/2016 Jan
C/Hong Kong/11106/2016 Jan
C/Hong Kong/19109/2016 Feb
C/Hong Kong/33535/2018 Feb
C/Hong Kong/47144/2018 Feb
C/Hong Kong/19944/2016 Feb
C/Hong Kong/97303/2018 Apr
C/Hong Kong/91316/2018 Apr
C/Hong Kong/7985/2018 Jan
C/Hong Kong/7965/2018 Jan
C/Hong Kong/3363/2018 Jan
C/Hong Kong/27892/2018 Jan [G90E]
I63V
I12M, A39T
82
C/Hong Kong/47455/2018 Feb [P121S]
C/Hong Kong/60430/2018 Feb
C/Hong Kong/27393/2018 Jan
C/Hong Kong/60430/2018 Feb E3(am3)
N126S
C/India/P121719/2012 Feb [S16Y, D119S]
C/Hong Kong/16909/2016 Feb
C/Hong Kong/22986/2016 Feb
C/Hong Kong/27395/2018 Jan
A39E
C/Hong Kong/1498/2016 Jan
C/Hong Kong/2361/2016 Jan
C/Hong Kong/3586/2016 Jan
C/Hong Kong/30248/2018 Feb [L33I]
C/Hong Kong/6573/2016 Jan
C/Hong Kong/33417/2018 Feb
C/Cameroon/13560/2017 Dec [N32S, T124P]
C/Tokyo/1/2014 Feb
C/Yamagata/6/2014 Apr
C/Hong Kong/57069/2018 Feb [V40L]
C/Hong Kong/3286/2018 Jan
C/Hong Kong/1/2018 Apr
C/Hong Kong/1/2018 Apr E3(am3)
C/Leyte/1/2011
C/Hong Kong/1/2018 Apr MDCK3-ori
C/Leyte/3/2011
C/Hong Kong/6451/2016 Jan
C/Hong Kong/6451/2016 Jan E3(am3)
C/Hong Kong/96417/2018 Apr
C/Palawan/1/2013
C/Hong Kong/28015/2018 Jan
C/Leyte/2/2011
C/Hong Kong/17233/2016 Feb [P21S]
C/Minnesota/31/2015 Feb
C/Minnesota/30/2015 Feb
C/Hong Kong/6483/2009 Jan
C/Minnesota/04/2014 Dec
C/Hong Kong/24688/2016 Feb
C/Hong Kong/9284/2011 Jan
C/Hong Kong/15888/2016 Feb
C/Yamagata/16/2014 May
C/Yamagata/13/2014 May
C/Minnesota/28/2015 Feb
C/Hong Kong/96123/2018 Apr E3(am3)
C/Hong Kong/96123/2018 Apr
71
C/Miyagi/2/2014 Apr [D38E]
C/Hong Kong/24477/2016 Feb
C/Minnesota/27/2015 Feb
C/Kowloon/4956/2009
C/Minnesota/32/2015 Mar
C/Minnesota/03/2014 Dec
C/Minnesota/01/2016 Apr
C/Hong Kong/33189/2018 Feb
C/Hong Kong/9172/2007
C/Yamagata/35/2014 Aug
C/Hong Kong/6016/2008 Jan
C/Yamagata/1/2012 Jan
C/Yamagata/7/2012 Mar
C/Yamagata/28/2012 Jul
C/Yamagata/4/2012 Feb
N32S
C/Singapore/DSO-070193/2006 Nov
C/Singapore/DSO-050530/2006 May
C/Singapore/DSO-070203/2006 Dec
C/Yamagata/5/2002 Feb
C/Aichi/1/99 Apr
C/Yamagata/1/88 [S110F]
C/New Jersey/76
C/Miyagi/6/93
C/Miyagi/3/94
C/Miyagi/1/94
C/Miyagi/2/93
C/Miyagi/1/93 Feb
C/Yamagata/26/2004 May
C/California/78
C/Miyagi/7/93 May
T18A
C/Eastern India/1202/2011 Jul
C/Yamagata/3/2000 Feb
C/Saitama/2/2000
83
Q20K
I17T, A39T
C/Miyagi/2/94
94
C/Kyoto/1/79 [I73T]
C/Miyagi/7/96
C/Miyagi/4/96
88
C/Yamagata/3/2005 Sep
C/Yamagata/1/2005 Jun [N48S]
C/Yamagata/1/93 Apr
C/Sao Paulo/378/82
C/Miyagi/5/93
93
C/Nara/1/85 [D38E]
C/Sapporo/71
C/Yamagata/18/2004 May
C/Yamagata/27/2004 May
C/Nara/2/85
C/Yamagata/7/88 Nov
C/Kanagawa/1/76
C/England/892/83
C/Miyagi/77
C/Yamagata/7/81 Mar
C/Yamagata/10/81 Mar
C/Yamagata/26/81 Mar
C/Shizuoka/79
C/Miyagi/8/96 May
K32N
C/Nara/82
C/Kyoto/41/82
C/Mississippi/80
N32K
76
C/Greece/1/79 [Q20R, I74V]
C/Hyogo/1/83
C/Miyagi/4/93
C/Yamagata/5/92 Nov
C/Miyagi/3/93 Apr
C/Yamagata/8/88
C/Yamagata/64 [I73T, T124I]
S110F, D119G
96
C/Johannesburg/4/67
C/Johannesburg/1/66
C/Georgia/1/69 [L33I, N48S]
C/Yamagata/4/92 Nov
C/Miyagi/7/91 [I17L]
C/Yamagata/9/88 Dec
K32N
C/Miyagi/5/91 Feb [L31I]
C/Yamagata/1/86
I12T
71
T18A, I136V
C/Miyagi/1/90 [E138Q]
C/Kansas/1/79 [N48T]
C/Aichi/1/81 [Q20R]
C/Aomori/74 [L9F]
Y14C
C/Ann Arbor/1/50
84
C/JJ/50 [N48T, N126I]
C/Great Lakes/1167/54 [A39E, N48T]
C/Paris/1/67
C/Taylor/1233/47
0.003

H: NS1

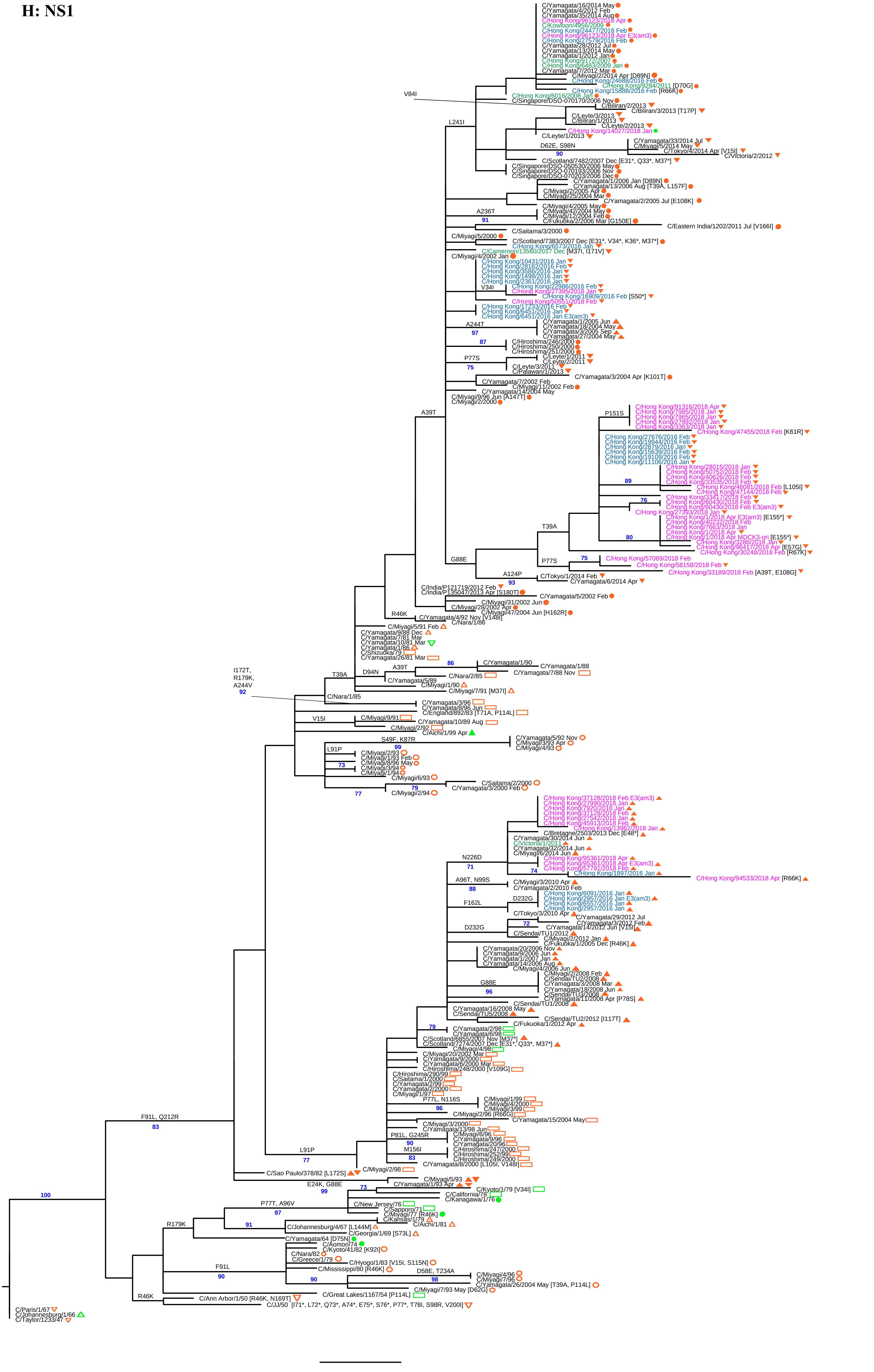
C/Yamagata/16/2014 May
C/Yamagata/4/2012 Feb
C/Yamagata/35/2014 Aug
C/Hong Kong/96123/2018 Apr
C/Kowloon/4956/2009
C/Hong Kong/24477/2016 Feb
C/Hong Kong/96123/2018 Apr E3(am3)
C/Hong Kong/27579/2016 Feb
C/Yamagata/28/2012 Jul
C/Yamagata/13/2014 May
C/Yamagata/1/2012 Jan
C/Hong Kong/9172/2007
C/Hong Kong/6483/2009 Jan
C/Yamagata/7/2012 Mar
C/Miyagi/2/2014 Apr [D89N]
C/Hong Kong/24688/2016 Feb
C/Hong Kong/9284/2011 [D70G]
C/Hong Kong/15888/2016 Feb [R66K]
V84I
C/Hong Kong/6016/2008 Jan
C/Singapore/DSO-070170/2006 Nov
C/Biliran/2/2013
C/Biliran/3/2013 [T17P]
L241I
C/Leyte/3/2013
C/Biliran/1/2013
C/Leyte/2/2013
C/Hong Kong/14027/2018 Jan
C/Leyte/1/2013
C/Yamagata/33/2014 Jul
D62E, S98N
C/Miyagi/5/2014 May
90
C/Tokyo/4/2014 Apr [V15I]
C/Victoria/2/2012
C/Scotland/7482/2007 Dec [E31*, Q33*, M37*]
C/Singapore/DSO-050530/2006 May
C/Singapore/DSO-070193/2006 Nov
C/Singapore/DSO-070203/2006 Dec
C/Yamagata/1/2006 Jan [D89N]
C/Yamagata/13/2006 Aug [T39A, L157F]
C/Miyagi/2/2005 Apr
C/Miyagi/25/2004 Mar
C/Yamagata/2/2005 Jul [E108K]
C/Miyagi/4/2005 May
A236T
C/Miyagi/42/2004 May
91
C/Miyagi/12/2004 Feb
C/Fukuoka/2/2006 Mar [G150E]
C/Eastern India/1202/2011 Jul [V166I]
C/Saitama/3/2000
C/Miyagi/5/2000
C/Scotland/7383/2007 Dec [E31*, V34*, K36*, M37*]
C/Hong Kong/6573/2016 Jan
C/Cameroon/13560/2017 Dec [M37I, I171V]
C/Miyagi/4/2002 Jan
C/Hong Kong/10431/2016 Jan
C/Hong Kong/28182/2016 Feb
C/Hong Kong/3586/2016 Jan
C/Hong Kong/1498/2016 Jan
C/Hong Kong/2361/2016 Jan
C/Hong Kong/22986/2016 Feb
V34I
C/Hong Kong/27395/2018 Jan
C/Hong Kong/16909/2016 Feb [S50*]
C/Hong Kong/50551/2018 Feb
C/Hong Kong/17233/2016 Feb
C/Hong Kong/6451/2016 Jan
C/Hong Kong/6451/2016 Jan E3(am3)
C/Yamagata/1/2005 Jun
A244T
C/Yamagata/18/2004 May
97
C/Yamagata/3/2005 Sep
C/Yamagata/27/2004 May
87
C/Hiroshima/246/2000
C/Hiroshima/250/2000
C/Hiroshima/251/2000
P77S
C/Leyte/1/2011
C/Leyte/2/2011
75
C/Leyte/3/2011
C/Palawan/1/2013
C/Yamagata/3/2004 Apr [K101T]
C/Yamagata/7/2002 Feb
C/Miyagi/11/2002 Feb
C/Yamagata/14/2004 May
C/Miyagi/9/96 Jun [A147T]
C/Miyagi/2/2000
A39T
C/Hong Kong/91316/2018 Apr
P151S
C/Hong Kong/7985/2018 Jan
C/Hong Kong/7965/2018 Jan
C/Hong Kong/27892/2018 Jan
C/Hong Kong/3363/2018 Jan
C/Hong Kong/47455/2018 Feb [K61R]
C/Hong Kong/27676/2016 Feb
C/Hong Kong/19944/2016 Feb
C/Hong Kong/2879/2016 Jan
C/Hong Kong/15639/2016 Feb
C/Hong Kong/19109/2016 Feb
C/Hong Kong/11106/2016 Jan
C/Hong Kong/28015/2018 Jan
C/Hong Kong/50752/2018 Feb
C/Hong Kong/40626/2018 Feb
89
C/Hong Kong/33535/2018 Feb
C/Hong Kong/46081/2018 Feb [L105I]
C/Hong Kong/47144/2018 Feb
C/Hong Kong/33417/2018 Feb
76
C/Hong Kong/60430/2018 Feb
C/Hong Kong/60430/2018 Feb E3(am3)
C/Hong Kong/27393/2018 Jan
C/Hong Kong/1/2018 Apr E3(am3) [E155*]
T39A
C/Hong Kong/40232/2018 Feb
C/Hong Kong/7663/2018 Jan
C/Hong Kong/1/2018 Apr
80
C/Hong Kong/1/2018 Apr MDCK3-ori [E155*]
C/Hong Kong/3286/2018 Jan
C/Hong Kong/96417/2018 Apr [E57G]
G88E
C/Hong Kong/30248/2018 Feb [R67K]
P77S
75
C/Hong Kong/57069/2018 Feb
C/Hong Kong/58158/2018 Feb
C/Hong Kong/33189/2018 Feb [A39T, E108G]
A124P
C/Tokyo/1/2014 Feb
93
C/Yamagata/6/2014 Apr
C/India/P121719/2012 Feb
C/India/P135047/2013 Apr [S180T]
C/Yamagata/5/2002 Feb
C/Miyagi/31/2002 Jun
C/Miyagi/28/2002 Apr
C/Miyagi/47/2004 Jun [H162R]
R46K
C/Yamagata/4/92 Nov [V148I]
C/Nara/1/86
C/Miyagi/5/91 Feb
C/Yamagata/9/88 Dec
C/Yamagata/7/81 Mar
C/Yamagata/10/81 Mar
C/Yamagata/1/86
C/Shizuoka/79
C/Yamagata/26/81 Mar
86
C/Yamagata/1/90
C/Yamagata/1/88
C/Yamagata/7/88 Nov
A39T
D94N
C/Nara/2/85
T39A
C/Yamagata/5/89
C/Miyagi/1/90
I172T,
R179K,
A244V
92
C/Miyagi/7/91 [M37I]
C/Nara/1/85
C/Yamagata/3/96
C/Yamagata/8/96 Jun
C/England/892/83 [T71A, P114L]
V15I
C/Miyagi/9/91
C/Yamagata/10/89 Aug
C/Miyagi/2/92
C/Aichi/1/99 Apr
S49F, K87R
C/Yamagata/5/92 Nov
99
C/Miyagi/3/93 Apr
C/Miyagi/4/93
L91P
C/Miyagi/2/93
C/Miyagi/1/93 Feb
C/Miyagi/8/96 May
73
C/Miyagi/3/94
C/Miyagi/1/94
C/Miyagi/6/93
C/Saitama/2/2000
79
C/Yamagata/3/2000 Feb
77
C/Miyagi/2/94
C/Hong Kong/37128/2018 Feb E3(am3)
C/Hong Kong/27990/2018 Jan
C/Hong Kong/7920/2018 Jan
C/Hong Kong/37128/2018 Feb
C/Hong Kong/27542/2018 Jan
C/Hong Kong/45913/2018 Feb
C/Hong Kong/13962/2018 Jan
C/Bretagne/2503/2013 Dec [E48*]
C/Yamagata/30/2014 Jun
C/Victoria/1/2011
C/Yamagata/32/2014 Jun
C/Miyagi/6/2014 Jun
N226D
C/Hong Kong/95361/2018 Apr
71
C/Hong Kong/95361/2018 Apr E3(am3)
C/Hong Kong/57791/2018 Feb
74
C/Hong Kong/1897/2016 Jan
C/Hong Kong/94533/2018 Apr [R66K]
A96T, N99S
C/Miyagi/3/2010 Apr
88
C/Yamagata/2/2010 Feb
C/Hong Kong/6091/2016 Jan
D232G
C/Hong Kong/2957/2016 Jan E3(am3)
F162L
C/Hong Kong/6557/2016 Jan
C/Hong Kong/2957/2016 Jan
C/Tokyo/3/2010 Apr
C/Yamagata/29/2012 Jul
72
C/Yamagata/3/2012 Feb
D232G
C/Yamagata/14/2012 Jun [V15I]
C/Sendai/TU1/2012
C/Miyagi/2/2012 Jan
C/Fukuoka/1/2005 Dec [R46K]
C/Yamagata/20/2006 Nov
C/Yamagata/9/2006 Jun
C/Yamagata/1/2007 Jan
C/Yamagata/14/2006 Aug
C/Miyagi/4/2006 Jun
C/Miyagi/2/2008 Feb
C/Sendai/TU2/2008
G88E
C/Yamagata/3/2008 Mar
96
C/Yamagata/18/2008 Jun
C/Sendai/TU3/2008
C/Yamagata/11/2008 Apr [P78S]
C/Sendai/TU1/2008
C/Yamagata/16/2008 May
C/Sendai/TU5/2008
C/Sendai/TU2/2012 [I117T]
C/Fukuoka/1/2012 Apr
79
C/Yamagata/2/98
C/Yamagata/6/98
C/Scotland/6855/2007 Nov [M37*]
C/Scotland/7274/2007 Dec [E31*, Q33*, M37*]
C/Miyagi/4/98
C/Miyagi/20/2002 Mar
C/Yamagata/9/2000
C/Yamagata/6/2000 Mar
C/Hiroshima/248/2000 [V109G]
C/Hiroshima/290/99
C/Saitama/1/2000
C/Yamagata/2/99
C/Yamagata/2/2000
C/Miyagi/1/97
P77L, N116S
C/Miyagi/1/99
C/Miyagi/4/2000
96
C/Miyagi/3/99
C/Miyagi/2/96 [R66G]
C/Yamagata/15/2004 May
F91L, Q212R
83
C/Miyagi/3/2000
C/Yamagata/13/98 Jun
P81L, G245R
C/Miyagi/6/96
90
C/Yamagata/9/96
C/Yamagata/20/96
M156I
C/Hiroshima/247/2000
C/Hiroshima/252/99
83
C/Hiroshima/249/2000
L91P
C/Yamagata/8/2000 [L105I, V148I]
77
C/Sao Paulo/378/82 [L172S]
C/Miyagi/2/98
C/Miyagi/5/93
E24K, G88E
C/Yamagata/1/93 Apr
73
99
C/Kyoto/1/79 [V34I]
100
C/California/78
C/Kanagawa/1/76
P77T, A96V
C/New Jersey/76
97
C/Sapporo/71
C/Miyagi/77 [R46K]
C/Kansas/1/79
R179K
91
C/Johannesburg/4/67 [L144M]
C/Aichi/1/81
C/Georgia/1/69 [S73L]
C/Yamagata/64 [D75N]
C/Aomori/74
C/Kyoto/41/82 [K92I]
C/Nara/82
C/Greece/1/79
C/Hyogo/1/83 [V15I, S115N]
F91L
C/Mississippi/80 [R46K]
90
D58E, T234A
C/Miyagi/4/96
90
98
C/Miyagi/7/96
C/Yamagata/26/2004 May [T39A, P114L]
C/Great Lakes/1167/54 [P114L]
C/Miyagi/7/93 May [D62G]
R46K
C/Ann Arbor/1/50 [R46K, N169T]
C/JJ/50 [I71*, L72*, Q73*, A74*, E75*, S76*, P77*, T78I, S98R, V200I]
C/Paris/1/67
C/Johannesburg/1/66
C/Taylor/1233/47
0.003

I: NS2

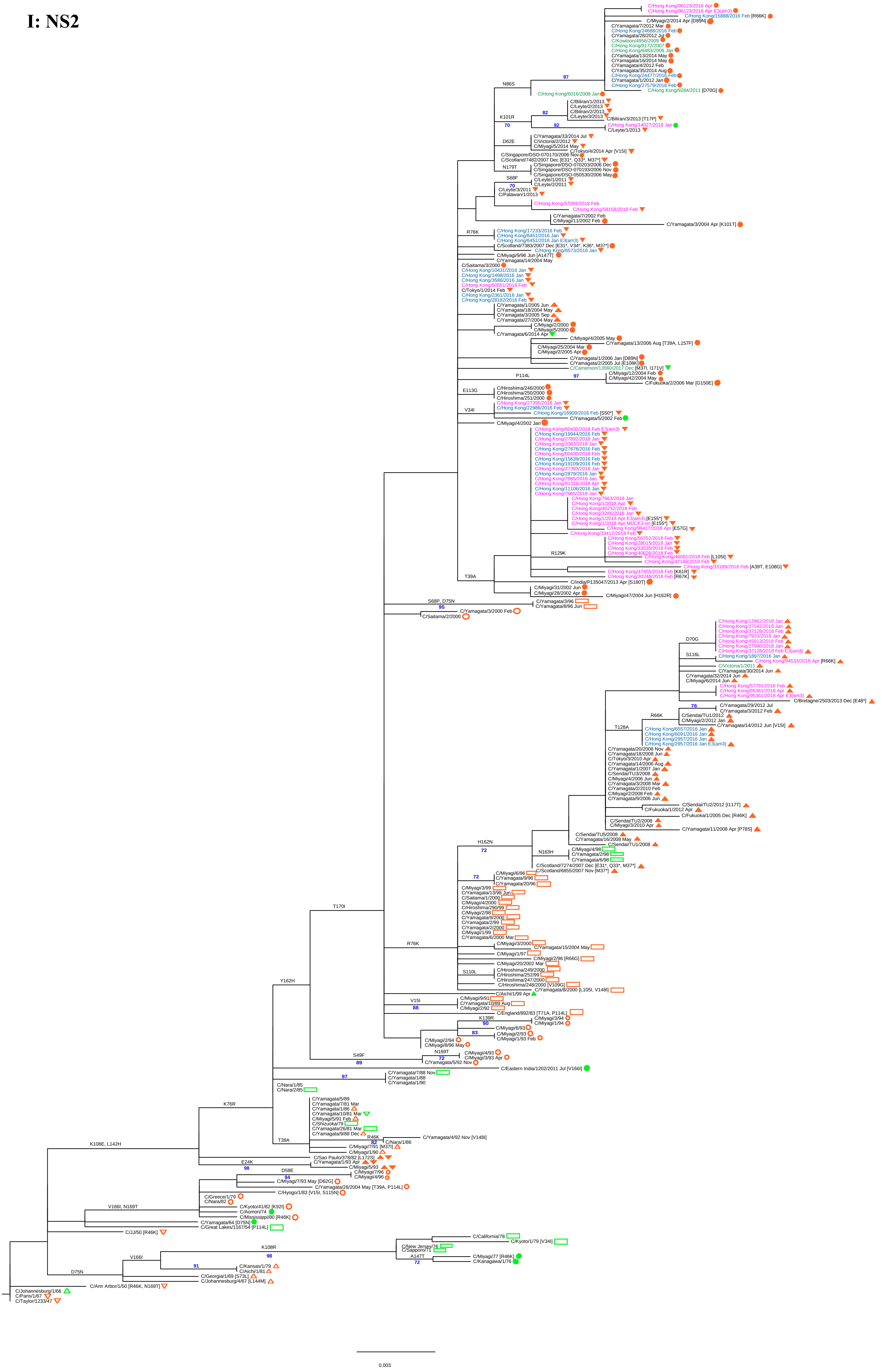

C/Hong Kong/96123/2018 Apr
C/Hong Kong/96123/2018 Apr E3(am3)
C/Hong Kong/15888/2016 Feb [R66K]
C/Miyagi/2/2014 Apr [D89N]
C/Yamagata/7/2012 Mar
C/Hong Kong/24688/2016 Feb
C/Yamagata/28/2012 Jul
C/Kowloon/4956/2009
C/Hong Kong/9172/2007
C/Hong Kong/6483/2009 Jan
C/Yamagata/13/2014 May
C/Yamagata/16/2014 May
C/Yamagata/4/2012 Feb
C/Yamagata/35/2014 Aug
C/Hong Kong/24477/2016 Feb
C/Yamagata/1/2012 Jan
C/Hong Kong/27579/2016 Feb
C/Hong Kong/9284/2011 [D70G]
87
N86S
C/Hong Kong/6016/2008 Jan
C/Biliran/1/2013
C/Leyte/2/2013
C/Biliran/2/2013
C/Leyte/3/2013
82
K101R
C/Biliran/3/2013 [T17P]
70
92
C/Hong Kong/14027/2018 Jan
C/Leyte/1/2013
C/Yamagata/33/2014 Jul
C/Victoria/2/2012
C/Miyagi/5/2014 May
D62E
C/Tokyo/4/2014 Apr [V15I]
C/Singapore/DSO-070170/2006 Nov
C/Scotland/7482/2007 Dec [E31*, Q33*, M37*]
N179T
C/Singapore/DSO-070203/2006 Dec
C/Singapore/DSO-070193/2006 Nov
C/Singapore/DSO-050530/2006 May
S68F
C/Leyte/1/2011
C/Leyte/2/2011
70
C/Leyte/3/2011
C/Palawan/1/2013
C/Hong Kong/57069/2018 Feb
C/Hong Kong/58158/2018 Feb
C/Yamagata/7/2002 Feb
C/Miyagi/11/2002 Feb
C/Yamagata/3/2004 Apr [K101T]
R76K
C/Hong Kong/17233/2016 Feb
C/Hong Kong/6451/2016 Jan
C/Hong Kong/6451/2016 Jan E3(am3)
C/Scotland/7383/2007 Dec [E31*, V34*, K36*, M37*]
C/Hong Kong/6573/2016 Jan
C/Miyagi/9/96 Jun [A147T]
C/Yamagata/14/2004 May
C/Saitama/3/2000
C/Hong Kong/10431/2016 Jan
C/Hong Kong/1498/2016 Jan
C/Hong Kong/3586/2016 Jan
C/Hong Kong/50551/2018 Feb
C/Tokyo/1/2014 Feb
C/Hong Kong/2361/2016 Jan
C/Hong Kong/28182/2016 Feb
C/Yamagata/1/2005 Jun
C/Yamagata/18/2004 May
C/Yamagata/3/2005 Sep
C/Yamagata/27/2004 May
C/Miyagi/2/2000
C/Miyagi/5/2000
C/Yamagata/6/2014 Apr
C/Miyagi/4/2005 May
C/Yamagata/13/2006 Aug [T39A, L157F]
C/Miyagi/25/2004 Mar
C/Miyagi/2/2005 Apr
C/Yamagata/1/2006 Jan [D89N]
C/Yamagata/2/2005 Jul [E108K]
C/Cameroon/13560/2017 Dec [M37I, I171V]
P114L
97
C/Miyagi/12/2004 Feb
C/Miyagi/42/2004 May
C/Fukuoka/2/2006 Mar [G150E]
E113G
C/Hiroshima/246/2000
C/Hiroshima/250/2000
C/Hiroshima/251/2000
C/Hong Kong/27395/2018 Jan
C/Hong Kong/22986/2016 Feb
V34I
C/Hong Kong/16909/2016 Feb [S50*]
C/Yamagata/5/2002 Feb
C/Miyagi/4/2002 Jan
C/Hong Kong/60430/2018 Feb E3(am3)
C/Hong Kong/19944/2016 Feb
C/Hong Kong/27892/2018 Jan
C/Hong Kong/3363/2018 Jan
C/Hong Kong/27676/2016 Feb
C/Hong Kong/60430/2018 Feb
C/Hong Kong/15639/2016 Feb
C/Hong Kong/19109/2016 Feb
C/Hong Kong/27393/2018 Jan
C/Hong Kong/2879/2016 Jan
C/Hong Kong/7985/2018 Jan
C/Hong Kong/91316/2018 Apr
C/Hong Kong/11106/2016 Jan
C/Hong Kong/7965/2018 Jan
C/Hong Kong/7663/2018 Jan
C/Hong Kong/1/2018 Apr
C/Hong Kong/40232/2018 Feb
C/Hong Kong/3286/2018 Jan
C/Hong Kong/1/2018 Apr E3(am3) [E155*]
C/Hong Kong/1/2018 Apr MDCK3-ori [E155*]
C/Hong Kong/96417/2018 Apr [E57G]
C/Hong Kong/33417/2018 Feb
C/Hong Kong/50752/2018 Feb
C/Hong Kong/28015/2018 Jan
C/Hong Kong/33535/2018 Feb
C/Hong Kong/40626/2018 Feb
R129K
C/Hong Kong/46081/2018 Feb [L105I]
C/Hong Kong/47144/2018 Feb
C/Hong Kong/33189/2018 Feb [A39T, E108G]
C/Hong Kong/47455/2018 Feb [K61R]
C/Hong Kong/30248/2018 Feb [R67K]
T39A
C/India/P135047/2013 Apr [S180T]
C/Miyagi/31/2002 Jun
C/Miyagi/28/2002 Apr
C/Miyagi/47/2004 Jun [H162R]
S68P, D75N
95
C/Yamagata/3/96
C/Yamagata/8/96 Jun
C/Yamagata/3/2000 Feb
C/Saitama/2/2000
C/Hong Kong/13962/2018 Jan
C/Hong Kong/27542/2018 Jan
C/Hong Kong/37128/2018 Feb
C/Hong Kong/7920/2018 Jan
C/Hong Kong/45913/2018 Feb
C/Hong Kong/27990/2018 Jan
C/Hong Kong/37128/2018 Feb E3(am3)
D70G
S116L
C/Hong Kong/1897/2016 Jan
C/Hong Kong/94533/2018 Apr [R66K]
C/Victoria/1/2011
C/Yamagata/30/2014 Jun
C/Yamagata/32/2014 Jun
C/Miyagi/6/2014 Jun
C/Hong Kong/57791/2018 Feb
C/Hong Kong/95361/2018 Apr
C/Hong Kong/95361/2018 Apr E3(am3)
C/Bretagne/2503/2013 Dec [E48*]
76
C/Yamagata/29/2012 Jul
C/Yamagata/3/2012 Feb
R66K
C/Sendai/TU1/2012
C/Miyagi/2/2012 Jan
C/Yamagata/14/2012 Jun [V15I]
T128A
C/Hong Kong/6557/2016 Jan
C/Hong Kong/6091/2016 Jan
C/Hong Kong/2957/2016 Jan
C/Hong Kong/2957/2016 Jan E3(am3)
C/Yamagata/20/2006 Nov
C/Yamagata/18/2008 Jun
C/Tokyo/3/2010 Apr
C/Yamagata/14/2006 Aug
C/Yamagata/1/2007 Jan
C/Sendai/TU3/2008
C/Miyagi/4/2006 Jun
C/Yamagata/3/2008 Mar
C/Yamagata/2/2010 Feb
C/Miyagi/2/2008 Feb
C/Yamagata/9/2006 Jun
C/Sendai/TU2/2012 [I117T]
C/Fukuoka/1/2012 Apr
C/Fukuoka/1/2005 Dec [R46K]
C/Sendai/TU2/2008
C/Miyagi/3/2010 Apr
C/Yamagata/11/2008 Apr [P78S]
C/Sendai/TU5/2008
C/Yamagata/16/2008 May
C/Sendai/TU1/2008
H162N
72
N163H
C/Miyagi/4/98
C/Yamagata/2/98
C/Yamagata/6/98
C/Scotland/7274/2007 Dec [E31*, Q33*, M37*]
C/Scotland/6855/2007 Nov [M37*]
72
C/Miyagi/6/96
C/Yamagata/9/96
C/Yamagata/20/96
C/Miyagi/3/99
C/Yamagata/13/98 Jun
C/Saitama/1/2000
C/Miyagi/4/2000
C/Hiroshima/290/99
C/Miyagi/2/98
C/Yamagata/9/2000
C/Yamagata/2/99
C/Yamagata/2/2000
C/Miyagi/1/99
C/Yamagata/6/2000 Mar
T170I
R76K
C/Miyagi/3/2000
C/Yamagata/15/2004 May
C/Miyagi/1/97
C/Miyagi/2/96 [R66G]
C/Miyagi/20/2002 Mar
S110L
C/Hiroshima/249/2000
C/Hiroshima/252/99
C/Hiroshima/247/2000
C/Hiroshima/248/2000 [V109G]
C/Aichi/1/99 Apr
C/Yamagata/8/2000 [L105I, V148I]
Y162H
V15I
88
C/Miyagi/9/91
C/Yamagata/10/89 Aug
C/Miyagi/2/92
C/England/892/83 [T71A, P114L]
K139R
90
C/Miyagi/3/94
C/Miyagi/1/94
83
C/Miyagi/6/93
C/Miyagi/2/93
C/Miyagi/1/93 Feb
C/Miyagi/2/94
C/Miyagi/8/96 May
S49F
89
N169T
72
C/Miyagi/4/93
C/Miyagi/3/93 Apr
C/Yamagata/5/92 Nov
C/Eastern India/1202/2011 Jul [V166I]
97
C/Yamagata/7/88 Nov
C/Yamagata/1/88
C/Yamagata/1/90
C/Nara/1/85
C/Nara/2/85
K76R
C/Yamagata/5/89
C/Yamagata/7/81 Mar
C/Yamagata/1/86
C/Yamagata/10/81 Mar
C/Miyagi/5/91 Feb
C/Shizuoka/79
C/Yamagata/26/81 Mar
C/Yamagata/9/88 Dec
T39A
R46K
82
C/Nara/1/86
C/Yamagata/4/92 Nov [V148I]
C/Miyagi/7/91 [M37I]
C/Miyagi/1/90
K108E, L142H
C/Sao Paulo/378/82 [L172S]
E24K
98
C/Yamagata/1/93 Apr
C/Miyagi/5/93
D58E
94
C/Miyagi/7/96
C/Miyagi/4/96
C/Miyagi/7/93 May [D62G]
C/Yamagata/26/2004 May [T39A, P114L]
C/Hyogo/1/83 [V15I, S115N]
C/Greece/1/79
C/Nara/82
V166I, N169T
C/Kyoto/41/82 [K92I]
C/Aomori/74
C/Mississippi/80 [R46K]
C/Yamagata/64 [D75N]
C/Great Lakes/1167/54 [P114L]
C/JJ/50 [R46K]
C/California/78
C/Kyoto/1/79 [V34I]
K108R
98
C/New Jersey/76
C/Sapporo/71
A147T
72
C/Miyagi/77 [R46K]
C/Kanagawa/1/76
V166I
91
C/Kansas/1/79
C/Aichi/1/81
D75N
C/Georgia/1/69 [S73L]
C/Johannesburg/4/67 [L144M]
C/Ann Arbor/1/50 [R46K, N169T]
C/Johannesburg/1/66
C/Paris/1/67
C/Taylor/1233/47
0.003

Figure S2. HEF glycoprotein alignment with HE gene and HEF phylogenies.

A

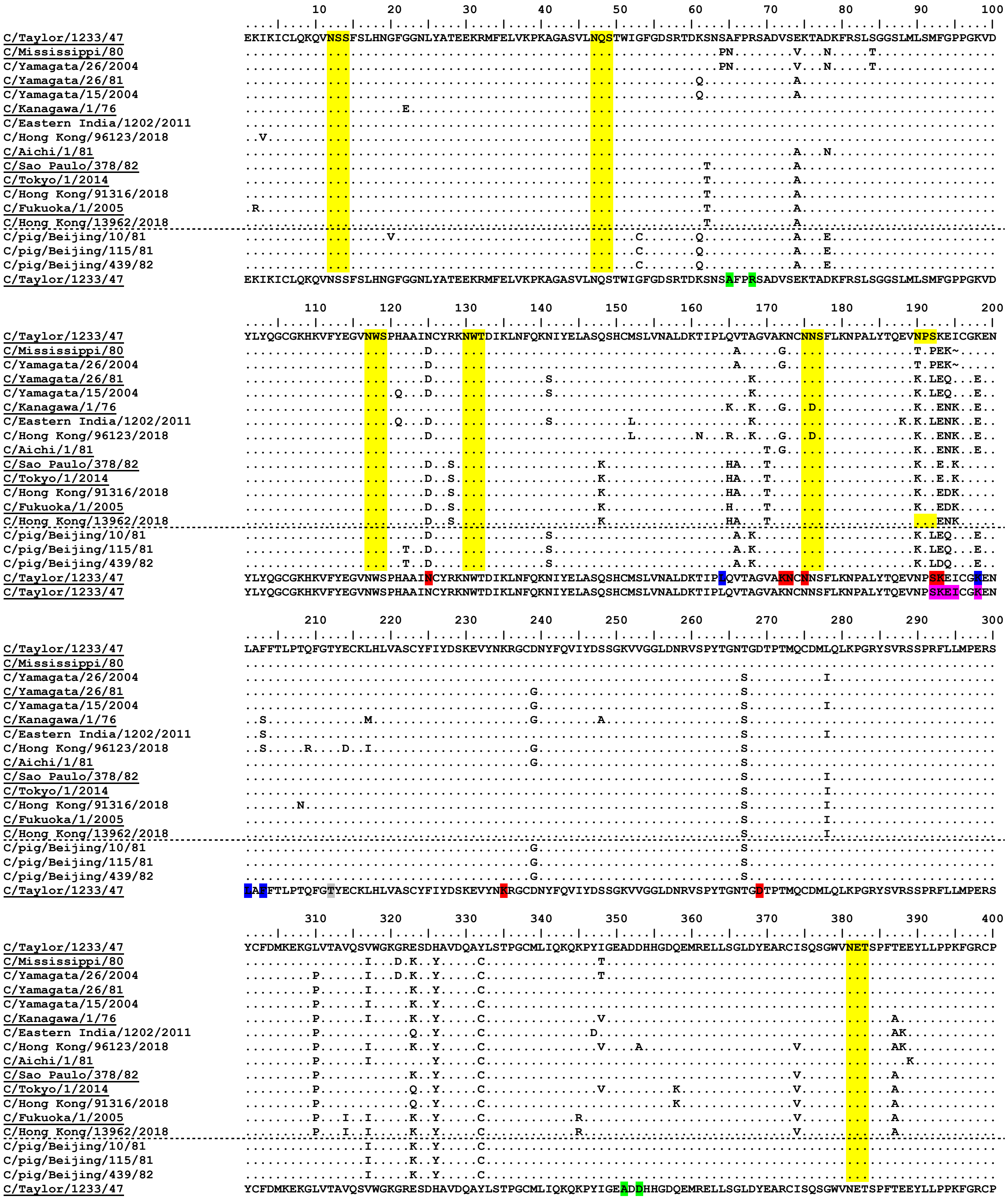

```
                                   410       420       430       440       450       460       470       480       490       500
                           ....|....|....|....|....|....|....|....|....|....|....|....|....|....|....|....|....|....|....|....|
C/Taylor/1233/47           LAAKEESIPKIPDGLLIPTSGTDTIVTKPKSRIFGIDDLIIGLLFVAIVEAGIGGYLLGSRKESGGGVTKESAEKGFEKIGNDIQILRSSTNIAIEKLND
C/Mississippi/80           ........................T...........................................................................
C/Yamagata/26/2004         ........................T...........................................................................
C/Yamagata/26/81           ........................T...........................................................................
C/Yamagata/15/2004         ........................T...........................................................................
C/Kanagawa/1/76            ........................T...................................K..........................K....T.......
C/Eastern India/1202/2011  ........................T...................................................................T.......
C/Hong Kong/96123/2018     ........................T...................................................................T.......
C/Aichi/1/81               ........................T...........................................................................
C/Sao Paulo/378/82         ........................T...........................................................................
C/Tokyo/1/2014             ........................T...........................................................................
C/Hong Kong/91316/2018     ........................T...........................................................................
C/Fukuoka/1/2005           ........................T...........................................................................
C/Hong Kong/13962/2018     ........................T...........................................................................
C/pig/Beijing/10/81        ........................T..................F........................................................
C/pig/Beijing/115/81       ........................T...........................................................................
C/pig/Beijing/439/82       ........................T..................F........................................................
C/Taylor/1233/47           LAAKEESIPKIPDGLLIPTSGTDTIVTKPKSRIFGIDDLIIGLLFVAIVEAGIGGYLLGSRKESGGGVTKESAEKGFEKIGNDIQILRSSTNIAIEKLND

                                   510       520       530       540       550       560       570       580       590       600
                           ....|....|....|....|....|....|....|....|....|....|....|....|....|....|....|....|....|....|....|....|
C/Taylor/1233/47           RITHDEQAIRDLTLEIENARSEALLGELGIIRALLVGNISIGLQESLWELASEITNRAGDLAVEVSPGCWIIDNNICDQSCQNFIFKFNETAPVPTIPPL
C/Mississippi/80           ................................................................I...................................
C/Yamagata/26/2004         ..S.............................................................I...................................
C/Yamagata/26/81           ..S.........................E.......................................................................
C/Yamagata/15/2004         ..S......................................................................D..........................
C/Kanagawa/1/76            ..S...............................................................................D.................
C/Eastern India/1202/2011  ..S...............................................................................D.................
C/Hong Kong/96123/2018     ..S...............................................................................D.................
C/Aichi/1/81               ..S.................................................................................................
C/Sao Paulo/378/82         ..S.................................................................................................
C/Tokyo/1/2014             ..S......................................................................D..........................
C/Hong Kong/91316/2018     ..S......................................................................D..........................
C/Fukuoka/1/2005           ..S.................................................................................................
C/Hong Kong/13962/2018     ..S......................................................................D..........................
C/pig/Beijing/10/81        ..S.................................................................................................
C/pig/Beijing/115/81       ..S.................................................................................................
C/pig/Beijing/439/82       ..S.................................................................................................
C/Taylor/1233/47           RITHDEQAIRDLTLEIENARSEALLGELGIIRALLVGNISIGLQESLWELASEITNRAGDLAVEVSPGCWIIDNNICDQSCQNFIFKFNETAPVPTIPPL

                                   610       620       630       640
                           ....|....|....|....|....|....|....|....|.
C/Taylor/1233/47           DTKIDLQSDPFYWGSSLGLAITTPISLAALVISGIAICRTK
C/Mississippi/80           .........................................
C/Yamagata/26/2004         ......................AA.................
C/Yamagata/26/81           ......................AA.................
C/Yamagata/15/2004         ......................AA.................
C/Kanagawa/1/76            ......................AA.................
C/Eastern India/1202/2011  ..............R.......AA.................
C/Hong Kong/96123/2018     ......................AA.................
C/Aichi/1/81               ......................AA.................
C/Sao Paulo/378/82         ......................AA.................
C/Tokyo/1/2014             ......................AA.................
C/Hong Kong/91316/2018     ......................AA.................
C/Fukuoka/1/2005           ......................AA.................
C/Hong Kong/13962/2018     ......................AA.................
C/pig/Beijing/10/81        .........................................
C/pig/Beijing/115/81       .........................................
C/pig/Beijing/439/82       .........................................
C/Taylor/1233/47           DTKIDLQSDPFYWGSSLGLAITTPISLAALVISGIAICRTK
```

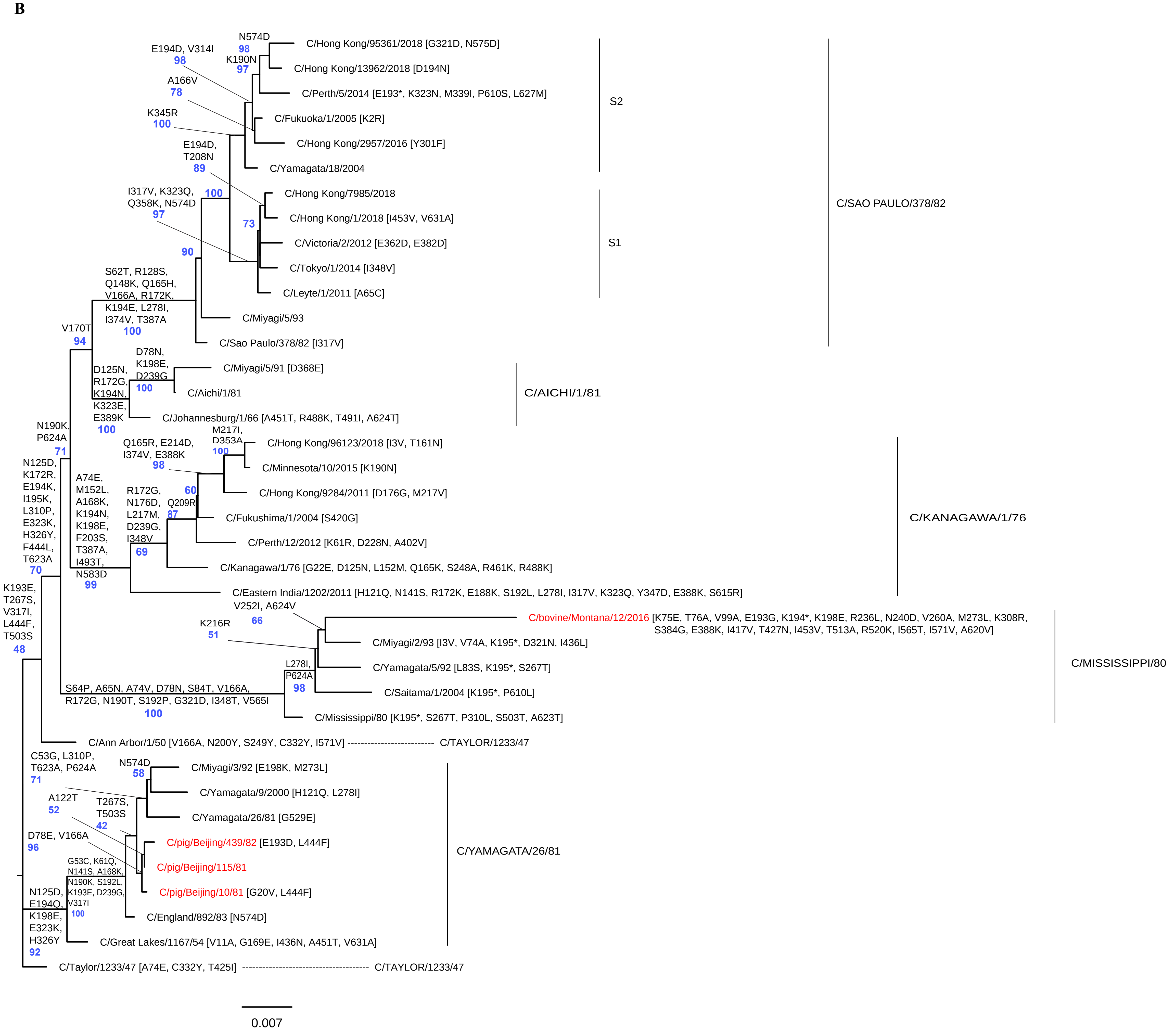
B
N574D
98
C/Hong Kong/95361/2018 [G321D, N575D]
E194D, V314I
98
K190N
97
C/Hong Kong/13962/2018 [D194N]
C/Perth/5/2014 [E193*, K323N, M339I, P610S, L627M]
A166V
78
C/Fukuoka/1/2005 [K2R]
K345R
100
C/Hong Kong/2957/2016 [Y301F]
E194D,
T208N
89
C/Yamagata/18/2004
S2
I317V, K323Q,
Q358K, N574D
97
100
C/Hong Kong/7985/2018
C/Hong Kong/1/2018 [I453V, V631A]
73
C/Victoria/2/2012 [E362D, E382D]
90
C/Tokyo/1/2014 [I348V]
C/Leyte/1/2011 [A65C]
S1
S62T, R128S,
Q148K, Q165H,
V166A, R172K,
K194E, L278I,
I374V, T387A
100
C/Miyagi/5/93
C/Sao Paulo/378/82 [I317V]
C/SAO PAULO/378/82
V170T
94
D78N,
K198E,
D239G
100
C/Miyagi/5/91 [D368E]
D125N,
R172G,
K194N,
K323E,
E389K
100
C/Aichi/1/81
C/Johannesburg/1/66 [A451T, R488K, T491I, A624T]
C/AICHI/1/81
N190K,
P624A
71
M217I,
D353A
100
C/Hong Kong/96123/2018 [I3V, T161N]
Q165R, E214D,
I374V, E388K
98
C/Minnesota/10/2015 [K190N]
C/Hong Kong/9284/2011 [D176G, M217V]
N125D,
K172R,
E194K,
I195K,
L310P,
E323K,
H326Y,
F444L,
T623A
70
A74E,
M152L,
A168K,
K194N,
K198E,
F203S,
T387A,
I493T,
N583D
99
R172G,
N176D,
L217M,
D239G,
I348V
69
60
Q209R
87
C/Fukushima/1/2004 [S420G]
C/Perth/12/2012 [K61R, D228N, A402V]
C/Kanagawa/1/76 [G22E, D125N, L152M, Q165K, S248A, R461K, R488K]
C/Eastern India/1202/2011 [H121Q, N141S, R172K, E188K, S192L, L278I, I317V, K323Q, Y347D, E388K, S615R]
C/KANAGAWA/1/76
V252I, A624V
66
K216R
51
C/bovine/Montana/12/2016 [K75E, T76A, V99A, E193G, K194*, K198E, R236L, N240D, V260A, M273L, K308R, S384G, E388K, I417V, T427N, I453V, T513A, R520K, I565T, I571V, A620V]
C/Miyagi/2/93 [I3V, V74A, K195*, D321N, I436L]
K193E,
T267S,
V317I,
L444F,
T503S
48
L278I,
P624A
98
C/Yamagata/5/92 [L83S, K195*, S267T]
C/Saitama/1/2004 [K195*, P610L]
C/MISSISSIPPI/80
S64P, A65N, A74V, D78N, S84T, V166A,
R172G, N190T, S192P, G321D, I348T, V565I
100
C/Mississippi/80 [K195*, S267T, P310L, S503T, A623T]
C/Ann Arbor/1/50 [V166A, N200Y, S249Y, C332Y, I571V] -------------------------- C/TAYLOR/1233/47
C53G, L310P,
T623A, P624A
71
N574D
58
C/Miyagi/3/92 [E198K, M273L]
C/Yamagata/9/2000 [H121Q, L278I]
A122T
52
T267S,
T503S
42
C/Yamagata/26/81 [G529E]
D78E, V166A
96
C/pig/Beijing/439/82 [E193D, L444F]
C/pig/Beijing/115/81
C/YAMAGATA/26/81
G53C, K61Q,
N141S, A168K,
N190K, S192L,
K193E, D239G,
V317I
100
C/pig/Beijing/10/81 [G20V, L444F]
N125D,
E194Q,
K198E,
E323K,
H326Y
92
C/England/892/83 [N574D]
C/Great Lakes/1167/54 [V11A, G169E, I436N, A451T, V631A]
C/Taylor/1233/47 [A74E, C332Y, T425I] ------------------------------------- C/TAYLOR/1233/47
0.007

C

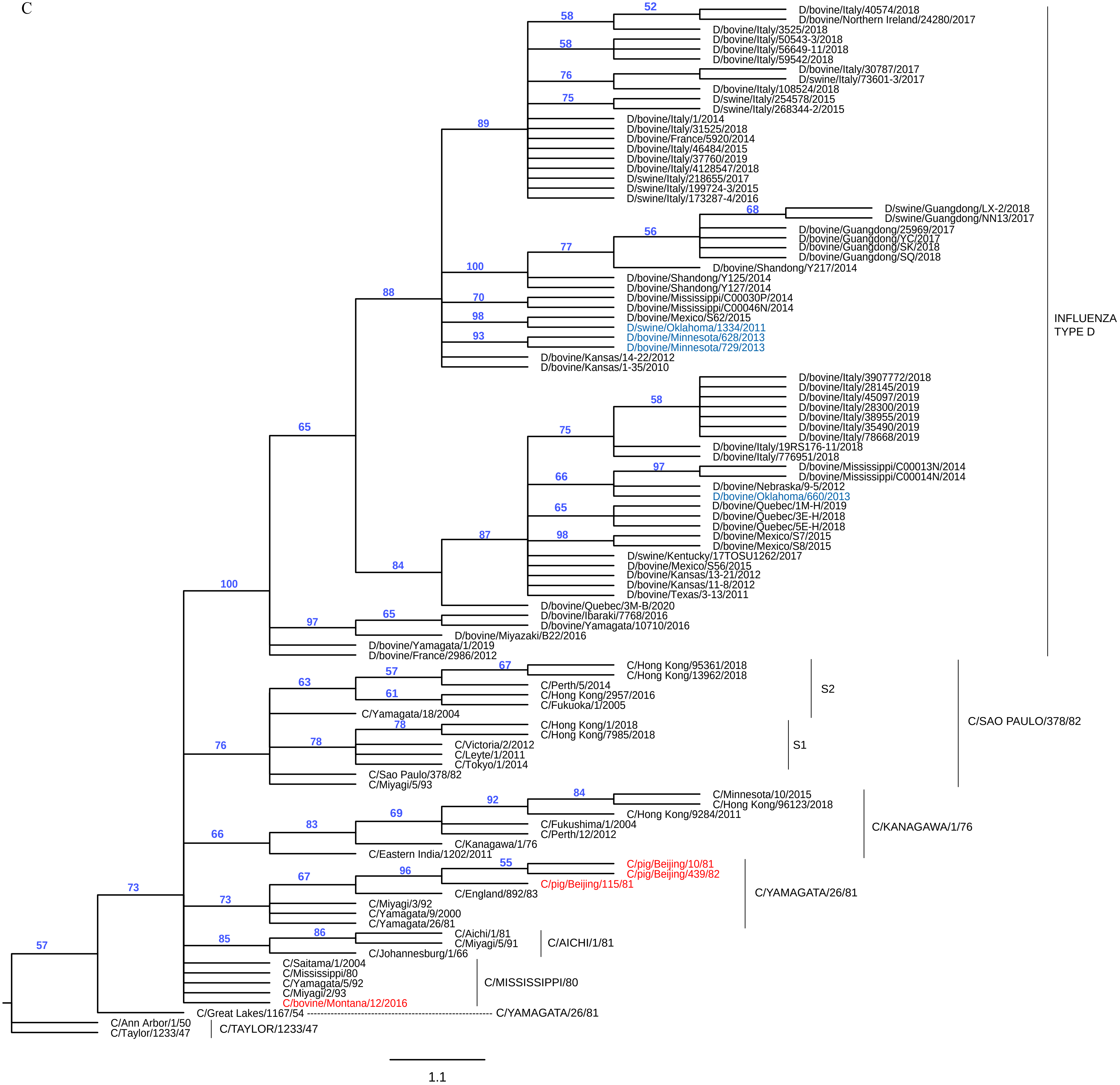
D/bovine/Italy/40574/2018
D/bovine/Northern Ireland/24280/2017
D/bovine/Italy/3525/2018
D/bovine/Italy/50543-3/2018
D/bovine/Italy/56649-11/2018
D/bovine/Italy/59542/2018
D/bovine/Italy/30787/2017
D/swine/Italy/73601-3/2017
D/bovine/Italy/108524/2018
D/swine/Italy/254578/2015
D/swine/Italy/268344-2/2015
D/bovine/Italy/1/2014
D/bovine/Italy/31525/2018
D/bovine/France/5920/2014
D/bovine/Italy/46484/2015
D/bovine/Italy/37760/2019
D/bovine/Italy/4128547/2018
D/swine/Italy/218655/2017
D/swine/Italy/199724-3/2015
D/swine/Italy/173287-4/2016
D/swine/Guangdong/LX-2/2018
D/swine/Guangdong/NN13/2017
D/bovine/Guangdong/25969/2017
D/bovine/Guangdong/YC/2017
D/bovine/Guangdong/SK/2018
D/bovine/Guangdong/SQ/2018
D/bovine/Shandong/Y217/2014
D/bovine/Shandong/Y125/2014
D/bovine/Shandong/Y127/2014
D/bovine/Mississippi/C00030P/2014
D/bovine/Mississippi/C00046N/2014
D/bovine/Mexico/S62/2015
D/swine/Oklahoma/1334/2011
D/bovine/Minnesota/628/2013
D/bovine/Minnesota/729/2013
D/bovine/Kansas/14-22/2012
D/bovine/Kansas/1-35/2010
D/bovine/Italy/3907772/2018
D/bovine/Italy/28145/2019
D/bovine/Italy/45097/2019
D/bovine/Italy/28300/2019
D/bovine/Italy/38955/2019
D/bovine/Italy/35490/2019
D/bovine/Italy/78668/2019
D/bovine/Italy/19RS176-11/2018
D/bovine/Italy/776951/2018
D/bovine/Mississippi/C00013N/2014
D/bovine/Mississippi/C00014N/2014
D/bovine/Nebraska/9-5/2012
D/bovine/Oklahoma/660/2013
D/bovine/Quebec/1M-H/2019
D/bovine/Quebec/3E-H/2018
D/bovine/Quebec/5E-H/2018
D/bovine/Mexico/S7/2015
D/bovine/Mexico/S8/2015
D/swine/Kentucky/17TOSU1262/2017
D/bovine/Mexico/S56/2015
D/bovine/Kansas/13-21/2012
D/bovine/Kansas/11-8/2012
D/bovine/Texas/3-13/2011
D/bovine/Quebec/3M-B/2020
D/bovine/Ibaraki/7768/2016
D/bovine/Yamagata/10710/2016
D/bovine/Miyazaki/B22/2016
D/bovine/Yamagata/1/2019
D/bovine/France/2986/2012
INFLUENZA
TYPE D
C/Hong Kong/95361/2018
C/Hong Kong/13962/2018
C/Perth/5/2014
C/Hong Kong/2957/2016
C/Fukuoka/1/2005
C/Yamagata/18/2004
S2
C/Hong Kong/1/2018
C/Hong Kong/7985/2018
C/Victoria/2/2012
C/Leyte/1/2011
C/Tokyo/1/2014
S1
C/Sao Paulo/378/82
C/Miyagi/5/93
C/SAO PAULO/378/82
C/Minnesota/10/2015
C/Hong Kong/96123/2018
C/Hong Kong/9284/2011
C/Fukushima/1/2004
C/Perth/12/2012
C/Kanagawa/1/76
C/Eastern India/1202/2011
C/KANAGAWA/1/76
C/pig/Beijing/10/81
C/pig/Beijing/439/82
C/pig/Beijing/115/81
C/England/892/83
C/Miyagi/3/92
C/Yamagata/9/2000
C/Yamagata/26/81
C/YAMAGATA/26/81
C/Aichi/1/81
C/Miyagi/5/91
C/Johannesburg/1/66
C/AICHI/1/81
C/Saitama/1/2004
C/Mississippi/80
C/Yamagata/5/92
C/Miyagi/2/93
C/bovine/Montana/12/2016
C/MISSISSIPPI/80
C/Great Lakes/1167/54
C/YAMAGATA/26/81
C/Ann Arbor/1/50
C/Taylor/1233/47
C/TAYLOR/1233/47
1.1

Table S1. ICV-positive samples shared with WHO CC, London that yielded gene sequences.

Virus name	Clinical Sample Collection		Sample Received[a]		rt-RTPCR	Age	Sex	Details on Admission[d]	Co-infecting agent[e]	Complete gene sequences available[f]							GISAID: Isolate Accession No[g]
	Date	Week			C_t value	(years)[b]	F/M[c]			HE	PB2	PB1	P3	NP	MP	NS	
C/Hong Kong/6016/2008			MDCK1	V						Y	Y	Y	Y	Y	Y	Y	EPI_ISL_381036
C/Hong Kong/6483/2009			MDCK4	V						Y	Y	Y	Y	Y	Y	Y	EPI_ISL_380254
C/Hong Kong/9284/2011			LLC3	V						Y		Y	Y	Y	Y	Y	EPI_ISL_380255
C/Hong Kong/1498/2016	2016-01-03	53/2015	NPA	cs	20.03	<1	M	Bronchiolitis	ND	Y	Y	Y	Y	Y	Y	Y	EPI_ISL_380247
C/Hong Kong/1897/2016	2016-01-05	1/2016	NPA	cs	18.56	<1	F	URTI	ND	Y	Y	Y	Y	Y	Y	Y	EPI_ISL_380246
C/Hong Kong/2361/2016	2016-01-05	1/2016	NPA	cs	20.73	4	F	Fever	ND	Y	Y		Y	Y	Y	Y	EPI_ISL_380245
C/Hong Kong/2879/2016	2016-01-06	1/2016	NPS	cs	23.95	89	F	Pneumonia	ND	Y					Y	Y	EPI_ISL_381022
C/Hong Kong/2957/2016	2016-01-07	1/2016	NPA	cs	15.71	1	M	URTI	ND	Y	Y	Y	Y	Y	Y	Y	EPI_ISL_380243
C/Hong Kong/2957/2016	2016-01-07	1/2016	E3(am3)	V		1	M			Y	Y		Y	Y	Y	Y	EPI_ISL_380209
C/Hong Kong/3586/2016	2016-01-08	1/2016	NPA	cs	18.23	13	F	URTI	ND	Y	Y	Y	Y	Y	Y	Y	EPI_ISL_380242
C/Hong Kong/6091/2016	2016-01-14	2/2016	NPS	cs	23.53	58	F	Pneumonia	ND	Y					Y	Y	EPI_ISL_381031
C/Hong Kong/6451/2016	2016-01-14	2/2016	NPA	cs	16.55	2	M	URTI	ND	Y	Y	Y	Y	Y	Y	Y	EPI_ISL_380241
C/Hong Kong/6451/2016	2016-01-14	2/2016	E3(am3)	V		2	M			Y	Y	Y	Y	Y	Y	Y	EPI_ISL_380207
C/Hong Kong/6557/2016	2016-01-14	2/2016	NPS	cs	19.79	43	F	URTI	ND	Y					Y	Y	EPI_ISL_381032
C/Hong Kong/6573/2016	2016-01-14	2/2016	NPA	cs	11.91	14	F	LOC	ND	Y					Y	Y	EPI_ISL_381033
C/Hong Kong/10828/2016	2016-01-22	3/2016	NPS	cs	19.30	<1	F	URTI	ND	Y							EPI_ISL_381013
C/Hong Kong/10431/2016	2016-01-23	3/2016	NPA	cs	25.87	2	F	URTI	ND	Y						Y	EPI_ISL_381012
C/Hong Kong/11106/2016	2016-01-25	4/2016	NPS	cs	17.55	2	F	Cellulitis	ND	Y	Y		Y	Y	Y	Y	EPI_ISL_380240
C/Hong Kong/15639/2016	2016-02-01	5/2016	NPA	cs	16.46	6	F	URTI	ND	Y				Y	Y	Y	EPI_ISL_380239
C/Hong Kong/15888/2016	2016-02-03	5/2016	NPA	cs	23.89	4	M		ND	Y					Y	Y	EPI_ISL_381015
C/Hong Kong/16909/2016	2016-02-04	5/2016	NPA	cs	18.76	3	M	Pneumonia	ND	Y	Y			Y	Y	Y	EPI_ISL_380237
C/Hong Kong/17233/2016	2016-02-05	5/2016	NPS	cs	17.57	2	F		RSV	Y	Y		Y	Y	Y	Y	EPI_ISL_380236
C/Hong Kong/19944/2016	2016-02-08	6/2016	NPA	cs	14.15	5	M	Febrile convulsions	PIV2, Picornavirus	Y				Y	Y	Y	EPI_ISL_380234
C/Hong Kong/19109/2016	2016-02-10	6/2016	NPS	cs	15.11	47	M	Acute pulmonary oedema	ND	Y	Y	Y	Y	Y	Y	Y	EPI_ISL_380232
C/Hong Kong/22986/2016	2016-02-14	6/2016	NPA	cs	24.47	86	F	Heart failure	ND	Y					Y	Y	EPI_ISL_381016
C/Hong Kong/24477/2016	2016-02-15	7/2016	NPS	cs	21.14	86	M		ND	Y					Y	Y	EPI_ISL_381017
C/Hong Kong/24688/2016	2016-02-17	7/2016	NPA	cs	14.73	5	F	URTI	ND	Y	Y	Y	Y	Y	Y	Y	EPI_ISL_380227
C/Hong Kong/27579/2016	2016-02-20	7/2016	NPS	cs	21.80	84	M	URTI	ND	Y	Y	Y	Y			Y	EPI_ISL_381019
C/Hong Kong/27676/2016	2016-02-20	7/2016	NPA	cs	19.73	1	F	URTI	ND	Y	Y	Y	Y	Y	Y	Y	EPI_ISL_380226
C/Hong Kong/28182/2016	2016-02-20	7/2016	NPS	cs	22.19	<1	M	Bronchiolitis	ND	Y						Y	EPI_ISL_381021
C/Hong Kong/3286/2018	2018-01-03	1/2018	NPA	cs	17.77	6	M	URTI	ND	Y				Y	Y	Y	EPI_ISL_380225
C/Hong Kong/3363/2018	2018-01-03	1/2018	NPS	cs	22.49	82	M	COAD	ND	Y	Y	Y	Y	Y	Y	Y	EPI_ISL_380224
C/Hong Kong/7920/2018	2018-01-09	2/2018	NPA	cs	17.88	4	M		ND	Y			Y	Y	Y	Y	EPI_ISL_380223
C/Hong Kong/7663/2018	2018-01-10	2/2018	NPS	cs	23.76	2	M	B-ALL	ND							Y	EPI_ISL_395062
C/Hong Kong/7965/2018	2018-01-10	2/2018	NPA	cs	19.89	53	F	Pneumonia	ND	Y	Y	Y	Y	Y	Y	Y	EPI_ISL_380222
C/Hong Kong/7985/2018	2018-01-10	2/2018	NPA	cs	27.94	1	F		PIV3	Y	Y		Y		Y	Y	EPI_ISL_381034
C/Hong Kong/13962/2018	2018-01-17	3/2018	NPA	cs	12.22	9	M	Pneumonia	ND	Y					Y	Y	EPI_ISL_381014
C/Hong Kong/14027/2018	2018-01-17	3/2018	NPS	cs	20.13	57	F		ND	Y	Y		Y	Y	Y	Y	EPI_ISL_380221
C/Hong Kong/27393/2018	2018-01-29	5/2018	NPA	cs	22.04	4	M	URTI	Adenovirus	Y	Y		Y	Y	Y	Y	EPI_ISL_380220
C/Hong Kong/27395/2018	2018-01-29	5/2018	NPA	cs	17.63	5	M	URTI	ND	Y	Y		Y	Y	Y	Y	EPI_ISL_380219
C/Hong Kong/27542/2018	2018-01-29	5/2018	NPS	cs	24.43	66	M	Abdominal pain	ND	Y						Y	EPI_ISL_381018
C/Hong Kong/27892/2018	2018-01-29	5/2018	NPS	cs	21.22	20	M	Tachycardia	ND	Y	Y		Y	Y	Y	Y	EPI_ISL_380218
C/Hong Kong/27990/2018	2018-01-29	5/2018	NPS	cs	18.68	25	F	Vertigo	ND	Y	Y		Y	Y	Y	Y	EPI_ISL_380217
C/Hong Kong/28015/2018	2018-01-30	5/2018	NPS	cs	24.02	77	M	Fever	HMPV	Y					Y	Y	EPI_ISL_381020
C/Hong Kong/30248/2018	2018-02-01	5/2018	NPS	cs	18.69	4	F	URTI	ND	Y					Y	Y	EPI_ISL_381023
C/Hong Kong/33189/2018	2018-02-04	5/2018	NPA	cs	18.37	78	M	Pneumonia	ND(ICU case)	Y					Y	Y	EPI_ISL_381024
C/Hong Kong/33417/2018	2018-02-04	5/2018	NPA	cs	12.38	2	M	URTI	ND	Y	Y	Y	Y	Y	Y	Y	EPI_ISL_380216
C/Hong Kong/33535/2018	2018-02-04	5/2018	NPS	cs	13.64	38	F		ND	Y	Y	Y	Y	Y	Y	Y	EPI_ISL_380215
C/Hong Kong/37128/2018	2018-02-07	6/2018	NPA	cs	12.79	4	M	URTI	PIV4	Y	Y	Y	Y	Y	Y	Y	EPI_ISL_380213
C/Hong Kong/37128/2018	2018-02-07	6/2018	E3(am3)	V		4	M			Y	Y	Y	Y	Y	Y	Y	EPI_ISL_380244

C/Hong Kong/40626/2018	2018-02-09	6/2018	NPA	cs	15.97	13	F	URTI	ND	Y			Y	Y	Y	Y	EPI_ISL_380212
C/Hong Kong/46081/2018	2018-02-13	7/2018	NPS	cs	18.65	29	M	URTI	ND	Y				Y		Y	EPI_ISL_380211
C/Hong Kong/40232/2018	2018-02-10	6/2018	NPA	cs	21.28	2	F	Febrile convulsions	ND							Y	EPI_ISL_395061
C/Hong Kong/45913/2018	2018-02-14	7/2018	NPS	cs	13.42	<1	M	URTI	ND	Y	Y		Y	Y	Y	Y	EPI_ISL_380210
C/Hong Kong/47455/2018	2018-02-16	7/2018	NPS	cs	21.17	1	M	Febrile convulsions	ND	Y					Y	Y	EPI_ISL_381026
C/Hong Kong/47144/2018	2018-02-17	7/2018	NPA	cs	19.65	2	M	Pneumonia	ND	Y					Y	Y	EPI_ISL_381025
C/Hong Kong/50551/2018	2018-02-18	7/2018	NPS	cs	16.18	41	M		ND	Y						Y	EPI_ISL_381027
C/Hong Kong/50752/2018	2018-02-19	8/2018	NPS	cs	15.76	11	F	URTI	ND	Y						Y	EPI_ISL_381028
C/Hong Kong/58158/2018	2018-02-24	8/2018	NPS	cs	24.51	80	F	R-knee arthritis	ND	Y						Y	EPI_ISL_381030
C/Hong Kong/57069/2018	2018-02-25	8/2018	NPS	cs		32	M	Pneumonia	ND						Y	Y	EPI_ISL_391812
C/Hong Kong/57791/2018	2018-02-26	9/2018	NPS	cs	22.01	85	F	Tachycardia	ND	Y					Y	Y	EPI_ISL_381029
C/Hong Kong/60430/2018	2018-02-28	9/2018	NPA	cs	16.30	1	M	URTI	Rhinovirus	Y	Y	Y	Y	Y	Y	Y	EPI_ISL_380214
C/Hong Kong/60430/2018	2018-02-28	9/2018	E3(am3)	V		1	M			Y	Y	Y	Y	Y	Y	Y	EPI_ISL_380248
C/Hong Kong/91316/2018	2018-04-06	14/2018	NPS	cs	26.11	23	F	Fever	ND	Y	Y			Y	Y	Y	EPI_ISL_380228
C/Hong Kong/94533/2018	2018-04-12	15/2018	NPA	cs	21.83	2	M	URTI	Picornavirus	Y	Y	Y	Y	Y	Y	Y	EPI_ISL_380229
C/Hong Kong/95361/2018	2018-04-14	15/2018	NPS	cs	20.91	1	M		ND	Y	Y		Y	Y	Y	Y	EPI_ISL_380230
C/Hong Kong/95361/2018	2018-04-14	15/2018	E3(am3)	V		1	M			Y	Y	Y	Y	Y	Y	Y	EPI_ISL_380208
C/Hong Kong/96417/2018	2018-04-14	15/2018	NPA	cs	20.70	2	F	Fever	ND	Y	Y	Y	Y	Y	Y	Y	EPI_ISL_380233
C/Hong Kong/96123/2018	2018-04-15	16/2018	TNS	cs	15.89	9	M	Febrile convulsions	ND	Y	Y	Y	Y	Y	Y	Y	EPI_ISL_380231
C/Hong Kong/96123/2018	2018-04-15	16/2018	E3(am3)	V		9	M			Y	Y	Y	Y	Y	Y	Y	EPI_ISL_380238
C/Hong Kong/97303/2018	2018-04-17	16/2018	NPS	cs	29.89	80	F	Pneumonia	ND	Y					Y		EPI_ISL_381035
C/Hong Kong/1/2018	2018-04-29	17/2018	NPA	cs	18.83	4	F		ND	Y	Y	Y	Y	Y	Y	Y	EPI_ISL_380253
C/Hong Kong/1/2018	2018-04-29	17/2018	E3(am3)	V		4	F			Y	Y	Y	Y	Y	Y	Y	EPI_ISL_381011
C/Hong Kong/1/2018	2018-04-29	17/2018	MDCK3	V		4	F			Y	Y	Y	Y	Y	Y	Y	EPI_ISL_380252

[a]V = Virus isolate; cs = clinical specimen; NPA = Nasal/Pharyngeal Aspirate; NPS = Nasal/Pharyngeal Swab;TNS = Throat and Nasal Swab; Others (MDCK1/3/4, LLC3) = passage history in cell culture. [b]Age given in years. [c]Sex, M = Male, F = Female. [d]B-ALL = B-cell Acute Lymphocitic Leukaemia, COAD = Chronic Obstructive Airways Disease, LOC = Loss of Consciousness, URTI - Upper Respiratory Tract Infection. [e]PIV = Parainfluenza virus (types 2, 3, 4); RSV = Respiratory syncytial virus; HMPV = Human metapneumovirus; ND = None Detected (of those that were screened for). [f]For each sample the recovery of at least one gene sequence encompassing complete open reading frame(s) is indicated (Y). [g]Corresponding EpiFlu database (GISAID) isolate accession numbers are given. Viruses isolated in the amniotic cavity of embryonated hens' eggs during the course of this study, two from the 2015-16 outbreak and five from the 2017-18, are highlighted.

Table S2. Minority variants of the HE genes of ICVs from Hong Kong sequenced for this study.

Virus name/passage history[a]	Clade[b]	Nucleotide number[c]	Total Reads	Concensus nucleotide[d]	Alternate nucleotide[d]	Supporting Reads	Frequency of alternate (%)	HEF amino acid position[e]	Codon position	S/NS[f]	Amino acid substitution[g]
C/Hong Kong/6016/2008 MDCK1	K	556	5286	G	A	159	3.01	172	1	NS	G - R
C/Hong Kong/6016/2008 MDCK1		827	5396	C	A	140	2.59	262	2	NS	P - H
C/Hong Kong/6016/2008 MDCK1		1397	6672	G	A	354	5.30	452	2	NS	G - E
C/Hong Kong/6483/2009 MDCK4	K	373	2654	GT	G	28	1.05	111		1 del	FS
C/Hong Kong/6483/2009 MDCK4		733	1774	G	A	42	2.37	231	1	NS	E - K
C/Hong Kong/6483/2009 MDCK4		1388	2376	T	C	34	1.43	449	2	NS	V - A
C/Hong Kong/6483/2009 MDCK4		1389	2477	T	A	36	1.45	449	3	S	V/A
C/Hong Kong/6483/2009 MDCK4		1397	3051	G	A	123	4.03	452	2	NS	G - E
C/Hong Kong/9284/2011 LLC3	K	43	4671	G	GA	52	1.1	1		1 ins	FS
C/Hong Kong/9284/2011 LLC3		180	5773	G	A	72	1.25	46	3	S	L
C/Hong Kong/9284/2011 LLC3		1388	317	T	C	13	4.10	449	2	NS	V - A
C/Hong Kong/9284/2011 LLC3		1389	370	T	A	18	4.86	449	3	S	V/A
C/Hong Kong/9284/2011 LLC3		1397	767	G	A	50	6.51	452	2	NS	G - E
C/Hong Kong/9284/2011 LLC3		1432	1529	T	G	38	2.48	464	1	NS	S - A
C/Hong Kong/9284/2011 LLC3		1606	3670	G	A	119	3.24	522	1	NS	E - K
C/Hong Kong/1498/2016 cs	S1	373	956	GT	G	15	1.57	111		1 del	FS
C/Hong Kong/1498/2016 cs		547	996	G	A	11	1.10	169	1	NS	G - R
C/Hong Kong/1498/2016 cs		649	1164	T	C	12	1.03	203	1	NS	F - L
C/Hong Kong/1498/2016 cs		810	1047	A	G	11	1.05	256	3	S	L
C/Hong Kong/1498/2016 cs		1388	1024	T	C	54	5.27	449	2	NS	V - A
C/Hong Kong/1498/2016 cs		1397	2138	G	A	111	5.19	452	2	NS	G - E
C/Hong Kong/1498/2016 cs		1432	3912	T	G	67	1.71	464	1	NS	S - A
C/Hong Kong/1498/2016 cs		1641	6167	C	T	88	1.43	533	3	S	A
C/Hong Kong/1498/2016 cs		1718	6791	G	A	144	2.12	559	2	NS	G - E
C/Hong Kong/1498/2016 cs		1719	6786	A	G	916	13.49	559	3	S	G/E
C/Hong Kong/1498/2016 cs		1730	7015	T	C	82	1.17	563	2	NS	V - A
C/Hong Kong/1897/2016 cs	S2	153	1870	C	T	25	1.34	37	3	S	V
C/Hong Kong/1897/2016 cs		1388	2443	T	C	85	3.48	449	2	NS	V - A
C/Hong Kong/1897/2016 cs		1389	2705	T	A	96	3.54	449	3	S	V/A
C/Hong Kong/1897/2016 cs		1397	3846	G	A	238	6.19	452	2	NS	G - E
C/Hong Kong/1897/2016 cs		1461	6445	T	A	79	1.22	473	3	S	A
C/Hong Kong/1897/2016 cs		1718	13739	G	A	192	1.40	559	2	NS	G - E
C/Hong Kong/1897/2016 cs		1719	14754	A	G	1077	7.29	559	3	S	G/E
C/Hong Kong/2361/2016 cs	S1	236	281	C	A	24	8.51	65	2	NS	A - D
C/Hong Kong/2361/2016 cs		276	325	TAAATTCCG	T	35	10.77	78		8 del	FS
C/Hong Kong/2361/2016 cs		1388	256	T	C	29	11.33	449	2	NS	V - A
C/Hong Kong/2361/2016 cs		1397	1051	G	A	135	12.84	452	2	NS	G - E
C/Hong Kong/2361/2016 cs		1432	2105	T	G	35	1.66	464	1	NS	S - A
C/Hong Kong/2361/2016 cs		1468	2041	G	A	24	1.18	476	1	NS	G - R
C/Hong Kong/2361/2016 cs		1632	3490	A	G	59	1.69	530	3	NS	I - M
C/Hong Kong/2361/2016 cs		1636	3821	A	G	43	1.13	532	1	NS	R - G
C/Hong Kong/2361/2016 cs		1701	3639	A	C	59	1.62	553	3	NS	E - D
C/Hong Kong/2361/2016 cs		1703	3251	T	G	216	6.63	554	2	NS	I - R
C/Hong Kong/2361/2016 cs		1719	12398	A	G	467	3.76	559	3	S	G
C/Hong Kong/2879/2016 cs	S1	102	310	C	T	13	4.19	20	3	S	G
C/Hong Kong/2879/2016 cs		1399	57	A	G	11	19.30	453	1	NS	I - V
C/Hong Kong/2957/2016 cs	S2	1388	2203	T	C	72	3.27	449	2	NS	V - A
C/Hong Kong/2957/2016 cs		1389	2348	T	A	87	3.69	449	3	S	V/A
C/Hong Kong/2957/2016 cs		1397	3570	G	A	258	7.22	452	2	NS	G - E

C/Hong Kong/2957/2016 cs		1461	5714	T	A	62	1.08	473	3	S	A
C/Hong Kong/2957/2016 cs		1718	11337	G	A	183	1.61	559	2	NS	G - E
C/Hong Kong/2957/2016 cs		1719	11915	A	G	1090	9.14	559	3	S	G/E
C/Hong Kong/2957/2016 E3(am3)		244	3233	A	G	41	1.27	68	1	NS	R - X
C/Hong Kong/2957/2016 E3(am3)		245	3182	G	C	35	1.10	68	2	NS	R - X
C/Hong Kong/2957/2016 E3(am3)		246	3267	G	T	39	1.19	68	3	NS	R - X
C/Hong Kong/2957/2016 E3(am3)		714	2529	T	C	33	1.30	224	3	S	Y
C/Hong Kong/2957/2016 E3(am3)		716	2525	T	G	27	1.07	225	2	NS	F - C
C/Hong Kong/2957/2016 E3(am3)		1396	2600	G	A	64	2.46	452	1	NS	G - R
C/Hong Kong/2957/2016 E3(am3)		1397	2597	G	A	44	1.69	452	2	NS	G - E/K
C/Hong Kong/2957/2016 E3(am3)		1398	3008	A	G	302	10.03	452	3	S	G/R/E/K
C/Hong Kong/2957/2016 E3(am3)		1399	3033	A	G	506	16.68	453	1	NS	I - V
C/Hong Kong/2957/2016 E3(am3)		1717	2708	G	A	31	1.14	559	1	NS	G - R
C/Hong Kong/3586/2016 cs	S1	425	1639	G	A	18	1.10	128	2	NS	S - N
C/Hong Kong/3586/2016 cs		426	1645	T	A	72	4.23	128	3	NS	S - R
C/Hong Kong/3586/2016 cs		994	1694	TG	T	23	1.35	318		1 del	FS
C/Hong Kong/3586/2016 cs		1113	1711	T	C	20	1.17	357	3	S	D
C/Hong Kong/3586/2016 cs		1140	1636	A	G	26	1.59	366	3	S	G
C/Hong Kong/3586/2016 cs		1388	1593	T	C	68	4.27	449	2	NS	V - A
C/Hong Kong/3586/2016 cs		1389	1737	T	A	80	4.60	449	3	S	V/A
C/Hong Kong/3586/2016 cs		1397	2846	G	A	145	5.09	452	2	NS	G - E
C/Hong Kong/3586/2016 cs		1505	5379	G	A	60	1.12	488	2	NS	R - K
C/Hong Kong/3586/2016 cs		1718	9108	G	A	202	2.22	559	2	NS	G - E
C/Hong Kong/3586/2016 cs		1719	9387	A	G	1393	14.82	559	3	S	G/E
C/Hong Kong/6091/2016 cs	S2	1397	342	G	A	18	5.25	452	2	NS	G - E/K
C/Hong Kong/6091/2016 cs		1398	345	G	A	47	13.62	452	3	S	G/E/K
C/Hong Kong/6091/2016 cs		1399	347	G	A	19	5.48	453	1	NS	V - I
C/Hong Kong/6091/2016 cs		1408	356	T	C	13	3.65	456	1	NS	Y - H
C/Hong Kong/6091/2016 cs		1570	525	A	G	27	5.14	510	1	NS	R - G
C/Hong Kong/6091/2016 cs		1833	561	T	C	10	1.78	597	3	S	I
C/Hong Kong/6091/2016 cs		1866	552	A	G	11	1.99	608	3	S	S
C/Hong Kong/6451/2016 cs	S1	373	2251	GT	G	25	1.11	111		1 del	FS
C/Hong Kong/6451/2016 cs		994	2557	TG	T	29	1.13	318		1 del	FS
C/Hong Kong/6451/2016 cs		1389	2477	T	A	100	4.03	449	3	S	V
C/Hong Kong/6451/2016 cs		1397	3744	G	A	250	6.68	452	2	NS	G - E
C/Hong Kong/6451/2016 cs		1718	12090	G	A	171	1.41	559	2	NS	G - E
C/Hong Kong/6451/2016 cs		1719	12990	A	G	924	7.11	559	3	S	G/E
C/Hong Kong/6451/2016 E3(am3)		1396	4832	G	A	53	1.10	452	1	NS	G - R
C/Hong Kong/6451/2016 E3(am3)		1397	4759	G	A	61	1.28	452	2	NS	G - E/K
C/Hong Kong/6451/2016 E3(am3)		1398	5033	A	G	264	5.24	452	3	S	G/R/E/K
C/Hong Kong/6451/2016 E3(am3)		1399	5175	A	G	696	13.45	453	1	NS	I - V
C/Hong Kong/6557/2016 cs	S2	231	135	T	A	18	13.33	63	3	NS	N - K
C/Hong Kong/6557/2016 cs		232	129	T	G	18	13.95	64	1	NS	S - A
C/Hong Kong/6557/2016 cs		1397	631	G	A	81	12.84	452	2	NS	G - E/K
C/Hong Kong/6557/2016 cs		1398	636	G	A	79	12.42	452	3	S	G/E/K
C/Hong Kong/6557/2016 cs		1432	974	T	G	12	1.23	464	1	NS	S - A
C/Hong Kong/6557/2016 cs		1711	1259	A	G	17	1.35	557	1	NS	R - G
C/Hong Kong/6557/2016 cs		1712	1243	G	A	19	1.53	557	2	NS	R - K/Q
C/Hong Kong/6557/2016 cs		1718	2993	G	A	130	4.34	559	2	NS	G - E
C/Hong Kong/6557/2016 cs		1721	3036	A	G	48	1.58	560	2	NS	D - G
C/Hong Kong/6573/2016 cs	S1	1397	477	G	A	46	9.64	452	2	NS	G - E
C/Hong Kong/6573/2016 cs		1426	868	A	G	16	1.84	462	1	NS	K - E

C/Hong Kong/6573/2016 cs		1427	872	A	C	15	1.72	462	2	NS	K - T/A
C/Hong Kong/6573/2016 cs		1432	730	T	G	68	9.30	464	1	NS	S - A
C/Hong Kong/6573/2016 cs		1437	785	A	G	18	2.29	465	3	S	G
C/Hong Kong/6573/2016 cs		1718	2065	G	A	125	6.05	559	2	NS	G - E
C/Hong Kong/6573/2016 cs		1721	2063	A	G	27	1.31	560	2	NS	D - G
C/Hong Kong/11106/2016 cs	S1	1397	322	G	A	27	8.39	452	2	NS	G - E
C/Hong Kong/11106/2016 cs		1432	457	T	G	11	2.40	464	1	NS	S - A
C/Hong Kong/11106/2016 cs		1719	489	A	G	23	4.69	559	3	S	G
C/Hong Kong/15639/2016 cs	S1	857	416	C	T	34	8.17	272	2	NS	T - I
C/Hong Kong/15639/2016 cs		1388	410	T	C	33	8.05	449	2	NS	V - A
C/Hong Kong/15639/2016 cs		1389	460	T	A	38	8.26	449	3	S	V/A
C/Hong Kong/15639/2016 cs		1397	1326	G	A	97	7.31	452	2	NS	G - E
C/Hong Kong/15639/2016 cs		1432	2171	T	G	33	1.52	464	1	NS	S - A
C/Hong Kong/15639/2016 cs		1718	3973	G	A	110	2.77	559	2	NS	G - E
C/Hong Kong/16909/2016 cs	S1	373	517	GT	G	10	1.93	111		1 del	FS
C/Hong Kong/16909/2016 cs		1388	681	T	C	102	14.98	449	2	NS	V - A
C/Hong Kong/16909/2016 cs		1389	727	T	A	117	16.09	449	3	S	V/A
C/Hong Kong/16909/2016 cs		1397	2335	G	A	273	11.68	452	2	NS	G - E
C/Hong Kong/16909/2016 cs		1432	4582	T	G	92	2.00	464	1	NS	S - A
C/Hong Kong/16909/2016 cs		1718	10283	G	A	233	2.27	559	2	NS	G - E
C/Hong Kong/16909/2016 cs		1719	10757	A	G	1706	15.85	559	3	S	G/E
C/Hong Kong/17233/2016 cs	S1	178	986	T	C	11	1.12	46	1	S	L
C/Hong Kong/17233/2016 cs		1388	525	T	C	38	7.24	449	2	NS	V - A
C/Hong Kong/17233/2016 cs		1389	561	T	A	39	6.94	449	3	S	V/A
C/Hong Kong/17233/2016 cs		1397	1474	G	A	90	6.11	452	2	NS	G - E
C/Hong Kong/17233/2016 cs		1432	2620	T	G	33	1.26	464	1	NS	S - A
C/Hong Kong/17233/2016 cs		1719	4096	A	G	191	4.66	559	3	S	G
C/Hong Kong/19944/2016 cs	S1	1397	1631	G	A	194	11.89	452	2	NS	G - E
C/Hong Kong/19944/2016 cs		1432	3136	T	G	57	1.81	464	1	NS	S - A
C/Hong Kong/19944/2016 cs		1718	7402	G	A	224	3.03	559	2	NS	G - E
C/Hong Kong/19109/2016 cs	S1	267	3357	G	A	96	2.86	75	3	S	K
C/Hong Kong/19109/2016 cs		269	3237	C	T	81	2.50	76	2	NS	T - I
C/Hong Kong/19109/2016 cs		272	3114	C	A	49	1.57	77	2	NS	A - D
C/Hong Kong/19109/2016 cs		273	3072	T	C	38	1.24	77	3	S	A/D
C/Hong Kong/19109/2016 cs		1388	3015	T	C	77	2.55	449	2	NS	V - A
C/Hong Kong/19109/2016 cs		1389	3046	T	A	85	2.79	449	3	S	V/A
C/Hong Kong/19109/2016 cs		1397	4199	G	A	234	5.57	452	2	NS	G - E
C/Hong Kong/19109/2016 cs		1718	11463	G	A	156	1.36	559	2	NS	G - E
C/Hong Kong/19109/2016 cs		1719	12307	A	G	726	5.90	559	3	S	G/E
C/Hong Kong/22986/2016 cs	S1	1719	219	A	G	43	19.63	559	3	S	G
C/Hong Kong/24477/2016 cs	K	1397	335	G	A	11	3.28	452	2	NS	G - E
C/Hong Kong/24477/2016 cs		1461	493	T	A	23	4.63	473	3	S	A
C/Hong Kong/24477/2016 cs		1464	530	A	AG	23	4.33	474		1 ins	FS
C/Hong Kong/24477/2016 cs		1465	505	A	G	13	2.51	475	1	NS	K - E
C/Hong Kong/24477/2016 cs		1466	517	A	C	12	2.32	475	2	NS	K - T/A
C/Hong Kong/24477/2016 cs		1467	524	A	G	14	2.67	475	3	NS	K/E/Q/T/A/P
C/Hong Kong/24477/2016 cs		1471	751	T	G	12	1.59	477	1	NS	F - V
C/Hong Kong/24477/2016 cs		1545	958	A	G	10	1.04	501	3	S	R
C/Hong Kong/24477/2016 cs		1664	1149	T	G	32	2.79	541	2	NS	I - R
C/Hong Kong/24477/2016 cs		1665	1161	A	G	29	2.50	541	3	ns	R/M
C/Hong Kong/24477/2016 cs		1719	1024	A	G	42	4.10	559	3	S	G
C/Hong Kong/24477/2016 cs		1740	894	C	T	11	1.23	566	3	S	S

C/Hong Kong/24477/2016 cs		1764	1110	C	T	13	1.17	574	3	S	N
C/Hong Kong/24477/2016 cs		1789	1150	G	A	12	1.04	583	1	NS	D - N
C/Hong Kong/24477/2016 cs		1803	1181	A	G	12	1.02	587	3	S	K
C/Hong Kong/24477/2016 cs		1836	1045	C	T	13	1.24	598	3	S	P
C/Hong Kong/24477/2016 cs		1875	1055	C	T	13	1.23	611	3	S	F
C/Hong Kong/24688/2016 cs	K	373	5992	GT	G	69	1.15	111		1 del	FS
C/Hong Kong/24688/2016 cs		1397	7829	G	A	216	2.76	452	2	NS	G - E
C/Hong Kong/24688/2016 cs		1432	12301	T	G	135	1.10	464	1	NS	S - A
C/Hong Kong/24688/2016 cs		1461	12327	T	A	222	1.80	473	3	S	A
C/Hong Kong/24688/2016 cs		1467	14013	A	G	148	1.06	475	3	S	K
C/Hong Kong/24688/2016 cs		1469	12704	G	GA	143	1.12	476		1 ins	FS
C/Hong Kong/24688/2016 cs		1519	15711	A	G	1081	6.88	493	1	NS	T - V
C/Hong Kong/27579/2016 cs	K	373	3493	GT	G	41	1.17	111		1 del	FS
C/Hong Kong/27579/2016 cs		1389	4556	T	A	90	1.97	449	3	S	V
C/Hong Kong/27579/2016 cs		1397	5602	G	A	246	4.39	452	2	NS	G - E
C/Hong Kong/27579/2016 cs		1432	8709	T	G	225	2.58	464	1	NS	S - A
C/Hong Kong/27579/2016 cs		1454	8538	A	G	97	1.14	471	2	NS	E - G
C/Hong Kong/27579/2016 cs		1461	8519	T	A	215	2.52	473	3	S	A
C/Hong Kong/27579/2016 cs		1464	11050	A	AG	140	1.26	474		1 ins	FS
C/Hong Kong/27579/2016 cs		1465	10855	A	G	144	1.32	475	1	NS	K - E
C/Hong Kong/27579/2016 cs		1466	10971	A	C	122	1.11	475	2	NS	K - T/A
C/Hong Kong/27579/2016 cs		1467	10575	A	G	129	1.22	475	3	NS	K/E/Q/T/A/P
C/Hong Kong/27579/2016 cs		1468	9865	G	GA	99	1	476		1 ins	FS
C/Hong Kong/27579/2016 cs		1469	10114	G	GA	106	1.05	476		1 ins	FS
C/Hong Kong/27579/2016 cs		1719	17601	A	G	363	2.06	559	3	S	G
C/Hong Kong/27676/2016 cs	S1	554	1573	CA	C	19	1.21	171		1 del	FS
C/Hong Kong/27676/2016 cs		599	1528	CACAAGAAGT	C	20	1.3	186		9 ins	3 aa ins
C/Hong Kong/27676/2016 cs		1319	1519	T	C	35	2.30	426	2	NS	V- A
C/Hong Kong/27676/2016 cs		1388	1604	T	C	80	4.99	449	2	NS	V - A
C/Hong Kong/27676/2016 cs		1389	1741	T	A	88	5.05	449	3	S	V/A
C/Hong Kong/27676/2016 cs		1397	3022	G	A	164	5.43	452	2	NS	G - E/K
C/Hong Kong/27676/2016 cs		1718	9740	G	A	120	1.23	559	2	NS	G - E
C/Hong Kong/27676/2016 cs		1719	9951	A	G	826	8.29	559	3	S	G/E
C/Hong Kong/28182/2016 cs	S1	1683	85	G	A	10	11.76	547	3	S	L
C/Hong Kong/28182/2016 cs		1710	79	T	C	12	15.19	556	3	S	N
C/Hong Kong/28182/2016 cs		1713	87	A	G	15	17.24	557	3	S	R
C/Hong Kong/28182/2016 cs		1728	85	G	A	12	14.12	562	3	S	A
C/Hong Kong/28182/2016 cs		1740	82	T	C	12	14.63	566	3	S	S
C/Hong Kong/28182/2016 cs		1762	102	G	A	17	16.67	574	1	NS	D - N
C/Hong Kong/28182/2016 cs		1764	105	T	C	14	13.33	574	3	S	D/N
C/Hong Kong/28182/2016 cs		1789	110	A	G	15	13.64	583	1	NS	N - D
C/Hong Kong/28182/2016 cs		1803	108	G	A	16	14.81	587	3	S	K
C/Hong Kong/28182/2016 cs		1836	108	T	C	13	11.93	598	3	S	P
C/Hong Kong/28182/2016 cs		1875	117	T	C	12	10.26	611	3	S	F
C/Hong Kong/28182/2016 cs		1901	107	T	C	18	16.82	620	2	NS	V - A
C/Hong Kong/3286/2018 cs	S1	54	333	GAT	G	48	14.41	4		2 del	FS
C/Hong Kong/3286/2018 cs		109	295	G	T	14	4.73	23	1	NS	G - L*
C/Hong Kong/3286/2018 cs		110	313	G	A	15	4.79	23	2	NS	G - Q*
C/Hong Kong/3286/2018 cs		112	315	A	C	13	4.11	24	1	NS	N - H
C/Hong Kong/3286/2018 cs		161	290	A	G	14	4.83	40	2	NS	K - R
C/Hong Kong/3286/2018 cs		192	283	ATGGAT	A	19	6.67	50		5 del	FS
C/Hong Kong/3286/2018 cs		1388	185	T	C	22	11.89	449	2	NS	V - A

C/Hong Kong/3286/2018 cs		1389	197	T	A	28	14.21	449	3	S	V/A
C/Hong Kong/3286/2018 cs		1397	1031	G	A	72	6.98	452	2	NS	G - E
C/Hong Kong/3286/2018 cs		1718	2767	G	A	64	2.31	559	2	NS	G - E
C/Hong Kong/3286/2018 cs		1719	2751	A	G	458	16.64	559	3	S	G/E
C/Hong Kong/3286/2018 cs		1795	3513	AT	A	53	1.51	585		1 del	FS
C/Hong Kong/3363/2018 cs	S1	87	674	C	T	25	3.71	15	3	S	F
C/Hong Kong/3363/2018 cs		373	723	GT	G	11	1.52	111		1 del	FS
C/Hong Kong/3363/2018 cs		1221	774	T	C	10	1.29	393	3	S	P
C/Hong Kong/3363/2018 cs		1388	823	T	C	50	6.06	449	2	NS	V - A
C/Hong Kong/3363/2018 cs		1389	924	T	A	64	6.90	449	3	S	V/A
C/Hong Kong/3363/2018 cs		1397	2097	G	A	119	5.67	452	2	NS	G - E
C/Hong Kong/3363/2018 cs		1489	4228	G	A	51	1.21	483	1	NS	D - N
C/Hong Kong/3363/2018 cs		1718	7360	G	A	106	1.44	559	2	NS	G - E
C/Hong Kong/3363/2018 cs		1719	7439	A	G	1129	15.17	559	3	S	G/E
C/Hong Kong/3363/2018 cs		1913	5539	C	A	64	1.15	624	2	NS	A - D
C/Hong Kong/7920/2018 cs	S2	223	288	A	C	11	3.81	61	1	NS	K - Q
C/Hong Kong/7920/2018 cs		1388	287	T	C	36	12.54	449	2	NS	V - A
C/Hong Kong/7920/2018 cs		1389	343	T	A	48	13.99	449	3	S	V/A
C/Hong Kong/7920/2018 cs		1397	1321	G	A	140	10.60	452	2	NS	G - E
C/Hong Kong/7920/2018 cs		1432	2457	T	G	88	3.57	464	1	NS	S - A
C/Hong Kong/7920/2018 cs		1437	2173	A	G	22	1.01	465	3	S	G
C/Hong Kong/7920/2018 cs		1461	2262	T	A	29	1.28	473	3	S	A
C/Hong Kong/7920/2018 cs		1467	2424	A	G	25	1.03	475	3	S	K
C/Hong Kong/7920/2018 cs		1718	4800	G	A	99	2.06	559	2	NS	G - E
C/Hong Kong/7920/2018 cs		1719	4959	A	G	679	13.69	559	3	S	G/E
C/Hong Kong/7965/2018 cs	S1	488	2241	C	A	40	1.78	149	2	NS	S - *
C/Hong Kong/7965/2018 cs		1388	2356	T	C	112	4.75	449	2	NS	V - A
C/Hong Kong/7965/2018 cs		1389	2410	T	A	129	5.35	449	3	S	V/A
C/Hong Kong/7965/2018 cs		1397	4072	G	A	245	6.01	452	2	NS	G - E/K
C/Hong Kong/7965/2018 cs		1407	4912	C	T	173	3.52	455	3	S	G
C/Hong Kong/7965/2018 cs		1432	7694	T	G	119	1.54	464	1	NS	S - A
C/Hong Kong/7965/2018 cs		1718	14101	G	A	204	1.45	559	2	NS	G - E
C/Hong Kong/7965/2018 cs		1719	15231	A	G	1360	8.92	559	3	S	G/E
C/Hong Kong/7985/2018 cs	S1	150	149	T	C	11	7.38	36	3	S	L
C/Hong Kong/7985/2018 cs		252	165	T	C	15	9.09	70	3	S	A
C/Hong Kong/7985/2018 cs		490	177	C	T	15	8.47	150	1	NS	H - Y
C/Hong Kong/7985/2018 cs		1397	506	G	A	22	4.34	452	2	NS	G - E
C/Hong Kong/7985/2018 cs		1432	701	T	G	11	1.56	464	1	NS	S - A
C/Hong Kong/7985/2018 cs		1582	1158	T	C	19	1.64	514	1	S	L
C/Hong Kong/7985/2018 cs		1651	1184	G	A	17	1.44	537	1	NS	G - R
C/Hong Kong/7985/2018 cs		1716	893	A	G	21	2.35	558	3	S	A
C/Hong Kong/7985/2018 cs		1719	872	A	G	13	1.49	559	3	S	G
C/Hong Kong/7985/2018 cs		1776	1260	T	A	37	2.92	578	3	NS	D - E
C/Hong Kong/13962/2018 cs	S2	1397	1318	G	A	164	12.42	452	2	NS	G - E
C/Hong Kong/13962/2018 cs		1432	2168	T	G	69	3.18	464	1	NS	S - A
C/Hong Kong/13962/2018 cs		1437	2021	A	G	32	1.58	465	3	S	G
C/Hong Kong/13962/2018 cs		1461	2168	T	A	66	3.04	473	3	S	A
C/Hong Kong/13962/2018 cs		1465	2253	A	G	48	2.10	475	1	NS	K - E
C/Hong Kong/13962/2018 cs		1466	2269	A	C	42	1.85	475	2	NS	K - T/A
C/Hong Kong/13962/2018 cs		1467	2257	A	G	40	1.77	475	3	NS	K/E/Q/T/A/P
C/Hong Kong/13962/2018 cs		1581	3988	C	T	44	1.10	513	3	S	T
C/Hong Kong/13962/2018 cs		1712	4249	G	A	51	1.20	557	2	NS	R - K

C/Hong Kong/13962/2018 cs		1718	6118	G	A	173	2.83	559	2	NS	G - E
C/Hong Kong/13962/2018 cs		1464	2294	A	AG	48	2.09	474		1 ins	FS
C/Hong Kong/13962/2018 cs		1468	2546	G	GA	28	1.1	476		1 ins	FS
C/Hong Kong/13962/2018 cs		1469	2584	G	GA	33	1.28	476		1 ins	FS
C/Hong Kong/14027/2018 cs	K	1388	419	T	C	26	6.19	449	2	NS	V - A
C/Hong Kong/14027/2018 cs		1389	462	T	A	32	6.91	449	3	S	V/A
C/Hong Kong/14027/2018 cs		1397	1533	G	A	67	4.37	452	2	NS	G - E
C/Hong Kong/14027/2018 cs		1432	2490	T	G	54	2.17	464	1	NS	S - A
C/Hong Kong/14027/2018 cs		1437	2099	A	G	54	2.57	465	3	S	G
C/Hong Kong/14027/2018 cs		1454	2317	A	G	27	1.16	471	2	NS	E - G
C/Hong Kong/14027/2018 cs		1461	2394	T	A	87	3.62	473	3	S	A
C/Hong Kong/14027/2018 cs		1464	3062	A	AG	91	2.96	474		1 ins	FS
C/Hong Kong/14027/2018 cs		1465	2939	A	C	45	1.51	475	1	NS	K - Q
C/Hong Kong/14027/2018 cs		1466	3026	A	C	35	1.15	475	2	NS	K - T/P
C/Hong Kong/14027/2018 cs		1467	3010	A	G	60	1.99	475	3	NS	K/E/Q/T/A/P
C/Hong Kong/14027/2018 cs		1468	3201	G	GA	37	1.15	476		1 ins	FS
C/Hong Kong/14027/2018 cs		1469	3272	G	GA	38	1.16	476		1 ins	FS
C/Hong Kong/14027/2018 cs		1719	6011	A	G	171	2.84	559	3	S	G
C/Hong Kong/27393/2018 cs	S1	994	493	TG	T	20	4.04	318		1 del	FS
C/Hong Kong/27393/2018 cs		1079	424	C	T	17	4.01	346	2	NS	P - L
C/Hong Kong/27393/2018 cs		1388	467	T	C	48	10.28	449	2	NS	V - A
C/Hong Kong/27393/2018 cs		1389	561	T	A	56	9.96	449	3	S	V/A
C/Hong Kong/27393/2018 cs		1397	1521	G	A	165	10.85	452	2	NS	G - E
C/Hong Kong/27393/2018 cs		1432	3034	T	G	55	1.81	464	1	NS	S - A
C/Hong Kong/27393/2018 cs		1718	5454	G	A	112	2.05	559	2	NS	G - E
C/Hong Kong/27393/2018 cs		1719	5945	A	G	657	11.05	559	3	S	G/E
C/Hong Kong/27395/2018 cs	S1	373	2283	GT	G	23	1	111		1 del	FS
C/Hong Kong/27395/2018 cs		1388	2704	T	C	113	4.18	449	2	NS	V - A
C/Hong Kong/27395/2018 cs		1389	2835	T	A	136	4.79	449	3	S	V/A
C/Hong Kong/27395/2018 cs		1397	4284	G	A	249	5.81	452	2	NS	G - E
C/Hong Kong/27395/2018 cs		1719	12552	A	G	581	4.63	559	3	S	G
C/Hong Kong/27892/2018 cs	S1	196	703	A	G	59	8.39	52	1	NS	I - V
C/Hong Kong/27892/2018 cs		370	534	A	G	16	2.99	110	1	NS	K - E
C/Hong Kong/27892/2018 cs		374	461	T	C	11	2.39	111	2	NS	V- A
C/Hong Kong/27892/2018 cs		1388	466	T	C	19	4.08	449	2	NS	V - A
C/Hong Kong/27892/2018 cs		1389	538	T	A	20	3.70	449	3	S	V/A
C/Hong Kong/27892/2018 cs		1397	1399	G	A	75	5.35	452	2	NS	G - E
C/Hong Kong/27892/2018 cs		1432	2374	T	G	30	1.26	464	1	NS	S - A
C/Hong Kong/27892/2018 cs		1575	3470	T	C	75	2.16	511	3	S	D
C/Hong Kong/27892/2018 cs		1581	3223	C	T	98	3.04	513	3	S	T
C/Hong Kong/27892/2018 cs		1718	3608	G	A	54	1.50	559	2	NS	G - E
C/Hong Kong/27892/2018 cs		1719	3584	A	G	413	11.51	559	3	S	G/E
C/Hong Kong/27892/2018 cs		1748	3700	G	A	100	2.70	569	2	NS	C - Y
C/Hong Kong/27892/2018 cs		1952	2444	T	C	26	1.06	637	2	NS	I - T
C/Hong Kong/27990/2018 cs	S2	1388	475	T	C	43	9.05	449	2	NS	V - A
C/Hong Kong/27990/2018 cs		1389	514	T	A	55	10.70	449	3	S	V/A
C/Hong Kong/27990/2018 cs		1397	1400	G	A	118	8.42	452	2	NS	G - E
C/Hong Kong/27990/2018 cs		1432	2504	T	G	53	2.11	464	1	NS	S - A
C/Hong Kong/27990/2018 cs		1718	4430	G	A	48	1.08	559	2	NS	G - E
C/Hong Kong/27990/2018 cs		1719	4549	A	G	429	9.42	559	3	S	G/E
C/Hong Kong/28015/2018 cs	S1	1397	300	G	A	14	4.67	452	2	NS	G - E
C/Hong Kong/28015/2018 cs		1718	1291	G	A	61	4.73	559	2	NS	G - E

C/Hong Kong/28015/2018 cs		1721	1298	A	G	26	2.00	560	2	NS	D - G
C/Hong Kong/28015/2018 cs		1951	1548	A	G	35	2.26	637	1	NS	I - V
C/Hong Kong/30248/2018 cs	S1	164	184	C	T	10	5.43	41	2	NS	A - V
C/Hong Kong/30248/2018 cs		1397	436	G	A	42	9.63	452	2	NS	G - E
C/Hong Kong/30248/2018 cs		1432	561	T	G	14	2.49	464	1	NS	S - A
C/Hong Kong/30248/2018 cs		1455	617	G	A	12	1.94	471	3	S	E
C/Hong Kong/30248/2018 cs		1470	656	A	G	17	2.59	476	3	S	G
C/Hong Kong/30248/2018 cs		1473	638	C	T	13	2.04	477	3	S	F
C/Hong Kong/30248/2018 cs		1611	761	T	C	17	2.23	523	3	S	A
C/Hong Kong/30248/2018 cs		1615	758	T	C	19	2.51	525	1	S	L
C/Hong Kong/30248/2018 cs		1718	1317	G	A	63	4.78	559	2	NS	G - E
C/Hong Kong/30248/2018 cs		1721	1319	A	G	18	1.36	560	2	NS	D - G
C/Hong Kong/30248/2018 cs		1770	1645	T	C	41	2.49	576	3	S	I
C/Hong Kong/30248/2018 cs		1788	1730	A	G	43	2.48	582	3	S	Q
C/Hong Kong/30248/2018 cs		1891	1553	T	C	50	3.22	617	1	S	L
C/Hong Kong/33189/2018 cs	S1	1397	1449	G	A	91	6.27	452	2	NS	G - E
C/Hong Kong/33189/2018 cs		1491	3265	C	T	79	2.42	483	3	S	D
C/Hong Kong/33189/2018 cs		1611	5283	T	C	60	1.13	523	3	S	A
C/Hong Kong/33189/2018 cs		1718	6453	G	A	88	1.36	559	2	NS	G - E
C/Hong Kong/33189/2018 cs		1719	6951	A	G	1231	17.70	559	3	S	G/E
C/Hong Kong/33417/2018 cs	S1	1388	2662	T	C	154	5.79	449	2	NS	V - A
C/Hong Kong/33417/2018 cs		1389	2831	T	A	183	6.45	449	3	S	V/A
C/Hong Kong/33417/2018 cs		1397	4583	G	A	283	6.17	452	2	NS	G - E
C/Hong Kong/33417/2018 cs		1718	17791	G	A	207	1.16	559	2	NS	G - E
C/Hong Kong/33417/2018 cs		1719	19140	A	G	1237	6.46	559	3	S	G/E
C/Hong Kong/33535/2018 cs	S1	1388	2860	T	C	107	3.74	449	2	NS	V - A
C/Hong Kong/33535/2018 cs		1389	3009	T	A	121	4.01	449	3	S	V/A
C/Hong Kong/33535/2018 cs		1397	4354	G	A	293	6.73	452	2	NS	G - E
C/Hong Kong/33535/2018 cs		1718	13158	G	A	176	1.34	559	2	NS	G - E
C/Hong Kong/33535/2018 cs		1719	14112	A	G	941	6.67	559	3	S	G/E
C/Hong Kong/37128/2018 cs	S2	184	742	CAAAGTACAT	C	11	1.48	48		9 ins	3 aa ins
C/Hong Kong/37128/2018 cs		1388	927	T	C	126	13.59	449	2	NS	V - A
C/Hong Kong/37128/2018 cs		1389	1015	T	A	157	15.47	449	3	S	V/A
C/Hong Kong/37128/2018 cs		1397	2955	G	A	243	8.22	452	2	NS	G - E
C/Hong Kong/37128/2018 cs		1432	5570	T	G	107	1.92	464	1	NS	S - A
C/Hong Kong/37128/2018 cs		1461	5499	T	A	165	2.99	473	3	S	A
C/Hong Kong/37128/2018 cs		1464	6277	A	AG	102	1.62	474		1 ins	FS
C/Hong Kong/37128/2018 cs		1465	6236	A	G	119	1.89	475	1	NS	K - E
C/Hong Kong/37128/2018 cs		1466	6291	A	C	99	1.57	475	2	NS	K - T/A
C/Hong Kong/37128/2018 cs		1467	6270	A	G	97	1.55	475	3	NS	K/E/Q/T/A/P
C/Hong Kong/37128/2018 cs		1469	6488	G	GA	92	1.41	476		1 ins	FS
C/Hong Kong/37128/2018 cs		1718	14751	G	A	294	1.99	559	2	NS	G - E
C/Hong Kong/37128/2018 cs		1719	15183	A	G	2211	14.56	559	3	S	G/E
C/Hong Kong/37128/2018 E3(am3)		283	1871	C	A	58	3.10	81	1	NS	R - S
C/Hong Kong/37128/2018 E3(am3)		536	2326	A	T	311	13.18	165	2	NS	H - L
C/Hong Kong/37128/2018 E3(am3)		540	1964	G	T	305	15.48	166	3	S	A
C/Hong Kong/37128/2018 E3(am3)		541	2071	A	T	60	2.85	167	1	NS	T - S
C/Hong Kong/37128/2018 E3(am3)		548	1723	G	A	19	1.10	169	2	NS	G - E
C/Hong Kong/37128/2018 E3(am3)		549	1723	G	T	18	1.04	169	3	ns	G - G/D
C/Hong Kong/37128/2018 E3(am3)		1364	1948	T	C	37	1.90	441	2	NS	I - T
C/Hong Kong/37128/2018 E3(am3)		1388	1782	T	C	22	1.23	449	2	NS	V - A
C/Hong Kong/37128/2018 E3(am3)		1389	1965	T	A	28	1.42	449	3	S	V/A

C/Hong Kong/37128/2018 E3(am3)		1396	2265	G	A	74	3.27	452	1	NS	G - R
C/Hong Kong/37128/2018 E3(am3)		1397	2207	G	A	44	1.99	452	2	NS	G - E/K
C/Hong Kong/37128/2018 E3(am3)		1398	2610	A	G	220	8.43	452	3	S	G/R/E/K
C/Hong Kong/37128/2018 E3(am3)		1719	3234	A	G	56	1.73	559	3	S	G
C/Hong Kong/40626/2018 cs	S1	1388	441	T	C	64	14.51	449	2	NS	V - A
C/Hong Kong/40626/2018 cs		1389	507	T	A	73	14.40	449	3	S	V/A
C/Hong Kong/40626/2018 cs		1397	1819	G	A	203	11.16	452	2	NS	G - E
C/Hong Kong/40626/2018 cs		1432	3141	T	G	50	1.59	464	1	NS	S - A
C/Hong Kong/40626/2018 cs		1718	6578	G	A	160	2.43	559	2	NS	G - E
C/Hong Kong/40626/2018 cs		1719	6970	A	G	1016	14.57	559	3	S	G/E
C/Hong Kong/46081/2018 cs	S1	1397	198	G	A	13	6.57	452	2	NS	G - E
C/Hong Kong/46081/2018 cs		1432	298	T	G	16	5.37	464	1	NS	S - A
C/Hong Kong/46081/2018 cs		1467	369	A	AG	15	4.04	475		1 ins	FS
C/Hong Kong/46081/2018 cs		1718	799	G	A	53	6.63	559	2	NS	G - E
C/Hong Kong/46081/2018 cs		1813	1088	A	G	12	1.10	591	1	NS	T - A
C/Hong Kong/45913/2018 cs	S2	1388	535	T	C	87	16.23	449	2	NS	V - A
C/Hong Kong/45913/2018 cs		1389	570	T	A	107	18.71	449	3	S	V/A
C/Hong Kong/45913/2018 cs		1397	1936	G	A	212	10.94	452	2	NS	G - E
C/Hong Kong/45913/2018 cs		1432	3577	T	G	80	2.23	464	1	NS	S - A
C/Hong Kong/45913/2018 cs		1461	3631	T	A	103	2.83	473	3	S	A
C/Hong Kong/45913/2018 cs		1464	4310	A	AG	58	1.33	474		1 ins	FS
C/Hong Kong/45913/2018 cs		1465	4257	A	G	61	1.42	475	1	NS	K - E
C/Hong Kong/45913/2018 cs		1466	4236	A	C	54	1.27	475	2	NS	K - T/A
C/Hong Kong/45913/2018 cs		1467	4290	A	G	67	1.56	475	3	NS	K/E/Q/T/A/P
C/Hong Kong/45913/2018 cs		1468	4321	G	GA	46	1.06	476		1 ins	FS
C/Hong Kong/45913/2018 cs		1718	9333	G	A	215	2.30	559	2	NS	G - E
C/Hong Kong/45913/2018 cs		1719	9737	A	G	1416	14.53	559	3	S	G/E
C/Hong Kong/47455/2018 cs	S1	1397	203	G	A	11	5.42	452	2	NS	G - E
C/Hong Kong/47455/2018 cs		1718	545	G	A	17	3.12	559	2	NS	G - E
C/Hong Kong/47144/2018 cs	S1	1397	192	G	A	17	8.85	452	2	NS	G - E
C/Hong Kong/47144/2018 cs		1719	326	A	G	53	16.26	559	3	S	G
C/Hong Kong/50551/2018 cs	S1	1388	83	T	C	15	18.07	449	2	NS	V - A
C/Hong Kong/50551/2018 cs		1396	635	G	A	23	3.62	452	1	NS	G - R
C/Hong Kong/50551/2018 cs		1397	644	G	A	40	6.20	452	2	NS	G - E/K
C/Hong Kong/50551/2018 cs		1679	1253	C	T	14	1.12	546	2	NS	S - F
C/Hong Kong/50551/2018 cs		1719	1315	A	G	144	10.95	559	3	S	G
C/Hong Kong/50752/2018 cs	S1	1397	246	G	A	13	5.28	452	2	NS	G - E
C/Hong Kong/50752/2018 cs		1718	1702	G	A	73	4.29	559	2	NS	G - E
C/Hong Kong/50752/2018 cs		1721	1713	A	G	18	1.05	560	2	NS	D - G
C/Hong Kong/57791/2018 cs	S2	1397	248	G	A	32	12.90	452	2	NS	G - E
C/Hong Kong/57791/2018 cs		1432	409	T	G	20	4.88	464	1	NS	S - A
C/Hong Kong/57791/2018 cs		1456	450	T	C	15	3.33	472	1	NS	S - P
C/Hong Kong/57791/2018 cs		1765	527	G	A	15	2.85	575	1	NS	D - N
C/Hong Kong/57791/2018 cs		1770	531	C	T	14	2.64	576	3	S	I
C/Hong Kong/57791/2018 cs		1911	516	C	T	19	3.68	623	3	S	A
C/Hong Kong/60430/2018 cs	S1	1388	2339	T	C	130	5.56	449	2	NS	V - A
C/Hong Kong/60430/2018 cs		1389	2558	T	A	151	5.88	449	3	S	V/A
C/Hong Kong/60430/2018 cs		1397	4068	G	A	261	6.41	452	2	NS	G - E
C/Hong Kong/60430/2018 cs		1718	16256	G	A	209	1.29	559	2	NS	G - E
C/Hong Kong/60430/2018 cs		1719	17162	A	G	1299	7.57	559	3	S	G/E
C/Hong Kong/60430/2018 E3(am3)		536	5670	A	T	229	4.02	165	2	NS	H - L
C/Hong Kong/60430/2018 E3(am3)		540	5173	G	T	226	4.36	166	3	S	A

C/Hong Kong/60430/2018 E3(am3)		613	5130	A	C	581	11.32	191	1	NS	T - P
C/Hong Kong/60430/2018 E3(am3)		1372	5127	C	T	337	6.57	444	1	NS	L - F
C/Hong Kong/60430/2018 E3(am3)		1388	4810	T	C	52	1.08	449	2	NS	V - A
C/Hong Kong/60430/2018 E3(am3)		1389	5253	T	A	61	1.16	449	3	S	V/A
C/Hong Kong/60430/2018 E3(am3)		1396	6179	G	A	107	1.73	452	1	NS	G - R
C/Hong Kong/60430/2018 E3(am3)		1397	6036	G	A	101	1.67	452	2	NS	G - E/K
C/Hong Kong/60430/2018 E3(am3)		1398	6359	A	G	469	7.37	452	3	S	G/R/E/K
C/Hong Kong/60430/2018 E3(am3)		1399	6513	A	G	1263	19.39	453	1	NS	I - V
C/Hong Kong/94533/2018 cs	S2	43	2038	GA	G	24	1.17	1		1 del	FS
C/Hong Kong/94533/2018 cs		990	1471	C	T	50	3.40	316	3	S	S
C/Hong Kong/94533/2018 cs		1389	1825	T	A	60	3.29	449	3	S	V
C/Hong Kong/94533/2018 cs		1397	2753	G	A	163	5.92	452	2	NS	G - E
C/Hong Kong/94533/2018 cs		1432	5421	T	G	76	1.40	464	1	NS	S - A
C/Hong Kong/94533/2018 cs		1718	10318	G	A	182	1.76	559	2	NS	G - E
C/Hong Kong/94533/2018 cs		1719	10665	A	G	1233	11.55	559	3	S	G/E
C/Hong Kong/95361/2018 cs	S2	1397	487	G	A	52	10.66	452	2	NS	G - E
C/Hong Kong/95361/2018 cs		1432	749	T	G	23	3.05	464	1	NS	S - A
C/Hong Kong/95361/2018 cs		1520	953	T	C	10	1.05	493	2	NS	1 - T
C/Hong Kong/95361/2018 cs		1718	1017	G	A	28	2.75	559	2	NS	G - E
C/Hong Kong/95361/2018 cs		1719	1002	A	G	151	15.05	559	3	S	G/E
C/Hong Kong/95361/2018 E3(am3)		536	4075	A	T	534	13.10	165	2	NS	H - L
C/Hong Kong/95361/2018 E3(am3)		540	3686	G	T	497	13.48	166	3	S	A
C/Hong Kong/95361/2018 E3(am3)		541	3645	A	T	80	2.16	167	1	NS	T - S
C/Hong Kong/95361/2018 E3(am3)		548	3326	G	A	47	1.41	169	2	NS	G - E
C/Hong Kong/95361/2018 E3(am3)		549	3616	A	T	39	1.08	169	3	ns	G - G/D
C/Hong Kong/95361/2018 E3(am3)		1197	3645	T	C	50	1.37	385	3	S	P
C/Hong Kong/95361/2018 E3(am3)		1388	3403	T	C	36	1.06	449	2	NS	V - A
C/Hong Kong/95361/2018 E3(am3)		1389	3676	T	A	41	1.11	449	3	S	V/A
C/Hong Kong/95361/2018 E3(am3)		1396	4397	G	A	74	1.68	452	1	NS	G - R
C/Hong Kong/95361/2018 E3(am3)		1397	4163	G	A	74	1.78	452	2	NS	G - E/K
C/Hong Kong/95361/2018 E3(am3)		1398	4479	A	G	333	7.43	452	3	S	G/R/E/K
C/Hong Kong/96417/2018 cs	S1	656	903	C	T	16	1.77	205	2	NS	T - I
C/Hong Kong/96417/2018 cs		665	939	A	C	11	1.17	208	2	NS	N - T
C/Hong Kong/96417/2018 cs		732	1022	A	G	11	1.08	230	3	S	K
C/Hong Kong/96417/2018 cs		994	1236	TG	T	26	2.09	318		1 del	FS
C/Hong Kong/96417/2018 cs		1240	976	C	T	19	1.94	400	1	NS	P - S
C/Hong Kong/96417/2018 cs		1388	1036	T	C	57	5.50	449	2	NS	V - A
C/Hong Kong/96417/2018 cs		1397	2341	G	A	152	6.49	452	2	NS	G - E
C/Hong Kong/96417/2018 cs		1432	4814	T	G	99	2.05	464	1	NS	S - A
C/Hong Kong/96417/2018 cs		1537	5752	A	G	68	1.18	499	1	NS	N - D
C/Hong Kong/96417/2018 cs		1597	6576	G	A	129	1.96	519	1	NS	A - T
C/Hong Kong/96417/2018 cs		1718	7570	G	A	150	1.98	559	2	NS	G - E
C/Hong Kong/96417/2018 cs		1719	7740	A	G	1237	15.97	559	3	S	G/E
C/Hong Kong/96417/2018 cs		1896	6463	C	T	163	2.52	618	3	S	G
C/Hong Kong/96123/2018 cs	K	373	4733	GT	G	49	1.03	111		1 del	FS
C/Hong Kong/96123/2018 cs		1397	6224	G	A	184	2.96	452	2	NS	G - E
C/Hong Kong/96123/2018 cs		1432	9937	T	G	134	1.35	464	1	NS	S - A
C/Hong Kong/96123/2018 cs		1437	8133	A	G	92	1.13	465	3	S	G
C/Hong Kong/96123/2018 E3(am3)		1396	4430	G	A	102	2.30	452	1	NS	G - R
C/Hong Kong/96123/2018 E3(am3)		1397	4230	G	A	59	1.39	452	2	NS	G - E/K
C/Hong Kong/96123/2018 E3(am3)		1398	4586	A	G	342	7.45	452	3	S	G/R/E/K
C/Hong Kong/96123/2018 E3(am3)		1399	4633	A	G	923	19.92	453	1	NS	I - V

C/Hong Kong/96123/2018 E3(am3)		1833	4937	T	C	66	1.32	597	3	S	I
C/Hong Kong/1/2018 cs	S1	1059	1465	G	A	16	1.09	339	3	NS	M - I
C/Hong Kong/1/2018 cs		1397	3760	G	A	300	7.97	452	2	NS	G - E
C/Hong Kong/1/2018 cs		1718	14164	G	A	175	1.24	559	2	NS	G - E
C/Hong Kong/1/2018 cs		1719	15183	A	G	855	5.63	559	3	S	G/E
C/Hong Kong/1/2018 cs		1943	7933	G	A	156	1.97	634	2	NS	G - E
C/Hong Kong/1/2018 E3(am3)		331	2158	G	A	27	1.25	97	1	NS	G - R
C/Hong Kong/1/2018 E3(am3)		536	2474	A	T	54	2.18	165	2	NS	H - L
C/Hong Kong/1/2018 E3(am3)		540	2278	G	T	95	4.16	166	3	S	A
C/Hong Kong/1/2018 E3(am3)		541	2268	A	T	31	1.37	167	1	NS	T - S
C/Hong Kong/1/2018 E3(am3)		1388	2167	T	C	25	1.15	449	2	NS	V - A
C/Hong Kong/1/2018 E3(am3)		1389	2382	T	A	31	1.30	449	3	S	V/A
C/Hong Kong/1/2018 E3(am3)		1396	3034	G	A	58	1.91	452	1	NS	G - R
C/Hong Kong/1/2018 E3(am3)		1397	2838	G	A	54	1.90	452	2	NS	G - E/K
C/Hong Kong/1/2018 E3(am3)		1398	3039	A	G	330	10.86	452	3	S	G/R/E/K
C/Hong Kong/1/2018 E3(am3)		1941	2550	T	A	29	1.14	633	3	S	S
C/Hong Kong/1/2018 E3(am3)		1943	2082	G	A	26	1.25	634	2	NS	G - E
C/Hong Kong/1/2018 MDCK3		1388	1091	T	C	183	16.77	449	2	NS	V - A
C/Hong Kong/1/2018 MDCK3		1389	1315	T	A	225	17.11	449	3	S	V/A
C/Hong Kong/1/2018 MDCK3		1394	3241	C	CA	33	1.01	451		1 ins	FS
C/Hong Kong/1/2018 MDCK3		1397	4212	G	A	288	6.72	452	2	NS	G - E
C/Hong Kong/1/2018 MDCK3		1718	21719	G	A	243	1.12	559	2	NS	G - E
C/Hong Kong/1/2018 MDCK3		1719	21818	A	G	1421	6.51	559	3	S	G/E
C/Hong Kong/1/2018 MDCK3		1939	12173	T	C	291	2.39	633	1	NS	S - P
C/Hong Kong/1/2018 MDCK3		1943	11210	G	A	251	2.24	634	2	NS	G - E

Results of Varscan/bcftools analyses are shown for alternate nucleotides supported by at least 10 reads. [a]Virus name and passage history are shown by order as in Table S1 (cs = clinical specimen, MDCK = Madin Darby Canine Kidney cells, E = Egg [am = amniotic], LLC = LLC-MK2 a rhesus monkey kidney cell line). [b]Virus clade is indicated: Kanagawa (K), São Paulo 1 (S1) and São Paulo 2 (S2). [c]Nucleotide number relates to the complete HEF reading frame (inclusive of signal peptide). [d]Nucleotide numbering relates to the first nucleotide where there are insertions or deletions in some reads. [e]Amino acid position numbering relates to mature HEF (i.e.signal peptide removed) and numbers above 432 are in HEF2. [f]Mutations resulting in synonymous (S) or non-synonymous (NS/ns) amino acid substitutions and indicated, together with numbers of nucleotide insertions (ins) and deletions (del) as appropriate. [g]Amino acid substitutions, with generation of stop codons (*), and indels are indicated together with nucleotide insertions and deletions that would cause frame shifts (FS). Mutations in the same codon within an individual virus are highlighted.

Table S3. Primers used for ICV gene sequencing.

A: Primers for Sanger sequencing of the HE gene

Primer	Sequence	Position[c]	Length[d]	T_m (°C)[e]	Purification[f]
Forward					
HERTF1[a]	AGAAGCAGGGG*K*TTAATAATGTT	non-coding + 1-5	23	62-64	CART
HEF1	GTCTTGAATCA*R*AGTACATGG	175-195	21	58-60	CART
HEF2	GAATTGGCTTCA*M*AATCACATTG	472-494	23	62-64	CART
HEF3	CCAACAATGCAATGTGACATG	853-873	21	60	HPLC
HEF4[b]	CTTCCTCCCAAATTTGG*R*AGATG	1216-1238	23	66-68	CART
HEF5	GGATT*R*CAAGAATCTTT*R*TGGGA	1666-1688	23	62-66	CART
Reverse					
HER1	TGGGCCAAACATACTCA*R*CAT	327-307	21	60-62	CART
HER2	TTTTTTAAGAA*R*CTGT*Y*GTTGCA	584-562	23	58-62	CART
HER3	CTGGATCATGTCACATTGCAT	879-859	21	60	HPLC
HER4[a]	GGGTTTGGTTACAGTGGTATC	1329-1309	21	62	HPLC
HER5	TAAGCC*Y*AAGCTGCTTCCCCA	1899-1879	21	64-66	CART
HERTR1	CATT*W*TTCAAAATTGTC*Y*TAATCA	1989-1966	24	58-60	CART
HERTR2[b]	AGCAGTAGCAAGGGG*W*TTTTTGTT	non-coding	24	68	CART

B: Primers for NGS sequencing of the whole genome

Primer	Sequence	Position[c]	Length[d]	T_m (°C)[e]	Purification[f]	100µM mix (µl)[g]
Forward						
NGSCF1	*ACGCGTGATC*AGCAGAAGCAGAGG	non-coding	14 (+10)	44 (76)	HPLC	50
NGSCF2	*ACGCGTGATC*AGCAGAAGCAGGGG	non-coding	14 (+10)	46 (78)	HPLC	200
NGSCF3	*ACGCGTGATC*AGCAAAAGCAGGGG	non-coding	14 (+10)	44 (76)	HPLC	50
NGSCF4	*ACGCGTGATC*AGCAGAAGCAGGAG	non-coding	14 (+10)	44 (76)	HPLC	50
Reverse						
NGSCR1	*ACGCGTGATC*AGCAGTAGCAAGAGG	non-coding	15 (+10)	46 (78)	HPLC	100
NGSCR2	*ACGCGTGATC*AGCAGTAGCAAGGGG	non-coding	15 (+10)	48 (80)	HPLC	150
NGSCR3	*ACGCGTGATC*AGCAGTAGCAAGGAG	non-coding	15 (+10)	46 (78)	HPLC	50
NGSCR4	*ACGCGTGATC*AGCAGGAGCAAGGGG	non-coding	15 (+10)	50 (82)	HPLC	50
						700

Primers used for Sanger sequencing of the HE gene (A) and NGS whole genome sequencing (B) are shown. All sequences are given in the 5'-3' orientation as they should be synthesised. For Sanger sequencing primers, positions of polymorphism are shown in italics using IUPAC codes. Primers used for: [a]5'-half gene amplification, [b]3'-half gene amplification and 'internal' primers used for good coverage in Sanger sequencing; the half gene primer mixes used 5µM concentrations of each primer and for Sanger sequencing primer concentrations of 2µM were used. [c]Primers are positioned in the non-coding regions of genes and the coding region of the HE gene. [d]Primer lengths are shown and for NGS primers the MBT sequence (1) is shown in italics. [e]Approximate T_m values are shown accounting for the polymorphism in the Sanger sequencing primers and the increased T_m (in parentheses) after the first rounds of RTPCR due to the MBT sequence. [f]All primers were made by Sigma-Aldrich and supplied as 100µM stocks in water: CART = cartridge purified (due to nucleotide polymorphisms), HPLC = HPLC purified. [g]The 10x concentration primer mix for NGS is shown with amounts of individual primers adjusted for the number of genes that use each primer.

1. Zhou B, Wentworth DE. 2012. Influenza A virus molecular virology techniques. Methods Mol Biol 865:175-92.

Table S4. RTPCR set-up and thermal cycling conditions

A: Set-up for single reactions

Reagent	NGS	Sanger
Water[a]	10.65	16.3
2.5x Buffer[a]	12	16
5µM primer mix (Sanger)	--	3
10µM primer mix (NGS)	1.05	--
RNase inhibitor[b]	0.3	0.4
Enzyme mix[a]	1	1.3
	25µl	37µl
RNA extract	5	3
	30µl	40µl

B: Thermal cycling Conditions[c]

NGS		Sanger	
42°C/50m		42°C/30m	
50°C/10m		50°C/10m	
94°C/5m		94°C/5m	
94°C/30s	]	94°C/30s	]
43°C/30s	] +5	55°C/30s	] +40
68°C/4m	]	68°C/2m30s	]
94°C/30s	}	68°C/10m	
57°C/30s	} +31	Hold 4°C	
68°C/4m	}		
68°C/10m			
Hold 4°C			

[a]Reagents supplied in the QIAGEN OneStep *ahead* RT-PCR kit. [b]Promega RNasin® Ribonuclease Inhibitor (#N2515). [c]All RTPCRs were performed on a Bio-Rad DNA ENGINE DYAD Peltier thermal cycler with temperatures being calculated and repeat numbers of cycles indicated (+).
