# Supplementary material for: Molecular Characterization of Influenza C Viruses from Outbreaks in Hong Kong SAR, China
Source: J Virol. 2020 Oct 14;94(21):e01051-20. doi: 10.1128/JVI.01051-20 (PMC7565627; doi:10.1128/JVI.01051-20)
Supplement: Supplemental file 2 [file JVI.01051-20-s0002.pdf]

Table S5. GISAID accession numbers for gene sequences downloaded and used in phylogeny

Segment ID	Segment	Country	Collection date	Isolate-ID	Isolate name	Originating Lab	Submitting Lab	Authors
EPI231537	HE	Japan	1981-Jan-01	EPI_ISL_66336	C/Aichi/1/81		Import from public-domain	Matsuzaki,Y.; Sugawara,K.; Furuse,Y.; Shimotai,Y.; Hongo,S.; Oshitani,H.; Mizuta,K.; Nishimura,H.
EPI232028	MP	Japan	1981-Jan-01	EPI_ISL_66336	C/Aichi/1/81		Import from public-domain	Matsuzaki,Y.; Sugawara,K.; Furuse,Y.; Shimotai,Y.; Hongo,S.; Oshitani,H.; Mizuta,K.; Nishimura,H.
EPI816583	NP	Japan	1981-Jan-01	EPI_ISL_66336	C/Aichi/1/81		Import from public-domain	Matsuzaki,Y.; Sugawara,K.; Furuse,Y.; Shimotai,Y.; Hongo,S.; Oshitani,H.; Mizuta,K.; Nishimura,H.
EPI231542	NS	Japan	1981-Jan-01	EPI_ISL_66336	C/Aichi/1/81		Import from public-domain	Matsuzaki,Y.; Sugawara,K.; Furuse,Y.; Shimotai,Y.; Hongo,S.; Oshitani,H.; Mizuta,K.; Nishimura,H.
EPI816484	P3	Japan	1981-Jan-01	EPI_ISL_66336	C/Aichi/1/81		Import from public-domain	Matsuzaki,Y.; Sugawara,K.; Furuse,Y.; Shimotai,Y.; Hongo,S.; Oshitani,H.; Mizuta,K.; Nishimura,H.
EPI813815	PB1	Japan	1981-Jan-01	EPI_ISL_66336	C/Aichi/1/81		Import from public-domain	Matsuzaki,Y.; Sugawara,K.; Furuse,Y.; Shimotai,Y.; Hongo,S.; Oshitani,H.; Mizuta,K.; Nishimura,H.
EPI814538	PB2	Japan	1981-Jan-01	EPI_ISL_66336	C/Aichi/1/81		Import from public-domain	Matsuzaki,Y.; Sugawara,K.; Furuse,Y.; Shimotai,Y.; Hongo,S.; Oshitani,H.; Mizuta,K.; Nishimura,H.
EPI231580	HE	Japan	1999-Apr-07	EPI_ISL_66363	C/Aichi/1/99		Import from public-domain	Matsuzaki,Y.; Sugawara,K.; Furuse,Y.; Shimotai,Y.; Hongo,S.; Oshitani,H.; Mizuta,K.; Nishimura,H.
EPI231585	MP	Japan	1999-Apr-07	EPI_ISL_66363	C/Aichi/1/99		Import from public-domain	Matsuzaki,Y.; Sugawara,K.; Furuse,Y.; Shimotai,Y.; Hongo,S.; Oshitani,H.; Mizuta,K.; Nishimura,H.
EPI816611	NP	Japan	1999-Apr-07	EPI_ISL_66363	C/Aichi/1/99		Import from public-domain	Matsuzaki,Y.; Sugawara,K.; Furuse,Y.; Shimotai,Y.; Hongo,S.; Oshitani,H.; Mizuta,K.; Nishimura,H.
EPI231586	NS	Japan	1999-Apr-07	EPI_ISL_66363	C/Aichi/1/99		Import from public-domain	Matsuzaki,Y.; Sugawara,K.; Furuse,Y.; Shimotai,Y.; Hongo,S.; Oshitani,H.; Mizuta,K.; Nishimura,H.
EPI816506	P3	Japan	1999-Apr-07	EPI_ISL_66363	C/Aichi/1/99		Import from public-domain	Matsuzaki,Y.; Sugawara,K.; Furuse,Y.; Shimotai,Y.; Hongo,S.; Oshitani,H.; Mizuta,K.; Nishimura,H.
EPI816423	PB1	Japan	1999-Apr-07	EPI_ISL_66363	C/Aichi/1/99		Import from public-domain	Matsuzaki,Y.; Sugawara,K.; Furuse,Y.; Shimotai,Y.; Hongo,S.; Oshitani,H.; Mizuta,K.; Nishimura,H.
EPI813742	PB2	Japan	1999-Apr-07	EPI_ISL_66363	C/Aichi/1/99		Import from public-domain	Matsuzaki,Y.; Sugawara,K.; Furuse,Y.; Shimotai,Y.; Hongo,S.; Oshitani,H.; Mizuta,K.; Nishimura,H.
EPI403452	HE	Canada	2011-Apr-15	EPI_ISL_131508	C/Alberta/10161/2011		Import from public-domain	Pabbaraju,K.; Wong,S.; Wong,A.; May-Hadford,J.; Tellier,R.; Fonseca,K.
EPI403467	MP	Canada	2011-Apr-15	EPI_ISL_131508	C/Alberta/10161/2011		Import from public-domain	Pabbaraju,K.; Wong,S.; Wong,A.; May-Hadford,J.; Tellier,R.; Fonseca,K.
EPI403464	MP	Canada	2010-Dec-21	EPI_ISL_131515	C/Alberta/21100/2010		Import from public-domain	Pabbaraju,K.; Wong,S.; Wong,A.; May-Hadford,J.; Tellier,R.; Fonseca,K.
EPI403465	MP	Canada	2011-Jan-24	EPI_ISL_131516	C/Alberta/2193/2011		Import from public-domain	Pabbaraju,K.; Wong,S.; Wong,A.; May-Hadford,J.; Tellier,R.; Fonseca,K.
EPI403458	MP	Canada	2011-Feb-25	EPI_ISL_131513	C/Alberta/2616/2011		Import from public-domain	Pabbaraju,K.; Wong,S.; Wong,A.; May-Hadford,J.; Tellier,R.; Fonseca,K.
EPI403447	HE	Canada	2011-Mar-04	EPI_ISL_131503	C/Alberta/2921/2011		Import from public-domain	Pabbaraju,K.; Wong,S.; Wong,A.; May-Hadford,J.; Tellier,R.; Fonseca,K.
EPI403459	MP	Canada	2011-Mar-04	EPI_ISL_131503	C/Alberta/2921/2011		Import from public-domain	Pabbaraju,K.; Wong,S.; Wong,A.; May-Hadford,J.; Tellier,R.; Fonseca,K.
EPI403448	HE	Canada	2011-Mar-04	EPI_ISL_131504	C/Alberta/3087/2011		Import from public-domain	Pabbaraju,K.; Wong,S.; Wong,A.; May-Hadford,J.; Tellier,R.; Fonseca,K.
EPI403460	MP	Canada	2011-Mar-04	EPI_ISL_131504	C/Alberta/3087/2011		Import from public-domain	Pabbaraju,K.; Wong,S.; Wong,A.; May-Hadford,J.; Tellier,R.; Fonseca,K.
EPI403449	HE	Canada	2011-Mar-18	EPI_ISL_131505	C/Alberta/3502/2011		Import from public-domain	Pabbaraju,K.; Wong,S.; Wong,A.; May-Hadford,J.; Tellier,R.; Fonseca,K.
EPI403461	MP	Canada	2011-Mar-18	EPI_ISL_131505	C/Alberta/3502/2011		Import from public-domain	Pabbaraju,K.; Wong,S.; Wong,A.; May-Hadford,J.; Tellier,R.; Fonseca,K.
EPI403462	MP	Canada	2011-Apr-08	EPI_ISL_131514	C/Alberta/4406/2011		Import from public-domain	Pabbaraju,K.; Wong,S.; Wong,A.; May-Hadford,J.; Tellier,R.; Fonseca,K.
EPI403450	HE	Canada	2011-Feb-19	EPI_ISL_131506	C/Alberta/4753/2011		Import from public-domain	Pabbaraju,K.; Wong,S.; Wong,A.; May-Hadford,J.; Tellier,R.; Fonseca,K.
EPI403463	MP	Canada	2011-Feb-19	EPI_ISL_131506	C/Alberta/4753/2011		Import from public-domain	Pabbaraju,K.; Wong,S.; Wong,A.; May-Hadford,J.; Tellier,R.; Fonseca,K.
EPI403451	HE	Canada	2011-Feb-22	EPI_ISL_131507	C/Alberta/4941/2011		Import from public-domain	Pabbaraju,K.; Wong,S.; Wong,A.; May-Hadford,J.; Tellier,R.; Fonseca,K.
EPI403466	MP	Canada	2011-Feb-22	EPI_ISL_131507	C/Alberta/4941/2011		Import from public-domain	Pabbaraju,K.; Wong,S.; Wong,A.; May-Hadford,J.; Tellier,R.; Fonseca,K.
EPI232040	HE	United States	1950-Jan-01	EPI_ISL_66438	C/Ann Arbor/1/50		Import from public-domain	Muraki,Y.; Washioka,H.; Sugawara,K.; Matsuzaki,Y.; Takashita,E.; Hongo,S.
EPI232042	MP	United States	1950-Jan-01	EPI_ISL_66438	C/Ann Arbor/1/50		Import from public-domain	Muraki,Y.; Washioka,H.; Sugawara,K.; Matsuzaki,Y.; Takashita,E.; Hongo,S.
EPI232041	NP	United States	1950-Jan-01	EPI_ISL_66438	C/Ann Arbor/1/50		Import from public-domain	Muraki,Y.; Washioka,H.; Sugawara,K.; Matsuzaki,Y.; Takashita,E.; Hongo,S.

Segment ID	Segment	Country	Collection date	Isolate-ID	Isolate name	Originating Lab	Submitting Lab	Authors
EPI232043	NS	United States	1950-Jan-01	EPI_ISL_66438	C/Ann Arbor/1/50		Import from public-domain	Muraki,Y.; Washioka,H.; Sugawara,K.; Matsuzaki,Y.; Takashita,E.; Hongo,S.
EPI232039	P3	United States	1950-Jan-01	EPI_ISL_66438	C/Ann Arbor/1/50		Import from public-domain	Muraki,Y.; Washioka,H.; Sugawara,K.; Matsuzaki,Y.; Takashita,E.; Hongo,S.
EPI232038	PB1	United States	1950-Jan-01	EPI_ISL_66438	C/Ann Arbor/1/50		Import from public-domain	Muraki,Y.; Washioka,H.; Sugawara,K.; Matsuzaki,Y.; Takashita,E.; Hongo,S.
EPI232037	PB2	United States	1950-Jan-01	EPI_ISL_66438	C/Ann Arbor/1/50		Import from public-domain	Muraki,Y.; Washioka,H.; Sugawara,K.; Matsuzaki,Y.; Takashita,E.; Hongo,S.
EPI232045	HE	Japan	1974-Jan-01	EPI_ISL_66326	C/Aomori/74		Import from public-domain	Matsuzaki,Y.; Sugawara,K.; Furuse,Y.; Shimotai,Y.; Hongo,S.; Oshitani,H.; Mizuta,K.; Nishimura,H.
EPI232044	MP	Japan	1974-Jan-01	EPI_ISL_66326	C/Aomori/74		Import from public-domain	Matsuzaki,Y.; Sugawara,K.; Furuse,Y.; Shimotai,Y.; Hongo,S.; Oshitani,H.; Mizuta,K.; Nishimura,H.
EPI816575	NP	Japan	1974-Jan-01	EPI_ISL_66326	C/Aomori/74		Import from public-domain	Matsuzaki,Y.; Sugawara,K.; Furuse,Y.; Shimotai,Y.; Hongo,S.; Oshitani,H.; Mizuta,K.; Nishimura,H.
EPI231527	NS	Japan	1974-Jan-01	EPI_ISL_66326	C/Aomori/74		Import from public-domain	Matsuzaki,Y.; Sugawara,K.; Furuse,Y.; Shimotai,Y.; Hongo,S.; Oshitani,H.; Mizuta,K.; Nishimura,H.
EPI816465	P3	Japan	1974-Jan-01	EPI_ISL_66326	C/Aomori/74		Import from public-domain	Matsuzaki,Y.; Sugawara,K.; Furuse,Y.; Shimotai,Y.; Hongo,S.; Oshitani,H.; Mizuta,K.; Nishimura,H.
EPI813785	PB1	Japan	1974-Jan-01	EPI_ISL_66326	C/Aomori/74		Import from public-domain	Matsuzaki,Y.; Sugawara,K.; Furuse,Y.; Shimotai,Y.; Hongo,S.; Oshitani,H.; Mizuta,K.; Nishimura,H.
EPI814893	PB2	Japan	1974-Jan-01	EPI_ISL_66326	C/Aomori/74		Import from public-domain	Matsuzaki,Y.; Sugawara,K.; Furuse,Y.; Shimotai,Y.; Hongo,S.; Oshitani,H.; Mizuta,K.; Nishimura,H.
EPI231592	PB2	Germany	1985-Jan-01	EPI_ISL_66364	C/Berlin/1/85		Import from public-domain	Santibanez Koref,M.F.
EPI1183991	HE	Germany	2013-Feb-11	EPI_ISL_300539	C/Berlin/13-03024/2013		Robert Koch Institute Nationales Referenzzentrum für Influenza	Biere, B.; Fritsch, A.; Schweiger, B.
EPI581546	HE	Philippines	2013-Jan-01	EPI_ISL_176781	C/Biliran/1/2013		Import from public-domain	Odagiri,T.; Matsuzaki,Y.; Okamoto,M.; Hongo,S.; Oshitani,H.
EPI621713	MP	Philippines	2013-Jan-01	EPI_ISL_176781	C/Biliran/1/2013		Import from public-domain	Odagiri,T.; Matsuzaki,Y.; Okamoto,M.; Hongo,S.; Oshitani,H.
EPI621697	NS	Philippines	2013-Jan-01	EPI_ISL_176781	C/Biliran/1/2013		Import from public-domain	Odagiri,T.; Matsuzaki,Y.; Okamoto,M.; Hongo,S.; Oshitani,H.
EPI581547	HE	Philippines	2013-Jan-01	EPI_ISL_176782	C/Biliran/2/2013		Import from public-domain	Odagiri,T.; Matsuzaki,Y.; Okamoto,M.; Hongo,S.; Oshitani,H.
EPI621716	MP	Philippines	2013-Jan-01	EPI_ISL_176782	C/Biliran/2/2013		Import from public-domain	Odagiri,T.; Matsuzaki,Y.; Okamoto,M.; Hongo,S.; Oshitani,H.
EPI621698	NS	Philippines	2013-Jan-01	EPI_ISL_176782	C/Biliran/2/2013		Import from public-domain	Odagiri,T.; Matsuzaki,Y.; Okamoto,M.; Hongo,S.; Oshitani,H.
EPI581548	HE	Philippines	2013-Jan-01	EPI_ISL_176783	C/Biliran/3/2013		Import from public-domain	Odagiri,T.; Matsuzaki,Y.; Okamoto,M.; Hongo,S.; Oshitani,H.
EPI621715	MP	Philippines	2013-Jan-01	EPI_ISL_176783	C/Biliran/3/2013		Import from public-domain	Odagiri,T.; Matsuzaki,Y.; Okamoto,M.; Hongo,S.; Oshitani,H.
EPI621699	NS	Philippines	2013-Jan-01	EPI_ISL_176783	C/Biliran/3/2013		Import from public-domain	Odagiri,T.; Matsuzaki,Y.; Okamoto,M.; Hongo,S.; Oshitani,H.
EPI1183993	HE	Germany	2013-Mar-04	EPI_ISL_300541	C/Brandenburg/13-04332/2013		Robert Koch Institute Nationales Referenzzentrum für Influenza	Biere, B.; Fritsch, A.; Schweiger, B.
EPI1183996	HE	Germany	2013-Mar-25	EPI_ISL_300544	C/Brandenburg/13-05206/2013		Robert Koch Institute Nationales Referenzzentrum für Influenza	Biere, B.; Fritsch, A.; Schweiger, B.
EPI1432119	HE	France	2018-Mar-05	EPI_ISL_354111	C/Bretagne/1196/2018	Institut Pasteur	Institut Pasteur	Behillil,S
EPI1432103	HE	France	2013-Dec-23	EPI_ISL_354105	C/Bretagne/2503/2013	Institut Pasteur	Institut Pasteur	
EPI1432107	MP	France	2013-Dec-23	EPI_ISL_354105	C/Bretagne/2503/2013	Institut Pasteur	Institut Pasteur	
EPI1432106	NP	France	2013-Dec-23	EPI_ISL_354105	C/Bretagne/2503/2013	Institut Pasteur	Institut Pasteur	
EPI1432108	NS	France	2013-Dec-23	EPI_ISL_354105	C/Bretagne/2503/2013	Institut Pasteur	Institut Pasteur	
EPI1432109	P3	France	2013-Dec-23	EPI_ISL_354105	C/Bretagne/2503/2013	Institut Pasteur	Institut Pasteur	
EPI1432105	PB1	France	2013-Dec-23	EPI_ISL_354105	C/Bretagne/2503/2013	Institut Pasteur	Institut Pasteur	
EPI1432104	PB2	France	2013-Dec-23	EPI_ISL_354105	C/Bretagne/2503/2013	Institut Pasteur	Institut Pasteur	
EPI231531	HE	United States	1978-Jan-01	EPI_ISL_66330	C/California/78		Import from public-domain	Nakada,S.; Creager,R.S.; Krystal,M.; Aaronson,R.P.; Palese,P.

Segment ID	Segment	Country	Collection date	Isolate-ID	Isolate name	Originating Lab	Submitting Lab	Authors
EPI231593	MP	United States	1978-Jan-01	EPI_ISL_66365	C/California/78		Import from public-domain	Tada,Y.
EPI232019	NP	United States	1978-Jan-01	EPI_ISL_66330	C/California/78		Import from public-domain	Nakada,S.; Creager,R.S.; Krystal,M.; Aaronson,R.P.; Palese,P.
EPI232050	NS	United States	1978-Jan-01	EPI_ISL_66330	C/California/78		Import from public-domain	Nakada,S.; Creager,R.S.; Krystal,M.; Aaronson,R.P.; Palese,P.
EPI232048	P3	United States	1978-Jan-01	EPI_ISL_66365	C/California/78		Import from public-domain	Tada,Y.
EPI232047	PB1	United States	1978-Jan-01	EPI_ISL_66365	C/California/78		Import from public-domain	Tada,Y.
EPI232046	PB2	United States	1978-Jan-01	EPI_ISL_66365	C/California/78		Import from public-domain	Tada,Y.
EPI1259829	HE	Cameroon	2017-Dec-27	EPI_ISL_315877	C/Cameroon/13560/2017	Centre Pasteur du Cameroun	Crick Worldwide Influenza Centre	
EPI1259832	MP	Cameroon	2017-Dec-27	EPI_ISL_315877	C/Cameroon/13560/2017	Centre Pasteur du Cameroun	Crick Worldwide Influenza Centre	
EPI1259830	NP	Cameroon	2017-Dec-27	EPI_ISL_315877	C/Cameroon/13560/2017	Centre Pasteur du Cameroun	Crick Worldwide Influenza Centre	
EPI1259831	NS	Cameroon	2017-Dec-27	EPI_ISL_315877	C/Cameroon/13560/2017	Centre Pasteur du Cameroun	Crick Worldwide Influenza Centre	
EPI1259828	P3	Cameroon	2017-Dec-27	EPI_ISL_315877	C/Cameroon/13560/2017	Centre Pasteur du Cameroun	Crick Worldwide Influenza Centre	
EPI1259834	PB1	Cameroon	2017-Dec-27	EPI_ISL_315877	C/Cameroon/13560/2017	Centre Pasteur du Cameroun	Crick Worldwide Influenza Centre	
EPI1259833	PB2	Cameroon	2017-Dec-27	EPI_ISL_315877	C/Cameroon/13560/2017	Centre Pasteur du Cameroun	Crick Worldwide Influenza Centre	
EPI1259835	HE	Cameroon	2017-Dec-27	EPI_ISL_315878	C/Cameroon/13565/2017	Centre Pasteur du Cameroun	Crick Worldwide Influenza Centre	
EPI272316	HE	Spain	2009-Aug-25	EPI_ISL_77214	C/Catalonia/1266/2009		Import from public-domain	Anton,A.; de Molina,P.; Marcos,M.A.; Martinez,A.; Cardenosa,N.; Godoy,P.; Torner,N.; Martinez,M.J.; Ramon,S.; Tudo,G.; Isanta,R.;
EPI272317	HE	Spain	2009-Aug-26	EPI_ISL_77215	C/Catalonia/1284/2009		Import from public-domain	Anton,A.; de Molina,P.; Marcos,M.A.; Martinez,A.; Cardenosa,N.; Godoy,P.; Torner,N.; Martinez,M.J.; Ramon,S.; Tudo,G.; Isanta,R.;
EPI272318	HE	Spain	2009-Sep-01	EPI_ISL_77216	C/Catalonia/1318/2009		Import from public-domain	Anton,A.; de Molina,P.; Marcos,M.A.; Martinez,A.; Cardenosa,N.; Godoy,P.; Torner,N.; Martinez,M.J.; Ramon,S.; Tudo,G.; Isanta,R.;
EPI272319	HE	Spain	2009-Sep-14	EPI_ISL_77217	C/Catalonia/1372/2009		Import from public-domain	Anton,A.; de Molina,P.; Marcos,M.A.; Martinez,A.; Cardenosa,N.; Godoy,P.; Torner,N.; Martinez,M.J.; Ramon,S.; Tudo,G.; Isanta,R.;
EPI272320	HE	Spain	2009-Sep-09	EPI_ISL_77218	C/Catalonia/1373/2009		Import from public-domain	Anton,A.; de Molina,P.; Marcos,M.A.; Martinez,A.; Cardenosa,N.; Godoy,P.; Torner,N.; Martinez,M.J.; Ramon,S.; Tudo,G.; Isanta,R.;
EPI272321	HE	Spain	2009-Sep-28	EPI_ISL_77219	C/Catalonia/1430/2009		Import from public-domain	Anton,A.; de Molina,P.; Marcos,M.A.; Martinez,A.; Cardenosa,N.; Godoy,P.; Torner,N.; Martinez,M.J.; Ramon,S.; Tudo,G.; Isanta,R.;
EPI272322	HE	Spain	2009-Sep-29	EPI_ISL_77220	C/Catalonia/1457/2009		Import from public-domain	Anton,A.; de Molina,P.; Marcos,M.A.; Martinez,A.; Cardenosa,N.; Godoy,P.; Torner,N.; Martinez,M.J.; Ramon,S.; Tudo,G.; Isanta,R.;
EPI272323	HE	Spain	2009-Nov-09	EPI_ISL_77221	C/Catalonia/1754/2009		Import from public-domain	Anton,A.; de Molina,P.; Marcos,M.A.; Martinez,A.; Cardenosa,N.; Godoy,P.; Torner,N.; Martinez,M.J.; Ramon,S.; Tudo,G.; Isanta,R.;
EPI272324	HE	Spain	2009-Nov-11	EPI_ISL_77222	C/Catalonia/1824/2009		Import from public-domain	Anton,A.; de Molina,P.; Marcos,M.A.; Martinez,A.; Cardenosa,N.; Godoy,P.; Torner,N.; Martinez,M.J.; Ramon,S.; Tudo,G.; Isanta,R.;
EPI272325	HE	Spain	2009-Nov-23	EPI_ISL_77223	C/Catalonia/1945/2009		Import from public-domain	Anton,A.; de Molina,P.; Marcos,M.A.; Martinez,A.; Cardenosa,N.; Godoy,P.; Torner,N.; Martinez,M.J.; Ramon,S.; Tudo,G.; Isanta,R.;
EPI272326	HE	Spain	2009-Dec-09	EPI_ISL_77224	C/Catalonia/2072/2009		Import from public-domain	Anton,A.; de Molina,P.; Marcos,M.A.; Martinez,A.; Cardenosa,N.; Godoy,P.; Torner,N.; Martinez,M.J.; Ramon,S.; Tudo,G.; Isanta,R.;
EPI272327	HE	Spain	2010-Apr-13	EPI_ISL_77225	C/Catalonia/2588/2010		Import from public-domain	Anton,A.; de Molina,P.; Marcos,M.A.; Martinez,A.; Cardenosa,N.; Godoy,P.; Torner,N.; Martinez,M.J.; Ramon,S.; Tudo,G.; Isanta,R.;
EPI1345812	HE	Spain	2016-Dec-25	EPI_ISL_336014	C/Catalonia/NSVH100540232/2016	Hospital Universitari Vall d'Hebron	Hospital Universitari Vall d'Hebron	
EPI1345816	HE	Spain	2017-Jan-28	EPI_ISL_336018	C/Catalonia/NSVH100562094/2017	Hospital Universitari Vall d'Hebron	Hospital Universitari Vall d'Hebron	
EPI1345814	HE	Spain	2017-Mar-10	EPI_ISL_336016	C/Catalonia/NSVH100585686/2017	Hospital Universitari Vall d'Hebron	Hospital Universitari Vall d'Hebron	
EPI1440611	HE	Spain	2019-Jan-26	EPI_ISL_355939	C/Catalonia/NSVH100972890/2019	Hospital Universitari Vall d'Hebron	Hospital Universitari Vall d'Hebron	
EPI1345818	HE	Spain	2017-Apr-07	EPI_ISL_336020	C/Catalonia/NSVH110463212/2017	Hospital Universitari Vall d'Hebron	Hospital Universitari Vall d'Hebron	
EPI1345815	HE	Spain	2017-Apr-23	EPI_ISL_336017	C/Catalonia/NSVH110469004/2017	Hospital Universitari Vall d'Hebron	Hospital Universitari Vall d'Hebron	
EPI1345813	HE	Spain	2017-Jun-10	EPI_ISL_336015	C/Catalonia/NSVH110476640/2017	Hospital Universitari Vall d'Hebron	Hospital Universitari Vall d'Hebron	

Segment ID	Segment	Country	Collection date	Isolate-ID	Isolate name	Originating Lab	Submitting Lab	Authors
EPI1324929	HE	Spain	2018-Aug-25	EPI_ISL_332166	C/Catalonia/NSVH110683183/2018	Hospital Universitari Vall d'Hebron	Hospital Universitari Vall d'Hebron	
EPI463757	HE	India	2011-Jul-15	EPI_ISL_144547	C/Eastern India/1202/2011		Import from public-domain	Roy Mukherjee,T.; Chawla-Sarkar,M.
EPI463762	MP	India	2011-Jul-15	EPI_ISL_144547	C/Eastern India/1202/2011		Import from public-domain	Roy Mukherjee,T.; Chawla-Sarkar,M.
EPI463758	NP	India	2011-Jul-15	EPI_ISL_144547	C/Eastern India/1202/2011		Import from public-domain	Roy Mukherjee,T.; Chawla-Sarkar,M.
EPI463777	NS	India	2011-Jul-15	EPI_ISL_144547	C/Eastern India/1202/2011		Import from public-domain	Roy Mukherjee,T.; Chawla-Sarkar,M.
EPI463759	P3	India	2011-Jul-15	EPI_ISL_144547	C/Eastern India/1202/2011		Import from public-domain	Roy Mukherjee,T.; Chawla-Sarkar,M.
EPI463760	PB1	India	2011-Jul-15	EPI_ISL_144547	C/Eastern India/1202/2011		Import from public-domain	Roy Mukherjee,T.; Chawla-Sarkar,M.
EPI463761	PB2	India	2011-Jul-15	EPI_ISL_144547	C/Eastern India/1202/2011		Import from public-domain	Roy Mukherjee,T.; Chawla-Sarkar,M.
EPI231554	HE	United Kingdc	1983-Jan-01	EPI_ISL_66345	C/England/892/83[a]		Import from public-domain	Buonagurio,D.A.; Nakada,S.; Desselberger,U.; Krystal,M.; Palese,P.
EPI232051	MP	United Kingdc	1983-Jan-01	EPI_ISL_66366	C/England/83[a]		Import from public-domain	Gao,P.
EPI816590	NP	United Kingdc	1983-Aug-01	EPI_ISL_230236	C/England/892/1983[a]		Import from public-domain	Matsuzaki,Y.; Sugawara,K.; Furuse,Y.; Shimotai,Y.; Hongo,S.; Oshitani,H.; Mizuta,K.; Nishimura,H.
EPI232027	NS	United Kingdc	1983-Jan-01	EPI_ISL_66345	C/England/892/83[a]		Import from public-domain	Buonagurio,D.A.; Nakada,S.; Desselberger,U.; Krystal,M.; Palese,P.
EPI816491	P3	United Kingdc	1983-Aug-01	EPI_ISL_230236	C/England/892/1983[a]		Import from public-domain	Matsuzaki,Y.; Sugawara,K.; Furuse,Y.; Shimotai,Y.; Hongo,S.; Oshitani,H.; Mizuta,K.; Nishimura,H.
EPI813794	PB1	United Kingdc	1983-Aug-01	EPI_ISL_230236	C/England/892/1983[a]		Import from public-domain	Matsuzaki,Y.; Sugawara,K.; Furuse,Y.; Shimotai,Y.; Hongo,S.; Oshitani,H.; Mizuta,K.; Nishimura,H.
EPI813697	PB2	United Kingdc	1983-Aug-01	EPI_ISL_230236	C/England/892/1983[a]		Import from public-domain	Matsuzaki,Y.; Sugawara,K.; Furuse,Y.; Shimotai,Y.; Hongo,S.; Oshitani,H.; Mizuta,K.; Nishimura,H.
EPI711153	HE	Fiji	2012-Jul-10	EPI_ISL_212091	C/Fiji/18/2012	National Centre for Scientific Services for Virology and Vector Borne Diseases	WHO Collaborating Centre for Reference and Research on Influenza	
EPI813667	HE	Japan	2005-Dec-28	EPI_ISL_230252	C/Fukuoka/1/2005		Import from public-domain	Matsuzaki,Y.; Sugawara,K.; Furuse,Y.; Shimotai,Y.; Hongo,S.; Oshitani,H.; Mizuta,K.; Nishimura,H.
EPI816698	MP	Japan	2005-Dec-28	EPI_ISL_230252	C/Fukuoka/1/2005		Import from public-domain	Matsuzaki,Y.; Sugawara,K.; Furuse,Y.; Shimotai,Y.; Hongo,S.; Oshitani,H.; Mizuta,K.; Nishimura,H.
EPI816632	NP	Japan	2005-Dec-28	EPI_ISL_230252	C/Fukuoka/1/2005		Import from public-domain	Matsuzaki,Y.; Sugawara,K.; Furuse,Y.; Shimotai,Y.; Hongo,S.; Oshitani,H.; Mizuta,K.; Nishimura,H.
EPI816767	NS	Japan	2005-Dec-28	EPI_ISL_230252	C/Fukuoka/1/2005		Import from public-domain	Matsuzaki,Y.; Sugawara,K.; Furuse,Y.; Shimotai,Y.; Hongo,S.; Oshitani,H.; Mizuta,K.; Nishimura,H.
EPI816531	P3	Japan	2005-Dec-28	EPI_ISL_230252	C/Fukuoka/1/2005		Import from public-domain	Matsuzaki,Y.; Sugawara,K.; Furuse,Y.; Shimotai,Y.; Hongo,S.; Oshitani,H.; Mizuta,K.; Nishimura,H.
EPI813813	PB1	Japan	2005-Dec-28	EPI_ISL_230252	C/Fukuoka/1/2005		Import from public-domain	Matsuzaki,Y.; Sugawara,K.; Furuse,Y.; Shimotai,Y.; Hongo,S.; Oshitani,H.; Mizuta,K.; Nishimura,H.
EPI813716	PB2	Japan	2005-Dec-28	EPI_ISL_230252	C/Fukuoka/1/2005		Import from public-domain	Matsuzaki,Y.; Sugawara,K.; Furuse,Y.; Shimotai,Y.; Hongo,S.; Oshitani,H.; Mizuta,K.; Nishimura,H.
EPI813687	HE	Japan	2012-Apr-11	EPI_ISL_230272	C/Fukuoka/1/2012		Import from public-domain	Matsuzaki,Y.; Sugawara,K.; Furuse,Y.; Shimotai,Y.; Hongo,S.; Oshitani,H.; Mizuta,K.; Nishimura,H.
EPI816723	MP	Japan	2012-Apr-11	EPI_ISL_230272	C/Fukuoka/1/2012		Import from public-domain	Matsuzaki,Y.; Sugawara,K.; Furuse,Y.; Shimotai,Y.; Hongo,S.; Oshitani,H.; Mizuta,K.; Nishimura,H.
EPI816655	NP	Japan	2012-Apr-11	EPI_ISL_230272	C/Fukuoka/1/2012		Import from public-domain	Matsuzaki,Y.; Sugawara,K.; Furuse,Y.; Shimotai,Y.; Hongo,S.; Oshitani,H.; Mizuta,K.; Nishimura,H.
EPI816792	NS	Japan	2012-Apr-11	EPI_ISL_230272	C/Fukuoka/1/2012		Import from public-domain	Matsuzaki,Y.; Sugawara,K.; Furuse,Y.; Shimotai,Y.; Hongo,S.; Oshitani,H.; Mizuta,K.; Nishimura,H.
EPI816556	P3	Japan	2012-Apr-11	EPI_ISL_230272	C/Fukuoka/1/2012		Import from public-domain	Matsuzaki,Y.; Sugawara,K.; Furuse,Y.; Shimotai,Y.; Hongo,S.; Oshitani,H.; Mizuta,K.; Nishimura,H.
EPI816452	PB1	Japan	2012-Apr-11	EPI_ISL_230272	C/Fukuoka/1/2012		Import from public-domain	Matsuzaki,Y.; Sugawara,K.; Furuse,Y.; Shimotai,Y.; Hongo,S.; Oshitani,H.; Mizuta,K.; Nishimura,H.
EPI813762	PB2	Japan	2012-Apr-11	EPI_ISL_230272	C/Fukuoka/1/2012		Import from public-domain	Matsuzaki,Y.; Sugawara,K.; Furuse,Y.; Shimotai,Y.; Hongo,S.; Oshitani,H.; Mizuta,K.; Nishimura,H.
EPI228330	HE	Japan	2004-Jan-01	EPI_ISL_65166	C/Fukuoka/2/2004		Import from public-domain	Matsuzaki,Y.
EPI813668	HE	Japan	2006-Mar-08	EPI_ISL_230253	C/Fukuoka/2/2006		Import from public-domain	Matsuzaki,Y.; Sugawara,K.; Furuse,Y.; Shimotai,Y.; Hongo,S.; Oshitani,H.; Mizuta,K.; Nishimura,H.
EPI816699	MP	Japan	2006-Mar-08	EPI_ISL_230253	C/Fukuoka/2/2006		Import from public-domain	Matsuzaki,Y.; Sugawara,K.; Furuse,Y.; Shimotai,Y.; Hongo,S.; Oshitani,H.; Mizuta,K.; Nishimura,H.

Segment ID	Segment	Country	Collection date	Isolate-ID	Isolate name	Originating Lab	Submitting Lab	Authors
EPI816633	NP	Japan	2006-Mar-08	EPI_ISL_230253	C/Fukuoka/2/2006		Import from public-domain	Matsuzaki,Y.; Sugawara,K.; Furuse,Y.; Shimotai,Y.; Hongo,S.; Oshitani,H.; Mizuta,K.; Nishimura,H.
EPI816768	NS	Japan	2006-Mar-08	EPI_ISL_230253	C/Fukuoka/2/2006		Import from public-domain	Matsuzaki,Y.; Sugawara,K.; Furuse,Y.; Shimotai,Y.; Hongo,S.; Oshitani,H.; Mizuta,K.; Nishimura,H.
EPI816532	P3	Japan	2006-Mar-08	EPI_ISL_230253	C/Fukuoka/2/2006		Import from public-domain	Matsuzaki,Y.; Sugawara,K.; Furuse,Y.; Shimotai,Y.; Hongo,S.; Oshitani,H.; Mizuta,K.; Nishimura,H.
EPI813814	PB1	Japan	2006-Mar-08	EPI_ISL_230253	C/Fukuoka/2/2006		Import from public-domain	Matsuzaki,Y.; Sugawara,K.; Furuse,Y.; Shimotai,Y.; Hongo,S.; Oshitani,H.; Mizuta,K.; Nishimura,H.
EPI813717	PB2	Japan	2006-Mar-08	EPI_ISL_230253	C/Fukuoka/2/2006		Import from public-domain	Matsuzaki,Y.; Sugawara,K.; Furuse,Y.; Shimotai,Y.; Hongo,S.; Oshitani,H.; Mizuta,K.; Nishimura,H.
EPI228331	HE	Japan	2004-Jan-01	EPI_ISL_65167	C/Fukuoka/3/2004		Import from public-domain	Matsuzaki,Y.
EPI228325	HE	Japan	2004-Jan-01	EPI_ISL_65161	C/Fukushima/1/2004		Import from public-domain	Matsuzaki,Y.
EPI231525	HE	United States	1969-Jan-01	EPI_ISL_66324	C/Georgia/1/69		Import from public-domain	Matsuzaki,Y.; Sugawara,K.; Furuse,Y.; Shimotai,Y.; Hongo,S.; Oshitani,H.; Mizuta,K.; Nishimura,H.
EPI816671	MP	United States	1969-Jan-01	EPI_ISL_66324	C/Georgia/1/69		Import from public-domain	Matsuzaki,Y.; Sugawara,K.; Furuse,Y.; Shimotai,Y.; Hongo,S.; Oshitani,H.; Mizuta,K.; Nishimura,H.
EPI816573	NP	United States	1969-Jan-01	EPI_ISL_66324	C/Georgia/1/69		Import from public-domain	Matsuzaki,Y.; Sugawara,K.; Furuse,Y.; Shimotai,Y.; Hongo,S.; Oshitani,H.; Mizuta,K.; Nishimura,H.
EPI816739	NS	United States	1969-Jan-01	EPI_ISL_66324	C/Georgia/1/69		Import from public-domain	Matsuzaki,Y.; Sugawara,K.; Furuse,Y.; Shimotai,Y.; Hongo,S.; Oshitani,H.; Mizuta,K.; Nishimura,H.
EPI816463	P3	United States	1969-Jan-01	EPI_ISL_66324	C/Georgia/1/69		Import from public-domain	Matsuzaki,Y.; Sugawara,K.; Furuse,Y.; Shimotai,Y.; Hongo,S.; Oshitani,H.; Mizuta,K.; Nishimura,H.
EPI813783	PB1	United States	1969-Jan-01	EPI_ISL_66324	C/Georgia/1/69		Import from public-domain	Matsuzaki,Y.; Sugawara,K.; Furuse,Y.; Shimotai,Y.; Hongo,S.; Oshitani,H.; Mizuta,K.; Nishimura,H.
EPI814812	PB2	United States	1969-Jan-01	EPI_ISL_66324	C/Georgia/1/69		Import from public-domain	Matsuzaki,Y.; Sugawara,K.; Furuse,Y.; Shimotai,Y.; Hongo,S.; Oshitani,H.; Mizuta,K.; Nishimura,H.
EPI231516	HE	United States	1954-Jan-01	EPI_ISL_66318	C/Great Lakes/1167/54[b]		Import from public-domain	Buonagurio,D.A.; Nakada,S.; Desselberger,U.; Krystal,M.; Palese,P.
EPI816668	MP	United States	1954-Aug-01	EPI_ISL_230235	C/Great Lakes/1167/1954[b]		Import from public-domain	Matsuzaki,Y.; Sugawara,K.; Furuse,Y.; Shimotai,Y.; Hongo,S.; Oshitani,H.; Mizuta,K.; Nishimura,H.
EPI816570	NP	United States	1954-Aug-01	EPI_ISL_230235	C/Great Lakes/1167/1954[b]		Import from public-domain	Matsuzaki,Y.; Sugawara,K.; Furuse,Y.; Shimotai,Y.; Hongo,S.; Oshitani,H.; Mizuta,K.; Nishimura,H.
EPI232015	NS	United States	1954-Jan-01	EPI_ISL_66318	C/Great Lakes/1167/54[b]		Import from public-domain	Buonagurio,D.A.; Nakada,S.; Desselberger,U.; Krystal,M.; Palese,P.
EPI816459	P3	United States	1954-Aug-01	EPI_ISL_230235	C/Great Lakes/1167/1954[b]		Import from public-domain	Matsuzaki,Y.; Sugawara,K.; Furuse,Y.; Shimotai,Y.; Hongo,S.; Oshitani,H.; Mizuta,K.; Nishimura,H.
EPI813769	PB1	United States	1954-Aug-01	EPI_ISL_230235	C/Great Lakes/1167/1954[b]		Import from public-domain	Matsuzaki,Y.; Sugawara,K.; Furuse,Y.; Shimotai,Y.; Hongo,S.; Oshitani,H.; Mizuta,K.; Nishimura,H.
EPI813694	PB2	United States	1954-Aug-01	EPI_ISL_230235	C/Great Lakes/1167/1954[b]		Import from public-domain	Matsuzaki,Y.; Sugawara,K.; Furuse,Y.; Shimotai,Y.; Hongo,S.; Oshitani,H.; Mizuta,K.; Nishimura,H.
EPI231534	HE	Greece	1979-Jan-01	EPI_ISL_66333	C/Greece/1/79[c]		Import from public-domain	Matsuzaki,Y.; Sugawara,K.; Furuse,Y.; Shimotai,Y.; Hongo,S.; Oshitani,H.; Mizuta,K.; Nishimura,H.
EPI231604	MP	Greece	1979-Jan-01	EPI_ISL_66367	C/Greece/79[c]		Import from public-domain	Matsuzaki,Y.
EPI231603	NP	Greece	1979-Jan-01	EPI_ISL_66367	C/Greece/79[c]		Import from public-domain	Matsuzaki,Y.
EPI231600	NS	Greece	1979-Jan-01	EPI_ISL_66367	C/Greece/79[c]		Import from public-domain	Matsuzaki,Y.
EPI231602	P3	Greece	1979-Jan-01	EPI_ISL_66367	C/Greece/79[c]		Import from public-domain	Matsuzaki,Y.
EPI231601	PB1	Greece	1979-Jan-01	EPI_ISL_66367	C/Greece/79[c]		Import from public-domain	Matsuzaki,Y.
EPI231599	PB2	Greece	1979-Jan-01	EPI_ISL_66367	C/Greece/79[c]		Import from public-domain	Matsuzaki,Y.
EPI1183987	HE	Germany	2012-Nov-21	EPI_ISL_300535	C/Hamburg/13-00418/2012		Robert Koch Institute Nationales Referenzzentrum für Influenza	Biere, B.; Fritsch, A.; Schweiger, B.
EPI1432114	HE	France	2014-Apr-14	EPI_ISL_354108	C/Haute Normandie/1493/2014	Institut Pasteur	Institut Pasteur	Behillil,S
EPI1432117	HE	France	2016-Apr-01	EPI_ISL_354109	C/Haute normandie/2097/2016	Institut Pasteur	Institut Pasteur	Behillil,S
EPI1432112	HE	France	2014-Jan-30	EPI_ISL_354107	C/Haute Normandie/392/2014	Institut Pasteur	Institut Pasteur	Behillil,S
EPI1183994	HE	Germany	2013-Mar-11	EPI_ISL_300542	C/Hessen/13-04588/2013		Robert Koch Institute Nationales Referenzzentrum für Influenza	Biere, B.; Fritsch, A.; Schweiger, B.

Segment ID	Segment	Country	Collection date	Isolate-ID	Isolate name	Originating Lab	Submitting Lab	Authors
EPI232052	HE	Japan	2000-Jan-01	EPI_ISL_66439	C/Hiroshima/246/2000		Import from public-domain	Matsuzaki,Y.
EPI232058	MP	Japan	2000-Jan-01	EPI_ISL_66439	C/Hiroshima/246/2000		Import from public-domain	Matsuzaki,Y.
EPI232057	NS	Japan	2000-Jan-01	EPI_ISL_66439	C/Hiroshima/246/2000		Import from public-domain	Matsuzaki,Y.
EPI231612	HE	Japan	2000-Jan-01	EPI_ISL_66369	C/Hiroshima/247/2000		Import from public-domain	Matsuzaki,Y.
EPI231606	MP	Japan	2000-Jan-01	EPI_ISL_66369	C/Hiroshima/247/2000		Import from public-domain	Matsuzaki,Y.
EPI231607	NS	Japan	2000-Jan-01	EPI_ISL_66369	C/Hiroshima/247/2000		Import from public-domain	Matsuzaki,Y.
EPI232060	HE	Japan	2000-Jan-01	EPI_ISL_66441	C/Hiroshima/248/2000		Import from public-domain	Matsuzaki,Y.
EPI232066	MP	Japan	2000-Jan-01	EPI_ISL_66441	C/Hiroshima/248/2000		Import from public-domain	Matsuzaki,Y.
EPI232065	NS	Japan	2000-Jan-01	EPI_ISL_66441	C/Hiroshima/248/2000		Import from public-domain	Matsuzaki,Y.
EPI231619	HE	Japan	2000-Jan-01	EPI_ISL_66371	C/Hiroshima/249/2000		Import from public-domain	Matsuzaki,Y.
EPI231615	MP	Japan	2000-Jan-01	EPI_ISL_66371	C/Hiroshima/249/2000		Import from public-domain	Matsuzaki,Y.
EPI231616	NS	Japan	2000-Jan-01	EPI_ISL_66371	C/Hiroshima/249/2000		Import from public-domain	Matsuzaki,Y.
EPI232068	HE	Japan	2000-Jan-01	EPI_ISL_66443	C/Hiroshima/250/2000		Import from public-domain	Matsuzaki,Y.
EPI232074	MP	Japan	2000-Jan-01	EPI_ISL_66443	C/Hiroshima/250/2000		Import from public-domain	Matsuzaki,Y.
EPI232073	NS	Japan	2000-Jan-01	EPI_ISL_66443	C/Hiroshima/250/2000		Import from public-domain	Matsuzaki,Y.
EPI231628	HE	Japan	2000-Jan-01	EPI_ISL_66373	C/Hiroshima/251/2000		Import from public-domain	Matsuzaki,Y.
EPI231622	MP	Japan	2000-Jan-01	EPI_ISL_66373	C/Hiroshima/251/2000		Import from public-domain	Matsuzaki,Y.
EPI231623	NS	Japan	2000-Jan-01	EPI_ISL_66373	C/Hiroshima/251/2000		Import from public-domain	Matsuzaki,Y.
EPI231630	HE	Japan	1999-Jan-01	EPI_ISL_66375	C/Hiroshima/252/99		Import from public-domain	Matsuzaki,Y.
EPI231635	MP	Japan	1999-Jan-01	EPI_ISL_66375	C/Hiroshima/252/99		Import from public-domain	Matsuzaki,Y.
EPI231636	NS	Japan	1999-Jan-01	EPI_ISL_66375	C/Hiroshima/252/99		Import from public-domain	Matsuzaki,Y.
EPI231643	HE	Japan	1999-Jan-01	EPI_ISL_66376	C/Hiroshima/290/99		Import from public-domain	Matsuzaki,Y.
EPI231639	MP	Japan	1999-Jan-01	EPI_ISL_66376	C/Hiroshima/290/99		Import from public-domain	Matsuzaki,Y.
EPI231637	NS	Japan	1999-Jan-01	EPI_ISL_66376	C/Hiroshima/290/99		Import from public-domain	Matsuzaki,Y.
EPI228329	HE	Japan	2004-Jan-01	EPI_ISL_65165	C/Hiroshima/4/2004		Import from public-domain	Matsuzaki,Y.
EPI231555	HE	Japan	1983-Jan-01	EPI_ISL_66346	C/Hyogo/1/83		Import from public-domain	Adachi,K.; Kitame,F.; Sugawara,K.; Nishimura,H.; Nakamura,K.
EPI232077	MP	Japan	1983-Jan-01	EPI_ISL_66436	C/Hyogo/1/83		Import from public-domain	Matsuzaki,Y.
EPI232026	NS	Japan	1983-Jan-01	EPI_ISL_66436	C/Hyogo/1/83		Import from public-domain	Matsuzaki,Y.
EPI743436	HE	India	2012-Feb-24	EPI_ISL_218313	C/India/P121719/2012		Import from public-domain	Potdar,V.A.; Dakhave,M.R.; Manchanda,A.
EPI743438	MP	India	2012-Feb-24	EPI_ISL_218313	C/India/P121719/2012		Import from public-domain	Potdar,V.A.; Dakhave,M.R.; Manchanda,A.
EPI743437	NP	India	2012-Feb-24	EPI_ISL_218313	C/India/P121719/2012		Import from public-domain	Potdar,V.A.; Dakhave,M.R.; Manchanda,A.
EPI743538	NS	India	2012-Feb-24	EPI_ISL_218313	C/India/P121719/2012		Import from public-domain	Potdar,V.A.; Dakhave,M.R.; Manchanda,A.
EPI743435	P3	India	2012-Feb-24	EPI_ISL_218313	C/India/P121719/2012		Import from public-domain	Potdar,V.A.; Dakhave,M.R.; Manchanda,A.

Segment ID	Segment	Country	Collection date	Isolate-ID	Isolate name	Originating Lab	Submitting Lab	Authors
EPI743434	PB1	India	2012-Feb-24	EPI_ISL_218313	C/India/P121719/2012		Import from public-domain	Potdar,V.A.; Dakhave,M.R.; Manchanda,A.
EPI743433	PB2	India	2012-Feb-24	EPI_ISL_218313	C/India/P121719/2012		Import from public-domain	Potdar,V.A.; Dakhave,M.R.; Manchanda,A.
EPI743443	HE	India	2013-Apr-25	EPI_ISL_218314	C/India/P135047/2013		Import from public-domain	Potdar,V.A.; Dakhave,M.R.; Manchanda,A.
EPI743445	MP	India	2013-Apr-25	EPI_ISL_218314	C/India/P135047/2013		Import from public-domain	Potdar,V.A.; Dakhave,M.R.; Manchanda,A.
EPI743444	NP	India	2013-Apr-25	EPI_ISL_218314	C/India/P135047/2013		Import from public-domain	Potdar,V.A.; Dakhave,M.R.; Manchanda,A.
EPI743539	NS	India	2013-Apr-25	EPI_ISL_218314	C/India/P135047/2013		Import from public-domain	Potdar,V.A.; Dakhave,M.R.; Manchanda,A.
EPI743442	P3	India	2013-Apr-25	EPI_ISL_218314	C/India/P135047/2013		Import from public-domain	Potdar,V.A.; Dakhave,M.R.; Manchanda,A.
EPI743441	PB1	India	2013-Apr-25	EPI_ISL_218314	C/India/P135047/2013		Import from public-domain	Potdar,V.A.; Dakhave,M.R.; Manchanda,A.
EPI743440	PB2	India	2013-Apr-25	EPI_ISL_218314	C/India/P135047/2013		Import from public-domain	Potdar,V.A.; Dakhave,M.R.; Manchanda,A.
EPI283749	HE		1950-Jan-01	EPI_ISL_80396	C/JJ/1950[d]		Import from public-domain	Pachler,K.
EPI231515	MP		1950-Jan-01	EPI_ISL_66314	C/JJ/50[d]		Import from public-domain	Yamashita,M.; Krystal,M.; Palese,P.
EPI283750	NP		1950-Jan-01	EPI_ISL_80396	C/JJ/1950[d]		Import from public-domain	Pachler,K.
EPI283752	NS		1950-Jan-01	EPI_ISL_80396	C/JJ/1950[d]		Import from public-domain	Pachler,K.
EPI231509	P3		1950-Jan-01	EPI_ISL_66314	C/JJ/50[d]		Import from public-domain	Yamashita,M.; Krystal,M.; Palese,P.
EPI231510	PB1		1950-Jan-01	EPI_ISL_66314	C/JJ/50[d]		Import from public-domain	Yamashita,M.; Krystal,M.; Palese,P.
EPI231511	PB2		1950-Jan-01	EPI_ISL_66314	C/JJ/50[d]		Import from public-domain	Yamashita,M.; Krystal,M.; Palese,P.
EPI232083	HE	South Africa	1966-Jan-01	EPI_ISL_66432	C/Johannesburg/1/66		Import from public-domain	Crescenzo-Chaigne,B.
EPI232017	MP	South Africa	1966-Jan-01	EPI_ISL_66432	C/Johannesburg/1/66		Import from public-domain	Crescenzo-Chaigne,B.
EPI232078	NP	South Africa	1966-Jan-01	EPI_ISL_66432	C/Johannesburg/1/66		Import from public-domain	Crescenzo-Chaigne,B.
EPI232018	NS	South Africa	1966-Jan-01	EPI_ISL_66432	C/Johannesburg/1/66		Import from public-domain	Crescenzo-Chaigne,B.
EPI231519	NS	South Africa	1966-Jan-01	EPI_ISL_66321	C/Johannesburg/1/66		Import from public-domain	Buonagurio,D.A.; Nakada,S.; Fitch,W.M.; Palese,P.
EPI231520	NS	South Africa	1966-Jan-01	EPI_ISL_66321	C/Johannesburg/1/66		Import from public-domain	Buonagurio,D.A.; Nakada,S.; Fitch,W.M.; Palese,P.
EPI232079	P3	South Africa	1966-Jan-01	EPI_ISL_66432	C/Johannesburg/1/66		Import from public-domain	Crescenzo-Chaigne,B.
EPI232080	PB1	South Africa	1966-Jan-01	EPI_ISL_66432	C/Johannesburg/1/66		Import from public-domain	Crescenzo-Chaigne,B.
EPI232081	PB2	South Africa	1966-Jan-01	EPI_ISL_66432	C/Johannesburg/1/66		Import from public-domain	Crescenzo-Chaigne,B.
EPI231523	HE	South Africa	1967-Jan-01	EPI_ISL_66323	C/Johannesburg/4/67		Import from public-domain	Matsuzaki,Y.; Ikeda,T.; Abiko,C.; Aoki,Y.; Mizuta,K.; Shimotai,Y.; Sugawara,K.; Hongo,S.; Sugawara,K.; Furuse,Y.; Shimotai,Y.;
EPI816669	MP	South Africa	1967-Jan-01	EPI_ISL_66323	C/Johannesburg/4/67		Import from public-domain	Matsuzaki,Y.; Ikeda,T.; Abiko,C.; Aoki,Y.; Mizuta,K.; Shimotai,Y.; Sugawara,K.; Hongo,S.; Sugawara,K.; Furuse,Y.; Shimotai,Y.;
EPI369793	NP	South Africa	1967-Jan-01	EPI_ISL_118428	C/Johannesburg/4/67		Import from public-domain	Matsuzaki,Y.; Ikeda,T.; Abiko,C.; Aoki,Y.; Mizuta,K.; Shimotai,Y.; Sugawara,K.; Hongo,S.
EPI231524	NS	South Africa	1967-Jan-01	EPI_ISL_66323	C/Johannesburg/4/67		Import from public-domain	Matsuzaki,Y.; Ikeda,T.; Abiko,C.; Aoki,Y.; Mizuta,K.; Shimotai,Y.; Sugawara,K.; Hongo,S.; Sugawara,K.; Furuse,Y.; Shimotai,Y.;
EPI816461	P3	South Africa	1967-Jan-01	EPI_ISL_66323	C/Johannesburg/4/67		Import from public-domain	Matsuzaki,Y.; Ikeda,T.; Abiko,C.; Aoki,Y.; Mizuta,K.; Shimotai,Y.; Sugawara,K.; Hongo,S.; Sugawara,K.; Furuse,Y.; Shimotai,Y.;
EPI813784	PB1	South Africa	1967-Jan-01	EPI_ISL_66323	C/Johannesburg/4/67		Import from public-domain	Matsuzaki,Y.; Ikeda,T.; Abiko,C.; Aoki,Y.; Mizuta,K.; Shimotai,Y.; Sugawara,K.; Hongo,S.; Sugawara,K.; Furuse,Y.; Shimotai,Y.;
EPI814734	PB2	South Africa	1967-Jan-01	EPI_ISL_66323	C/Johannesburg/4/67		Import from public-domain	Matsuzaki,Y.; Ikeda,T.; Abiko,C.; Aoki,Y.; Mizuta,K.; Shimotai,Y.; Sugawara,K.; Hongo,S.; Sugawara,K.; Furuse,Y.; Shimotai,Y.;
EPI232085	HE	Japan	1976-Jan-01	EPI_ISL_66328	C/Kanagawa/1/76		Import from public-domain	Matsuzaki,Y.; Sugawara,K.; Furuse,Y.; Shimotai,Y.; Hongo,S.; Oshitani,H.; Mizuta,K.; Nishimura,H.

Segment ID	Segment	Country	Collection date	Isolate-ID	Isolate name	Originating Lab	Submitting Lab	Authors
EPI232086	MP	Japan	1976-Jan-01	EPI_ISL_66328	C/Kanagawa/1/76		Import from public-domain	Matsuzaki,Y.; Sugawara,K.; Furuse,Y.; Shimotai,Y.; Hongo,S.; Oshitani,H.; Mizuta,K.; Nishimura,H.
EPI816576	NP	Japan	1976-Jan-01	EPI_ISL_66328	C/Kanagawa/1/76		Import from public-domain	Matsuzaki,Y.; Sugawara,K.; Furuse,Y.; Shimotai,Y.; Hongo,S.; Oshitani,H.; Mizuta,K.; Nishimura,H.
EPI231529	NS	Japan	1976-Jan-01	EPI_ISL_66328	C/Kanagawa/1/76		Import from public-domain	Matsuzaki,Y.; Sugawara,K.; Furuse,Y.; Shimotai,Y.; Hongo,S.; Oshitani,H.; Mizuta,K.; Nishimura,H.
EPI816467	P3	Japan	1976-Jan-01	EPI_ISL_66328	C/Kanagawa/1/76		Import from public-domain	Matsuzaki,Y.; Sugawara,K.; Furuse,Y.; Shimotai,Y.; Hongo,S.; Oshitani,H.; Mizuta,K.; Nishimura,H.
EPI813787	PB1	Japan	1976-Jan-01	EPI_ISL_66328	C/Kanagawa/1/76		Import from public-domain	Matsuzaki,Y.; Sugawara,K.; Furuse,Y.; Shimotai,Y.; Hongo,S.; Oshitani,H.; Mizuta,K.; Nishimura,H.
EPI814540	PB2	Japan	1976-Jan-01	EPI_ISL_66328	C/Kanagawa/1/76		Import from public-domain	Matsuzaki,Y.; Sugawara,K.; Furuse,Y.; Shimotai,Y.; Hongo,S.; Oshitani,H.; Mizuta,K.; Nishimura,H.
EPI228327	HE	Japan	2004-Jan-01	EPI_ISL_65163	C/Kanagawa/2/2004		Import from public-domain	Matsuzaki,Y.
EPI231532	HE	United States	1979-Jan-01	EPI_ISL_66331	C/Kansas/1/79		Import from public-domain	Matsuzaki,Y.; Sugawara,K.; Furuse,Y.; Shimotai,Y.; Hongo,S.; Oshitani,H.; Mizuta,K.; Nishimura,H.
EPI231649	MP	United States	1979-Jan-01	EPI_ISL_66331	C/Kansas/1/79		Import from public-domain	Matsuzaki,Y.; Sugawara,K.; Furuse,Y.; Shimotai,Y.; Hongo,S.; Oshitani,H.; Mizuta,K.; Nishimura,H.
EPI816579	NP	United States	1979-Jan-01	EPI_ISL_66331	C/Kansas/1/79		Import from public-domain	Matsuzaki,Y.; Sugawara,K.; Furuse,Y.; Shimotai,Y.; Hongo,S.; Oshitani,H.; Mizuta,K.; Nishimura,H.
EPI231648	NS	United States	1979-Jan-01	EPI_ISL_66331	C/Kansas/1/79		Import from public-domain	Matsuzaki,Y.; Sugawara,K.; Furuse,Y.; Shimotai,Y.; Hongo,S.; Oshitani,H.; Mizuta,K.; Nishimura,H.
EPI816470	P3	United States	1979-Jan-01	EPI_ISL_66331	C/Kansas/1/79		Import from public-domain	Matsuzaki,Y.; Sugawara,K.; Furuse,Y.; Shimotai,Y.; Hongo,S.; Oshitani,H.; Mizuta,K.; Nishimura,H.
EPI813788	PB1	United States	1979-Jan-01	EPI_ISL_66331	C/Kansas/1/79		Import from public-domain	Matsuzaki,Y.; Sugawara,K.; Furuse,Y.; Shimotai,Y.; Hongo,S.; Oshitani,H.; Mizuta,K.; Nishimura,H.
EPI814533	PB2	United States	1979-Jan-01	EPI_ISL_66331	C/Kansas/1/79		Import from public-domain	Matsuzaki,Y.; Sugawara,K.; Furuse,Y.; Shimotai,Y.; Hongo,S.; Oshitani,H.; Mizuta,K.; Nishimura,H.
EPI231533	HE	United States	1979-Jan-01	EPI_ISL_66332	C/Kansas/2/79		Import from public-domain	Matsuzaki,Y.
EPI232087	HE	Japan	1979-Jan-01	EPI_ISL_66334	C/Kyoto/1/79		Import from public-domain	Matsuzaki,Y.; Sugawara,K.; Furuse,Y.; Shimotai,Y.; Hongo,S.; Oshitani,H.; Mizuta,K.; Nishimura,H.
EPI232088	MP	Japan	1979-Jan-01	EPI_ISL_66334	C/Kyoto/1/79		Import from public-domain	Matsuzaki,Y.; Sugawara,K.; Furuse,Y.; Shimotai,Y.; Hongo,S.; Oshitani,H.; Mizuta,K.; Nishimura,H.
EPI816580	NP	Japan	1979-Jan-01	EPI_ISL_66334	C/Kyoto/1/79		Import from public-domain	Matsuzaki,Y.; Sugawara,K.; Furuse,Y.; Shimotai,Y.; Hongo,S.; Oshitani,H.; Mizuta,K.; Nishimura,H.
EPI231535	NS	Japan	1979-Jan-01	EPI_ISL_66334	C/Kyoto/1/79		Import from public-domain	Matsuzaki,Y.; Sugawara,K.; Furuse,Y.; Shimotai,Y.; Hongo,S.; Oshitani,H.; Mizuta,K.; Nishimura,H.
EPI816481	P3	Japan	1979-Jan-01	EPI_ISL_66334	C/Kyoto/1/79		Import from public-domain	Matsuzaki,Y.; Sugawara,K.; Furuse,Y.; Shimotai,Y.; Hongo,S.; Oshitani,H.; Mizuta,K.; Nishimura,H.
EPI813791	PB1	Japan	1979-Jan-01	EPI_ISL_66334	C/Kyoto/1/79		Import from public-domain	Matsuzaki,Y.; Sugawara,K.; Furuse,Y.; Shimotai,Y.; Hongo,S.; Oshitani,H.; Mizuta,K.; Nishimura,H.
EPI814535	PB2	Japan	1979-Jan-01	EPI_ISL_66334	C/Kyoto/1/79		Import from public-domain	Matsuzaki,Y.; Sugawara,K.; Furuse,Y.; Shimotai,Y.; Hongo,S.; Oshitani,H.; Mizuta,K.; Nishimura,H.
EPI231544	HE	Japan	1982-Jan-01	EPI_ISL_66341	C/Kyoto/41/82		Import from public-domain	Adachi,K.; Kitame,F.; Sugawara,K.; Nishimura,H.; Nakamura,K.
EPI232089	MP	Japan	1982-Jan-01	EPI_ISL_66435	C/Kyoto/41/82		Import from public-domain	Matsuzaki,Y.
EPI232024	NS	Japan	1982-Jan-01	EPI_ISL_66435	C/Kyoto/41/82		Import from public-domain	Matsuzaki,Y.
EPI581537	HE	Philippines	2011-Jan-01	EPI_ISL_176772	C/Leyte/1/2011		Import from public-domain	Odagiri,T.; Matsuzaki,Y.; Okamoto,M.; Hongo,S.; Oshitani,H.
EPI621701	MP	Philippines	2011-Jan-01	EPI_ISL_176772	C/Leyte/1/2011		Import from public-domain	Odagiri,T.; Matsuzaki,Y.; Okamoto,M.; Hongo,S.; Oshitani,H.
EPI621685	NS	Philippines	2011-Jan-01	EPI_ISL_176772	C/Leyte/1/2011		Import from public-domain	Odagiri,T.; Matsuzaki,Y.; Okamoto,M.; Hongo,S.; Oshitani,H.
EPI581549	HE	Philippines	2013-Jan-01	EPI_ISL_176784	C/Leyte/1/2013		Import from public-domain	Odagiri,T.; Matsuzaki,Y.; Okamoto,M.; Hongo,S.; Oshitani,H.
EPI621704	MP	Philippines	2013-Jan-01	EPI_ISL_176784	C/Leyte/1/2013		Import from public-domain	Odagiri,T.; Matsuzaki,Y.; Okamoto,M.; Hongo,S.; Oshitani,H.
EPI621688	NS	Philippines	2013-Jan-01	EPI_ISL_176784	C/Leyte/1/2013		Import from public-domain	Odagiri,T.; Matsuzaki,Y.; Okamoto,M.; Hongo,S.; Oshitani,H.
EPI581538	HE	Philippines	2011-Jan-01	EPI_ISL_176773	C/Leyte/2/2011		Import from public-domain	Odagiri,T.; Matsuzaki,Y.; Okamoto,M.; Hongo,S.; Oshitani,H.
EPI621702	MP	Philippines	2011-Jan-01	EPI_ISL_176773	C/Leyte/2/2011		Import from public-domain	Odagiri,T.; Matsuzaki,Y.; Okamoto,M.; Hongo,S.; Oshitani,H.

Segment ID	Segment	Country	Collection date	Isolate-ID	Isolate name	Originating Lab	Submitting Lab	Authors
EPI621686	NS	Philippines	2011-Jan-01	EPI_ISL_176773	C/Leyte/2/2011		Import from public-domain	Odagiri,T.; Matsuzaki,Y.; Okamoto,M.; Hongo,S.; Oshitani,H.
EPI581550	HE	Philippines	2013-Jan-01	EPI_ISL_176785	C/Leyte/2/2013		Import from public-domain	Odagiri,T.; Matsuzaki,Y.; Okamoto,M.; Hongo,S.; Oshitani,H.
EPI621705	MP	Philippines	2013-Jan-01	EPI_ISL_176785	C/Leyte/2/2013		Import from public-domain	Odagiri,T.; Matsuzaki,Y.; Okamoto,M.; Hongo,S.; Oshitani,H.
EPI621689	NS	Philippines	2013-Jan-01	EPI_ISL_176785	C/Leyte/2/2013		Import from public-domain	Odagiri,T.; Matsuzaki,Y.; Okamoto,M.; Hongo,S.; Oshitani,H.
EPI581539	HE	Philippines	2011-Jan-01	EPI_ISL_176774	C/Leyte/3/2011		Import from public-domain	Odagiri,T.; Matsuzaki,Y.; Okamoto,M.; Hongo,S.; Oshitani,H.
EPI621703	MP	Philippines	2011-Jan-01	EPI_ISL_176774	C/Leyte/3/2011		Import from public-domain	Odagiri,T.; Matsuzaki,Y.; Okamoto,M.; Hongo,S.; Oshitani,H.
EPI621687	NS	Philippines	2011-Jan-01	EPI_ISL_176774	C/Leyte/3/2011		Import from public-domain	Odagiri,T.; Matsuzaki,Y.; Okamoto,M.; Hongo,S.; Oshitani,H.
EPI581551	HE	Philippines	2013-Jan-01	EPI_ISL_176787	C/Leyte/3/2013		Import from public-domain	Odagiri,T.; Matsuzaki,Y.; Okamoto,M.; Hongo,S.; Oshitani,H.
EPI621706	MP	Philippines	2013-Jan-01	EPI_ISL_176787	C/Leyte/3/2013		Import from public-domain	Odagiri,T.; Matsuzaki,Y.; Okamoto,M.; Hongo,S.; Oshitani,H.
EPI621690	NS	Philippines	2013-Jan-01	EPI_ISL_176787	C/Leyte/3/2013		Import from public-domain	Odagiri,T.; Matsuzaki,Y.; Okamoto,M.; Hongo,S.; Oshitani,H.
EPI581553	HE	Philippines	2011-Jan-01	EPI_ISL_176994	C/Leyte/4/2011		Import from public-domain	Odagiri,T.; Matsuzaki,Y.; Okamoto,M.; Suzuki,A.; Saito,M.; Tamaki,R.; Lupisan,S.P.; Sombrero,L.T.; Hongo,S.; Oshitani,H.
EPI581554	HE	Philippines	2011-Jan-01	EPI_ISL_176995	C/Leyte/5/2011		Import from public-domain	Odagiri,T.; Matsuzaki,Y.; Okamoto,M.; Suzuki,A.; Saito,M.; Tamaki,R.; Lupisan,S.P.; Sombrero,L.T.; Hongo,S.; Oshitani,H.
EPI475501	HE	Japan	2012-Jan-01	EPI_ISL_148120	C/Mie/180/2012cs		Import from public-domain	Yano,T.
EPI475497	HE	Japan	2012-Jan-01	EPI_ISL_148116	C/Mie/204/2012cs		Import from public-domain	Yano,T.
EPI1033772	HE	United States	2014-Dec-27	EPI_ISL_272577	C/Minnesota/01/2014	Minnesota Department of Health	Centers for Disease Control and Prevention	
EPI1033773	MP	United States	2014-Dec-27	EPI_ISL_272577	C/Minnesota/01/2014	Minnesota Department of Health	Centers for Disease Control and Prevention	
EPI1033774	HE	United States	2015-Jan-01	EPI_ISL_272578	C/Minnesota/01/2015	Minnesota Department of Health	Centers for Disease Control and Prevention	
EPI1033775	MP	United States	2015-Jan-01	EPI_ISL_272578	C/Minnesota/01/2015	Minnesota Department of Health	Centers for Disease Control and Prevention	
EPI1033840	HE	United States	2016-Apr-21	EPI_ISL_272613	C/Minnesota/01/2016	Minnesota Department of Health	Centers for Disease Control and Prevention	
EPI1033841	MP	United States	2016-Apr-21	EPI_ISL_272613	C/Minnesota/01/2016	Minnesota Department of Health	Centers for Disease Control and Prevention	
EPI1033770	HE	United States	2014-Dec-26	EPI_ISL_272576	C/Minnesota/02/2014	Minnesota Department of Health	Centers for Disease Control and Prevention	
EPI1033771	MP	United States	2014-Dec-26	EPI_ISL_272576	C/Minnesota/02/2014	Minnesota Department of Health	Centers for Disease Control and Prevention	
EPI1033776	HE	United States	2015-Jan-09	EPI_ISL_272579	C/Minnesota/02/2015	Minnesota Department of Health	Centers for Disease Control and Prevention	
EPI1033777	MP	United States	2015-Jan-09	EPI_ISL_272579	C/Minnesota/02/2015	Minnesota Department of Health	Centers for Disease Control and Prevention	
EPI1033821	HE	United States	2014-Dec-15	EPI_ISL_272603	C/Minnesota/03/2014	Minnesota Department of Health	Centers for Disease Control and Prevention	
EPI1033822	MP	United States	2014-Dec-15	EPI_ISL_272603	C/Minnesota/03/2014	Minnesota Department of Health	Centers for Disease Control and Prevention	
EPI1033778	HE	United States	2015-Jan-14	EPI_ISL_272580	C/Minnesota/03/2015	Minnesota Department of Health	Centers for Disease Control and Prevention	
EPI1033779	MP	United States	2015-Jan-14	EPI_ISL_272580	C/Minnesota/03/2015	Minnesota Department of Health	Centers for Disease Control and Prevention	
EPI1033823	HE	United States	2014-Dec-25	EPI_ISL_272604	C/Minnesota/04/2014	Minnesota Department of Health	Centers for Disease Control and Prevention	
EPI1033824	MP	United States	2014-Dec-25	EPI_ISL_272604	C/Minnesota/04/2014	Minnesota Department of Health	Centers for Disease Control and Prevention	
EPI1033780	HE	United States	2015-Feb-06	EPI_ISL_272581	C/Minnesota/04/2015	Minnesota Department of Health	Centers for Disease Control and Prevention	
EPI1033781	MP	United States	2015-Feb-06	EPI_ISL_272581	C/Minnesota/04/2015	Minnesota Department of Health	Centers for Disease Control and Prevention	
EPI1033782	HE	United States	2015-Feb-05	EPI_ISL_272582	C/Minnesota/05/2015	Minnesota Department of Health	Centers for Disease Control and Prevention	

Segment ID	Segment	Country	Collection date	Isolate-ID	Isolate name	Originating Lab	Submitting Lab	Authors
EPI1033783	MP	United States	2015-Feb-05	EPI_ISL_272582	C/Minnesota/05/2015	Minnesota Department of Health	Centers for Disease Control and Prevention	
EPI1033784	HE	United States	2015-Feb-15	EPI_ISL_272583	C/Minnesota/06/2015	Minnesota Department of Health	Centers for Disease Control and Prevention	
EPI1033785	MP	United States	2015-Feb-15	EPI_ISL_272583	C/Minnesota/06/2015	Minnesota Department of Health	Centers for Disease Control and Prevention	
EPI1033786	HE	United States	2015-Feb-18	EPI_ISL_272584	C/Minnesota/07/2015	Minnesota Department of Health	Centers for Disease Control and Prevention	
EPI1033787	MP	United States	2015-Feb-18	EPI_ISL_272584	C/Minnesota/07/2015	Minnesota Department of Health	Centers for Disease Control and Prevention	
EPI1033788	HE	United States	2015-Mar-04	EPI_ISL_272585	C/Minnesota/08/2015	Minnesota Department of Health	Centers for Disease Control and Prevention	
EPI1033789	MP	United States	2015-Mar-04	EPI_ISL_272585	C/Minnesota/08/2015	Minnesota Department of Health	Centers for Disease Control and Prevention	
EPI1033790	HE	United States	2015-Mar-10	EPI_ISL_272586	C/Minnesota/09/2015	Minnesota Department of Health	Centers for Disease Control and Prevention	
EPI1033792	HE	United States	2015-Mar-09	EPI_ISL_272587	C/Minnesota/10/2015	Minnesota Department of Health	Centers for Disease Control and Prevention	
EPI1033793	MP	United States	2015-Mar-09	EPI_ISL_272587	C/Minnesota/10/2015	Minnesota Department of Health	Centers for Disease Control and Prevention	
EPI1033794	HE	United States	2015-Mar-12	EPI_ISL_272588	C/Minnesota/12/2015	Minnesota Department of Health	Centers for Disease Control and Prevention	
EPI1033795	MP	United States	2015-Mar-12	EPI_ISL_272588	C/Minnesota/12/2015	Minnesota Department of Health	Centers for Disease Control and Prevention	
EPI1033796	HE	United States	2015-Mar-21	EPI_ISL_272589	C/Minnesota/13/2015	Minnesota Department of Health	Centers for Disease Control and Prevention	
EPI1033797	MP	United States	2015-Mar-21	EPI_ISL_272589	C/Minnesota/13/2015	Minnesota Department of Health	Centers for Disease Control and Prevention	
EPI1033798	HE	United States	2015-Mar-23	EPI_ISL_272590	C/Minnesota/14/2015	Minnesota Department of Health	Centers for Disease Control and Prevention	
EPI1033799	MP	United States	2015-Mar-23	EPI_ISL_272590	C/Minnesota/14/2015	Minnesota Department of Health	Centers for Disease Control and Prevention	
EPI1033800	HE	United States	2015-Mar-20	EPI_ISL_272591	C/Minnesota/15/2015	Minnesota Department of Health	Centers for Disease Control and Prevention	
EPI1033801	MP	United States	2015-Mar-20	EPI_ISL_272591	C/Minnesota/15/2015	Minnesota Department of Health	Centers for Disease Control and Prevention	
EPI1033802	HE	United States	2015-Mar-19	EPI_ISL_272592	C/Minnesota/16/2015	Minnesota Department of Health	Centers for Disease Control and Prevention	
EPI1033803	HE	United States	2015-Mar-27	EPI_ISL_272593	C/Minnesota/17/2015	Minnesota Department of Health	Centers for Disease Control and Prevention	
EPI1033804	HE	United States	2015-Mar-27	EPI_ISL_272594	C/Minnesota/18/2015	Minnesota Department of Health	Centers for Disease Control and Prevention	
EPI1033805	MP	United States	2015-Mar-27	EPI_ISL_272594	C/Minnesota/18/2015	Minnesota Department of Health	Centers for Disease Control and Prevention	
EPI1033806	HE	United States	2015-Mar-30	EPI_ISL_272595	C/Minnesota/19/2015	Minnesota Department of Health	Centers for Disease Control and Prevention	
EPI1033807	MP	United States	2015-Mar-30	EPI_ISL_272595	C/Minnesota/19/2015	Minnesota Department of Health	Centers for Disease Control and Prevention	
EPI1033808	HE	United States	2015-Mar-29	EPI_ISL_272596	C/Minnesota/20/2015	Minnesota Department of Health	Centers for Disease Control and Prevention	
EPI1033809	MP	United States	2015-Mar-29	EPI_ISL_272596	C/Minnesota/20/2015	Minnesota Department of Health	Centers for Disease Control and Prevention	
EPI1033810	HE	United States	2015-Mar-29	EPI_ISL_272597	C/Minnesota/21/2015	Minnesota Department of Health	Centers for Disease Control and Prevention	
EPI1033811	HE	United States	2015-Mar-31	EPI_ISL_272598	C/Minnesota/22/2015	Minnesota Department of Health	Centers for Disease Control and Prevention	
EPI1033812	MP	United States	2015-Mar-31	EPI_ISL_272598	C/Minnesota/22/2015	Minnesota Department of Health	Centers for Disease Control and Prevention	
EPI1033813	HE	United States	2015-Apr-15	EPI_ISL_272599	C/Minnesota/23/2015	Minnesota Department of Health	Centers for Disease Control and Prevention	
EPI1033814	MP	United States	2015-Apr-15	EPI_ISL_272599	C/Minnesota/23/2015	Minnesota Department of Health	Centers for Disease Control and Prevention	
EPI1033815	HE	United States	2015-Apr-14	EPI_ISL_272600	C/Minnesota/24/2015	Minnesota Department of Health	Centers for Disease Control and Prevention	
EPI1033816	MP	United States	2015-Apr-14	EPI_ISL_272600	C/Minnesota/24/2015	Minnesota Department of Health	Centers for Disease Control and Prevention	

Segment ID	Segment	Country	Collection date	Isolate-ID	Isolate name	Originating Lab	Submitting Lab	Authors
EPI1033817	HE	United States	2015-Apr-14	EPI_ISL_272601	C/Minnesota/25/2015	Minnesota Department of Health	Centers for Disease Control and Prevention	
EPI1033818	MP	United States	2015-Apr-14	EPI_ISL_272601	C/Minnesota/25/2015	Minnesota Department of Health	Centers for Disease Control and Prevention	
EPI1033819	HE	United States	2015-Apr-15	EPI_ISL_272602	C/Minnesota/26/2015	Minnesota Department of Health	Centers for Disease Control and Prevention	
EPI1033820	MP	United States	2015-Apr-15	EPI_ISL_272602	C/Minnesota/26/2015	Minnesota Department of Health	Centers for Disease Control and Prevention	
EPI1033825	HE	United States	2015-Feb-04	EPI_ISL_272605	C/Minnesota/27/2015	Minnesota Department of Health	Centers for Disease Control and Prevention	
EPI1033826	MP	United States	2015-Feb-04	EPI_ISL_272605	C/Minnesota/27/2015	Minnesota Department of Health	Centers for Disease Control and Prevention	
EPI1033827	HE	United States	2015-Feb-08	EPI_ISL_272606	C/Minnesota/28/2015	Minnesota Department of Health	Centers for Disease Control and Prevention	
EPI1033828	MP	United States	2015-Feb-08	EPI_ISL_272606	C/Minnesota/28/2015	Minnesota Department of Health	Centers for Disease Control and Prevention	
EPI1033829	HE	United States	2015-Feb-23	EPI_ISL_272607	C/Minnesota/29/2015	Minnesota Department of Health	Centers for Disease Control and Prevention	
EPI1033830	MP	United States	2015-Feb-23	EPI_ISL_272607	C/Minnesota/29/2015	Minnesota Department of Health	Centers for Disease Control and Prevention	
EPI1033831	HE	United States	2015-Feb-23	EPI_ISL_272608	C/Minnesota/30/2015	Minnesota Department of Health	Centers for Disease Control and Prevention	
EPI1033832	MP	United States	2015-Feb-23	EPI_ISL_272608	C/Minnesota/30/2015	Minnesota Department of Health	Centers for Disease Control and Prevention	
EPI1033833	HE	United States	2015-Feb-28	EPI_ISL_272609	C/Minnesota/31/2015	Minnesota Department of Health	Centers for Disease Control and Prevention	
EPI1033834	MP	United States	2015-Feb-28	EPI_ISL_272609	C/Minnesota/31/2015	Minnesota Department of Health	Centers for Disease Control and Prevention	
EPI1033835	HE	United States	2015-Mar-02	EPI_ISL_272610	C/Minnesota/32/2015	Minnesota Department of Health	Centers for Disease Control and Prevention	
EPI1033836	MP	United States	2015-Mar-02	EPI_ISL_272610	C/Minnesota/32/2015	Minnesota Department of Health	Centers for Disease Control and Prevention	
EPI1033837	HE	United States	2015-Mar-11	EPI_ISL_272611	C/Minnesota/33/2015	Minnesota Department of Health	Centers for Disease Control and Prevention	
EPI1033838	MP	United States	2015-Mar-11	EPI_ISL_272611	C/Minnesota/33/2015	Minnesota Department of Health	Centers for Disease Control and Prevention	
EPI231536	HE	United States	1980-Jan-01	EPI_ISL_66335	C/Mississippi/80		Import from public-domain	Buonagurio,D.A.; Nakada,S.; Desselberger,U.; Krystal,M.; Palese,P.
EPI232094	MP	United States	1980-Jan-01	EPI_ISL_230286	C/Mississippi/80		Import from public-domain	Tada,Y.; Hongo,S.; Muraki,Y.; Sugawara,K.; Kitame,F.; Nakamura,K.
EPI816582	NP	United States	1980-Jan-01	EPI_ISL_230286	C/Mississippi/80		Import from public-domain	Tada,Y.; Hongo,S.; Muraki,Y.; Sugawara,K.; Kitame,F.; Nakamura,K.
EPI232095	NS	United States	1980-Jan-01	EPI_ISL_66335	C/Mississippi/80		Import from public-domain	Buonagurio,D.A.; Nakada,S.; Desselberger,U.; Krystal,M.; Palese,P.
EPI816483	P3	United States	1980-Jan-01	EPI_ISL_230286	C/Mississippi/80		Import from public-domain	Tada,Y.; Hongo,S.; Muraki,Y.; Sugawara,K.; Kitame,F.; Nakamura,K.
EPI815430	PB1	United States	1980-Jan-01	EPI_ISL_230286	C/Mississippi/80		Import from public-domain	Tada,Y.; Hongo,S.; Muraki,Y.; Sugawara,K.; Kitame,F.; Nakamura,K.
EPI814537	PB2	United States	1980-Jan-01	EPI_ISL_230286	C/Mississippi/80		Import from public-domain	Tada,Y.; Hongo,S.; Muraki,Y.; Sugawara,K.; Kitame,F.; Nakamura,K.
EPI231660	HE	Japan	1990-Jan-01	EPI_ISL_66355	C/Miyagi/1/90		Import from public-domain	Matsuzaki,Y.
EPI231665	MP	Japan	1990-Jan-01	EPI_ISL_66355	C/Miyagi/1/90		Import from public-domain	Matsuzaki,Y.
EPI231566	NS	Japan	1990-Jan-01	EPI_ISL_66355	C/Miyagi/1/90		Import from public-domain	Matsuzaki,Y.
EPI231666	HE	Japan	1993-Feb-02	EPI_ISL_66378	C/Miyagi/1/93		Import from public-domain	Matsuzaki,Y.; Sugawara,K.; Furuse,Y.; Shimotai,Y.; Hongo,S.; Oshitani,H.; Mizuta,K.; Nishimura,H.
EPI231671	MP	Japan	1993-Feb-02	EPI_ISL_66378	C/Miyagi/1/93		Import from public-domain	Matsuzaki,Y.; Sugawara,K.; Furuse,Y.; Shimotai,Y.; Hongo,S.; Oshitani,H.; Mizuta,K.; Nishimura,H.
EPI816604	NP	Japan	1993-Feb-02	EPI_ISL_66378	C/Miyagi/1/93		Import from public-domain	Matsuzaki,Y.; Sugawara,K.; Furuse,Y.; Shimotai,Y.; Hongo,S.; Oshitani,H.; Mizuta,K.; Nishimura,H.
EPI231672	NS	Japan	1993-Feb-02	EPI_ISL_66378	C/Miyagi/1/93		Import from public-domain	Matsuzaki,Y.; Sugawara,K.; Furuse,Y.; Shimotai,Y.; Hongo,S.; Oshitani,H.; Mizuta,K.; Nishimura,H.
EPI816499	P3	Japan	1993-Feb-02	EPI_ISL_66378	C/Miyagi/1/93		Import from public-domain	Matsuzaki,Y.; Sugawara,K.; Furuse,Y.; Shimotai,Y.; Hongo,S.; Oshitani,H.; Mizuta,K.; Nishimura,H.

Segment ID	Segment	Country	Collection date	Isolate-ID	Isolate name	Originating Lab	Submitting Lab	Authors
EPI816416	PB1	Japan	1993-Feb-02	EPI_ISL_66378	C/Miyagi/1/93		Import from public-domain	Matsuzaki,Y.; Sugawara,K.; Furuse,Y.; Shimotai,Y.; Hongo,S.; Oshitani,H.; Mizuta,K.; Nishimura,H.
EPI813731	PB2	Japan	1993-Feb-02	EPI_ISL_66378	C/Miyagi/1/93		Import from public-domain	Matsuzaki,Y.; Sugawara,K.; Furuse,Y.; Shimotai,Y.; Hongo,S.; Oshitani,H.; Mizuta,K.; Nishimura,H.
EPI231673	HE	Japan	1994-Jan-01	EPI_ISL_66379	C/Miyagi/1/94		Import from public-domain	Matsuzaki,Y.
EPI231678	MP	Japan	1994-Jan-01	EPI_ISL_66379	C/Miyagi/1/94		Import from public-domain	Matsuzaki,Y.
EPI231679	NS	Japan	1994-Jan-01	EPI_ISL_66379	C/Miyagi/1/94		Import from public-domain	Matsuzaki,Y.
EPI231680	HE	Japan	1997-Jan-01	EPI_ISL_66380	C/Miyagi/1/97		Import from public-domain	Matsuzaki,Y.
EPI231685	MP	Japan	1997-Jan-01	EPI_ISL_66380	C/Miyagi/1/97		Import from public-domain	Matsuzaki,Y.
EPI231686	NS	Japan	1997-Jan-01	EPI_ISL_66380	C/Miyagi/1/97		Import from public-domain	Matsuzaki,Y.
EPI231687	HE	Japan	1999-Jan-01	EPI_ISL_66381	C/Miyagi/1/99		Import from public-domain	Matsuzaki,Y.
EPI231692	MP	Japan	1999-Jan-01	EPI_ISL_66381	C/Miyagi/1/99		Import from public-domain	Matsuzaki,Y.
EPI231693	NS	Japan	1999-Jan-01	EPI_ISL_66381	C/Miyagi/1/99		Import from public-domain	Matsuzaki,Y.
EPI813654	HE	Japan	2002-Feb-22	EPI_ISL_230239	C/Miyagi/11/2002		Import from public-domain	Matsuzaki,Y.; Sugawara,K.; Furuse,Y.; Shimotai,Y.; Hongo,S.; Oshitani,H.; Mizuta,K.; Nishimura,H.
EPI816679	MP	Japan	2002-Feb-22	EPI_ISL_230239	C/Miyagi/11/2002		Import from public-domain	Matsuzaki,Y.; Sugawara,K.; Furuse,Y.; Shimotai,Y.; Hongo,S.; Oshitani,H.; Mizuta,K.; Nishimura,H.
EPI816615	NP	Japan	2002-Feb-22	EPI_ISL_230239	C/Miyagi/11/2002		Import from public-domain	Matsuzaki,Y.; Sugawara,K.; Furuse,Y.; Shimotai,Y.; Hongo,S.; Oshitani,H.; Mizuta,K.; Nishimura,H.
EPI816748	NS	Japan	2002-Feb-22	EPI_ISL_230239	C/Miyagi/11/2002		Import from public-domain	Matsuzaki,Y.; Sugawara,K.; Furuse,Y.; Shimotai,Y.; Hongo,S.; Oshitani,H.; Mizuta,K.; Nishimura,H.
EPI816512	P3	Japan	2002-Feb-22	EPI_ISL_230239	C/Miyagi/11/2002		Import from public-domain	Matsuzaki,Y.; Sugawara,K.; Furuse,Y.; Shimotai,Y.; Hongo,S.; Oshitani,H.; Mizuta,K.; Nishimura,H.
EPI813799	PB1	Japan	2002-Feb-22	EPI_ISL_230239	C/Miyagi/11/2002		Import from public-domain	Matsuzaki,Y.; Sugawara,K.; Furuse,Y.; Shimotai,Y.; Hongo,S.; Oshitani,H.; Mizuta,K.; Nishimura,H.
EPI813702	PB2	Japan	2002-Feb-22	EPI_ISL_230239	C/Miyagi/11/2002		Import from public-domain	Matsuzaki,Y.; Sugawara,K.; Furuse,Y.; Shimotai,Y.; Hongo,S.; Oshitani,H.; Mizuta,K.; Nishimura,H.
EPI228322	HE	Japan	2004-Feb-27	EPI_ISL_65158	C/Miyagi/12/2004		Import from public-domain	Matsuzaki,Y.; Sugawara,K.; Furuse,Y.; Shimotai,Y.; Hongo,S.; Oshitani,H.; Mizuta,K.; Nishimura,H.
EPI816689	MP	Japan	2004-Feb-27	EPI_ISL_65158	C/Miyagi/12/2004		Import from public-domain	Matsuzaki,Y.; Sugawara,K.; Furuse,Y.; Shimotai,Y.; Hongo,S.; Oshitani,H.; Mizuta,K.; Nishimura,H.
EPI816624	NP	Japan	2004-Feb-27	EPI_ISL_65158	C/Miyagi/12/2004		Import from public-domain	Matsuzaki,Y.; Sugawara,K.; Furuse,Y.; Shimotai,Y.; Hongo,S.; Oshitani,H.; Mizuta,K.; Nishimura,H.
EPI816758	NS	Japan	2004-Feb-27	EPI_ISL_65158	C/Miyagi/12/2004		Import from public-domain	Matsuzaki,Y.; Sugawara,K.; Furuse,Y.; Shimotai,Y.; Hongo,S.; Oshitani,H.; Mizuta,K.; Nishimura,H.
EPI816522	P3	Japan	2004-Feb-27	EPI_ISL_65158	C/Miyagi/12/2004		Import from public-domain	Matsuzaki,Y.; Sugawara,K.; Furuse,Y.; Shimotai,Y.; Hongo,S.; Oshitani,H.; Mizuta,K.; Nishimura,H.
EPI816429	PB1	Japan	2004-Feb-27	EPI_ISL_65158	C/Miyagi/12/2004		Import from public-domain	Matsuzaki,Y.; Sugawara,K.; Furuse,Y.; Shimotai,Y.; Hongo,S.; Oshitani,H.; Mizuta,K.; Nishimura,H.
EPI813733	PB2	Japan	2004-Feb-27	EPI_ISL_65158	C/Miyagi/12/2004		Import from public-domain	Matsuzaki,Y.; Sugawara,K.; Furuse,Y.; Shimotai,Y.; Hongo,S.; Oshitani,H.; Mizuta,K.; Nishimura,H.
EPI231696	HE	Japan	2000-Jan-01	EPI_ISL_66382	C/Miyagi/2/2000		Import from public-domain	Matsuzaki,Y.
EPI231694	MP	Japan	2000-Jan-01	EPI_ISL_66382	C/Miyagi/2/2000		Import from public-domain	Matsuzaki,Y.
EPI231695	NS	Japan	2000-Jan-01	EPI_ISL_66382	C/Miyagi/2/2000		Import from public-domain	Matsuzaki,Y.
EPI813665	HE	Japan	2005-Apr-07	EPI_ISL_230250	C/Miyagi/2/2005		Import from public-domain	Matsuzaki,Y.; Sugawara,K.; Furuse,Y.; Shimotai,Y.; Hongo,S.; Oshitani,H.; Mizuta,K.; Nishimura,H.
EPI816696	MP	Japan	2005-Apr-07	EPI_ISL_230250	C/Miyagi/2/2005		Import from public-domain	Matsuzaki,Y.; Sugawara,K.; Furuse,Y.; Shimotai,Y.; Hongo,S.; Oshitani,H.; Mizuta,K.; Nishimura,H.
EPI816630	NP	Japan	2005-Apr-07	EPI_ISL_230250	C/Miyagi/2/2005		Import from public-domain	Matsuzaki,Y.; Sugawara,K.; Furuse,Y.; Shimotai,Y.; Hongo,S.; Oshitani,H.; Mizuta,K.; Nishimura,H.
EPI816765	NS	Japan	2005-Apr-07	EPI_ISL_230250	C/Miyagi/2/2005		Import from public-domain	Matsuzaki,Y.; Sugawara,K.; Furuse,Y.; Shimotai,Y.; Hongo,S.; Oshitani,H.; Mizuta,K.; Nishimura,H.
EPI816529	P3	Japan	2005-Apr-07	EPI_ISL_230250	C/Miyagi/2/2005		Import from public-domain	Matsuzaki,Y.; Sugawara,K.; Furuse,Y.; Shimotai,Y.; Hongo,S.; Oshitani,H.; Mizuta,K.; Nishimura,H.

Segment ID	Segment	Country	Collection date	Isolate-ID	Isolate name	Originating Lab	Submitting Lab	Authors
EPI813811	PB1	Japan	2005-Apr-07	EPI_ISL_230250	C/Miyagi/2/2005		Import from public-domain	Matsuzaki,Y.; Sugawara,K.; Furuse,Y.; Shimotai,Y.; Hongo,S.; Oshitani,H.; Mizuta,K.; Nishimura,H.
EPI813714	PB2	Japan	2005-Apr-07	EPI_ISL_230250	C/Miyagi/2/2005		Import from public-domain	Matsuzaki,Y.; Sugawara,K.; Furuse,Y.; Shimotai,Y.; Hongo,S.; Oshitani,H.; Mizuta,K.; Nishimura,H.
EPI813678	HE	Japan	2008-Feb-21	EPI_ISL_230263	C/Miyagi/2/2008		Import from public-domain	Matsuzaki,Y.; Sugawara,K.; Furuse,Y.; Shimotai,Y.; Hongo,S.; Oshitani,H.; Mizuta,K.; Nishimura,H.
EPI816711	MP	Japan	2008-Feb-21	EPI_ISL_230263	C/Miyagi/2/2008		Import from public-domain	Matsuzaki,Y.; Sugawara,K.; Furuse,Y.; Shimotai,Y.; Hongo,S.; Oshitani,H.; Mizuta,K.; Nishimura,H.
EPI816644	NP	Japan	2008-Feb-21	EPI_ISL_230263	C/Miyagi/2/2008		Import from public-domain	Matsuzaki,Y.; Sugawara,K.; Furuse,Y.; Shimotai,Y.; Hongo,S.; Oshitani,H.; Mizuta,K.; Nishimura,H.
EPI816780	NS	Japan	2008-Feb-21	EPI_ISL_230263	C/Miyagi/2/2008		Import from public-domain	Matsuzaki,Y.; Sugawara,K.; Furuse,Y.; Shimotai,Y.; Hongo,S.; Oshitani,H.; Mizuta,K.; Nishimura,H.
EPI816544	P3	Japan	2008-Feb-21	EPI_ISL_230263	C/Miyagi/2/2008		Import from public-domain	Matsuzaki,Y.; Sugawara,K.; Furuse,Y.; Shimotai,Y.; Hongo,S.; Oshitani,H.; Mizuta,K.; Nishimura,H.
EPI816440	PB1	Japan	2008-Feb-21	EPI_ISL_230263	C/Miyagi/2/2008		Import from public-domain	Matsuzaki,Y.; Sugawara,K.; Furuse,Y.; Shimotai,Y.; Hongo,S.; Oshitani,H.; Mizuta,K.; Nishimura,H.
EPI813750	PB2	Japan	2008-Feb-21	EPI_ISL_230263	C/Miyagi/2/2008		Import from public-domain	Matsuzaki,Y.; Sugawara,K.; Furuse,Y.; Shimotai,Y.; Hongo,S.; Oshitani,H.; Mizuta,K.; Nishimura,H.
EPI813686	HE	Japan	2012-Jan-26	EPI_ISL_230271	C/Miyagi/2/2012		Import from public-domain	Matsuzaki,Y.; Sugawara,K.; Furuse,Y.; Shimotai,Y.; Hongo,S.; Oshitani,H.; Mizuta,K.; Nishimura,H.
EPI816722	MP	Japan	2012-Jan-26	EPI_ISL_230271	C/Miyagi/2/2012		Import from public-domain	Matsuzaki,Y.; Sugawara,K.; Furuse,Y.; Shimotai,Y.; Hongo,S.; Oshitani,H.; Mizuta,K.; Nishimura,H.
EPI816654	NP	Japan	2012-Jan-26	EPI_ISL_230271	C/Miyagi/2/2012		Import from public-domain	Matsuzaki,Y.; Sugawara,K.; Furuse,Y.; Shimotai,Y.; Hongo,S.; Oshitani,H.; Mizuta,K.; Nishimura,H.
EPI816791	NS	Japan	2012-Jan-26	EPI_ISL_230271	C/Miyagi/2/2012		Import from public-domain	Matsuzaki,Y.; Sugawara,K.; Furuse,Y.; Shimotai,Y.; Hongo,S.; Oshitani,H.; Mizuta,K.; Nishimura,H.
EPI816555	P3	Japan	2012-Jan-26	EPI_ISL_230271	C/Miyagi/2/2012		Import from public-domain	Matsuzaki,Y.; Sugawara,K.; Furuse,Y.; Shimotai,Y.; Hongo,S.; Oshitani,H.; Mizuta,K.; Nishimura,H.
EPI816451	PB1	Japan	2012-Jan-26	EPI_ISL_230271	C/Miyagi/2/2012		Import from public-domain	Matsuzaki,Y.; Sugawara,K.; Furuse,Y.; Shimotai,Y.; Hongo,S.; Oshitani,H.; Mizuta,K.; Nishimura,H.
EPI813761	PB2	Japan	2012-Jan-26	EPI_ISL_230271	C/Miyagi/2/2012		Import from public-domain	Matsuzaki,Y.; Sugawara,K.; Furuse,Y.; Shimotai,Y.; Hongo,S.; Oshitani,H.; Mizuta,K.; Nishimura,H.
EPI813688	HE	Japan	2014-Apr-30	EPI_ISL_230273	C/Miyagi/2/2014		Import from public-domain	Matsuzaki,Y.; Sugawara,K.; Furuse,Y.; Shimotai,Y.; Hongo,S.; Oshitani,H.; Mizuta,K.; Nishimura,H.
EPI816731	MP	Japan	2014-Apr-30	EPI_ISL_230273	C/Miyagi/2/2014		Import from public-domain	Matsuzaki,Y.; Sugawara,K.; Furuse,Y.; Shimotai,Y.; Hongo,S.; Oshitani,H.; Mizuta,K.; Nishimura,H.
EPI816663	NP	Japan	2014-Apr-30	EPI_ISL_230273	C/Miyagi/2/2014		Import from public-domain	Matsuzaki,Y.; Sugawara,K.; Furuse,Y.; Shimotai,Y.; Hongo,S.; Oshitani,H.; Mizuta,K.; Nishimura,H.
EPI816800	NS	Japan	2014-Apr-30	EPI_ISL_230273	C/Miyagi/2/2014		Import from public-domain	Matsuzaki,Y.; Sugawara,K.; Furuse,Y.; Shimotai,Y.; Hongo,S.; Oshitani,H.; Mizuta,K.; Nishimura,H.
EPI816564	P3	Japan	2014-Apr-30	EPI_ISL_230273	C/Miyagi/2/2014		Import from public-domain	Matsuzaki,Y.; Sugawara,K.; Furuse,Y.; Shimotai,Y.; Hongo,S.; Oshitani,H.; Mizuta,K.; Nishimura,H.
EPI816453	PB1	Japan	2014-Apr-30	EPI_ISL_230273	C/Miyagi/2/2014		Import from public-domain	Matsuzaki,Y.; Sugawara,K.; Furuse,Y.; Shimotai,Y.; Hongo,S.; Oshitani,H.; Mizuta,K.; Nishimura,H.
EPI813763	PB2	Japan	2014-Apr-30	EPI_ISL_230273	C/Miyagi/2/2014		Import from public-domain	Matsuzaki,Y.; Sugawara,K.; Furuse,Y.; Shimotai,Y.; Hongo,S.; Oshitani,H.; Mizuta,K.; Nishimura,H.
EPI232105	HE	Japan	1992-Jan-01	EPI_ISL_66383	C/Miyagi/2/92		Import from public-domain	Matsuzaki,Y.
EPI232104	MP	Japan	1992-Jan-01	EPI_ISL_66383	C/Miyagi/2/92		Import from public-domain	Matsuzaki,Y.
EPI231702	NS	Japan	1992-Jan-01	EPI_ISL_66383	C/Miyagi/2/92		Import from public-domain	Matsuzaki,Y.
EPI232106	HE	Japan	1993-Jan-01	EPI_ISL_66384	C/Miyagi/2/93		Import from public-domain	Matsuzaki,Y.
EPI231706	MP	Japan	1993-Jan-01	EPI_ISL_66384	C/Miyagi/2/93		Import from public-domain	Matsuzaki,Y.
EPI231705	NS	Japan	1993-Jan-01	EPI_ISL_66384	C/Miyagi/2/93		Import from public-domain	Matsuzaki,Y.
EPI231710	HE	Japan	1994-Jan-01	EPI_ISL_66385	C/Miyagi/2/94		Import from public-domain	Matsuzaki,Y.
EPI231711	MP	Japan	1994-Jan-01	EPI_ISL_66385	C/Miyagi/2/94		Import from public-domain	Matsuzaki,Y.
EPI231715	NS	Japan	1994-Jan-01	EPI_ISL_66385	C/Miyagi/2/94		Import from public-domain	Matsuzaki,Y.
EPI231720	HE	Japan	1996-Jan-01	EPI_ISL_66386	C/Miyagi/2/96		Import from public-domain	Matsuzaki,Y.

Segment ID	Segment	Country	Collection date	Isolate-ID	Isolate name	Originating Lab	Submitting Lab	Authors
EPI231722	MP	Japan	1996-Jan-01	EPI_ISL_66386	C/Miyagi/2/96		Import from public-domain	Matsuzaki,Y.
EPI231716	NS	Japan	1996-Jan-01	EPI_ISL_66386	C/Miyagi/2/96		Import from public-domain	Matsuzaki,Y.
EPI231728	HE	Japan	1998-Jan-01	EPI_ISL_66387	C/Miyagi/2/98		Import from public-domain	Matsuzaki,Y.
EPI231727	MP	Japan	1998-Jan-01	EPI_ISL_66387	C/Miyagi/2/98		Import from public-domain	Matsuzaki,Y.
EPI231723	NS	Japan	1998-Jan-01	EPI_ISL_66387	C/Miyagi/2/98		Import from public-domain	Matsuzaki,Y.
EPI813655	HE	Japan	2002-Mar-17	EPI_ISL_230240	C/Miyagi/20/2002		Import from public-domain	Matsuzaki,Y.; Sugawara,K.; Furuse,Y.; Shimotai,Y.; Hongo,S.; Oshitani,H.; Mizuta,K.; Nishimura,H.
EPI816680	MP	Japan	2002-Mar-17	EPI_ISL_230240	C/Miyagi/20/2002		Import from public-domain	Matsuzaki,Y.; Sugawara,K.; Furuse,Y.; Shimotai,Y.; Hongo,S.; Oshitani,H.; Mizuta,K.; Nishimura,H.
EPI816616	NP	Japan	2002-Mar-17	EPI_ISL_230240	C/Miyagi/20/2002		Import from public-domain	Matsuzaki,Y.; Sugawara,K.; Furuse,Y.; Shimotai,Y.; Hongo,S.; Oshitani,H.; Mizuta,K.; Nishimura,H.
EPI816749	NS	Japan	2002-Mar-17	EPI_ISL_230240	C/Miyagi/20/2002		Import from public-domain	Matsuzaki,Y.; Sugawara,K.; Furuse,Y.; Shimotai,Y.; Hongo,S.; Oshitani,H.; Mizuta,K.; Nishimura,H.
EPI816513	P3	Japan	2002-Mar-17	EPI_ISL_230240	C/Miyagi/20/2002		Import from public-domain	Matsuzaki,Y.; Sugawara,K.; Furuse,Y.; Shimotai,Y.; Hongo,S.; Oshitani,H.; Mizuta,K.; Nishimura,H.
EPI813800	PB1	Japan	2002-Mar-17	EPI_ISL_230240	C/Miyagi/20/2002		Import from public-domain	Matsuzaki,Y.; Sugawara,K.; Furuse,Y.; Shimotai,Y.; Hongo,S.; Oshitani,H.; Mizuta,K.; Nishimura,H.
EPI813703	PB2	Japan	2002-Mar-17	EPI_ISL_230240	C/Miyagi/20/2002		Import from public-domain	Matsuzaki,Y.; Sugawara,K.; Furuse,Y.; Shimotai,Y.; Hongo,S.; Oshitani,H.; Mizuta,K.; Nishimura,H.
EPI813660	HE	Japan	2004-Mar-31	EPI_ISL_230245	C/Miyagi/25/2004		Import from public-domain	Matsuzaki,Y.; Sugawara,K.; Furuse,Y.; Shimotai,Y.; Hongo,S.; Oshitani,H.; Mizuta,K.; Nishimura,H.
EPI816690	MP	Japan	2004-Mar-31	EPI_ISL_230245	C/Miyagi/25/2004		Import from public-domain	Matsuzaki,Y.; Sugawara,K.; Furuse,Y.; Shimotai,Y.; Hongo,S.; Oshitani,H.; Mizuta,K.; Nishimura,H.
EPI816625	NP	Japan	2004-Mar-31	EPI_ISL_230245	C/Miyagi/25/2004		Import from public-domain	Matsuzaki,Y.; Sugawara,K.; Furuse,Y.; Shimotai,Y.; Hongo,S.; Oshitani,H.; Mizuta,K.; Nishimura,H.
EPI816759	NS	Japan	2004-Mar-31	EPI_ISL_230245	C/Miyagi/25/2004		Import from public-domain	Matsuzaki,Y.; Sugawara,K.; Furuse,Y.; Shimotai,Y.; Hongo,S.; Oshitani,H.; Mizuta,K.; Nishimura,H.
EPI816523	P3	Japan	2004-Mar-31	EPI_ISL_230245	C/Miyagi/25/2004		Import from public-domain	Matsuzaki,Y.; Sugawara,K.; Furuse,Y.; Shimotai,Y.; Hongo,S.; Oshitani,H.; Mizuta,K.; Nishimura,H.
EPI813806	PB1	Japan	2004-Mar-31	EPI_ISL_230245	C/Miyagi/25/2004		Import from public-domain	Matsuzaki,Y.; Sugawara,K.; Furuse,Y.; Shimotai,Y.; Hongo,S.; Oshitani,H.; Mizuta,K.; Nishimura,H.
EPI813709	PB2	Japan	2004-Mar-31	EPI_ISL_230245	C/Miyagi/25/2004		Import from public-domain	Matsuzaki,Y.; Sugawara,K.; Furuse,Y.; Shimotai,Y.; Hongo,S.; Oshitani,H.; Mizuta,K.; Nishimura,H.
EPI813656	HE	Japan	2002-Apr-22	EPI_ISL_230241	C/Miyagi/28/2002		Import from public-domain	Matsuzaki,Y.; Sugawara,K.; Furuse,Y.; Shimotai,Y.; Hongo,S.; Oshitani,H.; Mizuta,K.; Nishimura,H.
EPI816681	MP	Japan	2002-Apr-22	EPI_ISL_230241	C/Miyagi/28/2002		Import from public-domain	Matsuzaki,Y.; Sugawara,K.; Furuse,Y.; Shimotai,Y.; Hongo,S.; Oshitani,H.; Mizuta,K.; Nishimura,H.
EPI816617	NP	Japan	2002-Apr-22	EPI_ISL_230241	C/Miyagi/28/2002		Import from public-domain	Matsuzaki,Y.; Sugawara,K.; Furuse,Y.; Shimotai,Y.; Hongo,S.; Oshitani,H.; Mizuta,K.; Nishimura,H.
EPI816750	NS	Japan	2002-Apr-22	EPI_ISL_230241	C/Miyagi/28/2002		Import from public-domain	Matsuzaki,Y.; Sugawara,K.; Furuse,Y.; Shimotai,Y.; Hongo,S.; Oshitani,H.; Mizuta,K.; Nishimura,H.
EPI816514	P3	Japan	2002-Apr-22	EPI_ISL_230241	C/Miyagi/28/2002		Import from public-domain	Matsuzaki,Y.; Sugawara,K.; Furuse,Y.; Shimotai,Y.; Hongo,S.; Oshitani,H.; Mizuta,K.; Nishimura,H.
EPI813801	PB1	Japan	2002-Apr-22	EPI_ISL_230241	C/Miyagi/28/2002		Import from public-domain	Matsuzaki,Y.; Sugawara,K.; Furuse,Y.; Shimotai,Y.; Hongo,S.; Oshitani,H.; Mizuta,K.; Nishimura,H.
EPI813704	PB2	Japan	2002-Apr-22	EPI_ISL_230241	C/Miyagi/28/2002		Import from public-domain	Matsuzaki,Y.; Sugawara,K.; Furuse,Y.; Shimotai,Y.; Hongo,S.; Oshitani,H.; Mizuta,K.; Nishimura,H.
EPI231730	HE	Japan	2000-Jan-01	EPI_ISL_66388	C/Miyagi/3/2000		Import from public-domain	Matsuzaki,Y.
EPI231736	MP	Japan	2000-Jan-01	EPI_ISL_66388	C/Miyagi/3/2000		Import from public-domain	Matsuzaki,Y.
EPI231735	NS	Japan	2000-Jan-01	EPI_ISL_66388	C/Miyagi/3/2000		Import from public-domain	Matsuzaki,Y.
EPI813679	HE	Japan	2010-Apr-12	EPI_ISL_230264	C/Miyagi/3/2010		Import from public-domain	Matsuzaki,Y.; Sugawara,K.; Furuse,Y.; Shimotai,Y.; Hongo,S.; Oshitani,H.; Mizuta,K.; Nishimura,H.
EPI816713	MP	Japan	2010-Apr-12	EPI_ISL_230264	C/Miyagi/3/2010		Import from public-domain	Matsuzaki,Y.; Sugawara,K.; Furuse,Y.; Shimotai,Y.; Hongo,S.; Oshitani,H.; Mizuta,K.; Nishimura,H.
EPI816645	NP	Japan	2010-Apr-12	EPI_ISL_230264	C/Miyagi/3/2010		Import from public-domain	Matsuzaki,Y.; Sugawara,K.; Furuse,Y.; Shimotai,Y.; Hongo,S.; Oshitani,H.; Mizuta,K.; Nishimura,H.
EPI816782	NS	Japan	2010-Apr-12	EPI_ISL_230264	C/Miyagi/3/2010		Import from public-domain	Matsuzaki,Y.; Sugawara,K.; Furuse,Y.; Shimotai,Y.; Hongo,S.; Oshitani,H.; Mizuta,K.; Nishimura,H.

Segment ID	Segment	Country	Collection date	Isolate-ID	Isolate name	Originating Lab	Submitting Lab	Authors
EPI816546	P3	Japan	2010-Apr-12	EPI_ISL_230264	C/Miyagi/3/2010		Import from public-domain	Matsuzaki,Y.; Sugawara,K.; Furuse,Y.; Shimotai,Y.; Hongo,S.; Oshitani,H.; Mizuta,K.; Nishimura,H.
EPI816442	PB1	Japan	2010-Apr-12	EPI_ISL_230264	C/Miyagi/3/2010		Import from public-domain	Matsuzaki,Y.; Sugawara,K.; Furuse,Y.; Shimotai,Y.; Hongo,S.; Oshitani,H.; Mizuta,K.; Nishimura,H.
EPI813752	PB2	Japan	2010-Apr-12	EPI_ISL_230264	C/Miyagi/3/2010		Import from public-domain	Matsuzaki,Y.; Sugawara,K.; Furuse,Y.; Shimotai,Y.; Hongo,S.; Oshitani,H.; Mizuta,K.; Nishimura,H.
EPI231737	HE	Japan	1991-Jan-01	EPI_ISL_66389	C/Miyagi/3/91		Import from public-domain	Kimura,H.
EPI231744	HE	Japan	1992-Jan-01	EPI_ISL_66390	C/Miyagi/3/92		Import from public-domain	Matsuzaki,Y.
EPI231747	HE	Japan	1993-Apr-13	EPI_ISL_66391	C/Miyagi/3/93		Import from public-domain	Matsuzaki,Y.; Sugawara,K.; Furuse,Y.; Shimotai,Y.; Hongo,S.; Oshitani,H.; Mizuta,K.; Nishimura,H.
EPI231750	MP	Japan	1993-Apr-13	EPI_ISL_66391	C/Miyagi/3/93		Import from public-domain	Matsuzaki,Y.; Sugawara,K.; Furuse,Y.; Shimotai,Y.; Hongo,S.; Oshitani,H.; Mizuta,K.; Nishimura,H.
EPI816605	NP	Japan	1993-Apr-13	EPI_ISL_66391	C/Miyagi/3/93		Import from public-domain	Matsuzaki,Y.; Sugawara,K.; Furuse,Y.; Shimotai,Y.; Hongo,S.; Oshitani,H.; Mizuta,K.; Nishimura,H.
EPI231751	NS	Japan	1993-Apr-13	EPI_ISL_66391	C/Miyagi/3/93		Import from public-domain	Matsuzaki,Y.; Sugawara,K.; Furuse,Y.; Shimotai,Y.; Hongo,S.; Oshitani,H.; Mizuta,K.; Nishimura,H.
EPI816500	P3	Japan	1993-Apr-13	EPI_ISL_66391	C/Miyagi/3/93		Import from public-domain	Matsuzaki,Y.; Sugawara,K.; Furuse,Y.; Shimotai,Y.; Hongo,S.; Oshitani,H.; Mizuta,K.; Nishimura,H.
EPI816417	PB1	Japan	1993-Apr-13	EPI_ISL_66391	C/Miyagi/3/93		Import from public-domain	Matsuzaki,Y.; Sugawara,K.; Furuse,Y.; Shimotai,Y.; Hongo,S.; Oshitani,H.; Mizuta,K.; Nishimura,H.
EPI813729	PB2	Japan	1993-Apr-13	EPI_ISL_66391	C/Miyagi/3/93		Import from public-domain	Matsuzaki,Y.; Sugawara,K.; Furuse,Y.; Shimotai,Y.; Hongo,S.; Oshitani,H.; Mizuta,K.; Nishimura,H.
EPI231756	HE	Japan	1994-Jan-01	EPI_ISL_66392	C/Miyagi/3/94		Import from public-domain	Matsuzaki,Y.
EPI231755	MP	Japan	1994-Jan-01	EPI_ISL_66392	C/Miyagi/3/94		Import from public-domain	Matsuzaki,Y.
EPI231758	NS	Japan	1994-Jan-01	EPI_ISL_66392	C/Miyagi/3/94		Import from public-domain	Matsuzaki,Y.
EPI231759	HE	Japan	1997-Jan-01	EPI_ISL_66393	C/Miyagi/3/97		Import from public-domain	Matsuzaki,Y.
EPI231764	HE	Japan	1999-Jan-01	EPI_ISL_66394	C/Miyagi/3/99		Import from public-domain	Matsuzaki,Y.
EPI231761	MP	Japan	1999-Jan-01	EPI_ISL_66394	C/Miyagi/3/99		Import from public-domain	Matsuzaki,Y.
EPI231760	NS	Japan	1999-Jan-01	EPI_ISL_66394	C/Miyagi/3/99		Import from public-domain	Matsuzaki,Y.
EPI813657	HE	Japan	2002-Jun-18	EPI_ISL_230242	C/Miyagi/31/2002		Import from public-domain	Matsuzaki,Y.; Sugawara,K.; Furuse,Y.; Shimotai,Y.; Hongo,S.; Oshitani,H.; Mizuta,K.; Nishimura,H.
EPI816682	MP	Japan	2002-Jun-18	EPI_ISL_230242	C/Miyagi/31/2002		Import from public-domain	Matsuzaki,Y.; Sugawara,K.; Furuse,Y.; Shimotai,Y.; Hongo,S.; Oshitani,H.; Mizuta,K.; Nishimura,H.
EPI816618	NP	Japan	2002-Jun-18	EPI_ISL_230242	C/Miyagi/31/2002		Import from public-domain	Matsuzaki,Y.; Sugawara,K.; Furuse,Y.; Shimotai,Y.; Hongo,S.; Oshitani,H.; Mizuta,K.; Nishimura,H.
EPI816751	NS	Japan	2002-Jun-18	EPI_ISL_230242	C/Miyagi/31/2002		Import from public-domain	Matsuzaki,Y.; Sugawara,K.; Furuse,Y.; Shimotai,Y.; Hongo,S.; Oshitani,H.; Mizuta,K.; Nishimura,H.
EPI816515	P3	Japan	2002-Jun-18	EPI_ISL_230242	C/Miyagi/31/2002		Import from public-domain	Matsuzaki,Y.; Sugawara,K.; Furuse,Y.; Shimotai,Y.; Hongo,S.; Oshitani,H.; Mizuta,K.; Nishimura,H.
EPI813802	PB1	Japan	2002-Jun-18	EPI_ISL_230242	C/Miyagi/31/2002		Import from public-domain	Matsuzaki,Y.; Sugawara,K.; Furuse,Y.; Shimotai,Y.; Hongo,S.; Oshitani,H.; Mizuta,K.; Nishimura,H.
EPI813705	PB2	Japan	2002-Jun-18	EPI_ISL_230242	C/Miyagi/31/2002		Import from public-domain	Matsuzaki,Y.; Sugawara,K.; Furuse,Y.; Shimotai,Y.; Hongo,S.; Oshitani,H.; Mizuta,K.; Nishimura,H.
EPI231767	HE	Japan	2000-Jan-01	EPI_ISL_66395	C/Miyagi/4/2000		Import from public-domain	Matsuzaki,Y.
EPI231772	MP	Japan	2000-Jan-01	EPI_ISL_66395	C/Miyagi/4/2000		Import from public-domain	Matsuzaki,Y.
EPI231769	NP	Japan	2000-Jan-01	EPI_ISL_66395	C/Miyagi/4/2000		Import from public-domain	Matsuzaki,Y.
EPI231773	NS	Japan	2000-Jan-01	EPI_ISL_66395	C/Miyagi/4/2000		Import from public-domain	Matsuzaki,Y.
EPI231771	PB1	Japan	2000-Jan-01	EPI_ISL_66395	C/Miyagi/4/2000		Import from public-domain	Matsuzaki,Y.
EPI813653	HE	Japan	2002-Jan-24	EPI_ISL_230238	C/Miyagi/4/2002		Import from public-domain	Matsuzaki,Y.; Sugawara,K.; Furuse,Y.; Shimotai,Y.; Hongo,S.; Oshitani,H.; Mizuta,K.; Nishimura,H.
EPI816678	MP	Japan	2002-Jan-24	EPI_ISL_230238	C/Miyagi/4/2002		Import from public-domain	Matsuzaki,Y.; Sugawara,K.; Furuse,Y.; Shimotai,Y.; Hongo,S.; Oshitani,H.; Mizuta,K.; Nishimura,H.

Segment ID	Segment	Country	Collection date	Isolate-ID	Isolate name	Originating Lab	Submitting Lab	Authors
EPI816747	NS	Japan	2002-Jan-24	EPI_ISL_230238	C/Miyagi/4/2002		Import from public-domain	Matsuzaki,Y.; Sugawara,K.; Furuse,Y.; Shimotai,Y.; Hongo,S.; Oshitani,H.; Mizuta,K.; Nishimura,H.
EPI816511	P3	Japan	2002-Jan-24	EPI_ISL_230238	C/Miyagi/4/2002		Import from public-domain	Matsuzaki,Y.; Sugawara,K.; Furuse,Y.; Shimotai,Y.; Hongo,S.; Oshitani,H.; Mizuta,K.; Nishimura,H.
EPI813798	PB1	Japan	2002-Jan-24	EPI_ISL_230238	C/Miyagi/4/2002		Import from public-domain	Matsuzaki,Y.; Sugawara,K.; Furuse,Y.; Shimotai,Y.; Hongo,S.; Oshitani,H.; Mizuta,K.; Nishimura,H.
EPI813701	PB2	Japan	2002-Jan-24	EPI_ISL_230238	C/Miyagi/4/2002		Import from public-domain	Matsuzaki,Y.; Sugawara,K.; Furuse,Y.; Shimotai,Y.; Hongo,S.; Oshitani,H.; Mizuta,K.; Nishimura,H.
EPI813666	HE	Japan	2005-May-16	EPI_ISL_230251	C/Miyagi/4/2005		Import from public-domain	Matsuzaki,Y.; Sugawara,K.; Furuse,Y.; Shimotai,Y.; Hongo,S.; Oshitani,H.; Mizuta,K.; Nishimura,H.
EPI816697	MP	Japan	2005-May-16	EPI_ISL_230251	C/Miyagi/4/2005		Import from public-domain	Matsuzaki,Y.; Sugawara,K.; Furuse,Y.; Shimotai,Y.; Hongo,S.; Oshitani,H.; Mizuta,K.; Nishimura,H.
EPI816631	NP	Japan	2005-May-16	EPI_ISL_230251	C/Miyagi/4/2005		Import from public-domain	Matsuzaki,Y.; Sugawara,K.; Furuse,Y.; Shimotai,Y.; Hongo,S.; Oshitani,H.; Mizuta,K.; Nishimura,H.
EPI816766	NS	Japan	2005-May-16	EPI_ISL_230251	C/Miyagi/4/2005		Import from public-domain	Matsuzaki,Y.; Sugawara,K.; Furuse,Y.; Shimotai,Y.; Hongo,S.; Oshitani,H.; Mizuta,K.; Nishimura,H.
EPI816530	P3	Japan	2005-May-16	EPI_ISL_230251	C/Miyagi/4/2005		Import from public-domain	Matsuzaki,Y.; Sugawara,K.; Furuse,Y.; Shimotai,Y.; Hongo,S.; Oshitani,H.; Mizuta,K.; Nishimura,H.
EPI813812	PB1	Japan	2005-May-16	EPI_ISL_230251	C/Miyagi/4/2005		Import from public-domain	Matsuzaki,Y.; Sugawara,K.; Furuse,Y.; Shimotai,Y.; Hongo,S.; Oshitani,H.; Mizuta,K.; Nishimura,H.
EPI813715	PB2	Japan	2005-May-16	EPI_ISL_230251	C/Miyagi/4/2005		Import from public-domain	Matsuzaki,Y.; Sugawara,K.; Furuse,Y.; Shimotai,Y.; Hongo,S.; Oshitani,H.; Mizuta,K.; Nishimura,H.
EPI813673	HE	Japan	2006-Jun-04	EPI_ISL_230258	C/Miyagi/4/2006		Import from public-domain	Matsuzaki,Y.; Sugawara,K.; Furuse,Y.; Shimotai,Y.; Hongo,S.; Oshitani,H.; Mizuta,K.; Nishimura,H.
EPI816705	MP	Japan	2006-Jun-04	EPI_ISL_230258	C/Miyagi/4/2006		Import from public-domain	Matsuzaki,Y.; Sugawara,K.; Furuse,Y.; Shimotai,Y.; Hongo,S.; Oshitani,H.; Mizuta,K.; Nishimura,H.
EPI816639	NP	Japan	2006-Jun-04	EPI_ISL_230258	C/Miyagi/4/2006		Import from public-domain	Matsuzaki,Y.; Sugawara,K.; Furuse,Y.; Shimotai,Y.; Hongo,S.; Oshitani,H.; Mizuta,K.; Nishimura,H.
EPI816774	NS	Japan	2006-Jun-04	EPI_ISL_230258	C/Miyagi/4/2006		Import from public-domain	Matsuzaki,Y.; Sugawara,K.; Furuse,Y.; Shimotai,Y.; Hongo,S.; Oshitani,H.; Mizuta,K.; Nishimura,H.
EPI816538	P3	Japan	2006-Jun-04	EPI_ISL_230258	C/Miyagi/4/2006		Import from public-domain	Matsuzaki,Y.; Sugawara,K.; Furuse,Y.; Shimotai,Y.; Hongo,S.; Oshitani,H.; Mizuta,K.; Nishimura,H.
EPI816435	PB1	Japan	2006-Jun-04	EPI_ISL_230258	C/Miyagi/4/2006		Import from public-domain	Matsuzaki,Y.; Sugawara,K.; Furuse,Y.; Shimotai,Y.; Hongo,S.; Oshitani,H.; Mizuta,K.; Nishimura,H.
EPI813745	PB2	Japan	2006-Jun-04	EPI_ISL_230258	C/Miyagi/4/2006		Import from public-domain	Matsuzaki,Y.; Sugawara,K.; Furuse,Y.; Shimotai,Y.; Hongo,S.; Oshitani,H.; Mizuta,K.; Nishimura,H.
EPI231778	HE	Japan	1993-Jan-01	EPI_ISL_66396	C/Miyagi/4/93		Import from public-domain	Matsuzaki,Y.
EPI231775	MP	Japan	1993-Jan-01	EPI_ISL_66396	C/Miyagi/4/93		Import from public-domain	Matsuzaki,Y.
EPI231774	NS	Japan	1993-Jan-01	EPI_ISL_66396	C/Miyagi/4/93		Import from public-domain	Matsuzaki,Y.
EPI231785	HE	Japan	1996-Jan-01	EPI_ISL_66397	C/Miyagi/4/96		Import from public-domain	Matsuzaki,Y.
EPI231782	MP	Japan	1996-Jan-01	EPI_ISL_66397	C/Miyagi/4/96		Import from public-domain	Matsuzaki,Y.
EPI231781	NS	Japan	1996-Jan-01	EPI_ISL_66397	C/Miyagi/4/96		Import from public-domain	Matsuzaki,Y.
EPI231788	HE	Japan	1997-Jan-01	EPI_ISL_66398	C/Miyagi/4/97		Import from public-domain	Matsuzaki,Y.
EPI231795	HE	Japan	1998-Jan-01	EPI_ISL_66399	C/Miyagi/4/98		Import from public-domain	Matsuzaki,Y.
EPI231790	MP	Japan	1998-Jan-01	EPI_ISL_66399	C/Miyagi/4/98		Import from public-domain	Matsuzaki,Y.
EPI231789	NS	Japan	1998-Jan-01	EPI_ISL_66399	C/Miyagi/4/98		Import from public-domain	Matsuzaki,Y.
EPI228323	HE	Japan	2004-May-27	EPI_ISL_65159	C/Miyagi/42/2004		Import from public-domain	Matsuzaki,Y.; Sugawara,K.; Furuse,Y.; Shimotai,Y.; Hongo,S.; Oshitani,H.; Mizuta,K.; Nishimura,H.
EPI816691	MP	Japan	2004-May-27	EPI_ISL_65159	C/Miyagi/42/2004		Import from public-domain	Matsuzaki,Y.; Sugawara,K.; Furuse,Y.; Shimotai,Y.; Hongo,S.; Oshitani,H.; Mizuta,K.; Nishimura,H.
EPI816626	NP	Japan	2004-May-27	EPI_ISL_65159	C/Miyagi/42/2004		Import from public-domain	Matsuzaki,Y.; Sugawara,K.; Furuse,Y.; Shimotai,Y.; Hongo,S.; Oshitani,H.; Mizuta,K.; Nishimura,H.
EPI816760	NS	Japan	2004-May-27	EPI_ISL_65159	C/Miyagi/42/2004		Import from public-domain	Matsuzaki,Y.; Sugawara,K.; Furuse,Y.; Shimotai,Y.; Hongo,S.; Oshitani,H.; Mizuta,K.; Nishimura,H.
EPI816524	P3	Japan	2004-May-27	EPI_ISL_65159	C/Miyagi/42/2004		Import from public-domain	Matsuzaki,Y.; Sugawara,K.; Furuse,Y.; Shimotai,Y.; Hongo,S.; Oshitani,H.; Mizuta,K.; Nishimura,H.

Segment ID	Segment	Country	Collection date	Isolate-ID	Isolate name	Originating Lab	Submitting Lab	Authors
EPI816430	PB1	Japan	2004-May-27	EPI_ISL_65159	C/Miyagi/42/2004		Import from public-domain	Matsuzaki,Y.; Sugawara,K.; Furuse,Y.; Shimotai,Y.; Hongo,S.; Oshitani,H.; Mizuta,K.; Nishimura,H.
EPI813726	PB2	Japan	2004-May-27	EPI_ISL_65159	C/Miyagi/42/2004		Import from public-domain	Matsuzaki,Y.; Sugawara,K.; Furuse,Y.; Shimotai,Y.; Hongo,S.; Oshitani,H.; Mizuta,K.; Nishimura,H.
EPI813661	HE	Japan	2004-Jun-18	EPI_ISL_230246	C/Miyagi/47/2004		Import from public-domain	Matsuzaki,Y.; Sugawara,K.; Furuse,Y.; Shimotai,Y.; Hongo,S.; Oshitani,H.; Mizuta,K.; Nishimura,H.
EPI816692	MP	Japan	2004-Jun-18	EPI_ISL_230246	C/Miyagi/47/2004		Import from public-domain	Matsuzaki,Y.; Sugawara,K.; Furuse,Y.; Shimotai,Y.; Hongo,S.; Oshitani,H.; Mizuta,K.; Nishimura,H.
EPI816627	NP	Japan	2004-Jun-18	EPI_ISL_230246	C/Miyagi/47/2004		Import from public-domain	Matsuzaki,Y.; Sugawara,K.; Furuse,Y.; Shimotai,Y.; Hongo,S.; Oshitani,H.; Mizuta,K.; Nishimura,H.
EPI816761	NS	Japan	2004-Jun-18	EPI_ISL_230246	C/Miyagi/47/2004		Import from public-domain	Matsuzaki,Y.; Sugawara,K.; Furuse,Y.; Shimotai,Y.; Hongo,S.; Oshitani,H.; Mizuta,K.; Nishimura,H.
EPI816525	P3	Japan	2004-Jun-18	EPI_ISL_230246	C/Miyagi/47/2004		Import from public-domain	Matsuzaki,Y.; Sugawara,K.; Furuse,Y.; Shimotai,Y.; Hongo,S.; Oshitani,H.; Mizuta,K.; Nishimura,H.
EPI813807	PB1	Japan	2004-Jun-18	EPI_ISL_230246	C/Miyagi/47/2004		Import from public-domain	Matsuzaki,Y.; Sugawara,K.; Furuse,Y.; Shimotai,Y.; Hongo,S.; Oshitani,H.; Mizuta,K.; Nishimura,H.
EPI813710	PB2	Japan	2004-Jun-18	EPI_ISL_230246	C/Miyagi/47/2004		Import from public-domain	Matsuzaki,Y.; Sugawara,K.; Furuse,Y.; Shimotai,Y.; Hongo,S.; Oshitani,H.; Mizuta,K.; Nishimura,H.
EPI231798	HE	Japan	2000-Jan-01	EPI_ISL_66400	C/Miyagi/5/2000		Import from public-domain	Matsuzaki,Y.
EPI231797	MP	Japan	2000-Jan-01	EPI_ISL_66400	C/Miyagi/5/2000		Import from public-domain	Matsuzaki,Y.
EPI231796	NS	Japan	2000-Jan-01	EPI_ISL_66400	C/Miyagi/5/2000		Import from public-domain	Matsuzaki,Y.
EPI813689	HE	Japan	2014-May-17	EPI_ISL_230274	C/Miyagi/5/2014		Import from public-domain	Matsuzaki,Y.; Sugawara,K.; Furuse,Y.; Shimotai,Y.; Hongo,S.; Oshitani,H.; Mizuta,K.; Nishimura,H.
EPI816732	MP	Japan	2014-May-17	EPI_ISL_230274	C/Miyagi/5/2014		Import from public-domain	Matsuzaki,Y.; Sugawara,K.; Furuse,Y.; Shimotai,Y.; Hongo,S.; Oshitani,H.; Mizuta,K.; Nishimura,H.
EPI816664	NP	Japan	2014-May-17	EPI_ISL_230274	C/Miyagi/5/2014		Import from public-domain	Matsuzaki,Y.; Sugawara,K.; Furuse,Y.; Shimotai,Y.; Hongo,S.; Oshitani,H.; Mizuta,K.; Nishimura,H.
EPI816801	NS	Japan	2014-May-17	EPI_ISL_230274	C/Miyagi/5/2014		Import from public-domain	Matsuzaki,Y.; Sugawara,K.; Furuse,Y.; Shimotai,Y.; Hongo,S.; Oshitani,H.; Mizuta,K.; Nishimura,H.
EPI816565	P3	Japan	2014-May-17	EPI_ISL_230274	C/Miyagi/5/2014		Import from public-domain	Matsuzaki,Y.; Sugawara,K.; Furuse,Y.; Shimotai,Y.; Hongo,S.; Oshitani,H.; Mizuta,K.; Nishimura,H.
EPI816454	PB1	Japan	2014-May-17	EPI_ISL_230274	C/Miyagi/5/2014		Import from public-domain	Matsuzaki,Y.; Sugawara,K.; Furuse,Y.; Shimotai,Y.; Hongo,S.; Oshitani,H.; Mizuta,K.; Nishimura,H.
EPI813764	PB2	Japan	2014-May-17	EPI_ISL_230274	C/Miyagi/5/2014		Import from public-domain	Matsuzaki,Y.; Sugawara,K.; Furuse,Y.; Shimotai,Y.; Hongo,S.; Oshitani,H.; Mizuta,K.; Nishimura,H.
EPI231808	HE	Japan	1991-Feb-25	EPI_ISL_66356	C/Miyagi/5/91		Import from public-domain	Matsuzaki,Y.; Sugawara,K.; Furuse,Y.; Shimotai,Y.; Hongo,S.; Oshitani,H.; Mizuta,K.; Nishimura,H.
EPI231804	MP	Japan	1991-Feb-25	EPI_ISL_66356	C/Miyagi/5/91		Import from public-domain	Matsuzaki,Y.; Sugawara,K.; Furuse,Y.; Shimotai,Y.; Hongo,S.; Oshitani,H.; Mizuta,K.; Nishimura,H.
EPI816594	NP	Japan	1991-Feb-25	EPI_ISL_66356	C/Miyagi/5/91		Import from public-domain	Matsuzaki,Y.; Sugawara,K.; Furuse,Y.; Shimotai,Y.; Hongo,S.; Oshitani,H.; Mizuta,K.; Nishimura,H.
EPI231567	NS	Japan	1991-Feb-25	EPI_ISL_66356	C/Miyagi/5/91		Import from public-domain	Matsuzaki,Y.; Sugawara,K.; Furuse,Y.; Shimotai,Y.; Hongo,S.; Oshitani,H.; Mizuta,K.; Nishimura,H.
EPI816495	P3	Japan	1991-Feb-25	EPI_ISL_66356	C/Miyagi/5/91		Import from public-domain	Matsuzaki,Y.; Sugawara,K.; Furuse,Y.; Shimotai,Y.; Hongo,S.; Oshitani,H.; Mizuta,K.; Nishimura,H.
EPI816413	PB1	Japan	1991-Feb-25	EPI_ISL_66356	C/Miyagi/5/91		Import from public-domain	Matsuzaki,Y.; Sugawara,K.; Furuse,Y.; Shimotai,Y.; Hongo,S.; Oshitani,H.; Mizuta,K.; Nishimura,H.
EPI813727	PB2	Japan	1991-Feb-25	EPI_ISL_66356	C/Miyagi/5/91		Import from public-domain	Matsuzaki,Y.; Sugawara,K.; Furuse,Y.; Shimotai,Y.; Hongo,S.; Oshitani,H.; Mizuta,K.; Nishimura,H.
EPI231813	HE	Japan	1993-Jan-01	EPI_ISL_66401	C/Miyagi/5/93		Import from public-domain	Matsuzaki,Y.
EPI231811	MP	Japan	1993-Jan-01	EPI_ISL_66401	C/Miyagi/5/93		Import from public-domain	Matsuzaki,Y.
EPI231812	NS	Japan	1993-Jan-01	EPI_ISL_66401	C/Miyagi/5/93		Import from public-domain	Matsuzaki,Y.
EPI813690	HE	Japan	2014-Jun-02	EPI_ISL_230275	C/Miyagi/6/2014		Import from public-domain	Matsuzaki,Y.; Sugawara,K.; Furuse,Y.; Shimotai,Y.; Hongo,S.; Oshitani,H.; Mizuta,K.; Nishimura,H.
EPI816733	MP	Japan	2014-Jun-02	EPI_ISL_230275	C/Miyagi/6/2014		Import from public-domain	Matsuzaki,Y.; Sugawara,K.; Furuse,Y.; Shimotai,Y.; Hongo,S.; Oshitani,H.; Mizuta,K.; Nishimura,H.
EPI816665	NP	Japan	2014-Jun-02	EPI_ISL_230275	C/Miyagi/6/2014		Import from public-domain	Matsuzaki,Y.; Sugawara,K.; Furuse,Y.; Shimotai,Y.; Hongo,S.; Oshitani,H.; Mizuta,K.; Nishimura,H.
EPI816802	NS	Japan	2014-Jun-02	EPI_ISL_230275	C/Miyagi/6/2014		Import from public-domain	Matsuzaki,Y.; Sugawara,K.; Furuse,Y.; Shimotai,Y.; Hongo,S.; Oshitani,H.; Mizuta,K.; Nishimura,H.

Segment ID	Segment	Country	Collection date	Isolate-ID	Isolate name	Originating Lab	Submitting Lab	Authors
EPI816566	P3	Japan	2014-Jun-02	EPI_ISL_230275	C/Miyagi/6/2014		Import from public-domain	Matsuzaki,Y.; Sugawara,K.; Furuse,Y.; Shimotai,Y.; Hongo,S.; Oshitani,H.; Mizuta,K.; Nishimura,H.
EPI816455	PB1	Japan	2014-Jun-02	EPI_ISL_230275	C/Miyagi/6/2014		Import from public-domain	Matsuzaki,Y.; Sugawara,K.; Furuse,Y.; Shimotai,Y.; Hongo,S.; Oshitani,H.; Mizuta,K.; Nishimura,H.
EPI813765	PB2	Japan	2014-Jun-02	EPI_ISL_230275	C/Miyagi/6/2014		Import from public-domain	Matsuzaki,Y.; Sugawara,K.; Furuse,Y.; Shimotai,Y.; Hongo,S.; Oshitani,H.; Mizuta,K.; Nishimura,H.
EPI231822	HE	Japan	1993-Jan-01	EPI_ISL_66403	C/Miyagi/6/93		Import from public-domain	Matsuzaki,Y.
EPI231817	MP	Japan	1993-Jan-01	EPI_ISL_66403	C/Miyagi/6/93		Import from public-domain	Matsuzaki,Y.
EPI231816	NS	Japan	1993-Jan-01	EPI_ISL_66403	C/Miyagi/6/93		Import from public-domain	Matsuzaki,Y.
EPI231829	HE	Japan	1996-Jan-01	EPI_ISL_66404	C/Miyagi/6/96		Import from public-domain	Matsuzaki,Y.
EPI231827	MP	Japan	1996-Jan-01	EPI_ISL_66404	C/Miyagi/6/96		Import from public-domain	Matsuzaki,Y.
EPI231823	NS	Japan	1996-Jan-01	EPI_ISL_66404	C/Miyagi/6/96		Import from public-domain	Matsuzaki,Y.
EPI231830	HE	Japan	1991-Jan-01	EPI_ISL_66357	C/Miyagi/7/91		Import from public-domain	Matsuzaki,Y.
EPI231834	MP	Japan	1991-Jan-01	EPI_ISL_66357	C/Miyagi/7/91		Import from public-domain	Matsuzaki,Y.
EPI231568	NS	Japan	1991-Jan-01	EPI_ISL_66357	C/Miyagi/7/91		Import from public-domain	Matsuzaki,Y.
EPI231836	HE	Japan	1993-May-19	EPI_ISL_66405	C/Miyagi/7/93		Import from public-domain	Matsuzaki,Y.; Sugawara,K.; Furuse,Y.; Shimotai,Y.; Hongo,S.; Oshitani,H.; Mizuta,K.; Nishimura,H.
EPI231841	MP	Japan	1993-May-19	EPI_ISL_66405	C/Miyagi/7/93		Import from public-domain	Matsuzaki,Y.; Sugawara,K.; Furuse,Y.; Shimotai,Y.; Hongo,S.; Oshitani,H.; Mizuta,K.; Nishimura,H.
EPI816606	NP	Japan	1993-May-19	EPI_ISL_66405	C/Miyagi/7/93		Import from public-domain	Matsuzaki,Y.; Sugawara,K.; Furuse,Y.; Shimotai,Y.; Hongo,S.; Oshitani,H.; Mizuta,K.; Nishimura,H.
EPI231842	NS	Japan	1993-May-19	EPI_ISL_66405	C/Miyagi/7/93		Import from public-domain	Matsuzaki,Y.; Sugawara,K.; Furuse,Y.; Shimotai,Y.; Hongo,S.; Oshitani,H.; Mizuta,K.; Nishimura,H.
EPI816501	P3	Japan	1993-May-19	EPI_ISL_66405	C/Miyagi/7/93		Import from public-domain	Matsuzaki,Y.; Sugawara,K.; Furuse,Y.; Shimotai,Y.; Hongo,S.; Oshitani,H.; Mizuta,K.; Nishimura,H.
EPI816418	PB1	Japan	1993-May-19	EPI_ISL_66405	C/Miyagi/7/93		Import from public-domain	Matsuzaki,Y.; Sugawara,K.; Furuse,Y.; Shimotai,Y.; Hongo,S.; Oshitani,H.; Mizuta,K.; Nishimura,H.
EPI813728	PB2	Japan	1993-May-19	EPI_ISL_66405	C/Miyagi/7/93		Import from public-domain	Matsuzaki,Y.; Sugawara,K.; Furuse,Y.; Shimotai,Y.; Hongo,S.; Oshitani,H.; Mizuta,K.; Nishimura,H.
EPI231849	HE	Japan	1996-Jan-01	EPI_ISL_66406	C/Miyagi/7/96		Import from public-domain	Matsuzaki,Y.
EPI231843	MP	Japan	1996-Jan-01	EPI_ISL_66406	C/Miyagi/7/96		Import from public-domain	Matsuzaki,Y.
EPI231846	NS	Japan	1996-Jan-01	EPI_ISL_66406	C/Miyagi/7/96		Import from public-domain	Matsuzaki,Y.
EPI232107	HE	Japan	1977-Jan-01	EPI_ISL_66329	C/Miyagi/77		Import from public-domain	Matsuzaki,Y.; Sugawara,K.; Furuse,Y.; Shimotai,Y.; Hongo,S.; Oshitani,H.; Mizuta,K.; Nishimura,H.
EPI232108	MP	Japan	1977-Jan-01	EPI_ISL_66329	C/Miyagi/77		Import from public-domain	Matsuzaki,Y.; Sugawara,K.; Furuse,Y.; Shimotai,Y.; Hongo,S.; Oshitani,H.; Mizuta,K.; Nishimura,H.
EPI816577	NP	Japan	1977-Jan-01	EPI_ISL_66329	C/Miyagi/77		Import from public-domain	Matsuzaki,Y.; Sugawara,K.; Furuse,Y.; Shimotai,Y.; Hongo,S.; Oshitani,H.; Mizuta,K.; Nishimura,H.
EPI231530	NS	Japan	1977-Jan-01	EPI_ISL_66329	C/Miyagi/77		Import from public-domain	Matsuzaki,Y.; Sugawara,K.; Furuse,Y.; Shimotai,Y.; Hongo,S.; Oshitani,H.; Mizuta,K.; Nishimura,H.
EPI816468	P3	Japan	1977-Jan-01	EPI_ISL_66329	C/Miyagi/77		Import from public-domain	Matsuzaki,Y.; Sugawara,K.; Furuse,Y.; Shimotai,Y.; Hongo,S.; Oshitani,H.; Mizuta,K.; Nishimura,H.
EPI813781	PB1	Japan	1977-Jan-01	EPI_ISL_66329	C/Miyagi/77		Import from public-domain	Matsuzaki,Y.; Sugawara,K.; Furuse,Y.; Shimotai,Y.; Hongo,S.; Oshitani,H.; Mizuta,K.; Nishimura,H.
EPI814971	PB2	Japan	1977-Jan-01	EPI_ISL_66329	C/Miyagi/77		Import from public-domain	Matsuzaki,Y.; Sugawara,K.; Furuse,Y.; Shimotai,Y.; Hongo,S.; Oshitani,H.; Mizuta,K.; Nishimura,H.
EPI231854	HE	Japan	1996-May-23	EPI_ISL_66407	C/Miyagi/8/96		Import from public-domain	Matsuzaki,Y.; Sugawara,K.; Furuse,Y.; Shimotai,Y.; Hongo,S.; Oshitani,H.; Mizuta,K.; Nishimura,H.
EPI231860	MP	Japan	1996-May-23	EPI_ISL_66407	C/Miyagi/8/96		Import from public-domain	Matsuzaki,Y.; Sugawara,K.; Furuse,Y.; Shimotai,Y.; Hongo,S.; Oshitani,H.; Mizuta,K.; Nishimura,H.
EPI816607	NP	Japan	1996-May-23	EPI_ISL_66407	C/Miyagi/8/96		Import from public-domain	Matsuzaki,Y.; Sugawara,K.; Furuse,Y.; Shimotai,Y.; Hongo,S.; Oshitani,H.; Mizuta,K.; Nishimura,H.
EPI231857	NS	Japan	1996-May-23	EPI_ISL_66407	C/Miyagi/8/96		Import from public-domain	Matsuzaki,Y.; Sugawara,K.; Furuse,Y.; Shimotai,Y.; Hongo,S.; Oshitani,H.; Mizuta,K.; Nishimura,H.

Segment ID	Segment	Country	Collection date	Isolate-ID	Isolate name	Originating Lab	Submitting Lab	Authors
EPI816502	P3	Japan	1996-May-23	EPI_ISL_66407	C/Miyagi/8/96		Import from public-domain	Matsuzaki,Y.; Sugawara,K.; Furuse,Y.; Shimotai,Y.; Hongo,S.; Oshitani,H.; Mizuta,K.; Nishimura,H.
EPI816419	PB1	Japan	1996-May-23	EPI_ISL_66407	C/Miyagi/8/96		Import from public-domain	Matsuzaki,Y.; Sugawara,K.; Furuse,Y.; Shimotai,Y.; Hongo,S.; Oshitani,H.; Mizuta,K.; Nishimura,H.
EPI813730	PB2	Japan	1996-May-23	EPI_ISL_66407	C/Miyagi/8/96		Import from public-domain	Matsuzaki,Y.; Sugawara,K.; Furuse,Y.; Shimotai,Y.; Hongo,S.; Oshitani,H.; Mizuta,K.; Nishimura,H.
EPI232109	HE	Japan	1991-Jan-01	EPI_ISL_66358	C/Miyagi/9/91		Import from public-domain	Matsuzaki,Y.
EPI232115	MP	Japan	1991-Jan-01	EPI_ISL_66358	C/Miyagi/9/91		Import from public-domain	Matsuzaki,Y.
EPI231569	NS	Japan	1991-Jan-01	EPI_ISL_66358	C/Miyagi/9/91		Import from public-domain	Matsuzaki,Y.
EPI231862	HE	Japan	1996-Jun-03	EPI_ISL_66408	C/Miyagi/9/96		Import from public-domain	Matsuzaki,Y.; Sugawara,K.; Furuse,Y.; Shimotai,Y.; Hongo,S.; Oshitani,H.; Mizuta,K.; Nishimura,H.
EPI231861	MP	Japan	1996-Jun-03	EPI_ISL_66408	C/Miyagi/9/96		Import from public-domain	Matsuzaki,Y.; Sugawara,K.; Furuse,Y.; Shimotai,Y.; Hongo,S.; Oshitani,H.; Mizuta,K.; Nishimura,H.
EPI816608	NP	Japan	1996-Jun-03	EPI_ISL_66408	C/Miyagi/9/96		Import from public-domain	Matsuzaki,Y.; Sugawara,K.; Furuse,Y.; Shimotai,Y.; Hongo,S.; Oshitani,H.; Mizuta,K.; Nishimura,H.
EPI231866	NS	Japan	1996-Jun-03	EPI_ISL_66408	C/Miyagi/9/96		Import from public-domain	Matsuzaki,Y.; Sugawara,K.; Furuse,Y.; Shimotai,Y.; Hongo,S.; Oshitani,H.; Mizuta,K.; Nishimura,H.
EPI816503	P3	Japan	1996-Jun-03	EPI_ISL_66408	C/Miyagi/9/96		Import from public-domain	Matsuzaki,Y.; Sugawara,K.; Furuse,Y.; Shimotai,Y.; Hongo,S.; Oshitani,H.; Mizuta,K.; Nishimura,H.
EPI816420	PB1	Japan	1996-Jun-03	EPI_ISL_66408	C/Miyagi/9/96		Import from public-domain	Matsuzaki,Y.; Sugawara,K.; Furuse,Y.; Shimotai,Y.; Hongo,S.; Oshitani,H.; Mizuta,K.; Nishimura,H.
EPI813732	PB2	Japan	1996-Jun-03	EPI_ISL_66408	C/Miyagi/9/96		Import from public-domain	Matsuzaki,Y.; Sugawara,K.; Furuse,Y.; Shimotai,Y.; Hongo,S.; Oshitani,H.; Mizuta,K.; Nishimura,H.
EPI232128	MP	Japan	1985-Jan-01	EPI_ISL_66352	C/Nara/1/85		Import from public-domain	Matsuzaki,Y.
EPI231561	NS	Japan	1985-Jan-01	EPI_ISL_66352	C/Nara/1/85		Import from public-domain	Matsuzaki,Y.
EPI231557	NS	Japan	1986-Jan-01	EPI_ISL_66348	C/Nara/1/86		Import from public-domain	Matsuzaki,Y.
EPI232141	HE	Japan	1985-Jan-01	EPI_ISL_66353	C/Nara/2/85		Import from public-domain	Matsuzaki,Y.
EPI232129	MP	Japan	1985-Jan-01	EPI_ISL_66353	C/Nara/2/85		Import from public-domain	Matsuzaki,Y.
EPI231562	NS	Japan	1985-Jan-01	EPI_ISL_66353	C/Nara/2/85		Import from public-domain	Matsuzaki,Y.
EPI231545	HE	Japan	1982-Jan-01	EPI_ISL_66342	C/Nara/82		Import from public-domain	Adachi,K.; Kitame,F.; Sugawara,K.; Nishimura,H.; Nakamura,K.
EPI232146	MP	Japan	1982-Jan-01	EPI_ISL_66434	C/Nara/82		Import from public-domain	Matsuzaki,Y.; Sugawara,K.; Furuse,Y.; Shimotai,Y.; Hongo,S.; Oshitani,H.; Mizuta,K.; Nishimura,H.
EPI816589	NP	Japan	1982-Jan-01	EPI_ISL_66434	C/Nara/82		Import from public-domain	Matsuzaki,Y.; Sugawara,K.; Furuse,Y.; Shimotai,Y.; Hongo,S.; Oshitani,H.; Mizuta,K.; Nishimura,H.
EPI232023	NS	Japan	1982-Jan-01	EPI_ISL_66434	C/Nara/82		Import from public-domain	Matsuzaki,Y.; Sugawara,K.; Furuse,Y.; Shimotai,Y.; Hongo,S.; Oshitani,H.; Mizuta,K.; Nishimura,H.
EPI816490	P3	Japan	1982-Jan-01	EPI_ISL_66434	C/Nara/82		Import from public-domain	Matsuzaki,Y.; Sugawara,K.; Furuse,Y.; Shimotai,Y.; Hongo,S.; Oshitani,H.; Mizuta,K.; Nishimura,H.
EPI815446	PB1	Japan	1982-Jan-01	EPI_ISL_66434	C/Nara/82		Import from public-domain	Matsuzaki,Y.; Sugawara,K.; Furuse,Y.; Shimotai,Y.; Hongo,S.; Oshitani,H.; Mizuta,K.; Nishimura,H.
EPI815369	PB2	Japan	1982-Jan-01	EPI_ISL_66434	C/Nara/82		Import from public-domain	Matsuzaki,Y.; Sugawara,K.; Furuse,Y.; Shimotai,Y.; Hongo,S.; Oshitani,H.; Mizuta,K.; Nishimura,H.
EPI231528	HE	United States	1976-Jan-01	EPI_ISL_66327	C/New Jersey/1/76[e]		Import from public-domain	Matsuzaki,Y.; Sugawara,K.; Furuse,Y.; Shimotai,Y.; Hongo,S.; Oshitani,H.; Mizuta,K.; Nishimura,H.
EPI231872	MP	United States	1976-Jan-01	EPI_ISL_66409	C/NewJersey/76[e]		Import from public-domain	Matsuzaki,Y.
EPI231871	NP	United States	1976-Jan-01	EPI_ISL_66409	C/NewJersey/76[e]		Import from public-domain	Matsuzaki,Y.
EPI231873	NS	United States	1976-Jan-01	EPI_ISL_66409	C/NewJersey/76[e]		Import from public-domain	Matsuzaki,Y.
EPI231870	P3	United States	1976-Jan-01	EPI_ISL_66409	C/NewJersey/76[e]		Import from public-domain	Matsuzaki,Y.
EPI231869	PB1	United States	1976-Jan-01	EPI_ISL_66409	C/NewJersey/76[e]		Import from public-domain	Matsuzaki,Y.
EPI231868	PB2	United States	1976-Jan-01	EPI_ISL_66409	C/NewJersey/76[e]		Import from public-domain	Matsuzaki,Y.

Segment ID	Segment	Country	Collection date	Isolate-ID	Isolate name	Originating Lab	Submitting Lab	Authors
EPI228324	HE	Japan	2004-Jan-01	EPI_ISL_65160	C/Niigata/1/2004		Import from public-domain	Matsuzaki,Y.
EPI1183986	HE	Germany	2012-Nov-16	EPI_ISL_300534	C/Nordrhein-Westfalen/13-00344/2012		Robert Koch Institute Nationales Referenzzentrum für Influenza	Biere, B.; Fritsch, A.; Schweiger, B.
EPI1183992	HE	Germany	2013-Feb-27	EPI_ISL_300540	C/Nordrhein-Westfalen/13-04022/2013		Robert Koch Institute Nationales Referenzzentrum für Influenza	Biere, B.; Fritsch, A.; Schweiger, B.
EPI228328	HE	Japan	2004-Jan-01	EPI_ISL_65164	C/Osaka/2/2004		Import from public-domain	Matsuzaki,Y.
EPI581552	HE	Philippines	2013-Jan-01	EPI_ISL_176788	C/Palawan/1/2013		Import from public-domain	Odagiri,T.; Matsuzaki,Y.; Okamoto,M.; Hongo,S.; Oshitani,H.
EPI621717	MP	Philippines	2013-Jan-01	EPI_ISL_176788	C/Palawan/1/2013		Import from public-domain	Odagiri,T.; Matsuzaki,Y.; Okamoto,M.; Hongo,S.; Oshitani,H.
EPI621700	NS	Philippines	2013-Jan-01	EPI_ISL_176788	C/Palawan/1/2013		Import from public-domain	Odagiri,T.; Matsuzaki,Y.; Okamoto,M.; Hongo,S.; Oshitani,H.
EPI231522	HE	France	1967-Jan-01	EPI_ISL_66322	C/Paris/1/67		Import from public-domain	Matsuzaki,Y.; Sugawara,K.; Furuse,Y.; Shimotai,Y.; Hongo,S.; Oshitani,H.; Mizuta,K.; Nishimura,H.
EPI816670	MP	France	1967-Jan-01	EPI_ISL_66322	C/Paris/1/67		Import from public-domain	Matsuzaki,Y.; Sugawara,K.; Furuse,Y.; Shimotai,Y.; Hongo,S.; Oshitani,H.; Mizuta,K.; Nishimura,H.
EPI816738	NS	France	1967-Jan-01	EPI_ISL_66322	C/Paris/1/67		Import from public-domain	Matsuzaki,Y.; Sugawara,K.; Furuse,Y.; Shimotai,Y.; Hongo,S.; Oshitani,H.; Mizuta,K.; Nishimura,H.
EPI816462	P3	France	1967-Jan-01	EPI_ISL_66322	C/Paris/1/67		Import from public-domain	Matsuzaki,Y.; Sugawara,K.; Furuse,Y.; Shimotai,Y.; Hongo,S.; Oshitani,H.; Mizuta,K.; Nishimura,H.
EPI813782	PB1	France	1967-Jan-01	EPI_ISL_66322	C/Paris/1/67		Import from public-domain	Matsuzaki,Y.; Sugawara,K.; Furuse,Y.; Shimotai,Y.; Hongo,S.; Oshitani,H.; Mizuta,K.; Nishimura,H.
EPI814787	PB2	France	1967-Jan-01	EPI_ISL_66322	C/Paris/1/67		Import from public-domain	Matsuzaki,Y.; Sugawara,K.; Furuse,Y.; Shimotai,Y.; Hongo,S.; Oshitani,H.; Mizuta,K.; Nishimura,H.
EPI711146	HE	Australia	2008-Oct-29	EPI_ISL_212084	C/Perth/1/2008	Pathwest QE II Medical Centre	WHO Collaborating Centre for Reference and Research on Influenza	
EPI711132	HE	Australia	2012-Sep-23	EPI_ISL_212070	C/Perth/1/2012	Pathwest QE II Medical Centre	WHO Collaborating Centre for Reference and Research on Influenza	
EPI675716	HE	Australia	2014-Jul-18	EPI_ISL_202517	C/Perth/1/2014	Pathwest QE II Medical Centre	WHO Collaborating Centre for Reference and Research on Influenza	Deng,Y-M.; Spirason,N.; Jelley,L.; Komadina,N.
EPI711140	HE	Australia	2012-Jul-18	EPI_ISL_212078	C/Perth/10/2012	Pathwest QE II Medical Centre	WHO Collaborating Centre for Reference and Research on Influenza	
EPI675712	HE	Australia	2014-Aug-17	EPI_ISL_202513	C/Perth/10/2014	Pathwest QE II Medical Centre	WHO Collaborating Centre for Reference and Research on Influenza	Deng,Y-M.; Spirason,N.; Jelley,L.; Komadina,N.
EPI711141	HE	Australia	2012-Jul-18	EPI_ISL_212079	C/Perth/11/2012	Pathwest QE II Medical Centre	WHO Collaborating Centre for Reference and Research on Influenza	
EPI711142	HE	Australia	2012-Jul-14	EPI_ISL_212080	C/Perth/12/2012	Pathwest QE II Medical Centre	WHO Collaborating Centre for Reference and Research on Influenza	
EPI675713	HE	Australia	2014-Aug-18	EPI_ISL_202514	C/Perth/12/2014	Pathwest QE II Medical Centre	WHO Collaborating Centre for Reference and Research on Influenza	Deng,Y-M.; Spirason,N.; Jelley,L.; Komadina,N.
EPI711143	HE	Australia	2012-Jun-01	EPI_ISL_212081	C/Perth/13/2012	Pathwest QE II Medical Centre	WHO Collaborating Centre for Reference and Research on Influenza	
EPI675722	HE	Australia	2014-Aug-13	EPI_ISL_202523	C/Perth/15/2014	Pathwest QE II Medical Centre	WHO Collaborating Centre for Reference and Research on Influenza	Deng,Y-M.; Spirason,N.; Jelley,L.; Komadina,N.
EPI675720	HE	Australia	2014-Aug-14	EPI_ISL_202521	C/Perth/17/2014	Pathwest QE II Medical Centre	WHO Collaborating Centre for Reference and Research on Influenza	Deng,Y-M.; Spirason,N.; Jelley,L.; Komadina,N.
EPI675721	HE	Australia	2014-Aug-23	EPI_ISL_202522	C/Perth/19/2014	Pathwest QE II Medical Centre	WHO Collaborating Centre for Reference and Research on Influenza	Deng,Y-M.; Spirason,N.; Jelley,L.; Komadina,N.
EPI711145	HE	Australia	2008-Oct-20	EPI_ISL_212083	C/Perth/2/2008	Pathwest QE II Medical Centre	WHO Collaborating Centre for Reference and Research on Influenza	
EPI711133	HE	Australia	2012-Sep-02	EPI_ISL_212071	C/Perth/2/2012	Pathwest QE II Medical Centre	WHO Collaborating Centre for Reference and Research on Influenza	
EPI675717	HE	Australia	2014-Jul-20	EPI_ISL_202518	C/Perth/2/2014	Pathwest QE II Medical Centre	WHO Collaborating Centre for Reference and Research on Influenza	Deng,Y-M.; Spirason,N.; Jelley,L.; Komadina,N.
EPI675714	HE	Australia	2014-Aug-26	EPI_ISL_202515	C/Perth/23/2014	Pathwest QE II Medical Centre	WHO Collaborating Centre for Reference and Research on Influenza	Deng,Y-M.; Spirason,N.; Jelley,L.; Komadina,N.
EPI675715	HE	Australia	2014-Sep-13	EPI_ISL_202516	C/Perth/26/2014	Pathwest QE II Medical Centre	WHO Collaborating Centre for Reference and Research on Influenza	Deng,Y-M.; Spirason,N.; Jelley,L.; Komadina,N.
EPI711147	HE	Australia	2008-Oct-03	EPI_ISL_212085	C/Perth/3/2008	Pathwest QE II Medical Centre	WHO Collaborating Centre for Reference and Research on Influenza	
EPI711134	HE	Australia	2012-Sep-01	EPI_ISL_212072	C/Perth/3/2012	Pathwest QE II Medical Centre	WHO Collaborating Centre for Reference and Research on Influenza	
EPI675710	HE	Australia	2014-Oct-03	EPI_ISL_202511	C/Perth/31/2014	Pathwest QE II Medical Centre	WHO Collaborating Centre for Reference and Research on Influenza	Deng,Y-M.; Spirason,N.; Jelley,L.; Komadina,N.

Segment ID	Segment	Country	Collection date	Isolate-ID	Isolate name	Originating Lab	Submitting Lab	Authors
EPI711135	HE	Australia	2012-Aug-19	EPI_ISL_212073	C/Perth/5/2012	Pathwest QE II Medical Centre	WHO Collaborating Centre for Reference and Research on Influenza	
EPI675718	HE	Australia	2014-Aug-06	EPI_ISL_202519	C/Perth/5/2014	Pathwest QE II Medical Centre	WHO Collaborating Centre for Reference and Research on Influenza	Deng,Y-M.; Spirason,N.; Jelley,L.; Komadina,N.
EPI711136	HE	Australia	2012-Aug-19	EPI_ISL_212074	C/Perth/6/2012	Pathwest QE II Medical Centre	WHO Collaborating Centre for Reference and Research on Influenza	
EPI711137	HE	Australia	2012-Aug-14	EPI_ISL_212075	C/Perth/7/2012	Pathwest QE II Medical Centre	WHO Collaborating Centre for Reference and Research on Influenza	
EPI711138	HE	Australia	2012-Aug-10	EPI_ISL_212076	C/Perth/8/2012	Pathwest QE II Medical Centre	WHO Collaborating Centre for Reference and Research on Influenza	
EPI675719	HE	Australia	2014-Jul-31	EPI_ISL_202520	C/Perth/8/2014	Pathwest QE II Medical Centre	WHO Collaborating Centre for Reference and Research on Influenza	Deng,Y-M.; Spirason,N.; Jelley,L.; Komadina,N.
EPI711144	HE	Australia	2010-Jul-01	EPI_ISL_212082	C/Perth/9/2010	Pathwest QE II Medical Centre	WHO Collaborating Centre for Reference and Research on Influenza	
EPI711139	HE	Australia	2012-Jul-28	EPI_ISL_212077	C/Perth/9/2012	Pathwest QE II Medical Centre	WHO Collaborating Centre for Reference and Research on Influenza	
EPI675711	HE	Australia	2014-Aug-17	EPI_ISL_202512	C/Perth/9/2014	Pathwest QE II Medical Centre	WHO Collaborating Centre for Reference and Research on Influenza	Deng,Y-M.; Spirason,N.; Jelley,L.; Komadina,N.
EPI1183998	HE	Germany	2014-Apr-07	EPI_ISL_300546	C/Rheinland-Pfalz/14-03242/2014		Robert Koch Institute Nationales Referenzzentrum für Influenza	Biere, B.; Fritsch, A.; Schweiger, B.
EPI1183984	HE	Germany	2012-Mar-26	EPI_ISL_300532	C/Sachsen/12-02741/2012		Robert Koch Institute Nationales Referenzzentrum für Influenza	Biere, B.; Fritsch, A.; Schweiger, B.
EPI231880	HE	Japan	2000-Jan-01	EPI_ISL_66410	C/Saitama/1/2000		Import from public-domain	Matsuzaki,Y.
EPI231874	MP	Japan	2000-Jan-01	EPI_ISL_66410	C/Saitama/1/2000		Import from public-domain	Matsuzaki,Y.
EPI231875	NS	Japan	2000-Jan-01	EPI_ISL_66410	C/Saitama/1/2000		Import from public-domain	Matsuzaki,Y.
EPI228326	HE	Japan	2004-Jan-01	EPI_ISL_65162	C/Saitama/1/2004		Import from public-domain	Matsuzaki,Y.
EPI231883	HE	Japan	2000-Jan-01	EPI_ISL_66411	C/Saitama/2/2000		Import from public-domain	Matsuzaki,Y.
EPI231882	MP	Japan	2000-Jan-01	EPI_ISL_66411	C/Saitama/2/2000		Import from public-domain	Matsuzaki,Y.
EPI231881	NS	Japan	2000-Jan-01	EPI_ISL_66411	C/Saitama/2/2000		Import from public-domain	Matsuzaki,Y.
EPI231892	HE	Japan	2000-Jan-01	EPI_ISL_66412	C/Saitama/3/2000		Import from public-domain	Matsuzaki,Y.
EPI231893	MP	Japan	2000-Jan-01	EPI_ISL_66412	C/Saitama/3/2000		Import from public-domain	Matsuzaki,Y.
EPI231894	NS	Japan	2000-Jan-01	EPI_ISL_66412	C/Saitama/3/2000		Import from public-domain	Matsuzaki,Y.
EPI231547	HE	Brazil	1982-Jan-01	EPI_ISL_66344	C/Sao Paulo/378/82		Import from public-domain	Matsuzaki,Y.; Sugawara,K.; Furuse,Y.; Shimotai,Y.; Hongo,S.; Oshitani,H.; Mizuta,K.; Nishimura,H.
EPI231551	MP	Brazil	1982-Jan-01	EPI_ISL_66344	C/Sao Paulo/378/82		Import from public-domain	Matsuzaki,Y.; Sugawara,K.; Furuse,Y.; Shimotai,Y.; Hongo,S.; Oshitani,H.; Mizuta,K.; Nishimura,H.
EPI816588	NP	Brazil	1982-Jan-01	EPI_ISL_66344	C/Sao Paulo/378/82		Import from public-domain	Matsuzaki,Y.; Sugawara,K.; Furuse,Y.; Shimotai,Y.; Hongo,S.; Oshitani,H.; Mizuta,K.; Nishimura,H.
EPI231548	NS	Brazil	1982-Jan-01	EPI_ISL_66344	C/Sao Paulo/378/82		Import from public-domain	Matsuzaki,Y.; Sugawara,K.; Furuse,Y.; Shimotai,Y.; Hongo,S.; Oshitani,H.; Mizuta,K.; Nishimura,H.
EPI816489	P3	Brazil	1982-Jan-01	EPI_ISL_66344	C/Sao Paulo/378/82		Import from public-domain	Matsuzaki,Y.; Sugawara,K.; Furuse,Y.; Shimotai,Y.; Hongo,S.; Oshitani,H.; Mizuta,K.; Nishimura,H.
EPI815604	PB1	Brazil	1982-Jan-01	EPI_ISL_66344	C/Sao Paulo/378/82		Import from public-domain	Matsuzaki,Y.; Sugawara,K.; Furuse,Y.; Shimotai,Y.; Hongo,S.; Oshitani,H.; Mizuta,K.; Nishimura,H.
EPI813721	PB2	Brazil	1982-Jan-01	EPI_ISL_66344	C/Sao Paulo/378/82		Import from public-domain	Matsuzaki,Y.; Sugawara,K.; Furuse,Y.; Shimotai,Y.; Hongo,S.; Oshitani,H.; Mizuta,K.; Nishimura,H.
EPI232156	HE	Japan	1971-Jan-01	EPI_ISL_66325	C/Sapporo/71		Import from public-domain	Matsuzaki,Y.; Sugawara,K.; Furuse,Y.; Shimotai,Y.; Hongo,S.; Oshitani,H.; Mizuta,K.; Nishimura,H.
EPI232157	MP	Japan	1971-Jan-01	EPI_ISL_66325	C/Sapporo/71		Import from public-domain	Matsuzaki,Y.; Sugawara,K.; Furuse,Y.; Shimotai,Y.; Hongo,S.; Oshitani,H.; Mizuta,K.; Nishimura,H.
EPI816574	NP	Japan	1971-Jan-01	EPI_ISL_66325	C/Sapporo/71		Import from public-domain	Matsuzaki,Y.; Sugawara,K.; Furuse,Y.; Shimotai,Y.; Hongo,S.; Oshitani,H.; Mizuta,K.; Nishimura,H.
EPI231526	NS	Japan	1971-Jan-01	EPI_ISL_66325	C/Sapporo/71		Import from public-domain	Matsuzaki,Y.; Sugawara,K.; Furuse,Y.; Shimotai,Y.; Hongo,S.; Oshitani,H.; Mizuta,K.; Nishimura,H.
EPI816464	P3	Japan	1971-Jan-01	EPI_ISL_66325	C/Sapporo/71		Import from public-domain	Matsuzaki,Y.; Sugawara,K.; Furuse,Y.; Shimotai,Y.; Hongo,S.; Oshitani,H.; Mizuta,K.; Nishimura,H.

Segment ID	Segment	Country	Collection date	Isolate-ID	Isolate name	Originating Lab	Submitting Lab	Authors
EPI813780	PB1	Japan	1971-Jan-01	EPI_ISL_66325	C/Sapporo/71		Import from public-domain	Matsuzaki,Y.; Sugawara,K.; Furuse,Y.; Shimotai,Y.; Hongo,S.; Oshitani,H.; Mizuta,K.; Nishimura,H.
EPI814887	PB2	Japan	1971-Jan-01	EPI_ISL_66325	C/Sapporo/71		Import from public-domain	Matsuzaki,Y.; Sugawara,K.; Furuse,Y.; Shimotai,Y.; Hongo,S.; Oshitani,H.; Mizuta,K.; Nishimura,H.
EPI1183988	HE	Germany	2012-Nov-27	EPI_ISL_300536	C/Schleswig-Holstein/13-00498/2012		Robert Koch Institute Nationales Referenzzentrum für Influenza	Biere, B.; Fritsch, A.; Schweiger, B.
EPI669896	NP	United Kingdc	2007-Jul-01	EPI_ISL_201031	C/Scotland/5500/2007		Import from public-domain	Smith,D.B.; Gaunt,E.R.; Digard,P.; Templeton,K.; Simmonds,P.
EPI669891	P3	United Kingdc	2007-Jul-01	EPI_ISL_201031	C/Scotland/5500/2007		Import from public-domain	Smith,D.B.; Gaunt,E.R.; Digard,P.; Templeton,K.; Simmonds,P.
EPI669877	PB1	United Kingdc	2007-Jul-01	EPI_ISL_201031	C/Scotland/5500/2007		Import from public-domain	Smith,D.B.; Gaunt,E.R.; Digard,P.; Templeton,K.; Simmonds,P.
EPI669886	PB2	United Kingdc	2007-Jul-01	EPI_ISL_201031	C/Scotland/5500/2007		Import from public-domain	Smith,D.B.; Gaunt,E.R.; Digard,P.; Templeton,K.; Simmonds,P.
EPI669892	HE	United Kingdc	2007-Nov-01	EPI_ISL_201034	C/Scotland/6855/2007		Import from public-domain	Smith,D.B.; Gaunt,E.R.; Digard,P.; Templeton,K.; Simmonds,P.
EPI669902	MP	United Kingdc	2007-Nov-01	EPI_ISL_201034	C/Scotland/6855/2007		Import from public-domain	Smith,D.B.; Gaunt,E.R.; Digard,P.; Templeton,K.; Simmonds,P.
EPI669901	NP	United Kingdc	2007-Nov-01	EPI_ISL_201034	C/Scotland/6855/2007		Import from public-domain	Smith,D.B.; Gaunt,E.R.; Digard,P.; Templeton,K.; Simmonds,P.
EPI671829	NS	United Kingdc	2007-Nov-01	EPI_ISL_201034	C/Scotland/6855/2007		Import from public-domain	Smith,D.B.; Gaunt,E.R.; Digard,P.; Templeton,K.; Simmonds,P.
EPI669890	P3	United Kingdc	2007-Nov-01	EPI_ISL_201034	C/Scotland/6855/2007		Import from public-domain	Smith,D.B.; Gaunt,E.R.; Digard,P.; Templeton,K.; Simmonds,P.
EPI669880	PB1	United Kingdc	2007-Nov-01	EPI_ISL_201034	C/Scotland/6855/2007		Import from public-domain	Smith,D.B.; Gaunt,E.R.; Digard,P.; Templeton,K.; Simmonds,P.
EPI669885	PB2	United Kingdc	2007-Nov-01	EPI_ISL_201034	C/Scotland/6855/2007		Import from public-domain	Smith,D.B.; Gaunt,E.R.; Digard,P.; Templeton,K.; Simmonds,P.
EPI669893	HE	United Kingdc	2007-Dec-01	EPI_ISL_201035	C/Scotland/7274/2007		Import from public-domain	Smith,D.B.; Gaunt,E.R.; Digard,P.; Templeton,K.; Simmonds,P.
EPI669903	MP	United Kingdc	2007-Dec-01	EPI_ISL_201035	C/Scotland/7274/2007		Import from public-domain	Smith,D.B.; Gaunt,E.R.; Digard,P.; Templeton,K.; Simmonds,P.
EPI669899	NP	United Kingdc	2007-Dec-01	EPI_ISL_201035	C/Scotland/7274/2007		Import from public-domain	Smith,D.B.; Gaunt,E.R.; Digard,P.; Templeton,K.; Simmonds,P.
EPI671832	NS	United Kingdc	2007-Dec-01	EPI_ISL_201035	C/Scotland/7274/2007		Import from public-domain	Smith,D.B.; Gaunt,E.R.; Digard,P.; Templeton,K.; Simmonds,P.
EPI669889	P3	United Kingdc	2007-Dec-01	EPI_ISL_201035	C/Scotland/7274/2007		Import from public-domain	Smith,D.B.; Gaunt,E.R.; Digard,P.; Templeton,K.; Simmonds,P.
EPI669881	PB1	United Kingdc	2007-Dec-01	EPI_ISL_201035	C/Scotland/7274/2007		Import from public-domain	Smith,D.B.; Gaunt,E.R.; Digard,P.; Templeton,K.; Simmonds,P.
EPI669884	PB2	United Kingdc	2007-Dec-01	EPI_ISL_201035	C/Scotland/7274/2007		Import from public-domain	Smith,D.B.; Gaunt,E.R.; Digard,P.; Templeton,K.; Simmonds,P.
EPI669894	HE	United Kingdc	2007-Dec-01	EPI_ISL_201033	C/Scotland/7383/2007		Import from public-domain	Smith,D.B.; Gaunt,E.R.; Digard,P.; Templeton,K.; Simmonds,P.
EPI669905	MP	United Kingdc	2007-Dec-01	EPI_ISL_201033	C/Scotland/7383/2007		Import from public-domain	Smith,D.B.; Gaunt,E.R.; Digard,P.; Templeton,K.; Simmonds,P.
EPI669897	NP	United Kingdc	2007-Dec-01	EPI_ISL_201033	C/Scotland/7383/2007		Import from public-domain	Smith,D.B.; Gaunt,E.R.; Digard,P.; Templeton,K.; Simmonds,P.
EPI671833	NS	United Kingdc	2007-Dec-01	EPI_ISL_201033	C/Scotland/7383/2007		Import from public-domain	Smith,D.B.; Gaunt,E.R.; Digard,P.; Templeton,K.; Simmonds,P.
EPI669888	P3	United Kingdc	2007-Dec-01	EPI_ISL_201033	C/Scotland/7383/2007		Import from public-domain	Smith,D.B.; Gaunt,E.R.; Digard,P.; Templeton,K.; Simmonds,P.
EPI669879	PB1	United Kingdc	2007-Dec-01	EPI_ISL_201033	C/Scotland/7383/2007		Import from public-domain	Smith,D.B.; Gaunt,E.R.; Digard,P.; Templeton,K.; Simmonds,P.
EPI669883	PB2	United Kingdc	2007-Dec-01	EPI_ISL_201033	C/Scotland/7383/2007		Import from public-domain	Smith,D.B.; Gaunt,E.R.; Digard,P.; Templeton,K.; Simmonds,P.
EPI669895	HE	United Kingdc	2007-Dec-01	EPI_ISL_201032	C/Scotland/7482/2007		Import from public-domain	Smith,D.B.; Gaunt,E.R.; Digard,P.; Templeton,K.; Simmonds,P.
EPI669904	MP	United Kingdc	2007-Dec-01	EPI_ISL_201032	C/Scotland/7482/2007		Import from public-domain	Smith,D.B.; Gaunt,E.R.; Digard,P.; Templeton,K.; Simmonds,P.
EPI671830	NS	United Kingdc	2007-Dec-01	EPI_ISL_201032	C/Scotland/7482/2007		Import from public-domain	Smith,D.B.; Gaunt,E.R.; Digard,P.; Templeton,K.; Simmonds,P.
EPI669887	P3	United Kingdc	2007-Dec-01	EPI_ISL_201032	C/Scotland/7482/2007		Import from public-domain	Smith,D.B.; Gaunt,E.R.; Digard,P.; Templeton,K.; Simmonds,P.
EPI669878	PB1	United Kingdc	2007-Dec-01	EPI_ISL_201032	C/Scotland/7482/2007		Import from public-domain	Smith,D.B.; Gaunt,E.R.; Digard,P.; Templeton,K.; Simmonds,P.

Segment ID	Segment	Country	Collection date	Isolate-ID	Isolate name	Originating Lab	Submitting Lab	Authors
EPI669882	PB2	United Kingdc	2007-Dec-01	EPI_ISL_201032	C/Scotland/7482/2007		Import from public-domain	Smith,D.B.; Gaunt,E.R.; Digard,P.; Templeton,K.; Simmonds,P.
EPI581540	HE	Japan	2008-Jan-01	EPI_ISL_176775	C/Sendai/TU1/2008		Import from public-domain	Odagiri,T.; Matsuzaki,Y.; Okamoto,M.; Hongo,S.; Oshitani,H.
EPI621707	MP	Japan	2008-Jan-01	EPI_ISL_176775	C/Sendai/TU1/2008		Import from public-domain	Odagiri,T.; Matsuzaki,Y.; Okamoto,M.; Hongo,S.; Oshitani,H.
EPI621691	NS	Japan	2008-Jan-01	EPI_ISL_176775	C/Sendai/TU1/2008		Import from public-domain	Odagiri,T.; Matsuzaki,Y.; Okamoto,M.; Hongo,S.; Oshitani,H.
EPI581544	HE	Japan	2012-Jan-01	EPI_ISL_176779	C/Sendai/TU1/2012		Import from public-domain	Odagiri,T.; Matsuzaki,Y.; Okamoto,M.; Hongo,S.; Oshitani,H.
EPI621711	MP	Japan	2012-Jan-01	EPI_ISL_176779	C/Sendai/TU1/2012		Import from public-domain	Odagiri,T.; Matsuzaki,Y.; Okamoto,M.; Hongo,S.; Oshitani,H.
EPI621695	NS	Japan	2012-Jan-01	EPI_ISL_176779	C/Sendai/TU1/2012		Import from public-domain	Odagiri,T.; Matsuzaki,Y.; Okamoto,M.; Hongo,S.; Oshitani,H.
EPI581541	HE	Japan	2008-Jan-01	EPI_ISL_176776	C/Sendai/TU2/2008		Import from public-domain	Odagiri,T.; Matsuzaki,Y.; Okamoto,M.; Hongo,S.; Oshitani,H.
EPI621708	MP	Japan	2008-Jan-01	EPI_ISL_176776	C/Sendai/TU2/2008		Import from public-domain	Odagiri,T.; Matsuzaki,Y.; Okamoto,M.; Hongo,S.; Oshitani,H.
EPI621692	NS	Japan	2008-Jan-01	EPI_ISL_176776	C/Sendai/TU2/2008		Import from public-domain	Odagiri,T.; Matsuzaki,Y.; Okamoto,M.; Hongo,S.; Oshitani,H.
EPI581545	HE	Japan	2012-Jan-01	EPI_ISL_176780	C/Sendai/TU2/2012		Import from public-domain	Odagiri,T.; Matsuzaki,Y.; Okamoto,M.; Hongo,S.; Oshitani,H.
EPI621712	MP	Japan	2012-Jan-01	EPI_ISL_176780	C/Sendai/TU2/2012		Import from public-domain	Odagiri,T.; Matsuzaki,Y.; Okamoto,M.; Hongo,S.; Oshitani,H.
EPI621696	NS	Japan	2012-Jan-01	EPI_ISL_176780	C/Sendai/TU2/2012		Import from public-domain	Odagiri,T.; Matsuzaki,Y.; Okamoto,M.; Hongo,S.; Oshitani,H.
EPI581542	HE	Japan	2008-Jan-01	EPI_ISL_176777	C/Sendai/TU3/2008		Import from public-domain	Odagiri,T.; Matsuzaki,Y.; Okamoto,M.; Hongo,S.; Oshitani,H.
EPI621709	MP	Japan	2008-Jan-01	EPI_ISL_176777	C/Sendai/TU3/2008		Import from public-domain	Odagiri,T.; Matsuzaki,Y.; Okamoto,M.; Hongo,S.; Oshitani,H.
EPI621693	NS	Japan	2008-Jan-01	EPI_ISL_176777	C/Sendai/TU3/2008		Import from public-domain	Odagiri,T.; Matsuzaki,Y.; Okamoto,M.; Hongo,S.; Oshitani,H.
EPI581555	HE	Japan	2008-Jan-01	EPI_ISL_176996	C/Sendai/TU4/2008		Import from public-domain	Odagiri,T.; Matsuzaki,Y.; Okamoto,M.; Suzuki,A.; Saito,M.; Tamaki,R.; Lupisan,S.P.; Sombrero,L.T.; Hongo,S.; Oshitani,H.
EPI581543	HE	Japan	2008-Jan-01	EPI_ISL_176778	C/Sendai/TU5/2008		Import from public-domain	Odagiri,T.; Matsuzaki,Y.; Okamoto,M.; Hongo,S.; Oshitani,H.
EPI621710	MP	Japan	2008-Jan-01	EPI_ISL_176778	C/Sendai/TU5/2008		Import from public-domain	Odagiri,T.; Matsuzaki,Y.; Okamoto,M.; Hongo,S.; Oshitani,H.
EPI621694	NS	Japan	2008-Jan-01	EPI_ISL_176778	C/Sendai/TU5/2008		Import from public-domain	Odagiri,T.; Matsuzaki,Y.; Okamoto,M.; Hongo,S.; Oshitani,H.
EPI231902	HE	Japan	1979-Jan-01	EPI_ISL_66413	C/Shizuoka/79		Import from public-domain	Matsuzaki,Y.; Sugawara,K.; Furuse,Y.; Shimotai,Y.; Hongo,S.; Oshitani,H.; Mizuta,K.; Nishimura,H.
EPI231904	MP	Japan	1979-Jan-01	EPI_ISL_66413	C/Shizuoka/79		Import from public-domain	Matsuzaki,Y.; Sugawara,K.; Furuse,Y.; Shimotai,Y.; Hongo,S.; Oshitani,H.; Mizuta,K.; Nishimura,H.
EPI816581	NP	Japan	1979-Jan-01	EPI_ISL_66413	C/Shizuoka/79		Import from public-domain	Matsuzaki,Y.; Sugawara,K.; Furuse,Y.; Shimotai,Y.; Hongo,S.; Oshitani,H.; Mizuta,K.; Nishimura,H.
EPI231905	NS	Japan	1979-Jan-01	EPI_ISL_66413	C/Shizuoka/79		Import from public-domain	Matsuzaki,Y.; Sugawara,K.; Furuse,Y.; Shimotai,Y.; Hongo,S.; Oshitani,H.; Mizuta,K.; Nishimura,H.
EPI816482	P3	Japan	1979-Jan-01	EPI_ISL_66413	C/Shizuoka/79		Import from public-domain	Matsuzaki,Y.; Sugawara,K.; Furuse,Y.; Shimotai,Y.; Hongo,S.; Oshitani,H.; Mizuta,K.; Nishimura,H.
EPI813790	PB1	Japan	1979-Jan-01	EPI_ISL_66413	C/Shizuoka/79		Import from public-domain	Matsuzaki,Y.; Sugawara,K.; Furuse,Y.; Shimotai,Y.; Hongo,S.; Oshitani,H.; Mizuta,K.; Nishimura,H.
EPI814536	PB2	Japan	1979-Jan-01	EPI_ISL_66413	C/Shizuoka/79		Import from public-domain	Matsuzaki,Y.; Sugawara,K.; Furuse,Y.; Shimotai,Y.; Hongo,S.; Oshitani,H.; Mizuta,K.; Nishimura,H.
EPI280421	HE	Singapore	2006-May-18	EPI_ISL_79749	C/Singapore/DSO-050530/2006		Import from public-domain	Ting,P.; Seah,S.L.K.; Lim,E.A.S.; Liaw,J.C.W.; Tan,B.H.
EPI509214	MP	Singapore	2006-May-18	EPI_ISL_79749	C/Singapore/DSO-050530/2006		Import from public-domain	Ting,P.; Seah,S.L.K.; Lim,E.A.S.; Liaw,J.C.W.; Tan,B.H.
EPI509218	NP	Singapore	2006-May-18	EPI_ISL_79749	C/Singapore/DSO-050530/2006		Import from public-domain	Ting,P.; Seah,S.L.K.; Lim,E.A.S.; Liaw,J.C.W.; Tan,B.H.
EPI509223	NS	Singapore	2006-May-18	EPI_ISL_79749	C/Singapore/DSO-050530/2006		Import from public-domain	Ting,P.; Seah,S.L.K.; Lim,E.A.S.; Liaw,J.C.W.; Tan,B.H.
EPI509227	P3	Singapore	2006-May-18	EPI_ISL_79749	C/Singapore/DSO-050530/2006		Import from public-domain	Ting,P.; Seah,S.L.K.; Lim,E.A.S.; Liaw,J.C.W.; Tan,B.H.
EPI509231	PB1	Singapore	2006-May-18	EPI_ISL_79749	C/Singapore/DSO-050530/2006		Import from public-domain	Ting,P.; Seah,S.L.K.; Lim,E.A.S.; Liaw,J.C.W.; Tan,B.H.

Segment ID	Segment	Country	Collection date	Isolate-ID	Isolate name	Originating Lab	Submitting Lab	Authors
EPI509235	PB2	Singapore	2006-May-18	EPI_ISL_79749	C/Singapore/DSO-050530/2006		Import from public-domain	Ting,P.; Seah,S.L.K.; Lim,E.A.S.; Liaw,J.C.W.; Tan,B.H.
EPI280419	HE	Singapore	2006-Nov-17	EPI_ISL_79747	C/Singapore/DSO-070170/2006		Import from public-domain	Ting,P.; Seah,S.L.K.; Lim,E.A.S.; Liaw,J.C.W.; Tan,B.H.
EPI509215	MP	Singapore	2006-Nov-17	EPI_ISL_79747	C/Singapore/DSO-070170/2006		Import from public-domain	Ting,P.; Seah,S.L.K.; Lim,E.A.S.; Liaw,J.C.W.; Tan,B.H.
EPI509219	NP	Singapore	2006-Nov-17	EPI_ISL_79747	C/Singapore/DSO-070170/2006		Import from public-domain	Ting,P.; Seah,S.L.K.; Lim,E.A.S.; Liaw,J.C.W.; Tan,B.H.
EPI509224	NS	Singapore	2006-Nov-17	EPI_ISL_79747	C/Singapore/DSO-070170/2006		Import from public-domain	Ting,P.; Seah,S.L.K.; Lim,E.A.S.; Liaw,J.C.W.; Tan,B.H.
EPI509228	P3	Singapore	2006-Nov-17	EPI_ISL_79747	C/Singapore/DSO-070170/2006		Import from public-domain	Ting,P.; Seah,S.L.K.; Lim,E.A.S.; Liaw,J.C.W.; Tan,B.H.
EPI509232	PB1	Singapore	2006-Nov-17	EPI_ISL_79747	C/Singapore/DSO-070170/2006		Import from public-domain	Ting,P.; Seah,S.L.K.; Lim,E.A.S.; Liaw,J.C.W.; Tan,B.H.
EPI509236	PB2	Singapore	2006-Nov-17	EPI_ISL_79747	C/Singapore/DSO-070170/2006		Import from public-domain	Ting,P.; Seah,S.L.K.; Lim,E.A.S.; Liaw,J.C.W.; Tan,B.H.
EPI280420	HE	Singapore	2006-Nov-29	EPI_ISL_79748	C/Singapore/DSO-070193/2006		Import from public-domain	Ting,P.; Seah,S.L.K.; Lim,E.A.S.; Liaw,J.C.W.; Tan,B.H.
EPI509216	MP	Singapore	2006-Nov-29	EPI_ISL_79748	C/Singapore/DSO-070193/2006		Import from public-domain	Ting,P.; Seah,S.L.K.; Lim,E.A.S.; Liaw,J.C.W.; Tan,B.H.
EPI509220	NP	Singapore	2006-Nov-29	EPI_ISL_79748	C/Singapore/DSO-070193/2006		Import from public-domain	Ting,P.; Seah,S.L.K.; Lim,E.A.S.; Liaw,J.C.W.; Tan,B.H.
EPI509225	NS	Singapore	2006-Nov-29	EPI_ISL_79748	C/Singapore/DSO-070193/2006		Import from public-domain	Ting,P.; Seah,S.L.K.; Lim,E.A.S.; Liaw,J.C.W.; Tan,B.H.
EPI509229	P3	Singapore	2006-Nov-29	EPI_ISL_79748	C/Singapore/DSO-070193/2006		Import from public-domain	Ting,P.; Seah,S.L.K.; Lim,E.A.S.; Liaw,J.C.W.; Tan,B.H.
EPI509233	PB1	Singapore	2006-Nov-29	EPI_ISL_79748	C/Singapore/DSO-070193/2006		Import from public-domain	Ting,P.; Seah,S.L.K.; Lim,E.A.S.; Liaw,J.C.W.; Tan,B.H.
EPI509237	PB2	Singapore	2006-Nov-29	EPI_ISL_79748	C/Singapore/DSO-070193/2006		Import from public-domain	Ting,P.; Seah,S.L.K.; Lim,E.A.S.; Liaw,J.C.W.; Tan,B.H.
EPI280418	HE	Singapore	2006-Dec-04	EPI_ISL_79746	C/Singapore/DSO-070203/2006		Import from public-domain	Ting,P.; Seah,S.L.K.; Lim,E.A.S.; Liaw,J.C.W.; Tan,B.H.
EPI509217	MP	Singapore	2006-Dec-04	EPI_ISL_79746	C/Singapore/DSO-070203/2006		Import from public-domain	Ting,P.; Seah,S.L.K.; Lim,E.A.S.; Liaw,J.C.W.; Tan,B.H.
EPI509221	NP	Singapore	2006-Dec-04	EPI_ISL_79746	C/Singapore/DSO-070203/2006		Import from public-domain	Ting,P.; Seah,S.L.K.; Lim,E.A.S.; Liaw,J.C.W.; Tan,B.H.
EPI509226	NS	Singapore	2006-Dec-04	EPI_ISL_79746	C/Singapore/DSO-070203/2006		Import from public-domain	Ting,P.; Seah,S.L.K.; Lim,E.A.S.; Liaw,J.C.W.; Tan,B.H.
EPI509230	P3	Singapore	2006-Dec-04	EPI_ISL_79746	C/Singapore/DSO-070203/2006		Import from public-domain	Ting,P.; Seah,S.L.K.; Lim,E.A.S.; Liaw,J.C.W.; Tan,B.H.
EPI509234	PB1	Singapore	2006-Dec-04	EPI_ISL_79746	C/Singapore/DSO-070203/2006		Import from public-domain	Ting,P.; Seah,S.L.K.; Lim,E.A.S.; Liaw,J.C.W.; Tan,B.H.
EPI509238	PB2	Singapore	2006-Dec-04	EPI_ISL_79746	C/Singapore/DSO-070203/2006		Import from public-domain	Ting,P.; Seah,S.L.K.; Lim,E.A.S.; Liaw,J.C.W.; Tan,B.H.
EPI232016	HE		1947-Jan-01	EPI_ISL_66317	C/Taylor/1233/47[f]		Import from public-domain	Buonagurio,D.A.; Nakada,S.; Fitch,W.M.; Palese,P.
EPI232199	MP		1947-Jan-01	EPI_ISL_66449	C/Taylor/1233/47[f]		Import from public-domain	Hidekazu,N.
EPI231521	NS		1947-Jan-01	EPI_ISL_66317	C/Taylor/1233/47[f]		Import from public-domain	Buonagurio,D.A.; Nakada,S.; Fitch,W.M.; Palese,P.
EPI816458	P3	United States	1947-Jan-01	EPI_ISL_230234	C/Taylor/1233/1947[f]		Import from public-domain	Matsuzaki,Y.; Sugawara,K.; Furuse,Y.; Shimotai,Y.; Hongo,S.; Oshitani,H.; Mizuta,K.; Nishimura,H.
EPI813768	PB1	United States	1947-Jan-01	EPI_ISL_230234	C/Taylor/1233/1947[f]		Import from public-domain	Matsuzaki,Y.; Sugawara,K.; Furuse,Y.; Shimotai,Y.; Hongo,S.; Oshitani,H.; Mizuta,K.; Nishimura,H.
EPI813693	PB2	United States	1947-Jan-01	EPI_ISL_230234	C/Taylor/1233/1947[f]		Import from public-domain	Matsuzaki,Y.; Sugawara,K.; Furuse,Y.; Shimotai,Y.; Hongo,S.; Oshitani,H.; Mizuta,K.; Nishimura,H.
EPI1183982	HE	Germany	2012-Mar-12	EPI_ISL_300530	C/Thueringen/12-02332/2012		Robert Koch Institute Nationales Referenzzentrum für Influenza	Biere, B.; Fritsch, A.; Schweiger, B.
EPI1183983	HE	Germany	2012-Mar-19	EPI_ISL_300531	C/Thueringen/12-02562/2012		Robert Koch Institute Nationales Referenzzentrum für Influenza	Biere, B.; Fritsch, A.; Schweiger, B.
EPI1183985	HE	Germany	2012-Oct-29	EPI_ISL_300533	C/Thueringen/13-00167/2012		Robert Koch Institute Nationales Referenzzentrum für Influenza	Biere, B.; Fritsch, A.; Schweiger, B.
EPI1183989	HE	Germany	2012-Nov-28	EPI_ISL_300537	C/Thueringen/13-00580/2012		Robert Koch Institute Nationales Referenzzentrum für Influenza	Biere, B.; Fritsch, A.; Schweiger, B.
EPI813691	HE	Japan	2014-Feb-01	EPI_ISL_230276	C/Tokyo/1/2014		Import from public-domain	Matsuzaki,Y.; Sugawara,K.; Furuse,Y.; Shimotai,Y.; Hongo,S.; Oshitani,H.; Mizuta,K.; Nishimura,H.

Segment ID	Segment	Country	Collection date	Isolate-ID	Isolate name	Originating Lab	Submitting Lab	Authors
EPI816734	MP	Japan	2014-Feb-01	EPI_ISL_230276	C/Tokyo/1/2014		Import from public-domain	Matsuzaki,Y.; Sugawara,K.; Furuse,Y.; Shimotai,Y.; Hongo,S.; Oshitani,H.; Mizuta,K.; Nishimura,H.
EPI816666	NP	Japan	2014-Feb-01	EPI_ISL_230276	C/Tokyo/1/2014		Import from public-domain	Matsuzaki,Y.; Sugawara,K.; Furuse,Y.; Shimotai,Y.; Hongo,S.; Oshitani,H.; Mizuta,K.; Nishimura,H.
EPI816803	NS	Japan	2014-Feb-01	EPI_ISL_230276	C/Tokyo/1/2014		Import from public-domain	Matsuzaki,Y.; Sugawara,K.; Furuse,Y.; Shimotai,Y.; Hongo,S.; Oshitani,H.; Mizuta,K.; Nishimura,H.
EPI816567	P3	Japan	2014-Feb-01	EPI_ISL_230276	C/Tokyo/1/2014		Import from public-domain	Matsuzaki,Y.; Sugawara,K.; Furuse,Y.; Shimotai,Y.; Hongo,S.; Oshitani,H.; Mizuta,K.; Nishimura,H.
EPI816456	PB1	Japan	2014-Feb-01	EPI_ISL_230276	C/Tokyo/1/2014		Import from public-domain	Matsuzaki,Y.; Sugawara,K.; Furuse,Y.; Shimotai,Y.; Hongo,S.; Oshitani,H.; Mizuta,K.; Nishimura,H.
EPI813766	PB2	Japan	2014-Feb-01	EPI_ISL_230276	C/Tokyo/1/2014		Import from public-domain	Matsuzaki,Y.; Sugawara,K.; Furuse,Y.; Shimotai,Y.; Hongo,S.; Oshitani,H.; Mizuta,K.; Nishimura,H.
EPI813680	HE	Japan	2010-Apr-23	EPI_ISL_230265	C/Tokyo/3/2010		Import from public-domain	Matsuzaki,Y.; Sugawara,K.; Furuse,Y.; Shimotai,Y.; Hongo,S.; Oshitani,H.; Mizuta,K.; Nishimura,H.
EPI816714	MP	Japan	2010-Apr-23	EPI_ISL_230265	C/Tokyo/3/2010		Import from public-domain	Matsuzaki,Y.; Sugawara,K.; Furuse,Y.; Shimotai,Y.; Hongo,S.; Oshitani,H.; Mizuta,K.; Nishimura,H.
EPI816646	NP	Japan	2010-Apr-23	EPI_ISL_230265	C/Tokyo/3/2010		Import from public-domain	Matsuzaki,Y.; Sugawara,K.; Furuse,Y.; Shimotai,Y.; Hongo,S.; Oshitani,H.; Mizuta,K.; Nishimura,H.
EPI816783	NS	Japan	2010-Apr-23	EPI_ISL_230265	C/Tokyo/3/2010		Import from public-domain	Matsuzaki,Y.; Sugawara,K.; Furuse,Y.; Shimotai,Y.; Hongo,S.; Oshitani,H.; Mizuta,K.; Nishimura,H.
EPI816547	P3	Japan	2010-Apr-23	EPI_ISL_230265	C/Tokyo/3/2010		Import from public-domain	Matsuzaki,Y.; Sugawara,K.; Furuse,Y.; Shimotai,Y.; Hongo,S.; Oshitani,H.; Mizuta,K.; Nishimura,H.
EPI816443	PB1	Japan	2010-Apr-23	EPI_ISL_230265	C/Tokyo/3/2010		Import from public-domain	Matsuzaki,Y.; Sugawara,K.; Furuse,Y.; Shimotai,Y.; Hongo,S.; Oshitani,H.; Mizuta,K.; Nishimura,H.
EPI813753	PB2	Japan	2010-Apr-23	EPI_ISL_230265	C/Tokyo/3/2010		Import from public-domain	Matsuzaki,Y.; Sugawara,K.; Furuse,Y.; Shimotai,Y.; Hongo,S.; Oshitani,H.; Mizuta,K.; Nishimura,H.
EPI813692	HE	Japan	2014-Apr-23	EPI_ISL_230277	C/Tokyo/4/2014		Import from public-domain	Matsuzaki,Y.; Sugawara,K.; Furuse,Y.; Shimotai,Y.; Hongo,S.; Oshitani,H.; Mizuta,K.; Nishimura,H.
EPI816735	MP	Japan	2014-Apr-23	EPI_ISL_230277	C/Tokyo/4/2014		Import from public-domain	Matsuzaki,Y.; Sugawara,K.; Furuse,Y.; Shimotai,Y.; Hongo,S.; Oshitani,H.; Mizuta,K.; Nishimura,H.
EPI816667	NP	Japan	2014-Apr-23	EPI_ISL_230277	C/Tokyo/4/2014		Import from public-domain	Matsuzaki,Y.; Sugawara,K.; Furuse,Y.; Shimotai,Y.; Hongo,S.; Oshitani,H.; Mizuta,K.; Nishimura,H.
EPI816804	NS	Japan	2014-Apr-23	EPI_ISL_230277	C/Tokyo/4/2014		Import from public-domain	Matsuzaki,Y.; Sugawara,K.; Furuse,Y.; Shimotai,Y.; Hongo,S.; Oshitani,H.; Mizuta,K.; Nishimura,H.
EPI816568	P3	Japan	2014-Apr-23	EPI_ISL_230277	C/Tokyo/4/2014		Import from public-domain	Matsuzaki,Y.; Sugawara,K.; Furuse,Y.; Shimotai,Y.; Hongo,S.; Oshitani,H.; Mizuta,K.; Nishimura,H.
EPI816457	PB1	Japan	2014-Apr-23	EPI_ISL_230277	C/Tokyo/4/2014		Import from public-domain	Matsuzaki,Y.; Sugawara,K.; Furuse,Y.; Shimotai,Y.; Hongo,S.; Oshitani,H.; Mizuta,K.; Nishimura,H.
EPI813767	PB2	Japan	2014-Apr-23	EPI_ISL_230277	C/Tokyo/4/2014		Import from public-domain	Matsuzaki,Y.; Sugawara,K.; Furuse,Y.; Shimotai,Y.; Hongo,S.; Oshitani,H.; Mizuta,K.; Nishimura,H.
EPI375431	HE	Australia	2011-Oct-24	EPI_ISL_118655	C/VICTORIA/1/2011	Victorian Infectious Diseases Reference Laboratory	WHO Collaborating Centre for Reference and Research on Influenza	Deng,Y-M; Iannello,P; Caldwell,N; Komadina,N.
EPI545173	HE	Australia	2012-Nov-01	EPI_ISL_167197	C/Victoria/2/2012		Import from public-domain	Liu,R.; Hause,B.M.; Li,F.
EPI545175	MP	Australia	2012-Nov-01	EPI_ISL_167197	C/Victoria/2/2012		Import from public-domain	Liu,R.; Hause,B.M.; Li,F.
EPI545174	NP	Australia	2012-Nov-01	EPI_ISL_167197	C/Victoria/2/2012		Import from public-domain	Liu,R.; Hause,B.M.; Li,F.
EPI545176	NS	Australia	2012-Nov-01	EPI_ISL_167197	C/Victoria/2/2012		Import from public-domain	Liu,R.; Hause,B.M.; Li,F.
EPI545172	P3	Australia	2012-Nov-01	EPI_ISL_167197	C/Victoria/2/2012		Import from public-domain	Liu,R.; Hause,B.M.; Li,F.
EPI545171	PB1	Australia	2012-Nov-01	EPI_ISL_167197	C/Victoria/2/2012		Import from public-domain	Liu,R.; Hause,B.M.; Li,F.
EPI545170	PB2	Australia	2012-Nov-01	EPI_ISL_167197	C/Victoria/2/2012		Import from public-domain	Liu,R.; Hause,B.M.; Li,F.
EPI711150	HE	Australia	2014-May-07	EPI_ISL_212088	C/Victoria/2/2014	Victorian Infectious Diseases Reference Laboratory	WHO Collaborating Centre for Reference and Research on Influenza	
EPI711151	HE	Australia	2014-Aug-12	EPI_ISL_212089	C/Victoria/3/2014	Victorian Infectious Diseases Reference Laboratory	WHO Collaborating Centre for Reference and Research on Influenza	
EPI711149	HE	Australia	2012-Oct-08	EPI_ISL_212087	C/Victoria/4/2012	Victorian Infectious Diseases Reference Laboratory	WHO Collaborating Centre for Reference and Research on Influenza	
EPI711152	HE	Australia	2014-Aug-08	EPI_ISL_212090	C/Victoria/4/2014	Victorian Infectious Diseases Reference Laboratory	WHO Collaborating Centre for Reference and Research on Influenza	
EPI813662	HE	Japan	2005-Jun-07	EPI_ISL_230247	C/Yamagata/1/2005		Import from public-domain	Matsuzaki,Y.; Sugawara,K.; Furuse,Y.; Shimotai,Y.; Hongo,S.; Oshitani,H.; Mizuta,K.; Nishimura,H.

Segment ID	Segment	Country	Collection date	Isolate-ID	Isolate name	Originating Lab	Submitting Lab	Authors
EPI816693	MP	Japan	2005-Jun-07	EPI_ISL_230247	C/Yamagata/1/2005		Import from public-domain	Matsuzaki,Y.; Sugawara,K.; Furuse,Y.; Shimotai,Y.; Hongo,S.; Oshitani,H.; Mizuta,K.; Nishimura,H.
EPI816628	NP	Japan	2005-Jun-07	EPI_ISL_230247	C/Yamagata/1/2005		Import from public-domain	Matsuzaki,Y.; Sugawara,K.; Furuse,Y.; Shimotai,Y.; Hongo,S.; Oshitani,H.; Mizuta,K.; Nishimura,H.
EPI816762	NS	Japan	2005-Jun-07	EPI_ISL_230247	C/Yamagata/1/2005		Import from public-domain	Matsuzaki,Y.; Sugawara,K.; Furuse,Y.; Shimotai,Y.; Hongo,S.; Oshitani,H.; Mizuta,K.; Nishimura,H.
EPI816526	P3	Japan	2005-Jun-07	EPI_ISL_230247	C/Yamagata/1/2005		Import from public-domain	Matsuzaki,Y.; Sugawara,K.; Furuse,Y.; Shimotai,Y.; Hongo,S.; Oshitani,H.; Mizuta,K.; Nishimura,H.
EPI813808	PB1	Japan	2005-Jun-07	EPI_ISL_230247	C/Yamagata/1/2005		Import from public-domain	Matsuzaki,Y.; Sugawara,K.; Furuse,Y.; Shimotai,Y.; Hongo,S.; Oshitani,H.; Mizuta,K.; Nishimura,H.
EPI813711	PB2	Japan	2005-Jun-07	EPI_ISL_230247	C/Yamagata/1/2005		Import from public-domain	Matsuzaki,Y.; Sugawara,K.; Furuse,Y.; Shimotai,Y.; Hongo,S.; Oshitani,H.; Mizuta,K.; Nishimura,H.
EPI813669	HE	Japan	2006-Jan-23	EPI_ISL_230254	C/Yamagata/1/2006		Import from public-domain	Matsuzaki,Y.; Sugawara,K.; Furuse,Y.; Shimotai,Y.; Hongo,S.; Oshitani,H.; Mizuta,K.; Nishimura,H.
EPI816700	MP	Japan	2006-Jan-23	EPI_ISL_230254	C/Yamagata/1/2006		Import from public-domain	Matsuzaki,Y.; Sugawara,K.; Furuse,Y.; Shimotai,Y.; Hongo,S.; Oshitani,H.; Mizuta,K.; Nishimura,H.
EPI816634	NP	Japan	2006-Jan-23	EPI_ISL_230254	C/Yamagata/1/2006		Import from public-domain	Matsuzaki,Y.; Sugawara,K.; Furuse,Y.; Shimotai,Y.; Hongo,S.; Oshitani,H.; Mizuta,K.; Nishimura,H.
EPI816769	NS	Japan	2006-Jan-23	EPI_ISL_230254	C/Yamagata/1/2006		Import from public-domain	Matsuzaki,Y.; Sugawara,K.; Furuse,Y.; Shimotai,Y.; Hongo,S.; Oshitani,H.; Mizuta,K.; Nishimura,H.
EPI816533	P3	Japan	2006-Jan-23	EPI_ISL_230254	C/Yamagata/1/2006		Import from public-domain	Matsuzaki,Y.; Sugawara,K.; Furuse,Y.; Shimotai,Y.; Hongo,S.; Oshitani,H.; Mizuta,K.; Nishimura,H.
EPI816431	PB1	Japan	2006-Jan-23	EPI_ISL_230254	C/Yamagata/1/2006		Import from public-domain	Matsuzaki,Y.; Sugawara,K.; Furuse,Y.; Shimotai,Y.; Hongo,S.; Oshitani,H.; Mizuta,K.; Nishimura,H.
EPI813718	PB2	Japan	2006-Jan-23	EPI_ISL_230254	C/Yamagata/1/2006		Import from public-domain	Matsuzaki,Y.; Sugawara,K.; Furuse,Y.; Shimotai,Y.; Hongo,S.; Oshitani,H.; Mizuta,K.; Nishimura,H.
EPI813674	HE	Japan	2007-Jan-17	EPI_ISL_230259	C/Yamagata/1/2007		Import from public-domain	Matsuzaki,Y.; Sugawara,K.; Furuse,Y.; Shimotai,Y.; Hongo,S.; Oshitani,H.; Mizuta,K.; Nishimura,H.
EPI816706	MP	Japan	2007-Jan-17	EPI_ISL_230259	C/Yamagata/1/2007		Import from public-domain	Matsuzaki,Y.; Sugawara,K.; Furuse,Y.; Shimotai,Y.; Hongo,S.; Oshitani,H.; Mizuta,K.; Nishimura,H.
EPI369790	NP	Japan	2007-Jan-17	EPI_ISL_118425	C/Yamagata/1/2007		Import from public-domain	Matsuzaki,Y.; Ikeda,T.; Abiko,C.; Aoki,Y.; Mizuta,K.; Shimotai,Y.; Sugawara,K.; Hongo,S.
EPI816775	NS	Japan	2007-Jan-17	EPI_ISL_230259	C/Yamagata/1/2007		Import from public-domain	Matsuzaki,Y.; Sugawara,K.; Furuse,Y.; Shimotai,Y.; Hongo,S.; Oshitani,H.; Mizuta,K.; Nishimura,H.
EPI816539	P3	Japan	2007-Jan-17	EPI_ISL_230259	C/Yamagata/1/2007		Import from public-domain	Matsuzaki,Y.; Sugawara,K.; Furuse,Y.; Shimotai,Y.; Hongo,S.; Oshitani,H.; Mizuta,K.; Nishimura,H.
EPI816436	PB1	Japan	2007-Jan-17	EPI_ISL_230259	C/Yamagata/1/2007		Import from public-domain	Matsuzaki,Y.; Sugawara,K.; Furuse,Y.; Shimotai,Y.; Hongo,S.; Oshitani,H.; Mizuta,K.; Nishimura,H.
EPI813746	PB2	Japan	2007-Jan-17	EPI_ISL_230259	C/Yamagata/1/2007		Import from public-domain	Matsuzaki,Y.; Sugawara,K.; Furuse,Y.; Shimotai,Y.; Hongo,S.; Oshitani,H.; Mizuta,K.; Nishimura,H.
EPI813681	HE	Japan	2012-Jan-16	EPI_ISL_230266	C/Yamagata/1/2012		Import from public-domain	Matsuzaki,Y.; Sugawara,K.; Furuse,Y.; Shimotai,Y.; Hongo,S.; Oshitani,H.; Mizuta,K.; Nishimura,H.
EPI816715	MP	Japan	2012-Jan-16	EPI_ISL_230266	C/Yamagata/1/2012		Import from public-domain	Matsuzaki,Y.; Sugawara,K.; Furuse,Y.; Shimotai,Y.; Hongo,S.; Oshitani,H.; Mizuta,K.; Nishimura,H.
EPI816647	NP	Japan	2012-Jan-16	EPI_ISL_230266	C/Yamagata/1/2012		Import from public-domain	Matsuzaki,Y.; Sugawara,K.; Furuse,Y.; Shimotai,Y.; Hongo,S.; Oshitani,H.; Mizuta,K.; Nishimura,H.
EPI816784	NS	Japan	2012-Jan-16	EPI_ISL_230266	C/Yamagata/1/2012		Import from public-domain	Matsuzaki,Y.; Sugawara,K.; Furuse,Y.; Shimotai,Y.; Hongo,S.; Oshitani,H.; Mizuta,K.; Nishimura,H.
EPI816548	P3	Japan	2012-Jan-16	EPI_ISL_230266	C/Yamagata/1/2012		Import from public-domain	Matsuzaki,Y.; Sugawara,K.; Furuse,Y.; Shimotai,Y.; Hongo,S.; Oshitani,H.; Mizuta,K.; Nishimura,H.
EPI816444	PB1	Japan	2012-Jan-16	EPI_ISL_230266	C/Yamagata/1/2012		Import from public-domain	Matsuzaki,Y.; Sugawara,K.; Furuse,Y.; Shimotai,Y.; Hongo,S.; Oshitani,H.; Mizuta,K.; Nishimura,H.
EPI813754	PB2	Japan	2012-Jan-16	EPI_ISL_230266	C/Yamagata/1/2012		Import from public-domain	Matsuzaki,Y.; Sugawara,K.; Furuse,Y.; Shimotai,Y.; Hongo,S.; Oshitani,H.; Mizuta,K.; Nishimura,H.
EPI231906	HE	Japan	1986-Jan-01	EPI_ISL_66347	C/Yamagata/1/86		Import from public-domain	Matsuzaki,Y.
EPI232158	MP	Japan	1986-Jan-01	EPI_ISL_66347	C/Yamagata/1/86		Import from public-domain	Matsuzaki,Y.
EPI231556	NS	Japan	1986-Jan-01	EPI_ISL_66347	C/Yamagata/1/86		Import from public-domain	Matsuzaki,Y.
EPI231912	MP	Japan	1988-Jan-01	EPI_ISL_66414	C/Yamagata/1/88		Import from public-domain	Hongo,S.
EPI231911	NS	Japan	1988-Jan-01	EPI_ISL_66414	C/Yamagata/1/88		Import from public-domain	Hongo,S.
EPI231565	NS	Japan	1990-Jan-01	EPI_ISL_66354	C/Yamagata/1/90		Import from public-domain	Matsuzaki,Y.

Segment ID	Segment	Country	Collection date	Isolate-ID	Isolate name	Originating Lab	Submitting Lab	Authors
EPI231573	HE	Japan	1993-Apr-30	EPI_ISL_66362	C/Yamagata/1/93		Import from public-domain	Matsuzaki,Y.; Sugawara,K.; Furuse,Y.; Shimotai,Y.; Hongo,S.; Oshitani,H.; Mizuta,K.; Nishimura,H.
EPI231577	MP	Japan	1993-Apr-30	EPI_ISL_66362	C/Yamagata/1/93		Import from public-domain	Matsuzaki,Y.; Sugawara,K.; Furuse,Y.; Shimotai,Y.; Hongo,S.; Oshitani,H.; Mizuta,K.; Nishimura,H.
EPI231579	NP	Japan	1993-Apr-30	EPI_ISL_66362	C/Yamagata/1/93		Import from public-domain	Matsuzaki,Y.; Sugawara,K.; Furuse,Y.; Shimotai,Y.; Hongo,S.; Oshitani,H.; Mizuta,K.; Nishimura,H.
EPI231574	NS	Japan	1993-Apr-30	EPI_ISL_66362	C/Yamagata/1/93		Import from public-domain	Matsuzaki,Y.; Sugawara,K.; Furuse,Y.; Shimotai,Y.; Hongo,S.; Oshitani,H.; Mizuta,K.; Nishimura,H.
EPI816498	P3	Japan	1993-Apr-30	EPI_ISL_66362	C/Yamagata/1/93		Import from public-domain	Matsuzaki,Y.; Sugawara,K.; Furuse,Y.; Shimotai,Y.; Hongo,S.; Oshitani,H.; Mizuta,K.; Nishimura,H.
EPI816415	PB1	Japan	1993-Apr-30	EPI_ISL_66362	C/Yamagata/1/93		Import from public-domain	Matsuzaki,Y.; Sugawara,K.; Furuse,Y.; Shimotai,Y.; Hongo,S.; Oshitani,H.; Mizuta,K.; Nishimura,H.
EPI813741	PB2	Japan	1993-Apr-30	EPI_ISL_66362	C/Yamagata/1/93		Import from public-domain	Matsuzaki,Y.; Sugawara,K.; Furuse,Y.; Shimotai,Y.; Hongo,S.; Oshitani,H.; Mizuta,K.; Nishimura,H.
EPI231539	HE	Japan	1981-Jan-01	EPI_ISL_66338	C/Yamagata/10/81[g]		Import from public-domain	Buonagurio,D.A.; Nakada,S.; Desselberger,U.; Krystal,M.; Palese,P.
EPI816673	MP	Japan	1981-Mar-18	EPI_ISL_230279	C/Yamagata/10/1981[g]		Import from public-domain	Matsuzaki,Y.; Sugawara,K.; Furuse,Y.; Shimotai,Y.; Hongo,S.; Oshitani,H.; Mizuta,K.; Nishimura,H.
EPI816585	NP	Japan	1981-Mar-18	EPI_ISL_230279	C/Yamagata/10/1981[g]		Import from public-domain	Matsuzaki,Y.; Sugawara,K.; Furuse,Y.; Shimotai,Y.; Hongo,S.; Oshitani,H.; Mizuta,K.; Nishimura,H.
EPI816742	NS	Japan	1981-Mar-18	EPI_ISL_230279	C/Yamagata/10/1981[g]		Import from public-domain	Matsuzaki,Y.; Sugawara,K.; Furuse,Y.; Shimotai,Y.; Hongo,S.; Oshitani,H.; Mizuta,K.; Nishimura,H.
EPI816486	P3	Japan	1981-Mar-18	EPI_ISL_230279	C/Yamagata/10/1981[g]		Import from public-domain	Matsuzaki,Y.; Sugawara,K.; Furuse,Y.; Shimotai,Y.; Hongo,S.; Oshitani,H.; Mizuta,K.; Nishimura,H.
EPI813793	PB1	Japan	1981-Mar-18	EPI_ISL_230279	C/Yamagata/10/1981[g]		Import from public-domain	Matsuzaki,Y.; Sugawara,K.; Furuse,Y.; Shimotai,Y.; Hongo,S.; Oshitani,H.; Mizuta,K.; Nishimura,H.
EPI813696	PB2	Japan	1981-Mar-18	EPI_ISL_230279	C/Yamagata/10/1981[g]		Import from public-domain	Matsuzaki,Y.; Sugawara,K.; Furuse,Y.; Shimotai,Y.; Hongo,S.; Oshitani,H.; Mizuta,K.; Nishimura,H.
EPI231913	HE	Japan	1989-Aug-03	EPI_ISL_66360	C/Yamagata/10/89		Import from public-domain	Matsuzaki,Y.; Sugawara,K.; Furuse,Y.; Shimotai,Y.; Hongo,S.; Oshitani,H.; Mizuta,K.; Nishimura,H.
EPI232159	MP	Japan	1989-Aug-03	EPI_ISL_66360	C/Yamagata/10/89		Import from public-domain	Matsuzaki,Y.; Sugawara,K.; Furuse,Y.; Shimotai,Y.; Hongo,S.; Oshitani,H.; Mizuta,K.; Nishimura,H.
EPI816593	NP	Japan	1989-Aug-03	EPI_ISL_66360	C/Yamagata/10/89		Import from public-domain	Matsuzaki,Y.; Sugawara,K.; Furuse,Y.; Shimotai,Y.; Hongo,S.; Oshitani,H.; Mizuta,K.; Nishimura,H.
EPI231571	NS	Japan	1989-Aug-03	EPI_ISL_66360	C/Yamagata/10/89		Import from public-domain	Matsuzaki,Y.; Sugawara,K.; Furuse,Y.; Shimotai,Y.; Hongo,S.; Oshitani,H.; Mizuta,K.; Nishimura,H.
EPI816494	P3	Japan	1989-Aug-03	EPI_ISL_66360	C/Yamagata/10/89		Import from public-domain	Matsuzaki,Y.; Sugawara,K.; Furuse,Y.; Shimotai,Y.; Hongo,S.; Oshitani,H.; Mizuta,K.; Nishimura,H.
EPI816412	PB1	Japan	1989-Aug-03	EPI_ISL_66360	C/Yamagata/10/89		Import from public-domain	Matsuzaki,Y.; Sugawara,K.; Furuse,Y.; Shimotai,Y.; Hongo,S.; Oshitani,H.; Mizuta,K.; Nishimura,H.
EPI813739	PB2	Japan	1989-Aug-03	EPI_ISL_66360	C/Yamagata/10/89		Import from public-domain	Matsuzaki,Y.; Sugawara,K.; Furuse,Y.; Shimotai,Y.; Hongo,S.; Oshitani,H.; Mizuta,K.; Nishimura,H.
EPI813676	HE	Japan	2008-Apr-30	EPI_ISL_230261	C/Yamagata/11/2008		Import from public-domain	Matsuzaki,Y.; Sugawara,K.; Furuse,Y.; Shimotai,Y.; Hongo,S.; Oshitani,H.; Mizuta,K.; Nishimura,H.
EPI816708	MP	Japan	2008-Apr-30	EPI_ISL_230261	C/Yamagata/11/2008		Import from public-domain	Matsuzaki,Y.; Sugawara,K.; Furuse,Y.; Shimotai,Y.; Hongo,S.; Oshitani,H.; Mizuta,K.; Nishimura,H.
EPI816641	NP	Japan	2008-Apr-30	EPI_ISL_230261	C/Yamagata/11/2008		Import from public-domain	Matsuzaki,Y.; Sugawara,K.; Furuse,Y.; Shimotai,Y.; Hongo,S.; Oshitani,H.; Mizuta,K.; Nishimura,H.
EPI816777	NS	Japan	2008-Apr-30	EPI_ISL_230261	C/Yamagata/11/2008		Import from public-domain	Matsuzaki,Y.; Sugawara,K.; Furuse,Y.; Shimotai,Y.; Hongo,S.; Oshitani,H.; Mizuta,K.; Nishimura,H.
EPI816541	P3	Japan	2008-Apr-30	EPI_ISL_230261	C/Yamagata/11/2008		Import from public-domain	Matsuzaki,Y.; Sugawara,K.; Furuse,Y.; Shimotai,Y.; Hongo,S.; Oshitani,H.; Mizuta,K.; Nishimura,H.
EPI816438	PB1	Japan	2008-Apr-30	EPI_ISL_230261	C/Yamagata/11/2008		Import from public-domain	Matsuzaki,Y.; Sugawara,K.; Furuse,Y.; Shimotai,Y.; Hongo,S.; Oshitani,H.; Mizuta,K.; Nishimura,H.
EPI813748	PB2	Japan	2008-Apr-30	EPI_ISL_230261	C/Yamagata/11/2008		Import from public-domain	Matsuzaki,Y.; Sugawara,K.; Furuse,Y.; Shimotai,Y.; Hongo,S.; Oshitani,H.; Mizuta,K.; Nishimura,H.
EPI813671	HE	Japan	2006-Aug-08	EPI_ISL_230256	C/Yamagata/13/2006		Import from public-domain	Matsuzaki,Y.; Sugawara,K.; Furuse,Y.; Shimotai,Y.; Hongo,S.; Oshitani,H.; Mizuta,K.; Nishimura,H.
EPI816702	MP	Japan	2006-Aug-08	EPI_ISL_230256	C/Yamagata/13/2006		Import from public-domain	Matsuzaki,Y.; Sugawara,K.; Furuse,Y.; Shimotai,Y.; Hongo,S.; Oshitani,H.; Mizuta,K.; Nishimura,H.
EPI816636	NP	Japan	2006-Aug-08	EPI_ISL_230256	C/Yamagata/13/2006		Import from public-domain	Matsuzaki,Y.; Sugawara,K.; Furuse,Y.; Shimotai,Y.; Hongo,S.; Oshitani,H.; Mizuta,K.; Nishimura,H.
EPI816771	NS	Japan	2006-Aug-08	EPI_ISL_230256	C/Yamagata/13/2006		Import from public-domain	Matsuzaki,Y.; Sugawara,K.; Furuse,Y.; Shimotai,Y.; Hongo,S.; Oshitani,H.; Mizuta,K.; Nishimura,H.
EPI816535	P3	Japan	2006-Aug-08	EPI_ISL_230256	C/Yamagata/13/2006		Import from public-domain	Matsuzaki,Y.; Sugawara,K.; Furuse,Y.; Shimotai,Y.; Hongo,S.; Oshitani,H.; Mizuta,K.; Nishimura,H.

Segment ID	Segment	Country	Collection date	Isolate-ID	Isolate name	Originating Lab	Submitting Lab	Authors
EPI816433	PB1	Japan	2006-Aug-08	EPI_ISL_230256	C/Yamagata/13/2006		Import from public-domain	Matsuzaki,Y.; Sugawara,K.; Furuse,Y.; Shimotai,Y.; Hongo,S.; Oshitani,H.; Mizuta,K.; Nishimura,H.
EPI813743	PB2	Japan	2006-Aug-08	EPI_ISL_230256	C/Yamagata/13/2006		Import from public-domain	Matsuzaki,Y.; Sugawara,K.; Furuse,Y.; Shimotai,Y.; Hongo,S.; Oshitani,H.; Mizuta,K.; Nishimura,H.
EPI603680	HE	Japan	2014-May-14	EPI_ISL_182751	C/Yamagata/13/2014		Import from public-domain	Tanaka,S.; Aoki,Y.; Matoba,Y.; Yahagi,K.; Mizuta,K.; Itagaki,T.; Katsushima,F.; Katsushima,Y.; Matsuzaki,Y.; Sugawara,K.;
EPI816725	MP	Japan	2014-May-14	EPI_ISL_182751	C/Yamagata/13/2014		Import from public-domain	Tanaka,S.; Aoki,Y.; Matoba,Y.; Yahagi,K.; Mizuta,K.; Itagaki,T.; Katsushima,F.; Katsushima,Y.; Matsuzaki,Y.; Sugawara,K.;
EPI816657	NP	Japan	2014-May-14	EPI_ISL_182751	C/Yamagata/13/2014		Import from public-domain	Tanaka,S.; Aoki,Y.; Matoba,Y.; Yahagi,K.; Mizuta,K.; Itagaki,T.; Katsushima,F.; Katsushima,Y.; Matsuzaki,Y.; Sugawara,K.;
EPI816794	NS	Japan	2014-May-14	EPI_ISL_182751	C/Yamagata/13/2014		Import from public-domain	Tanaka,S.; Aoki,Y.; Matoba,Y.; Yahagi,K.; Mizuta,K.; Itagaki,T.; Katsushima,F.; Katsushima,Y.; Matsuzaki,Y.; Sugawara,K.;
EPI816558	P3	Japan	2014-May-14	EPI_ISL_182751	C/Yamagata/13/2014		Import from public-domain	Tanaka,S.; Aoki,Y.; Matoba,Y.; Yahagi,K.; Mizuta,K.; Itagaki,T.; Katsushima,F.; Katsushima,Y.; Matsuzaki,Y.; Sugawara,K.;
EPI816475	PB1	Japan	2014-May-14	EPI_ISL_182751	C/Yamagata/13/2014		Import from public-domain	Tanaka,S.; Aoki,Y.; Matoba,Y.; Yahagi,K.; Mizuta,K.; Itagaki,T.; Katsushima,F.; Katsushima,Y.; Matsuzaki,Y.; Sugawara,K.;
EPI813775	PB2	Japan	2014-May-14	EPI_ISL_182751	C/Yamagata/13/2014		Import from public-domain	Tanaka,S.; Aoki,Y.; Matoba,Y.; Yahagi,K.; Mizuta,K.; Itagaki,T.; Katsushima,F.; Katsushima,Y.; Matsuzaki,Y.; Sugawara,K.;
EPI231920	HE	Japan	1998-Jun-08	EPI_ISL_66415	C/Yamagata/13/98		Import from public-domain	Matsuzaki,Y.; Sugawara,K.; Furuse,Y.; Shimotai,Y.; Hongo,S.; Oshitani,H.; Mizuta,K.; Nishimura,H.
EPI231918	MP	Japan	1998-Jun-08	EPI_ISL_66415	C/Yamagata/13/98		Import from public-domain	Matsuzaki,Y.; Sugawara,K.; Furuse,Y.; Shimotai,Y.; Hongo,S.; Oshitani,H.; Mizuta,K.; Nishimura,H.
EPI816610	NP	Japan	1998-Jun-08	EPI_ISL_66415	C/Yamagata/13/98		Import from public-domain	Matsuzaki,Y.; Sugawara,K.; Furuse,Y.; Shimotai,Y.; Hongo,S.; Oshitani,H.; Mizuta,K.; Nishimura,H.
EPI231919	NS	Japan	1998-Jun-08	EPI_ISL_66415	C/Yamagata/13/98		Import from public-domain	Matsuzaki,Y.; Sugawara,K.; Furuse,Y.; Shimotai,Y.; Hongo,S.; Oshitani,H.; Mizuta,K.; Nishimura,H.
EPI816505	P3	Japan	1998-Jun-08	EPI_ISL_66415	C/Yamagata/13/98		Import from public-domain	Matsuzaki,Y.; Sugawara,K.; Furuse,Y.; Shimotai,Y.; Hongo,S.; Oshitani,H.; Mizuta,K.; Nishimura,H.
EPI816422	PB1	Japan	1998-Jun-08	EPI_ISL_66415	C/Yamagata/13/98		Import from public-domain	Matsuzaki,Y.; Sugawara,K.; Furuse,Y.; Shimotai,Y.; Hongo,S.; Oshitani,H.; Mizuta,K.; Nishimura,H.
EPI813724	PB2	Japan	1998-Jun-08	EPI_ISL_66415	C/Yamagata/13/98		Import from public-domain	Matsuzaki,Y.; Sugawara,K.; Furuse,Y.; Shimotai,Y.; Hongo,S.; Oshitani,H.; Mizuta,K.; Nishimura,H.
EPI816684	MP	Japan	2004-May-07	EPI_ISL_230282	C/Yamagata/14/2004		Import from public-domain	Matsuzaki,Y.; Sugawara,K.; Furuse,Y.; Shimotai,Y.; Hongo,S.; Oshitani,H.; Mizuta,K.; Nishimura,H.
EPI816620	NP	Japan	2004-May-07	EPI_ISL_230282	C/Yamagata/14/2004		Import from public-domain	Matsuzaki,Y.; Sugawara,K.; Furuse,Y.; Shimotai,Y.; Hongo,S.; Oshitani,H.; Mizuta,K.; Nishimura,H.
EPI816753	NS	Japan	2004-May-07	EPI_ISL_230282	C/Yamagata/14/2004		Import from public-domain	Matsuzaki,Y.; Sugawara,K.; Furuse,Y.; Shimotai,Y.; Hongo,S.; Oshitani,H.; Mizuta,K.; Nishimura,H.
EPI816517	P3	Japan	2004-May-07	EPI_ISL_230282	C/Yamagata/14/2004		Import from public-domain	Matsuzaki,Y.; Sugawara,K.; Furuse,Y.; Shimotai,Y.; Hongo,S.; Oshitani,H.; Mizuta,K.; Nishimura,H.
EPI813803	PB1	Japan	2004-May-07	EPI_ISL_230282	C/Yamagata/14/2004		Import from public-domain	Matsuzaki,Y.; Sugawara,K.; Furuse,Y.; Shimotai,Y.; Hongo,S.; Oshitani,H.; Mizuta,K.; Nishimura,H.
EPI813706	PB2	Japan	2004-May-07	EPI_ISL_230282	C/Yamagata/14/2004		Import from public-domain	Matsuzaki,Y.; Sugawara,K.; Furuse,Y.; Shimotai,Y.; Hongo,S.; Oshitani,H.; Mizuta,K.; Nishimura,H.
EPI603688	HE	Japan	2006-Aug-28	EPI_ISL_182759	C/Yamagata/14/2006		Import from public-domain	Tanaka,S.; Aoki,Y.; Matoba,Y.; Yahagi,K.; Mizuta,K.; Itagaki,T.; Katsushima,F.; Katsushima,Y.; Matsuzaki,Y.; Sugawara,K.;
EPI816703	MP	Japan	2006-Aug-28	EPI_ISL_182759	C/Yamagata/14/2006		Import from public-domain	Tanaka,S.; Aoki,Y.; Matoba,Y.; Yahagi,K.; Mizuta,K.; Itagaki,T.; Katsushima,F.; Katsushima,Y.; Matsuzaki,Y.; Sugawara,K.;
EPI816637	NP	Japan	2006-Aug-28	EPI_ISL_182759	C/Yamagata/14/2006		Import from public-domain	Tanaka,S.; Aoki,Y.; Matoba,Y.; Yahagi,K.; Mizuta,K.; Itagaki,T.; Katsushima,F.; Katsushima,Y.; Matsuzaki,Y.; Sugawara,K.;
EPI816772	NS	Japan	2006-Aug-28	EPI_ISL_182759	C/Yamagata/14/2006		Import from public-domain	Tanaka,S.; Aoki,Y.; Matoba,Y.; Yahagi,K.; Mizuta,K.; Itagaki,T.; Katsushima,F.; Katsushima,Y.; Matsuzaki,Y.; Sugawara,K.;
EPI816536	P3	Japan	2006-Aug-28	EPI_ISL_182759	C/Yamagata/14/2006		Import from public-domain	Tanaka,S.; Aoki,Y.; Matoba,Y.; Yahagi,K.; Mizuta,K.; Itagaki,T.; Katsushima,F.; Katsushima,Y.; Matsuzaki,Y.; Sugawara,K.;
EPI816472	PB1	Japan	2006-Aug-28	EPI_ISL_182759	C/Yamagata/14/2006		Import from public-domain	Tanaka,S.; Aoki,Y.; Matoba,Y.; Yahagi,K.; Mizuta,K.; Itagaki,T.; Katsushima,F.; Katsushima,Y.; Matsuzaki,Y.; Sugawara,K.;
EPI813770	PB2	Japan	2006-Aug-28	EPI_ISL_182759	C/Yamagata/14/2006		Import from public-domain	Tanaka,S.; Aoki,Y.; Matoba,Y.; Yahagi,K.; Mizuta,K.; Itagaki,T.; Katsushima,F.; Katsushima,Y.; Matsuzaki,Y.; Sugawara,K.;
EPI813684	HE	Japan	2012-Jun-01	EPI_ISL_230269	C/Yamagata/14/2012		Import from public-domain	Matsuzaki,Y.; Sugawara,K.; Furuse,Y.; Shimotai,Y.; Hongo,S.; Oshitani,H.; Mizuta,K.; Nishimura,H.
EPI816719	MP	Japan	2012-Jun-01	EPI_ISL_230269	C/Yamagata/14/2012		Import from public-domain	Matsuzaki,Y.; Sugawara,K.; Furuse,Y.; Shimotai,Y.; Hongo,S.; Oshitani,H.; Mizuta,K.; Nishimura,H.
EPI816651	NP	Japan	2012-Jun-01	EPI_ISL_230269	C/Yamagata/14/2012		Import from public-domain	Matsuzaki,Y.; Sugawara,K.; Furuse,Y.; Shimotai,Y.; Hongo,S.; Oshitani,H.; Mizuta,K.; Nishimura,H.
EPI816788	NS	Japan	2012-Jun-01	EPI_ISL_230269	C/Yamagata/14/2012		Import from public-domain	Matsuzaki,Y.; Sugawara,K.; Furuse,Y.; Shimotai,Y.; Hongo,S.; Oshitani,H.; Mizuta,K.; Nishimura,H.

Segment ID	Segment	Country	Collection date	Isolate-ID	Isolate name	Originating Lab	Submitting Lab	Authors
EPI816552	P3	Japan	2012-Jun-01	EPI_ISL_230269	C/Yamagata/14/2012		Import from public-domain	Matsuzaki,Y.; Sugawara,K.; Furuse,Y.; Shimotai,Y.; Hongo,S.; Oshitani,H.; Mizuta,K.; Nishimura,H.
EPI816448	PB1	Japan	2012-Jun-01	EPI_ISL_230269	C/Yamagata/14/2012		Import from public-domain	Matsuzaki,Y.; Sugawara,K.; Furuse,Y.; Shimotai,Y.; Hongo,S.; Oshitani,H.; Mizuta,K.; Nishimura,H.
EPI813758	PB2	Japan	2012-Jun-01	EPI_ISL_230269	C/Yamagata/14/2012		Import from public-domain	Matsuzaki,Y.; Sugawara,K.; Furuse,Y.; Shimotai,Y.; Hongo,S.; Oshitani,H.; Mizuta,K.; Nishimura,H.
EPI603681	HE	Japan	2014-May-14	EPI_ISL_182752	C/Yamagata/14/2014		Import from public-domain	Tanaka,S.; Aoki,Y.; Matoba,Y.; Yahagi,K.; Mizuta,K.; Itagaki,T.; Katsushima,F.; Katsushima,Y.; Matsuzaki,Y.
EPI228320	HE	Japan	2004-May-11	EPI_ISL_65156	C/Yamagata/15/2004		Import from public-domain	Matsuzaki,Y.; Sugawara,K.; Furuse,Y.; Shimotai,Y.; Hongo,S.; Oshitani,H.; Mizuta,K.; Nishimura,H.
EPI816685	MP	Japan	2004-May-11	EPI_ISL_65156	C/Yamagata/15/2004		Import from public-domain	Matsuzaki,Y.; Sugawara,K.; Furuse,Y.; Shimotai,Y.; Hongo,S.; Oshitani,H.; Mizuta,K.; Nishimura,H.
EPI369797	NP	Japan	2004-May-11	EPI_ISL_118432	C/Yamagata/15/2004		Import from public-domain	Matsuzaki,Y.; Ikeda,T.; Abiko,C.; Aoki,Y.; Mizuta,K.; Shimotai,Y.; Sugawara,K.; Hongo,S.
EPI816754	NS	Japan	2004-May-11	EPI_ISL_65156	C/Yamagata/15/2004		Import from public-domain	Matsuzaki,Y.; Sugawara,K.; Furuse,Y.; Shimotai,Y.; Hongo,S.; Oshitani,H.; Mizuta,K.; Nishimura,H.
EPI816518	P3	Japan	2004-May-11	EPI_ISL_65156	C/Yamagata/15/2004		Import from public-domain	Matsuzaki,Y.; Sugawara,K.; Furuse,Y.; Shimotai,Y.; Hongo,S.; Oshitani,H.; Mizuta,K.; Nishimura,H.
EPI816426	PB1	Japan	2004-May-11	EPI_ISL_65156	C/Yamagata/15/2004		Import from public-domain	Matsuzaki,Y.; Sugawara,K.; Furuse,Y.; Shimotai,Y.; Hongo,S.; Oshitani,H.; Mizuta,K.; Nishimura,H.
EPI813737	PB2	Japan	2004-May-11	EPI_ISL_65156	C/Yamagata/15/2004		Import from public-domain	Matsuzaki,Y.; Sugawara,K.; Furuse,Y.; Shimotai,Y.; Hongo,S.; Oshitani,H.; Mizuta,K.; Nishimura,H.
EPI603682	HE	Japan	2014-May-16	EPI_ISL_182753	C/Yamagata/15/2014		Import from public-domain	Tanaka,S.; Aoki,Y.; Matoba,Y.; Yahagi,K.; Mizuta,K.; Itagaki,T.; Katsushima,F.; Katsushima,Y.; Matsuzaki,Y.
EPI813677	HE	Japan	2008-May-19	EPI_ISL_230262	C/Yamagata/16/2008		Import from public-domain	Matsuzaki,Y.; Sugawara,K.; Furuse,Y.; Shimotai,Y.; Hongo,S.; Oshitani,H.; Mizuta,K.; Nishimura,H.
EPI816709	MP	Japan	2008-May-19	EPI_ISL_230262	C/Yamagata/16/2008		Import from public-domain	Matsuzaki,Y.; Sugawara,K.; Furuse,Y.; Shimotai,Y.; Hongo,S.; Oshitani,H.; Mizuta,K.; Nishimura,H.
EPI816642	NP	Japan	2008-May-19	EPI_ISL_230262	C/Yamagata/16/2008		Import from public-domain	Matsuzaki,Y.; Sugawara,K.; Furuse,Y.; Shimotai,Y.; Hongo,S.; Oshitani,H.; Mizuta,K.; Nishimura,H.
EPI816778	NS	Japan	2008-May-19	EPI_ISL_230262	C/Yamagata/16/2008		Import from public-domain	Matsuzaki,Y.; Sugawara,K.; Furuse,Y.; Shimotai,Y.; Hongo,S.; Oshitani,H.; Mizuta,K.; Nishimura,H.
EPI816542	P3	Japan	2008-May-19	EPI_ISL_230262	C/Yamagata/16/2008		Import from public-domain	Matsuzaki,Y.; Sugawara,K.; Furuse,Y.; Shimotai,Y.; Hongo,S.; Oshitani,H.; Mizuta,K.; Nishimura,H.
EPI816439	PB1	Japan	2008-May-19	EPI_ISL_230262	C/Yamagata/16/2008		Import from public-domain	Matsuzaki,Y.; Sugawara,K.; Furuse,Y.; Shimotai,Y.; Hongo,S.; Oshitani,H.; Mizuta,K.; Nishimura,H.
EPI813749	PB2	Japan	2008-May-19	EPI_ISL_230262	C/Yamagata/16/2008		Import from public-domain	Matsuzaki,Y.; Sugawara,K.; Furuse,Y.; Shimotai,Y.; Hongo,S.; Oshitani,H.; Mizuta,K.; Nishimura,H.
EPI603683	HE	Japan	2014-May-21	EPI_ISL_182754	C/Yamagata/16/2014		Import from public-domain	Tanaka,S.; Aoki,Y.; Matoba,Y.; Yahagi,K.; Mizuta,K.; Itagaki,T.; Katsushima,F.; Katsushima,Y.; Matsuzaki,Y.; Sugawara,K.;
EPI816726	MP	Japan	2014-May-21	EPI_ISL_182754	C/Yamagata/16/2014		Import from public-domain	Tanaka,S.; Aoki,Y.; Matoba,Y.; Yahagi,K.; Mizuta,K.; Itagaki,T.; Katsushima,F.; Katsushima,Y.; Matsuzaki,Y.; Sugawara,K.;
EPI816658	NP	Japan	2014-May-21	EPI_ISL_182754	C/Yamagata/16/2014		Import from public-domain	Tanaka,S.; Aoki,Y.; Matoba,Y.; Yahagi,K.; Mizuta,K.; Itagaki,T.; Katsushima,F.; Katsushima,Y.; Matsuzaki,Y.; Sugawara,K.;
EPI816795	NS	Japan	2014-May-21	EPI_ISL_182754	C/Yamagata/16/2014		Import from public-domain	Tanaka,S.; Aoki,Y.; Matoba,Y.; Yahagi,K.; Mizuta,K.; Itagaki,T.; Katsushima,F.; Katsushima,Y.; Matsuzaki,Y.; Sugawara,K.;
EPI816559	P3	Japan	2014-May-21	EPI_ISL_182754	C/Yamagata/16/2014		Import from public-domain	Tanaka,S.; Aoki,Y.; Matoba,Y.; Yahagi,K.; Mizuta,K.; Itagaki,T.; Katsushima,F.; Katsushima,Y.; Matsuzaki,Y.; Sugawara,K.;
EPI816476	PB1	Japan	2014-May-21	EPI_ISL_182754	C/Yamagata/16/2014		Import from public-domain	Tanaka,S.; Aoki,Y.; Matoba,Y.; Yahagi,K.; Mizuta,K.; Itagaki,T.; Katsushima,F.; Katsushima,Y.; Matsuzaki,Y.; Sugawara,K.;
EPI813773	PB2	Japan	2014-May-21	EPI_ISL_182754	C/Yamagata/16/2014		Import from public-domain	Tanaka,S.; Aoki,Y.; Matoba,Y.; Yahagi,K.; Mizuta,K.; Itagaki,T.; Katsushima,F.; Katsushima,Y.; Matsuzaki,Y.; Sugawara,K.;
EPI813658	HE	Japan	2004-May-14	EPI_ISL_230243	C/Yamagata/18/2004		Import from public-domain	Matsuzaki,Y.; Sugawara,K.; Furuse,Y.; Shimotai,Y.; Hongo,S.; Oshitani,H.; Mizuta,K.; Nishimura,H.
EPI816686	MP	Japan	2004-May-14	EPI_ISL_230243	C/Yamagata/18/2004		Import from public-domain	Matsuzaki,Y.; Sugawara,K.; Furuse,Y.; Shimotai,Y.; Hongo,S.; Oshitani,H.; Mizuta,K.; Nishimura,H.
EPI816621	NP	Japan	2004-May-14	EPI_ISL_230243	C/Yamagata/18/2004		Import from public-domain	Matsuzaki,Y.; Sugawara,K.; Furuse,Y.; Shimotai,Y.; Hongo,S.; Oshitani,H.; Mizuta,K.; Nishimura,H.
EPI816755	NS	Japan	2004-May-14	EPI_ISL_230243	C/Yamagata/18/2004		Import from public-domain	Matsuzaki,Y.; Sugawara,K.; Furuse,Y.; Shimotai,Y.; Hongo,S.; Oshitani,H.; Mizuta,K.; Nishimura,H.
EPI816519	P3	Japan	2004-May-14	EPI_ISL_230243	C/Yamagata/18/2004		Import from public-domain	Matsuzaki,Y.; Sugawara,K.; Furuse,Y.; Shimotai,Y.; Hongo,S.; Oshitani,H.; Mizuta,K.; Nishimura,H.
EPI813804	PB1	Japan	2004-May-14	EPI_ISL_230243	C/Yamagata/18/2004		Import from public-domain	Matsuzaki,Y.; Sugawara,K.; Furuse,Y.; Shimotai,Y.; Hongo,S.; Oshitani,H.; Mizuta,K.; Nishimura,H.
EPI813707	PB2	Japan	2004-May-14	EPI_ISL_230243	C/Yamagata/18/2004		Import from public-domain	Matsuzaki,Y.; Sugawara,K.; Furuse,Y.; Shimotai,Y.; Hongo,S.; Oshitani,H.; Mizuta,K.; Nishimura,H.

Segment ID	Segment	Country	Collection date	Isolate-ID	Isolate name	Originating Lab	Submitting Lab	Authors
EPI603689	HE	Japan	2008-Jun-25	EPI_ISL_182760	C/Yamagata/18/2008		Import from public-domain	Tanaka,S.; Aoki,Y.; Matoba,Y.; Yahagi,K.; Mizuta,K.; Itagaki,T.; Katsushima,F.; Katsushima,Y.; Matsuzaki,Y.; Sugawara,K.;
EPI816710	MP	Japan	2008-Jun-25	EPI_ISL_182760	C/Yamagata/18/2008		Import from public-domain	Tanaka,S.; Aoki,Y.; Matoba,Y.; Yahagi,K.; Mizuta,K.; Itagaki,T.; Katsushima,F.; Katsushima,Y.; Matsuzaki,Y.; Sugawara,K.;
EPI816643	NP	Japan	2008-Jun-25	EPI_ISL_182760	C/Yamagata/18/2008		Import from public-domain	Tanaka,S.; Aoki,Y.; Matoba,Y.; Yahagi,K.; Mizuta,K.; Itagaki,T.; Katsushima,F.; Katsushima,Y.; Matsuzaki,Y.; Sugawara,K.;
EPI816779	NS	Japan	2008-Jun-25	EPI_ISL_182760	C/Yamagata/18/2008		Import from public-domain	Tanaka,S.; Aoki,Y.; Matoba,Y.; Yahagi,K.; Mizuta,K.; Itagaki,T.; Katsushima,F.; Katsushima,Y.; Matsuzaki,Y.; Sugawara,K.;
EPI816543	P3	Japan	2008-Jun-25	EPI_ISL_182760	C/Yamagata/18/2008		Import from public-domain	Tanaka,S.; Aoki,Y.; Matoba,Y.; Yahagi,K.; Mizuta,K.; Itagaki,T.; Katsushima,F.; Katsushima,Y.; Matsuzaki,Y.; Sugawara,K.;
EPI816473	PB1	Japan	2008-Jun-25	EPI_ISL_182760	C/Yamagata/18/2008		Import from public-domain	Tanaka,S.; Aoki,Y.; Matoba,Y.; Yahagi,K.; Mizuta,K.; Itagaki,T.; Katsushima,F.; Katsushima,Y.; Matsuzaki,Y.; Sugawara,K.;
EPI813771	PB2	Japan	2008-Jun-25	EPI_ISL_182760	C/Yamagata/18/2008		Import from public-domain	Tanaka,S.; Aoki,Y.; Matoba,Y.; Yahagi,K.; Mizuta,K.; Itagaki,T.; Katsushima,F.; Katsushima,Y.; Matsuzaki,Y.; Sugawara,K.;
EPI231922	HE	Japan	2000-Jan-01	EPI_ISL_66416	C/Yamagata/2/2000		Import from public-domain	Matsuzaki,Y.
EPI231926	MP	Japan	2000-Jan-01	EPI_ISL_66416	C/Yamagata/2/2000		Import from public-domain	Matsuzaki,Y.
EPI231921	NS	Japan	2000-Jan-01	EPI_ISL_66416	C/Yamagata/2/2000		Import from public-domain	Matsuzaki,Y.
EPI813663	HE	Japan	2005-Jul-05	EPI_ISL_230248	C/Yamagata/2/2005		Import from public-domain	Matsuzaki,Y.; Sugawara,K.; Furuse,Y.; Shimotai,Y.; Hongo,S.; Oshitani,H.; Mizuta,K.; Nishimura,H.
EPI816694	MP	Japan	2005-Jul-05	EPI_ISL_230248	C/Yamagata/2/2005		Import from public-domain	Matsuzaki,Y.; Sugawara,K.; Furuse,Y.; Shimotai,Y.; Hongo,S.; Oshitani,H.; Mizuta,K.; Nishimura,H.
EPI816629	NP	Japan	2005-Jul-05	EPI_ISL_230248	C/Yamagata/2/2005		Import from public-domain	Matsuzaki,Y.; Sugawara,K.; Furuse,Y.; Shimotai,Y.; Hongo,S.; Oshitani,H.; Mizuta,K.; Nishimura,H.
EPI816763	NS	Japan	2005-Jul-05	EPI_ISL_230248	C/Yamagata/2/2005		Import from public-domain	Matsuzaki,Y.; Sugawara,K.; Furuse,Y.; Shimotai,Y.; Hongo,S.; Oshitani,H.; Mizuta,K.; Nishimura,H.
EPI816527	P3	Japan	2005-Jul-05	EPI_ISL_230248	C/Yamagata/2/2005		Import from public-domain	Matsuzaki,Y.; Sugawara,K.; Furuse,Y.; Shimotai,Y.; Hongo,S.; Oshitani,H.; Mizuta,K.; Nishimura,H.
EPI813809	PB1	Japan	2005-Jul-05	EPI_ISL_230248	C/Yamagata/2/2005		Import from public-domain	Matsuzaki,Y.; Sugawara,K.; Furuse,Y.; Shimotai,Y.; Hongo,S.; Oshitani,H.; Mizuta,K.; Nishimura,H.
EPI813712	PB2	Japan	2005-Jul-05	EPI_ISL_230248	C/Yamagata/2/2005		Import from public-domain	Matsuzaki,Y.; Sugawara,K.; Furuse,Y.; Shimotai,Y.; Hongo,S.; Oshitani,H.; Mizuta,K.; Nishimura,H.
EPI816712	MP	Japan	2010-Feb-09	EPI_ISL_230283	C/Yamagata/2/2010		Import from public-domain	Matsuzaki,Y.; Sugawara,K.; Furuse,Y.; Shimotai,Y.; Hongo,S.; Oshitani,H.; Mizuta,K.; Nishimura,H.
EPI369788	NP	Japan	2010-Feb-09	EPI_ISL_118424	C/Yamagata/2/2010		Import from public-domain	Matsuzaki,Y.; Ikeda,T.; Abiko,C.; Aoki,Y.; Mizuta,K.; Shimotai,Y.; Sugawara,K.; Hongo,S.
EPI816781	NS	Japan	2010-Feb-09	EPI_ISL_230283	C/Yamagata/2/2010		Import from public-domain	Matsuzaki,Y.; Sugawara,K.; Furuse,Y.; Shimotai,Y.; Hongo,S.; Oshitani,H.; Mizuta,K.; Nishimura,H.
EPI816545	P3	Japan	2010-Feb-09	EPI_ISL_230283	C/Yamagata/2/2010		Import from public-domain	Matsuzaki,Y.; Sugawara,K.; Furuse,Y.; Shimotai,Y.; Hongo,S.; Oshitani,H.; Mizuta,K.; Nishimura,H.
EPI816441	PB1	Japan	2010-Feb-09	EPI_ISL_230283	C/Yamagata/2/2010		Import from public-domain	Matsuzaki,Y.; Sugawara,K.; Furuse,Y.; Shimotai,Y.; Hongo,S.; Oshitani,H.; Mizuta,K.; Nishimura,H.
EPI813751	PB2	Japan	2010-Feb-09	EPI_ISL_230283	C/Yamagata/2/2010		Import from public-domain	Matsuzaki,Y.; Sugawara,K.; Furuse,Y.; Shimotai,Y.; Hongo,S.; Oshitani,H.; Mizuta,K.; Nishimura,H.
EPI231934	HE	Japan	1998-Jan-01	EPI_ISL_66417	C/Yamagata/2/98		Import from public-domain	Matsuzaki,Y.
EPI231930	MP	Japan	1998-Jan-01	EPI_ISL_66417	C/Yamagata/2/98		Import from public-domain	Matsuzaki,Y.
EPI231929	NS	Japan	1998-Jan-01	EPI_ISL_66417	C/Yamagata/2/98		Import from public-domain	Matsuzaki,Y.
EPI231935	HE	Japan	1999-Jan-01	EPI_ISL_66418	C/Yamagata/2/99		Import from public-domain	Matsuzaki,Y.
EPI231939	MP	Japan	1999-Jan-01	EPI_ISL_66418	C/Yamagata/2/99		Import from public-domain	Matsuzaki,Y.
EPI231941	NS	Japan	1999-Jan-01	EPI_ISL_66418	C/Yamagata/2/99		Import from public-domain	Matsuzaki,Y.
EPI228317	HE	Japan	2004-Jan-01	EPI_ISL_65153	C/Yamagata/20/2004		Import from public-domain	Matsuzaki,Y.
EPI813672	HE	Japan	2006-Nov-24	EPI_ISL_230257	C/Yamagata/20/2006		Import from public-domain	Matsuzaki,Y.; Sugawara,K.; Furuse,Y.; Shimotai,Y.; Hongo,S.; Oshitani,H.; Mizuta,K.; Nishimura,H.
EPI816704	MP	Japan	2006-Nov-24	EPI_ISL_230257	C/Yamagata/20/2006		Import from public-domain	Matsuzaki,Y.; Sugawara,K.; Furuse,Y.; Shimotai,Y.; Hongo,S.; Oshitani,H.; Mizuta,K.; Nishimura,H.
EPI816638	NP	Japan	2006-Nov-24	EPI_ISL_230257	C/Yamagata/20/2006		Import from public-domain	Matsuzaki,Y.; Sugawara,K.; Furuse,Y.; Shimotai,Y.; Hongo,S.; Oshitani,H.; Mizuta,K.; Nishimura,H.

Segment ID	Segment	Country	Collection date	Isolate-ID	Isolate name	Originating Lab	Submitting Lab	Authors
EPI816773	NS	Japan	2006-Nov-24	EPI_ISL_230257	C/Yamagata/20/2006		Import from public-domain	Matsuzaki,Y.; Sugawara,K.; Furuse,Y.; Shimotai,Y.; Hongo,S.; Oshitani,H.; Mizuta,K.; Nishimura,H.
EPI816537	P3	Japan	2006-Nov-24	EPI_ISL_230257	C/Yamagata/20/2006		Import from public-domain	Matsuzaki,Y.; Sugawara,K.; Furuse,Y.; Shimotai,Y.; Hongo,S.; Oshitani,H.; Mizuta,K.; Nishimura,H.
EPI816434	PB1	Japan	2006-Nov-24	EPI_ISL_230257	C/Yamagata/20/2006		Import from public-domain	Matsuzaki,Y.; Sugawara,K.; Furuse,Y.; Shimotai,Y.; Hongo,S.; Oshitani,H.; Mizuta,K.; Nishimura,H.
EPI813744	PB2	Japan	2006-Nov-24	EPI_ISL_230257	C/Yamagata/20/2006		Import from public-domain	Matsuzaki,Y.; Sugawara,K.; Furuse,Y.; Shimotai,Y.; Hongo,S.; Oshitani,H.; Mizuta,K.; Nishimura,H.
EPI231943	HE	Japan	1996-Jan-01	EPI_ISL_66419	C/Yamagata/20/96		Import from public-domain	Matsuzaki,Y.
EPI231945	MP	Japan	1996-Jan-01	EPI_ISL_66419	C/Yamagata/20/96		Import from public-domain	Matsuzaki,Y.
EPI231946	NS	Japan	1996-Jan-01	EPI_ISL_66419	C/Yamagata/20/96		Import from public-domain	Matsuzaki,Y.
EPI228318	HE	Japan	2004-Jan-01	EPI_ISL_65154	C/Yamagata/21/2004		Import from public-domain	Matsuzaki,Y.
EPI813659	HE	Japan	2004-May-28	EPI_ISL_230244	C/Yamagata/26/2004		Import from public-domain	Matsuzaki,Y.; Sugawara,K.; Furuse,Y.; Shimotai,Y.; Hongo,S.; Oshitani,H.; Mizuta,K.; Nishimura,H.
EPI816687	MP	Japan	2004-May-28	EPI_ISL_230244	C/Yamagata/26/2004		Import from public-domain	Matsuzaki,Y.; Sugawara,K.; Furuse,Y.; Shimotai,Y.; Hongo,S.; Oshitani,H.; Mizuta,K.; Nishimura,H.
EPI816622	NP	Japan	2004-May-28	EPI_ISL_230244	C/Yamagata/26/2004		Import from public-domain	Matsuzaki,Y.; Sugawara,K.; Furuse,Y.; Shimotai,Y.; Hongo,S.; Oshitani,H.; Mizuta,K.; Nishimura,H.
EPI816756	NS	Japan	2004-May-28	EPI_ISL_230244	C/Yamagata/26/2004		Import from public-domain	Matsuzaki,Y.; Sugawara,K.; Furuse,Y.; Shimotai,Y.; Hongo,S.; Oshitani,H.; Mizuta,K.; Nishimura,H.
EPI816520	P3	Japan	2004-May-28	EPI_ISL_230244	C/Yamagata/26/2004		Import from public-domain	Matsuzaki,Y.; Sugawara,K.; Furuse,Y.; Shimotai,Y.; Hongo,S.; Oshitani,H.; Mizuta,K.; Nishimura,H.
EPI813805	PB1	Japan	2004-May-28	EPI_ISL_230244	C/Yamagata/26/2004		Import from public-domain	Matsuzaki,Y.; Sugawara,K.; Furuse,Y.; Shimotai,Y.; Hongo,S.; Oshitani,H.; Mizuta,K.; Nishimura,H.
EPI813708	PB2	Japan	2004-May-28	EPI_ISL_230244	C/Yamagata/26/2004		Import from public-domain	Matsuzaki,Y.; Sugawara,K.; Furuse,Y.; Shimotai,Y.; Hongo,S.; Oshitani,H.; Mizuta,K.; Nishimura,H.
EPI231538	HE	Japan	1981-Mar-18	EPI_ISL_66337	C/Yamagata/26/81		Import from public-domain	Matsuzaki,Y.; Sugawara,K.; Furuse,Y.; Shimotai,Y.; Hongo,S.; Oshitani,H.; Mizuta,K.; Nishimura,H.
EPI232166	MP	Japan	1981-Mar-18	EPI_ISL_66337	C/Yamagata/26/81		Import from public-domain	Matsuzaki,Y.; Sugawara,K.; Furuse,Y.; Shimotai,Y.; Hongo,S.; Oshitani,H.; Mizuta,K.; Nishimura,H.
EPI816586	NP	Japan	1981-Mar-18	EPI_ISL_66337	C/Yamagata/26/81		Import from public-domain	Matsuzaki,Y.; Sugawara,K.; Furuse,Y.; Shimotai,Y.; Hongo,S.; Oshitani,H.; Mizuta,K.; Nishimura,H.
EPI231543	NS	Japan	1981-Mar-18	EPI_ISL_66337	C/Yamagata/26/81		Import from public-domain	Matsuzaki,Y.; Sugawara,K.; Furuse,Y.; Shimotai,Y.; Hongo,S.; Oshitani,H.; Mizuta,K.; Nishimura,H.
EPI816487	P3	Japan	1981-Mar-18	EPI_ISL_66337	C/Yamagata/26/81		Import from public-domain	Matsuzaki,Y.; Sugawara,K.; Furuse,Y.; Shimotai,Y.; Hongo,S.; Oshitani,H.; Mizuta,K.; Nishimura,H.
EPI815441	PB1	Japan	1981-Mar-18	EPI_ISL_66337	C/Yamagata/26/81		Import from public-domain	Matsuzaki,Y.; Sugawara,K.; Furuse,Y.; Shimotai,Y.; Hongo,S.; Oshitani,H.; Mizuta,K.; Nishimura,H.
EPI813720	PB2	Japan	1981-Mar-18	EPI_ISL_66337	C/Yamagata/26/81		Import from public-domain	Matsuzaki,Y.; Sugawara,K.; Furuse,Y.; Shimotai,Y.; Hongo,S.; Oshitani,H.; Mizuta,K.; Nishimura,H.
EPI228321	HE	Japan	2004-May-24	EPI_ISL_65157	C/Yamagata/27/2004		Import from public-domain	Matsuzaki,Y.; Sugawara,K.; Furuse,Y.; Shimotai,Y.; Hongo,S.; Oshitani,H.; Mizuta,K.; Nishimura,H.
EPI816688	MP	Japan	2004-May-24	EPI_ISL_65157	C/Yamagata/27/2004		Import from public-domain	Matsuzaki,Y.; Sugawara,K.; Furuse,Y.; Shimotai,Y.; Hongo,S.; Oshitani,H.; Mizuta,K.; Nishimura,H.
EPI816623	NP	Japan	2004-May-24	EPI_ISL_65157	C/Yamagata/27/2004		Import from public-domain	Matsuzaki,Y.; Sugawara,K.; Furuse,Y.; Shimotai,Y.; Hongo,S.; Oshitani,H.; Mizuta,K.; Nishimura,H.
EPI816757	NS	Japan	2004-May-24	EPI_ISL_65157	C/Yamagata/27/2004		Import from public-domain	Matsuzaki,Y.; Sugawara,K.; Furuse,Y.; Shimotai,Y.; Hongo,S.; Oshitani,H.; Mizuta,K.; Nishimura,H.
EPI816521	P3	Japan	2004-May-24	EPI_ISL_65157	C/Yamagata/27/2004		Import from public-domain	Matsuzaki,Y.; Sugawara,K.; Furuse,Y.; Shimotai,Y.; Hongo,S.; Oshitani,H.; Mizuta,K.; Nishimura,H.
EPI816428	PB1	Japan	2004-May-24	EPI_ISL_65157	C/Yamagata/27/2004		Import from public-domain	Matsuzaki,Y.; Sugawara,K.; Furuse,Y.; Shimotai,Y.; Hongo,S.; Oshitani,H.; Mizuta,K.; Nishimura,H.
EPI813738	PB2	Japan	2004-May-24	EPI_ISL_65157	C/Yamagata/27/2004		Import from public-domain	Matsuzaki,Y.; Sugawara,K.; Furuse,Y.; Shimotai,Y.; Hongo,S.; Oshitani,H.; Mizuta,K.; Nishimura,H.
EPI813685	HE	Japan	2012-Jul-12	EPI_ISL_230270	C/Yamagata/28/2012		Import from public-domain	Matsuzaki,Y.; Sugawara,K.; Furuse,Y.; Shimotai,Y.; Hongo,S.; Oshitani,H.; Mizuta,K.; Nishimura,H.
EPI816720	MP	Japan	2012-Jul-12	EPI_ISL_230270	C/Yamagata/28/2012		Import from public-domain	Matsuzaki,Y.; Sugawara,K.; Furuse,Y.; Shimotai,Y.; Hongo,S.; Oshitani,H.; Mizuta,K.; Nishimura,H.
EPI816652	NP	Japan	2012-Jul-12	EPI_ISL_230270	C/Yamagata/28/2012		Import from public-domain	Matsuzaki,Y.; Sugawara,K.; Furuse,Y.; Shimotai,Y.; Hongo,S.; Oshitani,H.; Mizuta,K.; Nishimura,H.
EPI816789	NS	Japan	2012-Jul-12	EPI_ISL_230270	C/Yamagata/28/2012		Import from public-domain	Matsuzaki,Y.; Sugawara,K.; Furuse,Y.; Shimotai,Y.; Hongo,S.; Oshitani,H.; Mizuta,K.; Nishimura,H.

Segment ID	Segment	Country	Collection date	Isolate-ID	Isolate name	Originating Lab	Submitting Lab	Authors
EPI816553	P3	Japan	2012-Jul-12	EPI_ISL_230270	C/Yamagata/28/2012		Import from public-domain	Matsuzaki,Y.; Sugawara,K.; Furuse,Y.; Shimotai,Y.; Hongo,S.; Oshitani,H.; Mizuta,K.; Nishimura,H.
EPI816449	PB1	Japan	2012-Jul-12	EPI_ISL_230270	C/Yamagata/28/2012		Import from public-domain	Matsuzaki,Y.; Sugawara,K.; Furuse,Y.; Shimotai,Y.; Hongo,S.; Oshitani,H.; Mizuta,K.; Nishimura,H.
EPI813759	PB2	Japan	2012-Jul-12	EPI_ISL_230270	C/Yamagata/28/2012		Import from public-domain	Matsuzaki,Y.; Sugawara,K.; Furuse,Y.; Shimotai,Y.; Hongo,S.; Oshitani,H.; Mizuta,K.; Nishimura,H.
EPI816721	MP	Japan	2012-Jul-30	EPI_ISL_230285	C/Yamagata/29/2012		Import from public-domain	Matsuzaki,Y.; Sugawara,K.; Furuse,Y.; Shimotai,Y.; Hongo,S.; Oshitani,H.; Mizuta,K.; Nishimura,H.
EPI816653	NP	Japan	2012-Jul-30	EPI_ISL_230285	C/Yamagata/29/2012		Import from public-domain	Matsuzaki,Y.; Sugawara,K.; Furuse,Y.; Shimotai,Y.; Hongo,S.; Oshitani,H.; Mizuta,K.; Nishimura,H.
EPI816790	NS	Japan	2012-Jul-30	EPI_ISL_230285	C/Yamagata/29/2012		Import from public-domain	Matsuzaki,Y.; Sugawara,K.; Furuse,Y.; Shimotai,Y.; Hongo,S.; Oshitani,H.; Mizuta,K.; Nishimura,H.
EPI816554	P3	Japan	2012-Jul-30	EPI_ISL_230285	C/Yamagata/29/2012		Import from public-domain	Matsuzaki,Y.; Sugawara,K.; Furuse,Y.; Shimotai,Y.; Hongo,S.; Oshitani,H.; Mizuta,K.; Nishimura,H.
EPI816450	PB1	Japan	2012-Jul-30	EPI_ISL_230285	C/Yamagata/29/2012		Import from public-domain	Matsuzaki,Y.; Sugawara,K.; Furuse,Y.; Shimotai,Y.; Hongo,S.; Oshitani,H.; Mizuta,K.; Nishimura,H.
EPI813760	PB2	Japan	2012-Jul-30	EPI_ISL_230285	C/Yamagata/29/2012		Import from public-domain	Matsuzaki,Y.; Sugawara,K.; Furuse,Y.; Shimotai,Y.; Hongo,S.; Oshitani,H.; Mizuta,K.; Nishimura,H.
EPI231950	HE	Japan	2000-Feb-02	EPI_ISL_66420	C/Yamagata/3/2000		Import from public-domain	Matsuzaki,Y.; Sugawara,K.; Furuse,Y.; Shimotai,Y.; Hongo,S.; Oshitani,H.; Mizuta,K.; Nishimura,H.
EPI231953	MP	Japan	2000-Feb-02	EPI_ISL_66420	C/Yamagata/3/2000		Import from public-domain	Matsuzaki,Y.; Sugawara,K.; Furuse,Y.; Shimotai,Y.; Hongo,S.; Oshitani,H.; Mizuta,K.; Nishimura,H.
EPI231954	NP	Japan	2000-Feb-02	EPI_ISL_66420	C/Yamagata/3/2000		Import from public-domain	Matsuzaki,Y.; Sugawara,K.; Furuse,Y.; Shimotai,Y.; Hongo,S.; Oshitani,H.; Mizuta,K.; Nishimura,H.
EPI231949	NS	Japan	2000-Feb-02	EPI_ISL_66420	C/Yamagata/3/2000		Import from public-domain	Matsuzaki,Y.; Sugawara,K.; Furuse,Y.; Shimotai,Y.; Hongo,S.; Oshitani,H.; Mizuta,K.; Nishimura,H.
EPI816507	P3	Japan	2000-Feb-02	EPI_ISL_66420	C/Yamagata/3/2000		Import from public-domain	Matsuzaki,Y.; Sugawara,K.; Furuse,Y.; Shimotai,Y.; Hongo,S.; Oshitani,H.; Mizuta,K.; Nishimura,H.
EPI816424	PB1	Japan	2000-Feb-02	EPI_ISL_66420	C/Yamagata/3/2000		Import from public-domain	Matsuzaki,Y.; Sugawara,K.; Furuse,Y.; Shimotai,Y.; Hongo,S.; Oshitani,H.; Mizuta,K.; Nishimura,H.
EPI813734	PB2	Japan	2000-Feb-02	EPI_ISL_66420	C/Yamagata/3/2000		Import from public-domain	Matsuzaki,Y.; Sugawara,K.; Furuse,Y.; Shimotai,Y.; Hongo,S.; Oshitani,H.; Mizuta,K.; Nishimura,H.
EPI228319	HE	Japan	2004-Apr-16	EPI_ISL_65155	C/Yamagata/3/2004		Import from public-domain	Matsuzaki,Y.; Sugawara,K.; Furuse,Y.; Shimotai,Y.; Hongo,S.; Oshitani,H.; Mizuta,K.; Nishimura,H.
EPI816683	MP	Japan	2004-Apr-16	EPI_ISL_65155	C/Yamagata/3/2004		Import from public-domain	Matsuzaki,Y.; Sugawara,K.; Furuse,Y.; Shimotai,Y.; Hongo,S.; Oshitani,H.; Mizuta,K.; Nishimura,H.
EPI816619	NP	Japan	2004-Apr-16	EPI_ISL_65155	C/Yamagata/3/2004		Import from public-domain	Matsuzaki,Y.; Sugawara,K.; Furuse,Y.; Shimotai,Y.; Hongo,S.; Oshitani,H.; Mizuta,K.; Nishimura,H.
EPI816752	NS	Japan	2004-Apr-16	EPI_ISL_65155	C/Yamagata/3/2004		Import from public-domain	Matsuzaki,Y.; Sugawara,K.; Furuse,Y.; Shimotai,Y.; Hongo,S.; Oshitani,H.; Mizuta,K.; Nishimura,H.
EPI816516	P3	Japan	2004-Apr-16	EPI_ISL_65155	C/Yamagata/3/2004		Import from public-domain	Matsuzaki,Y.; Sugawara,K.; Furuse,Y.; Shimotai,Y.; Hongo,S.; Oshitani,H.; Mizuta,K.; Nishimura,H.
EPI816427	PB1	Japan	2004-Apr-16	EPI_ISL_65155	C/Yamagata/3/2004		Import from public-domain	Matsuzaki,Y.; Sugawara,K.; Furuse,Y.; Shimotai,Y.; Hongo,S.; Oshitani,H.; Mizuta,K.; Nishimura,H.
EPI813736	PB2	Japan	2004-Apr-16	EPI_ISL_65155	C/Yamagata/3/2004		Import from public-domain	Matsuzaki,Y.; Sugawara,K.; Furuse,Y.; Shimotai,Y.; Hongo,S.; Oshitani,H.; Mizuta,K.; Nishimura,H.
EPI813664	HE	Japan	2005-Sep-07	EPI_ISL_230249	C/Yamagata/3/2005		Import from public-domain	Matsuzaki,Y.; Sugawara,K.; Furuse,Y.; Shimotai,Y.; Hongo,S.; Oshitani,H.; Mizuta,K.; Nishimura,H.
EPI816695	MP	Japan	2005-Sep-07	EPI_ISL_230249	C/Yamagata/3/2005		Import from public-domain	Matsuzaki,Y.; Sugawara,K.; Furuse,Y.; Shimotai,Y.; Hongo,S.; Oshitani,H.; Mizuta,K.; Nishimura,H.
EPI369792	NP	Japan	2005-Sep-07	EPI_ISL_118427	C/Yamagata/3/2005		Import from public-domain	Matsuzaki,Y.; Ikeda,T.; Abiko,C.; Aoki,Y.; Mizuta,K.; Shimotai,Y.; Sugawara,K.; Hongo,S.
EPI816764	NS	Japan	2005-Sep-07	EPI_ISL_230249	C/Yamagata/3/2005		Import from public-domain	Matsuzaki,Y.; Sugawara,K.; Furuse,Y.; Shimotai,Y.; Hongo,S.; Oshitani,H.; Mizuta,K.; Nishimura,H.
EPI816528	P3	Japan	2005-Sep-07	EPI_ISL_230249	C/Yamagata/3/2005		Import from public-domain	Matsuzaki,Y.; Sugawara,K.; Furuse,Y.; Shimotai,Y.; Hongo,S.; Oshitani,H.; Mizuta,K.; Nishimura,H.
EPI813810	PB1	Japan	2005-Sep-07	EPI_ISL_230249	C/Yamagata/3/2005		Import from public-domain	Matsuzaki,Y.; Sugawara,K.; Furuse,Y.; Shimotai,Y.; Hongo,S.; Oshitani,H.; Mizuta,K.; Nishimura,H.
EPI813713	PB2	Japan	2005-Sep-07	EPI_ISL_230249	C/Yamagata/3/2005		Import from public-domain	Matsuzaki,Y.; Sugawara,K.; Furuse,Y.; Shimotai,Y.; Hongo,S.; Oshitani,H.; Mizuta,K.; Nishimura,H.
EPI813675	HE	Japan	2008-Mar-26	EPI_ISL_230260	C/Yamagata/3/2008		Import from public-domain	Matsuzaki,Y.; Sugawara,K.; Furuse,Y.; Shimotai,Y.; Hongo,S.; Oshitani,H.; Mizuta,K.; Nishimura,H.
EPI816707	MP	Japan	2008-Mar-26	EPI_ISL_230260	C/Yamagata/3/2008		Import from public-domain	Matsuzaki,Y.; Sugawara,K.; Furuse,Y.; Shimotai,Y.; Hongo,S.; Oshitani,H.; Mizuta,K.; Nishimura,H.
EPI816640	NP	Japan	2008-Mar-26	EPI_ISL_230260	C/Yamagata/3/2008		Import from public-domain	Matsuzaki,Y.; Sugawara,K.; Furuse,Y.; Shimotai,Y.; Hongo,S.; Oshitani,H.; Mizuta,K.; Nishimura,H.

Segment ID	Segment	Country	Collection date	Isolate-ID	Isolate name	Originating Lab	Submitting Lab	Authors
EPI816776	NS	Japan	2008-Mar-26	EPI_ISL_230260	C/Yamagata/3/2008		Import from public-domain	Matsuzaki,Y.; Sugawara,K.; Furuse,Y.; Shimotai,Y.; Hongo,S.; Oshitani,H.; Mizuta,K.; Nishimura,H.
EPI816540	P3	Japan	2008-Mar-26	EPI_ISL_230260	C/Yamagata/3/2008		Import from public-domain	Matsuzaki,Y.; Sugawara,K.; Furuse,Y.; Shimotai,Y.; Hongo,S.; Oshitani,H.; Mizuta,K.; Nishimura,H.
EPI816437	PB1	Japan	2008-Mar-26	EPI_ISL_230260	C/Yamagata/3/2008		Import from public-domain	Matsuzaki,Y.; Sugawara,K.; Furuse,Y.; Shimotai,Y.; Hongo,S.; Oshitani,H.; Mizuta,K.; Nishimura,H.
EPI813747	PB2	Japan	2008-Mar-26	EPI_ISL_230260	C/Yamagata/3/2008		Import from public-domain	Matsuzaki,Y.; Sugawara,K.; Furuse,Y.; Shimotai,Y.; Hongo,S.; Oshitani,H.; Mizuta,K.; Nishimura,H.
EPI813682	HE	Japan	2012-Feb-07	EPI_ISL_230267	C/Yamagata/3/2012		Import from public-domain	Matsuzaki,Y.; Sugawara,K.; Furuse,Y.; Shimotai,Y.; Hongo,S.; Oshitani,H.; Mizuta,K.; Nishimura,H.
EPI816716	MP	Japan	2012-Feb-07	EPI_ISL_230267	C/Yamagata/3/2012		Import from public-domain	Matsuzaki,Y.; Sugawara,K.; Furuse,Y.; Shimotai,Y.; Hongo,S.; Oshitani,H.; Mizuta,K.; Nishimura,H.
EPI816648	NP	Japan	2012-Feb-07	EPI_ISL_230267	C/Yamagata/3/2012		Import from public-domain	Matsuzaki,Y.; Sugawara,K.; Furuse,Y.; Shimotai,Y.; Hongo,S.; Oshitani,H.; Mizuta,K.; Nishimura,H.
EPI816785	NS	Japan	2012-Feb-07	EPI_ISL_230267	C/Yamagata/3/2012		Import from public-domain	Matsuzaki,Y.; Sugawara,K.; Furuse,Y.; Shimotai,Y.; Hongo,S.; Oshitani,H.; Mizuta,K.; Nishimura,H.
EPI816549	P3	Japan	2012-Feb-07	EPI_ISL_230267	C/Yamagata/3/2012		Import from public-domain	Matsuzaki,Y.; Sugawara,K.; Furuse,Y.; Shimotai,Y.; Hongo,S.; Oshitani,H.; Mizuta,K.; Nishimura,H.
EPI816445	PB1	Japan	2012-Feb-07	EPI_ISL_230267	C/Yamagata/3/2012		Import from public-domain	Matsuzaki,Y.; Sugawara,K.; Furuse,Y.; Shimotai,Y.; Hongo,S.; Oshitani,H.; Mizuta,K.; Nishimura,H.
EPI813755	PB2	Japan	2012-Feb-07	EPI_ISL_230267	C/Yamagata/3/2012		Import from public-domain	Matsuzaki,Y.; Sugawara,K.; Furuse,Y.; Shimotai,Y.; Hongo,S.; Oshitani,H.; Mizuta,K.; Nishimura,H.
EPI231956	HE	Japan	1988-Jan-01	EPI_ISL_66421	C/Yamagata/3/88		Import from public-domain	Muraki,Y.
EPI231960	HE	Japan	1996-Jan-01	EPI_ISL_66422	C/Yamagata/3/96		Import from public-domain	Matsuzaki,Y.
EPI231957	MP	Japan	1996-Jan-01	EPI_ISL_66422	C/Yamagata/3/96		Import from public-domain	Matsuzaki,Y.
EPI231962	NS	Japan	1996-Jan-01	EPI_ISL_66422	C/Yamagata/3/96		Import from public-domain	Matsuzaki,Y.
EPI603684	HE	Japan	2014-Jun-04	EPI_ISL_182755	C/Yamagata/30/2014		Import from public-domain	Tanaka,S.; Aoki,Y.; Matoba,Y.; Yahagi,K.; Mizuta,K.; Itagaki,T.; Katsushima,F.; Katsushima,Y.; Matsuzaki,Y.; Sugawara,K.;
EPI816727	MP	Japan	2014-Jun-04	EPI_ISL_182755	C/Yamagata/30/2014		Import from public-domain	Tanaka,S.; Aoki,Y.; Matoba,Y.; Yahagi,K.; Mizuta,K.; Itagaki,T.; Katsushima,F.; Katsushima,Y.; Matsuzaki,Y.; Sugawara,K.;
EPI816659	NP	Japan	2014-Jun-04	EPI_ISL_182755	C/Yamagata/30/2014		Import from public-domain	Tanaka,S.; Aoki,Y.; Matoba,Y.; Yahagi,K.; Mizuta,K.; Itagaki,T.; Katsushima,F.; Katsushima,Y.; Matsuzaki,Y.; Sugawara,K.;
EPI816796	NS	Japan	2014-Jun-04	EPI_ISL_182755	C/Yamagata/30/2014		Import from public-domain	Tanaka,S.; Aoki,Y.; Matoba,Y.; Yahagi,K.; Mizuta,K.; Itagaki,T.; Katsushima,F.; Katsushima,Y.; Matsuzaki,Y.; Sugawara,K.;
EPI816560	P3	Japan	2014-Jun-04	EPI_ISL_182755	C/Yamagata/30/2014		Import from public-domain	Tanaka,S.; Aoki,Y.; Matoba,Y.; Yahagi,K.; Mizuta,K.; Itagaki,T.; Katsushima,F.; Katsushima,Y.; Matsuzaki,Y.; Sugawara,K.;
EPI816477	PB1	Japan	2014-Jun-04	EPI_ISL_182755	C/Yamagata/30/2014		Import from public-domain	Tanaka,S.; Aoki,Y.; Matoba,Y.; Yahagi,K.; Mizuta,K.; Itagaki,T.; Katsushima,F.; Katsushima,Y.; Matsuzaki,Y.; Sugawara,K.;
EPI813774	PB2	Japan	2014-Jun-04	EPI_ISL_182755	C/Yamagata/30/2014		Import from public-domain	Tanaka,S.; Aoki,Y.; Matoba,Y.; Yahagi,K.; Mizuta,K.; Itagaki,T.; Katsushima,F.; Katsushima,Y.; Matsuzaki,Y.; Sugawara,K.;
EPI603685	HE	Japan	2014-Jun-17	EPI_ISL_182756	C/Yamagata/32/2014		Import from public-domain	Tanaka,S.; Aoki,Y.; Matoba,Y.; Yahagi,K.; Mizuta,K.; Itagaki,T.; Katsushima,F.; Katsushima,Y.; Matsuzaki,Y.; Sugawara,K.;
EPI816728	MP	Japan	2014-Jun-17	EPI_ISL_182756	C/Yamagata/32/2014		Import from public-domain	Tanaka,S.; Aoki,Y.; Matoba,Y.; Yahagi,K.; Mizuta,K.; Itagaki,T.; Katsushima,F.; Katsushima,Y.; Matsuzaki,Y.; Sugawara,K.;
EPI816660	NP	Japan	2014-Jun-17	EPI_ISL_182756	C/Yamagata/32/2014		Import from public-domain	Tanaka,S.; Aoki,Y.; Matoba,Y.; Yahagi,K.; Mizuta,K.; Itagaki,T.; Katsushima,F.; Katsushima,Y.; Matsuzaki,Y.; Sugawara,K.;
EPI816797	NS	Japan	2014-Jun-17	EPI_ISL_182756	C/Yamagata/32/2014		Import from public-domain	Tanaka,S.; Aoki,Y.; Matoba,Y.; Yahagi,K.; Mizuta,K.; Itagaki,T.; Katsushima,F.; Katsushima,Y.; Matsuzaki,Y.; Sugawara,K.;
EPI816561	P3	Japan	2014-Jun-17	EPI_ISL_182756	C/Yamagata/32/2014		Import from public-domain	Tanaka,S.; Aoki,Y.; Matoba,Y.; Yahagi,K.; Mizuta,K.; Itagaki,T.; Katsushima,F.; Katsushima,Y.; Matsuzaki,Y.; Sugawara,K.;
EPI816478	PB1	Japan	2014-Jun-17	EPI_ISL_182756	C/Yamagata/32/2014		Import from public-domain	Tanaka,S.; Aoki,Y.; Matoba,Y.; Yahagi,K.; Mizuta,K.; Itagaki,T.; Katsushima,F.; Katsushima,Y.; Matsuzaki,Y.; Sugawara,K.;
EPI813776	PB2	Japan	2014-Jun-17	EPI_ISL_182756	C/Yamagata/32/2014		Import from public-domain	Tanaka,S.; Aoki,Y.; Matoba,Y.; Yahagi,K.; Mizuta,K.; Itagaki,T.; Katsushima,F.; Katsushima,Y.; Matsuzaki,Y.; Sugawara,K.;
EPI603686	HE	Japan	2014-Jul-22	EPI_ISL_182757	C/Yamagata/33/2014		Import from public-domain	Tanaka,S.; Aoki,Y.; Matoba,Y.; Yahagi,K.; Mizuta,K.; Itagaki,T.; Katsushima,F.; Katsushima,Y.; Matsuzaki,Y.; Sugawara,K.;
EPI816729	MP	Japan	2014-Jul-22	EPI_ISL_182757	C/Yamagata/33/2014		Import from public-domain	Tanaka,S.; Aoki,Y.; Matoba,Y.; Yahagi,K.; Mizuta,K.; Itagaki,T.; Katsushima,F.; Katsushima,Y.; Matsuzaki,Y.; Sugawara,K.;
EPI816661	NP	Japan	2014-Jul-22	EPI_ISL_182757	C/Yamagata/33/2014		Import from public-domain	Tanaka,S.; Aoki,Y.; Matoba,Y.; Yahagi,K.; Mizuta,K.; Itagaki,T.; Katsushima,F.; Katsushima,Y.; Matsuzaki,Y.; Sugawara,K.;
EPI816798	NS	Japan	2014-Jul-22	EPI_ISL_182757	C/Yamagata/33/2014		Import from public-domain	Tanaka,S.; Aoki,Y.; Matoba,Y.; Yahagi,K.; Mizuta,K.; Itagaki,T.; Katsushima,F.; Katsushima,Y.; Matsuzaki,Y.; Sugawara,K.;

Segment ID	Segment	Country	Collection date	Isolate-ID	Isolate name	Originating Lab	Submitting Lab	Authors
EPI816562	P3	Japan	2014-Jul-22	EPI_ISL_182757	C/Yamagata/33/2014		Import from public-domain	Tanaka,S.; Aoki,Y.; Matoba,Y.; Yahagi,K.; Mizuta,K.; Itagaki,T.; Katsushima,F.; Katsushima,Y.; Matsuzaki,Y.; Sugawara,K.;
EPI816479	PB1	Japan	2014-Jul-22	EPI_ISL_182757	C/Yamagata/33/2014		Import from public-domain	Tanaka,S.; Aoki,Y.; Matoba,Y.; Yahagi,K.; Mizuta,K.; Itagaki,T.; Katsushima,F.; Katsushima,Y.; Matsuzaki,Y.; Sugawara,K.;
EPI813777	PB2	Japan	2014-Jul-22	EPI_ISL_182757	C/Yamagata/33/2014		Import from public-domain	Tanaka,S.; Aoki,Y.; Matoba,Y.; Yahagi,K.; Mizuta,K.; Itagaki,T.; Katsushima,F.; Katsushima,Y.; Matsuzaki,Y.; Sugawara,K.;
EPI603687	HE	Japan	2014-Aug-20	EPI_ISL_182758	C/Yamagata/35/2014		Import from public-domain	Tanaka,S.; Aoki,Y.; Matoba,Y.; Yahagi,K.; Mizuta,K.; Itagaki,T.; Katsushima,F.; Katsushima,Y.; Matsuzaki,Y.; Sugawara,K.;
EPI816730	MP	Japan	2014-Aug-20	EPI_ISL_182758	C/Yamagata/35/2014		Import from public-domain	Tanaka,S.; Aoki,Y.; Matoba,Y.; Yahagi,K.; Mizuta,K.; Itagaki,T.; Katsushima,F.; Katsushima,Y.; Matsuzaki,Y.; Sugawara,K.;
EPI816662	NP	Japan	2014-Aug-20	EPI_ISL_182758	C/Yamagata/35/2014		Import from public-domain	Tanaka,S.; Aoki,Y.; Matoba,Y.; Yahagi,K.; Mizuta,K.; Itagaki,T.; Katsushima,F.; Katsushima,Y.; Matsuzaki,Y.; Sugawara,K.;
EPI816799	NS	Japan	2014-Aug-20	EPI_ISL_182758	C/Yamagata/35/2014		Import from public-domain	Tanaka,S.; Aoki,Y.; Matoba,Y.; Yahagi,K.; Mizuta,K.; Itagaki,T.; Katsushima,F.; Katsushima,Y.; Matsuzaki,Y.; Sugawara,K.;
EPI816563	P3	Japan	2014-Aug-20	EPI_ISL_182758	C/Yamagata/35/2014		Import from public-domain	Tanaka,S.; Aoki,Y.; Matoba,Y.; Yahagi,K.; Mizuta,K.; Itagaki,T.; Katsushima,F.; Katsushima,Y.; Matsuzaki,Y.; Sugawara,K.;
EPI816480	PB1	Japan	2014-Aug-20	EPI_ISL_182758	C/Yamagata/35/2014		Import from public-domain	Tanaka,S.; Aoki,Y.; Matoba,Y.; Yahagi,K.; Mizuta,K.; Itagaki,T.; Katsushima,F.; Katsushima,Y.; Matsuzaki,Y.; Sugawara,K.;
EPI813778	PB2	Japan	2014-Aug-20	EPI_ISL_182758	C/Yamagata/35/2014		Import from public-domain	Tanaka,S.; Aoki,Y.; Matoba,Y.; Yahagi,K.; Mizuta,K.; Itagaki,T.; Katsushima,F.; Katsushima,Y.; Matsuzaki,Y.; Sugawara,K.;
EPI816717	MP	Japan	2012-Feb-22	EPI_ISL_230284	C/Yamagata/4/2012		Import from public-domain	Matsuzaki,Y.; Sugawara,K.; Furuse,Y.; Shimotai,Y.; Hongo,S.; Oshitani,H.; Mizuta,K.; Nishimura,H.
EPI816649	NP	Japan	2012-Feb-22	EPI_ISL_230284	C/Yamagata/4/2012		Import from public-domain	Matsuzaki,Y.; Sugawara,K.; Furuse,Y.; Shimotai,Y.; Hongo,S.; Oshitani,H.; Mizuta,K.; Nishimura,H.
EPI816786	NS	Japan	2012-Feb-22	EPI_ISL_230284	C/Yamagata/4/2012		Import from public-domain	Matsuzaki,Y.; Sugawara,K.; Furuse,Y.; Shimotai,Y.; Hongo,S.; Oshitani,H.; Mizuta,K.; Nishimura,H.
EPI816550	P3	Japan	2012-Feb-22	EPI_ISL_230284	C/Yamagata/4/2012		Import from public-domain	Matsuzaki,Y.; Sugawara,K.; Furuse,Y.; Shimotai,Y.; Hongo,S.; Oshitani,H.; Mizuta,K.; Nishimura,H.
EPI816446	PB1	Japan	2012-Feb-22	EPI_ISL_230284	C/Yamagata/4/2012		Import from public-domain	Matsuzaki,Y.; Sugawara,K.; Furuse,Y.; Shimotai,Y.; Hongo,S.; Oshitani,H.; Mizuta,K.; Nishimura,H.
EPI813756	PB2	Japan	2012-Feb-22	EPI_ISL_230284	C/Yamagata/4/2012		Import from public-domain	Matsuzaki,Y.; Sugawara,K.; Furuse,Y.; Shimotai,Y.; Hongo,S.; Oshitani,H.; Mizuta,K.; Nishimura,H.
EPI231964	HE	Japan	1988-Jan-01	EPI_ISL_66423	C/Yamagata/4/88		Import from public-domain	Umetsu,Y.
EPI231965	HE	Japan	1989-Jan-01	EPI_ISL_66424	C/Yamagata/4/89		Import from public-domain	Asahi,Y.; Sugawara,K.; Muraki,Y.; Hongo,S.; Kitame,F.; Nakamura,K.
EPI816674	MP	Japan	1992-Nov-19	EPI_ISL_230280	C/Yamagata/4/92		Import from public-domain	Matsuzaki,Y.; Sugawara,K.; Furuse,Y.; Shimotai,Y.; Hongo,S.; Oshitani,H.; Mizuta,K.; Nishimura,H.
EPI816595	NP	Japan	1992-Nov-19	EPI_ISL_230280	C/Yamagata/4/92		Import from public-domain	Matsuzaki,Y.; Sugawara,K.; Furuse,Y.; Shimotai,Y.; Hongo,S.; Oshitani,H.; Mizuta,K.; Nishimura,H.
EPI816744	NS	Japan	1992-Nov-19	EPI_ISL_230280	C/Yamagata/4/92		Import from public-domain	Matsuzaki,Y.; Sugawara,K.; Furuse,Y.; Shimotai,Y.; Hongo,S.; Oshitani,H.; Mizuta,K.; Nishimura,H.
EPI816496	P3	Japan	1992-Nov-19	EPI_ISL_230280	C/Yamagata/4/92		Import from public-domain	Matsuzaki,Y.; Sugawara,K.; Furuse,Y.; Shimotai,Y.; Hongo,S.; Oshitani,H.; Mizuta,K.; Nishimura,H.
EPI813795	PB1	Japan	1992-Nov-19	EPI_ISL_230280	C/Yamagata/4/92		Import from public-domain	Matsuzaki,Y.; Sugawara,K.; Furuse,Y.; Shimotai,Y.; Hongo,S.; Oshitani,H.; Mizuta,K.; Nishimura,H.
EPI813698	PB2	Japan	1992-Nov-19	EPI_ISL_230280	C/Yamagata/4/92		Import from public-domain	Matsuzaki,Y.; Sugawara,K.; Furuse,Y.; Shimotai,Y.; Hongo,S.; Oshitani,H.; Mizuta,K.; Nishimura,H.
EPI813652	HE	Japan	2002-Feb-07	EPI_ISL_230237	C/Yamagata/5/2002		Import from public-domain	Matsuzaki,Y.; Sugawara,K.; Furuse,Y.; Shimotai,Y.; Hongo,S.; Oshitani,H.; Mizuta,K.; Nishimura,H.
EPI816676	MP	Japan	2002-Feb-07	EPI_ISL_230237	C/Yamagata/5/2002		Import from public-domain	Matsuzaki,Y.; Sugawara,K.; Furuse,Y.; Shimotai,Y.; Hongo,S.; Oshitani,H.; Mizuta,K.; Nishimura,H.
EPI816613	NP	Japan	2002-Feb-07	EPI_ISL_230237	C/Yamagata/5/2002		Import from public-domain	Matsuzaki,Y.; Sugawara,K.; Furuse,Y.; Shimotai,Y.; Hongo,S.; Oshitani,H.; Mizuta,K.; Nishimura,H.
EPI816745	NS	Japan	2002-Feb-07	EPI_ISL_230237	C/Yamagata/5/2002		Import from public-domain	Matsuzaki,Y.; Sugawara,K.; Furuse,Y.; Shimotai,Y.; Hongo,S.; Oshitani,H.; Mizuta,K.; Nishimura,H.
EPI816509	P3	Japan	2002-Feb-07	EPI_ISL_230237	C/Yamagata/5/2002		Import from public-domain	Matsuzaki,Y.; Sugawara,K.; Furuse,Y.; Shimotai,Y.; Hongo,S.; Oshitani,H.; Mizuta,K.; Nishimura,H.
EPI813796	PB1	Japan	2002-Feb-07	EPI_ISL_230237	C/Yamagata/5/2002		Import from public-domain	Matsuzaki,Y.; Sugawara,K.; Furuse,Y.; Shimotai,Y.; Hongo,S.; Oshitani,H.; Mizuta,K.; Nishimura,H.
EPI813699	PB2	Japan	2002-Feb-07	EPI_ISL_230237	C/Yamagata/5/2002		Import from public-domain	Matsuzaki,Y.; Sugawara,K.; Furuse,Y.; Shimotai,Y.; Hongo,S.; Oshitani,H.; Mizuta,K.; Nishimura,H.
EPI231570	NS	Japan	1989-Jan-01	EPI_ISL_66359	C/Yamagata/5/89		Import from public-domain	Matsuzaki,Y.
EPI232176	HE	Japan	1992-Nov-26	EPI_ISL_66361	C/Yamagata/5/92		Import from public-domain	Matsuzaki,Y.; Sugawara,K.; Furuse,Y.; Shimotai,Y.; Hongo,S.; Oshitani,H.; Mizuta,K.; Nishimura,H.

Segment ID	Segment	Country	Collection date	Isolate-ID	Isolate name	Originating Lab	Submitting Lab	Authors
EPI816675	MP	Japan	1992-Nov-26	EPI_ISL_66361	C/Yamagata/5/92		Import from public-domain	Matsuzaki,Y.; Sugawara,K.; Furuse,Y.; Shimotai,Y.; Hongo,S.; Oshitani,H.; Mizuta,K.; Nishimura,H.
EPI816596	NP	Japan	1992-Nov-26	EPI_ISL_66361	C/Yamagata/5/92		Import from public-domain	Matsuzaki,Y.; Sugawara,K.; Furuse,Y.; Shimotai,Y.; Hongo,S.; Oshitani,H.; Mizuta,K.; Nishimura,H.
EPI231572	NS	Japan	1992-Nov-26	EPI_ISL_66361	C/Yamagata/5/92		Import from public-domain	Matsuzaki,Y.; Sugawara,K.; Furuse,Y.; Shimotai,Y.; Hongo,S.; Oshitani,H.; Mizuta,K.; Nishimura,H.
EPI816497	P3	Japan	1992-Nov-26	EPI_ISL_66361	C/Yamagata/5/92		Import from public-domain	Matsuzaki,Y.; Sugawara,K.; Furuse,Y.; Shimotai,Y.; Hongo,S.; Oshitani,H.; Mizuta,K.; Nishimura,H.
EPI816414	PB1	Japan	1992-Nov-26	EPI_ISL_66361	C/Yamagata/5/92		Import from public-domain	Matsuzaki,Y.; Sugawara,K.; Furuse,Y.; Shimotai,Y.; Hongo,S.; Oshitani,H.; Mizuta,K.; Nishimura,H.
EPI813740	PB2	Japan	1992-Nov-26	EPI_ISL_66361	C/Yamagata/5/92		Import from public-domain	Matsuzaki,Y.; Sugawara,K.; Furuse,Y.; Shimotai,Y.; Hongo,S.; Oshitani,H.; Mizuta,K.; Nishimura,H.
EPI231971	HE	Japan	2000-Mar-21	EPI_ISL_66425	C/Yamagata/6/2000		Import from public-domain	Matsuzaki,Y.; Sugawara,K.; Furuse,Y.; Shimotai,Y.; Hongo,S.; Oshitani,H.; Mizuta,K.; Nishimura,H.
EPI231968	MP	Japan	2000-Mar-21	EPI_ISL_66425	C/Yamagata/6/2000		Import from public-domain	Matsuzaki,Y.; Sugawara,K.; Furuse,Y.; Shimotai,Y.; Hongo,S.; Oshitani,H.; Mizuta,K.; Nishimura,H.
EPI816612	NP	Japan	2000-Mar-21	EPI_ISL_66425	C/Yamagata/6/2000		Import from public-domain	Matsuzaki,Y.; Sugawara,K.; Furuse,Y.; Shimotai,Y.; Hongo,S.; Oshitani,H.; Mizuta,K.; Nishimura,H.
EPI231972	NS	Japan	2000-Mar-21	EPI_ISL_66425	C/Yamagata/6/2000		Import from public-domain	Matsuzaki,Y.; Sugawara,K.; Furuse,Y.; Shimotai,Y.; Hongo,S.; Oshitani,H.; Mizuta,K.; Nishimura,H.
EPI816508	P3	Japan	2000-Mar-21	EPI_ISL_66425	C/Yamagata/6/2000		Import from public-domain	Matsuzaki,Y.; Sugawara,K.; Furuse,Y.; Shimotai,Y.; Hongo,S.; Oshitani,H.; Mizuta,K.; Nishimura,H.
EPI816425	PB1	Japan	2000-Mar-21	EPI_ISL_66425	C/Yamagata/6/2000		Import from public-domain	Matsuzaki,Y.; Sugawara,K.; Furuse,Y.; Shimotai,Y.; Hongo,S.; Oshitani,H.; Mizuta,K.; Nishimura,H.
EPI813735	PB2	Japan	2000-Mar-21	EPI_ISL_66425	C/Yamagata/6/2000		Import from public-domain	Matsuzaki,Y.; Sugawara,K.; Furuse,Y.; Shimotai,Y.; Hongo,S.; Oshitani,H.; Mizuta,K.; Nishimura,H.
EPI603679	HE	Japan	2014-Apr-16	EPI_ISL_182750	C/Yamagata/6/2014		Import from public-domain	Tanaka,S.; Aoki,Y.; Matoba,Y.; Yahagi,K.; Mizuta,K.; Itagaki,T.; Katsushima,F.; Katsushima,Y.; Matsuzaki,Y.; Sugawara,K.;
EPI816724	MP	Japan	2014-Apr-16	EPI_ISL_182750	C/Yamagata/6/2014		Import from public-domain	Tanaka,S.; Aoki,Y.; Matoba,Y.; Yahagi,K.; Mizuta,K.; Itagaki,T.; Katsushima,F.; Katsushima,Y.; Matsuzaki,Y.; Sugawara,K.;
EPI816656	NP	Japan	2014-Apr-16	EPI_ISL_182750	C/Yamagata/6/2014		Import from public-domain	Tanaka,S.; Aoki,Y.; Matoba,Y.; Yahagi,K.; Mizuta,K.; Itagaki,T.; Katsushima,F.; Katsushima,Y.; Matsuzaki,Y.; Sugawara,K.;
EPI816793	NS	Japan	2014-Apr-16	EPI_ISL_182750	C/Yamagata/6/2014		Import from public-domain	Tanaka,S.; Aoki,Y.; Matoba,Y.; Yahagi,K.; Mizuta,K.; Itagaki,T.; Katsushima,F.; Katsushima,Y.; Matsuzaki,Y.; Sugawara,K.;
EPI816557	P3	Japan	2014-Apr-16	EPI_ISL_182750	C/Yamagata/6/2014		Import from public-domain	Tanaka,S.; Aoki,Y.; Matoba,Y.; Yahagi,K.; Mizuta,K.; Itagaki,T.; Katsushima,F.; Katsushima,Y.; Matsuzaki,Y.; Sugawara,K.;
EPI816474	PB1	Japan	2014-Apr-16	EPI_ISL_182750	C/Yamagata/6/2014		Import from public-domain	Tanaka,S.; Aoki,Y.; Matoba,Y.; Yahagi,K.; Mizuta,K.; Itagaki,T.; Katsushima,F.; Katsushima,Y.; Matsuzaki,Y.; Sugawara,K.;
EPI813772	PB2	Japan	2014-Apr-16	EPI_ISL_182750	C/Yamagata/6/2014		Import from public-domain	Tanaka,S.; Aoki,Y.; Matoba,Y.; Yahagi,K.; Mizuta,K.; Itagaki,T.; Katsushima,F.; Katsushima,Y.; Matsuzaki,Y.; Sugawara,K.;
EPI231978	HE	Japan	1998-Jan-01	EPI_ISL_66426	C/Yamagata/6/98		Import from public-domain	Matsuzaki,Y.
EPI231975	MP	Japan	1998-Jan-01	EPI_ISL_66426	C/Yamagata/6/98		Import from public-domain	Matsuzaki,Y.
EPI231976	NS	Japan	1998-Jan-01	EPI_ISL_66426	C/Yamagata/6/98		Import from public-domain	Matsuzaki,Y.
EPI232180	HE	Japan	1964-Jan-01	EPI_ISL_66319	C/Yamagata/64		Import from public-domain	Matsuzaki,Y.; Sugawara,K.; Furuse,Y.; Shimotai,Y.; Hongo,S.; Oshitani,H.; Mizuta,K.; Nishimura,H.
EPI232179	MP	Japan	1964-Jan-01	EPI_ISL_66319	C/Yamagata/64		Import from public-domain	Matsuzaki,Y.; Sugawara,K.; Furuse,Y.; Shimotai,Y.; Hongo,S.; Oshitani,H.; Mizuta,K.; Nishimura,H.
EPI816571	NP	Japan	1964-Jan-01	EPI_ISL_66319	C/Yamagata/64		Import from public-domain	Matsuzaki,Y.; Sugawara,K.; Furuse,Y.; Shimotai,Y.; Hongo,S.; Oshitani,H.; Mizuta,K.; Nishimura,H.
EPI231517	NS	Japan	1964-Jan-01	EPI_ISL_66319	C/Yamagata/64		Import from public-domain	Matsuzaki,Y.; Sugawara,K.; Furuse,Y.; Shimotai,Y.; Hongo,S.; Oshitani,H.; Mizuta,K.; Nishimura,H.
EPI816460	P3	Japan	1964-Jan-01	EPI_ISL_66319	C/Yamagata/64		Import from public-domain	Matsuzaki,Y.; Sugawara,K.; Furuse,Y.; Shimotai,Y.; Hongo,S.; Oshitani,H.; Mizuta,K.; Nishimura,H.
EPI813779	PB1	Japan	1964-Jan-01	EPI_ISL_66319	C/Yamagata/64		Import from public-domain	Matsuzaki,Y.; Sugawara,K.; Furuse,Y.; Shimotai,Y.; Hongo,S.; Oshitani,H.; Mizuta,K.; Nishimura,H.
EPI814696	PB2	Japan	1964-Jan-01	EPI_ISL_66319	C/Yamagata/64		Import from public-domain	Matsuzaki,Y.; Sugawara,K.; Furuse,Y.; Shimotai,Y.; Hongo,S.; Oshitani,H.; Mizuta,K.; Nishimura,H.
EPI816677	MP	Japan	2002-Feb-16	EPI_ISL_230281	C/Yamagata/7/2002		Import from public-domain	Matsuzaki,Y.; Sugawara,K.; Furuse,Y.; Shimotai,Y.; Hongo,S.; Oshitani,H.; Mizuta,K.; Nishimura,H.
EPI816614	NP	Japan	2002-Feb-16	EPI_ISL_230281	C/Yamagata/7/2002		Import from public-domain	Matsuzaki,Y.; Sugawara,K.; Furuse,Y.; Shimotai,Y.; Hongo,S.; Oshitani,H.; Mizuta,K.; Nishimura,H.
EPI816746	NS	Japan	2002-Feb-16	EPI_ISL_230281	C/Yamagata/7/2002		Import from public-domain	Matsuzaki,Y.; Sugawara,K.; Furuse,Y.; Shimotai,Y.; Hongo,S.; Oshitani,H.; Mizuta,K.; Nishimura,H.

Segment ID	Segment	Country	Collection date	Isolate-ID	Isolate name	Originating Lab	Submitting Lab	Authors
EPI816510	P3	Japan	2002-Feb-16	EPI_ISL_230281	C/Yamagata/7/2002		Import from public-domain	Matsuzaki,Y.; Sugawara,K.; Furuse,Y.; Shimotai,Y.; Hongo,S.; Oshitani,H.; Mizuta,K.; Nishimura,H.
EPI813797	PB1	Japan	2002-Feb-16	EPI_ISL_230281	C/Yamagata/7/2002		Import from public-domain	Matsuzaki,Y.; Sugawara,K.; Furuse,Y.; Shimotai,Y.; Hongo,S.; Oshitani,H.; Mizuta,K.; Nishimura,H.
EPI813700	PB2	Japan	2002-Feb-16	EPI_ISL_230281	C/Yamagata/7/2002		Import from public-domain	Matsuzaki,Y.; Sugawara,K.; Furuse,Y.; Shimotai,Y.; Hongo,S.; Oshitani,H.; Mizuta,K.; Nishimura,H.
EPI813683	HE	Japan	2012-Mar-14	EPI_ISL_230268	C/Yamagata/7/2012		Import from public-domain	Matsuzaki,Y.; Sugawara,K.; Furuse,Y.; Shimotai,Y.; Hongo,S.; Oshitani,H.; Mizuta,K.; Nishimura,H.
EPI816718	MP	Japan	2012-Mar-14	EPI_ISL_230268	C/Yamagata/7/2012		Import from public-domain	Matsuzaki,Y.; Sugawara,K.; Furuse,Y.; Shimotai,Y.; Hongo,S.; Oshitani,H.; Mizuta,K.; Nishimura,H.
EPI816650	NP	Japan	2012-Mar-14	EPI_ISL_230268	C/Yamagata/7/2012		Import from public-domain	Matsuzaki,Y.; Sugawara,K.; Furuse,Y.; Shimotai,Y.; Hongo,S.; Oshitani,H.; Mizuta,K.; Nishimura,H.
EPI816787	NS	Japan	2012-Mar-14	EPI_ISL_230268	C/Yamagata/7/2012		Import from public-domain	Matsuzaki,Y.; Sugawara,K.; Furuse,Y.; Shimotai,Y.; Hongo,S.; Oshitani,H.; Mizuta,K.; Nishimura,H.
EPI816551	P3	Japan	2012-Mar-14	EPI_ISL_230268	C/Yamagata/7/2012		Import from public-domain	Matsuzaki,Y.; Sugawara,K.; Furuse,Y.; Shimotai,Y.; Hongo,S.; Oshitani,H.; Mizuta,K.; Nishimura,H.
EPI816447	PB1	Japan	2012-Mar-14	EPI_ISL_230268	C/Yamagata/7/2012		Import from public-domain	Matsuzaki,Y.; Sugawara,K.; Furuse,Y.; Shimotai,Y.; Hongo,S.; Oshitani,H.; Mizuta,K.; Nishimura,H.
EPI813757	PB2	Japan	2012-Mar-14	EPI_ISL_230268	C/Yamagata/7/2012		Import from public-domain	Matsuzaki,Y.; Sugawara,K.; Furuse,Y.; Shimotai,Y.; Hongo,S.; Oshitani,H.; Mizuta,K.; Nishimura,H.
EPI816672	MP	Japan	1981-Mar-18	EPI_ISL_230278	C/Yamagata/7/81		Import from public-domain	Matsuzaki,Y.; Sugawara,K.; Furuse,Y.; Shimotai,Y.; Hongo,S.; Oshitani,H.; Mizuta,K.; Nishimura,H.
EPI816584	NP	Japan	1981-Mar-18	EPI_ISL_230278	C/Yamagata/7/81		Import from public-domain	Matsuzaki,Y.; Sugawara,K.; Furuse,Y.; Shimotai,Y.; Hongo,S.; Oshitani,H.; Mizuta,K.; Nishimura,H.
EPI816741	NS	Japan	1981-Mar-18	EPI_ISL_230278	C/Yamagata/7/81		Import from public-domain	Matsuzaki,Y.; Sugawara,K.; Furuse,Y.; Shimotai,Y.; Hongo,S.; Oshitani,H.; Mizuta,K.; Nishimura,H.
EPI816485	P3	Japan	1981-Mar-18	EPI_ISL_230278	C/Yamagata/7/81		Import from public-domain	Matsuzaki,Y.; Sugawara,K.; Furuse,Y.; Shimotai,Y.; Hongo,S.; Oshitani,H.; Mizuta,K.; Nishimura,H.
EPI813792	PB1	Japan	1981-Mar-18	EPI_ISL_230278	C/Yamagata/7/81		Import from public-domain	Matsuzaki,Y.; Sugawara,K.; Furuse,Y.; Shimotai,Y.; Hongo,S.; Oshitani,H.; Mizuta,K.; Nishimura,H.
EPI813695	PB2	Japan	1981-Mar-18	EPI_ISL_230278	C/Yamagata/7/81		Import from public-domain	Matsuzaki,Y.; Sugawara,K.; Furuse,Y.; Shimotai,Y.; Hongo,S.; Oshitani,H.; Mizuta,K.; Nishimura,H.
EPI231558	HE	Japan	1988-Nov-03	EPI_ISL_66349	C/Yamagata/7/88		Import from public-domain	Matsuzaki,Y.; Sugawara,K.; Furuse,Y.; Shimotai,Y.; Hongo,S.; Oshitani,H.; Mizuta,K.; Nishimura,H.
EPI231980	MP	Japan	1988-Nov-03	EPI_ISL_66349	C/Yamagata/7/88		Import from public-domain	Matsuzaki,Y.; Sugawara,K.; Furuse,Y.; Shimotai,Y.; Hongo,S.; Oshitani,H.; Mizuta,K.; Nishimura,H.
EPI816591	NP	Japan	1988-Nov-03	EPI_ISL_66349	C/Yamagata/7/88		Import from public-domain	Matsuzaki,Y.; Sugawara,K.; Furuse,Y.; Shimotai,Y.; Hongo,S.; Oshitani,H.; Mizuta,K.; Nishimura,H.
EPI231563	NS	Japan	1988-Nov-03	EPI_ISL_66349	C/Yamagata/7/88		Import from public-domain	Matsuzaki,Y.; Sugawara,K.; Furuse,Y.; Shimotai,Y.; Hongo,S.; Oshitani,H.; Mizuta,K.; Nishimura,H.
EPI816492	P3	Japan	1988-Nov-03	EPI_ISL_66349	C/Yamagata/7/88		Import from public-domain	Matsuzaki,Y.; Sugawara,K.; Furuse,Y.; Shimotai,Y.; Hongo,S.; Oshitani,H.; Mizuta,K.; Nishimura,H.
EPI816410	PB1	Japan	1988-Nov-03	EPI_ISL_66349	C/Yamagata/7/88		Import from public-domain	Matsuzaki,Y.; Sugawara,K.; Furuse,Y.; Shimotai,Y.; Hongo,S.; Oshitani,H.; Mizuta,K.; Nishimura,H.
EPI232182	PB2	Japan	1988-Nov-03	EPI_ISL_66349	C/Yamagata/7/88		Import from public-domain	Matsuzaki,Y.; Sugawara,K.; Furuse,Y.; Shimotai,Y.; Hongo,S.; Oshitani,H.; Mizuta,K.; Nishimura,H.
EPI813722	PB2	Japan	1988-Nov-03	EPI_ISL_66349	C/Yamagata/7/88		Import from public-domain	Matsuzaki,Y.; Sugawara,K.; Furuse,Y.; Shimotai,Y.; Hongo,S.; Oshitani,H.; Mizuta,K.; Nishimura,H.
EPI231981	HE	Japan	2000-Jan-01	EPI_ISL_66427	C/Yamagata/8/2000		Import from public-domain	Matsuzaki,Y.
EPI231984	MP	Japan	2000-Jan-01	EPI_ISL_66427	C/Yamagata/8/2000		Import from public-domain	Matsuzaki,Y.
EPI231987	NS	Japan	2000-Jan-01	EPI_ISL_66427	C/Yamagata/8/2000		Import from public-domain	Matsuzaki,Y.
EPI231559	HE	Japan	1988-Jan-01	EPI_ISL_66350	C/Yamagata/8/88		Import from public-domain	Matsuzaki,Y.
EPI232187	MP	Japan	1988-Jan-01	EPI_ISL_66350	C/Yamagata/8/88		Import from public-domain	Matsuzaki,Y.
EPI231991	HE	Japan	1996-Jun-13	EPI_ISL_66428	C/Yamagata/8/96		Import from public-domain	Matsuzaki,Y.
EPI231994	MP	Japan	1996-Jun-13	EPI_ISL_66428	C/Yamagata/8/96		Import from public-domain	Matsuzaki,Y.
EPI816609	NP	Japan	1996-Jun-13	EPI_ISL_66428	C/Yamagata/8/96		Import from public-domain	Matsuzaki,Y.
EPI231989	NS	Japan	1996-Jun-13	EPI_ISL_66428	C/Yamagata/8/96		Import from public-domain	Matsuzaki,Y.

Segment ID	Segment	Country	Collection date	Isolate-ID	Isolate name	Originating Lab	Submitting Lab	Authors
EPI816504	P3	Japan	1996-Jun-13	EPI_ISL_66428	C/Yamagata/8/96		Import from public-domain	Matsuzaki,Y.
EPI816421	PB1	Japan	1996-Jun-13	EPI_ISL_66428	C/Yamagata/8/96		Import from public-domain	Matsuzaki,Y.
EPI813723	PB2	Japan	1996-Jun-13	EPI_ISL_66428	C/Yamagata/8/96		Import from public-domain	Matsuzaki,Y.
EPI232000	HE	Japan	2000-Jan-01	EPI_ISL_66429	C/Yamagata/9/2000		Import from public-domain	Matsuzaki,Y.
EPI231996	MP	Japan	2000-Jan-01	EPI_ISL_66429	C/Yamagata/9/2000		Import from public-domain	Matsuzaki,Y.
EPI232001	NS	Japan	2000-Jan-01	EPI_ISL_66429	C/Yamagata/9/2000		Import from public-domain	Matsuzaki,Y.
EPI813670	HE	Japan	2006-Jun-13	EPI_ISL_230255	C/Yamagata/9/2006		Import from public-domain	Matsuzaki,Y.; Sugawara,K.; Furuse,Y.; Shimotai,Y.; Hongo,S.; Oshitani,H.; Mizuta,K.; Nishimura,H.
EPI816701	MP	Japan	2006-Jun-13	EPI_ISL_230255	C/Yamagata/9/2006		Import from public-domain	Matsuzaki,Y.; Sugawara,K.; Furuse,Y.; Shimotai,Y.; Hongo,S.; Oshitani,H.; Mizuta,K.; Nishimura,H.
EPI816635	NP	Japan	2006-Jun-13	EPI_ISL_230255	C/Yamagata/9/2006		Import from public-domain	Matsuzaki,Y.; Sugawara,K.; Furuse,Y.; Shimotai,Y.; Hongo,S.; Oshitani,H.; Mizuta,K.; Nishimura,H.
EPI816770	NS	Japan	2006-Jun-13	EPI_ISL_230255	C/Yamagata/9/2006		Import from public-domain	Matsuzaki,Y.; Sugawara,K.; Furuse,Y.; Shimotai,Y.; Hongo,S.; Oshitani,H.; Mizuta,K.; Nishimura,H.
EPI816534	P3	Japan	2006-Jun-13	EPI_ISL_230255	C/Yamagata/9/2006		Import from public-domain	Matsuzaki,Y.; Sugawara,K.; Furuse,Y.; Shimotai,Y.; Hongo,S.; Oshitani,H.; Mizuta,K.; Nishimura,H.
EPI816432	PB1	Japan	2006-Jun-13	EPI_ISL_230255	C/Yamagata/9/2006		Import from public-domain	Matsuzaki,Y.; Sugawara,K.; Furuse,Y.; Shimotai,Y.; Hongo,S.; Oshitani,H.; Mizuta,K.; Nishimura,H.
EPI813719	PB2	Japan	2006-Jun-13	EPI_ISL_230255	C/Yamagata/9/2006		Import from public-domain	Matsuzaki,Y.; Sugawara,K.; Furuse,Y.; Shimotai,Y.; Hongo,S.; Oshitani,H.; Mizuta,K.; Nishimura,H.
EPI231560	HE	Japan	1988-Dec-12	EPI_ISL_66351	C/Yamagata/9/88		Import from public-domain	Matsuzaki,Y.; Sugawara,K.; Furuse,Y.; Shimotai,Y.; Hongo,S.; Oshitani,H.; Mizuta,K.; Nishimura,H.
EPI232188	MP	Japan	1988-Dec-12	EPI_ISL_66351	C/Yamagata/9/88		Import from public-domain	Matsuzaki,Y.; Sugawara,K.; Furuse,Y.; Shimotai,Y.; Hongo,S.; Oshitani,H.; Mizuta,K.; Nishimura,H.
EPI816592	NP	Japan	1988-Dec-12	EPI_ISL_66351	C/Yamagata/9/88		Import from public-domain	Matsuzaki,Y.; Sugawara,K.; Furuse,Y.; Shimotai,Y.; Hongo,S.; Oshitani,H.; Mizuta,K.; Nishimura,H.
EPI231564	NS	Japan	1988-Dec-12	EPI_ISL_66351	C/Yamagata/9/88		Import from public-domain	Matsuzaki,Y.; Sugawara,K.; Furuse,Y.; Shimotai,Y.; Hongo,S.; Oshitani,H.; Mizuta,K.; Nishimura,H.
EPI816493	P3	Japan	1988-Dec-12	EPI_ISL_66351	C/Yamagata/9/88		Import from public-domain	Matsuzaki,Y.; Sugawara,K.; Furuse,Y.; Shimotai,Y.; Hongo,S.; Oshitani,H.; Mizuta,K.; Nishimura,H.
EPI816411	PB1	Japan	1988-Dec-12	EPI_ISL_66351	C/Yamagata/9/88		Import from public-domain	Matsuzaki,Y.; Sugawara,K.; Furuse,Y.; Shimotai,Y.; Hongo,S.; Oshitani,H.; Mizuta,K.; Nishimura,H.
EPI813725	PB2	Japan	1988-Dec-12	EPI_ISL_66351	C/Yamagata/9/88		Import from public-domain	Matsuzaki,Y.; Sugawara,K.; Furuse,Y.; Shimotai,Y.; Hongo,S.; Oshitani,H.; Mizuta,K.; Nishimura,H.
EPI232005	HE	Japan	1996-Jan-01	EPI_ISL_66430	C/Yamagata/9/96		Import from public-domain	Matsuzaki,Y.
EPI232002	MP	Japan	1996-Jan-01	EPI_ISL_66430	C/Yamagata/9/96		Import from public-domain	Matsuzaki,Y.
EPI232007	NS	Japan	1996-Jan-01	EPI_ISL_66430	C/Yamagata/9/96		Import from public-domain	Matsuzaki,Y.

All influenza C gene sequences from cases of human infection were downloaded from GISAID on 2019-06-30 and aligned on a gene-specific basis using BioEdit. Those with full-length open reading frames were used in phylogenetic analyses. [a]C/England/892/1983, C/England/892/83 and C/England/83 identified as C/England/892/83 in Figures 2 and S2; [b]C/Great Lakes/1167/1954 and C/Great Lakes/1167/54 identified as C/Great Lakes/1167/54 in Figures 2 and S2; [c]C/Greece/1/79 and C/Greece/79 identified as C/Greece/1/79 in Figures 2 and S2; [d]C/JJ/1950 and C/JJ/50 identified as C/JJ/50 in Figures 2 and S2; [e]C/New Jersey/1/76 and C/NewJersey/76 identified as C/New Jersey/1/76 in Figures 2 and S2; [f]C/Taylor/1233/1947 and C/Taylor/1233/47 identified as C/Taylor/1233/47 in Figures 2 and S2; [g]C/Yamagata/10/1981 and C/Yamagata/10/81 identified as C/Yamagata/10/81 in Figures 2 and S2. We gratefully acknowledge the authors, originating and submitting laboratories of the sequences from GISAID's EpiFlu™ Database on which this research is based. The list is detailed above. All submitters of data may be contacted directly via https://www.gisaid.org/
